# Supplementary material for: Contrasting Epidemiology and Population Genetics of COVID-19 Infections Defined by Multilocus Genotypes in SARS-CoV-2 Genomes Sampled Globally
Source: Viruses. 2022 Jun 29;14(7):1434. doi: 10.3390/v14071434 (PMC9316073; doi:10.3390/v14071434)
Supplement: Supplementary file 1 [file viruses-14-01434-s001.zip › Chan et al Table S5_ver03-05-22.pdf]

MLG (2020)_ID	Continent	M	N	NSP1	NSP2	NSP3	NSP4	NSP5	NSP8	NSP12	NSP13	NSP14	ORF3a	ORF8	Spike	MLG	Freq	%
AF (2020)_MLG1	Africa	DT	PDSSSSSKRI	DR	TPIVGLSQIPA	SPEDGTASTATTVQM	FGG	AL	YQ	AL	SPYV	LFTFV	QARSGG	SVL	LVSGGGV	DTPDSSSSSKRIDRTPIVGLSQIPASPEDGTASTATTVQMFGGALYQALSPYVLFTFVQARSGGSVLLVSGGGV	91	15.10
AF (2020)_MLG2	Africa	DT	PDSSSSSRGI	DR	TPIVGLSQIPA	SPEDGTASTATTVQM	FGG	AL	YQ	AL	SPYV	LFTFV	QARSGG	SVL	LVSGGGV	DTPDSSSSSRGIDRTPIVGLSQIPASPEDGTASTATTVQMFGGALYQALSPYVLFTFVQARSGGSVLLVSGGGV	75	12.50
AF (2020)_MLG3	Africa	DT	PDSSSSSKRI	DR	TPIVGLSQVPA	SPEDGTASTATTVQM	FGG	AL	YQ	AL	SPYV	LFTFV	QARSGG	SVL	LVSGGGV	DTPDSSSSSKRIDRTPIVGLSQVPASPEDGTASTATTVQMFGGALYQALSPYVLFTFVQARSGGSVLLVSGGGV	58	9.70
AF (2020)_MLG4	Africa	DT	PDSSSSSKRI	DR	TPIVGLSQIPA	SPEDGTASTATTVQM	FGG	AL	HQ	AL	SPYV	LFTFV	QASSGG	SVL	LVSGGGV	DTPDSSSSSKRIDRTPIVGLSQIPASPEDGTASTATTVQMFGGALHQALSPYVLFTFVQASSGGSVLLVSGGGV	30	5.00
AF (2020)_MLG5	Africa	DT	PDSLSSSRGI	DR	TPIVGFSQIPA	SPEDGTASTATTVQM	FGG	AL	YQ	AL	SPYV	LFTFV	QARFGG	SVL	LVSGGGV	DTPDSLSSSRGIDRTPIVGFSQIPASPEDGTASTATTVQMFGGALYQALSPYVLFTFVQARFGGSVLLVSGGGV	28	4.70
AF (2020)_MLG6	Africa	DT	PDSSSSSKRI	DR	TPIVGLSQVPA	SPEDGIASTATTVQM	FSG	AL	YQ	AL	SPYV	LFTFV	QARSGG	SVL	LVSGGGV	DTPDSSSSSKRIDRTPIVGLSQVPASPEDGIASTATTVQMFSGALYQALSPYVLFTFVQARSGGSVLLVSGGGV	26	4.30
AF (2020)_MLG7	Africa	GT	PDSSSSSRGI	DR	TPIVGLSQIPA	SPEDGTASTATTVQM	FGG	AL	YQ	AL	SPYV	LFTFV	QARSGG	SVL	LVSGGGV	GTPDSSSSSRGIDRTPIVGLSQIPASPEDGTASTATTVQMFGGALYQALSPYVLFTFVQARSGGSVLLVSGGGV	24	4.00
AF (2020)_MLG8	Africa	DT	PDSSSSSKRI	DR	TPIVGLSQIPA	SPEEGTASTATTVQM	FGG	AL	YQ	AL	SPYV	LFTFV	QARSGG	SVL	LVSGGGV	DTPDSSSSSKRIDRTPIVGLSQIPASPEEGTASTATTVQMFGGALYQALSPYVLFTFVQARSGGSVLLVSGGGV	22	3.70
AF (2020)_MLG9	Africa	DT	PDSSSSSKRI	DR	TPIVGLSQVPA	SPEDGTASTATTVQM	FGG	AL	HQ	AL	SPYV	LFTFV	QASSGG	SVL	LVSGGGV	DTPDSSSSSKRIDRTPIVGLSQVPASPEDGTASTATTVQMFGGALHQALSPYVLFTFVQASSGGSVLLVSGGGV	22	3.70
AF (2020)_MLG10	Africa	DT	PDSSSSSRGI	DR	TPIVGLSQIPA	SPEDGTASTATTVQM	FGG	AL	YQ	AL	SPYV	LFTFV	HARSGG	SVL	LVSGGGV	DTPDSSSSSRGIDRTPIVGLSQIPASPEDGTASTATTVQMFGGALYQALSPYVLFTFVHARSGGSVLLVSGGGV	19	3.20
AF (2020)_MLG11	Africa	DT	PDSSSSSKRI	DR	TPIVGLSQIPA	SPEDGTASTATTVQM	FGG	AL	HQ	AL	SPYV	LFTFV	QARSGG	SVL	LVSGGGV	DTPDSSSSSKRIDRTPIVGLSQIPASPEDGTASTATTVQMFGGALHQALSPYVLFTFVQARSGGSVLLVSGGGV	17	2.80
AF (2020)_MLG12	Africa	DT	PDSSSSSKRI	DR	TPIVGLSQIPA	SPEDGIASTATTVQM	FSG	AL	YQ	AL	SPYV	LFTFV	QARSGG	SVL	LVSGGGV	DTPDSSSSSKRIDRTPIVGLSQIPASPEDGIASTATTVQMFSGALYQALSPYVLFTFVQARSGGSVLLVSGGGV	16	2.70
AF (2020)_MLG13	Africa	DT	PDSSSSSKRI	DR	TPIVGLSQVPA	SPEDGTASTATTVQM	FGG	AL	HQ	AL	SPYV	LFTFV	QARSGG	SVL	LVSGGGV	DTPDSSSSSKRIDRTPIVGLSQVPASPEDGTASTATTVQMFGGALHQALSPYVLFTFVQARSGGSVLLVSGGGV	15	2.50
AF (2020)_MLG14	Africa	DT	PDSLSSSRGI	DR	TPIVGFSQIPA	SPEDGTASTATTVQM	FGG	AL	YQ	AL	SPYV	LFTFV	QARSGG	SVL	LVSGGGV	DTPDSLSSSRGIDRTPIVGFSQIPASPEDGTASTATTVQMFGGALYQALSPYVLFTFVQARSGGSVLLVSGGGV	13	2.20
AF (2020)_MLG15	Africa	DT	PDSSSSNRGI	DR	TPIVGLSQIPA	SPEDGTASTATTVQM	FGG	AL	YQ	AP	SPYV	LFTFV	QARSGG	SVS	LVSGDGV	DTPDSSSSNRGIDRTPIVGLSQIPASPEDGTASTATTVQMFGGALYQAPSPYVLFTFVQARSGGSVSLVSGDGV	13	2.20
AF (2020)_MLG16	Africa	DT	PDSSSSSRGI	DR	IPIVGLSQIPA	SPEDGTASTATTVQM	FGG	AL	YQ	AL	SPYV	LFTFV	HARSGG	SVL	LVSGGGV	DTPDSSSSSRGIDRIPIVGLSQIPASPEDGTASTATTVQMFGGALYQALSPYVLFTFVHARSGGSVLLVSGGGV	10	1.70
AF (2020)_MLG17	Africa	DT	PDSSSSSRGI	DR	IPIVGLSQIPA	SPEDGTASTATTVQM	FGG	AL	YQ	AL	SPYV	LFTFV	HVRSGG	SVL	LVSGGGV	DTPDSSSSSRGIDRIPIVGLSQIPASPEDGTASTATTVQMFGGALYQALSPYVLFTFVHVRSGGSVLLVSGGGV	10	1.70
AF (2020)_MLG18	Africa	DT	PDSSSSSRGI	DR	TPIVGLSQVPA	SPEDGTASTATTVQM	FGG	AL	YQ	AL	SPYV	LFTFV	QARSGG	SVL	LVSGGGV	DTPDSSSSSRGIDRTPIVGLSQVPASPEDGTASTATTVQMFGGALYQALSPYVLFTFVQARSGGSVLLVSGGGV	9	1.50
AF (2020)_MLG19	Africa	DT	PDSSSSSKRI	DR	TPIVGLSQVPA	SPEDGTASTATTVQM	FGG	AL	YQ	AL	SPYV	LFTFV	QARSGG	SLL	LVSGGGV	DTPDSSSSSKRIDRTPIVGLSQVPASPEDGTASTATTVQMFGGALYQALSPYVLFTFVQARSGGSLLLVSGGGV	8	1.30
AF (2020)_MLG20	Africa	DT	PDSLSSSRGI	DR	TPIVGLSQIPA	SPEDGTASTATTVQM	FGG	AL	YQ	AL	SPYV	LFTFV	QARSGG	SVL	LVSGGGV	DTPDSLSSSRGIDRTPIVGLSQIPASPEDGTASTATTVQMFGGALYQALSPYVLFTFVQARSGGSVLLVSGGGV	7	1.20
AF (2020)_MLG21	Africa	DT	PDSSSSSRGI	DR	TPIVGLSQIPA	SPEDGTASTATTVHM	FGG	AL	YQ	AL	SPYV	LFTFV	HARSGG	SVL	LVSGGGV	DTPDSSSSSRGIDRTPIVGLSQIPASPEDGTASTATTVHMFGGALYQALSPYVLFTFVHARSGGSVLLVSGGGV	6	1.00
AF (2020)_MLG22	Africa	DT	PDSSSSSKRI	DR	TPIVGLSQIPA	SPEDGTASTATTVQM	FGG	AF	YQ	AL	SPYV	LFTFV	QARSGG	SVL	LVSGGGV	DTPDSSSSSKRIDRTPIVGLSQIPASPEDGTASTATTVQMFGGAFYQALSPYVLFTFVQARSGGSVLLVSGGGV	5	0.80
AF (2020)_MLG23	Africa	DT	PDSSSSSRGI	DR	TPIVGLSQIPA	SPEDGTASTATTVQM	FGG	AF	YQ	AL	SPYV	LFTFV	QARSGG	SVL	LVSGGGV	DTPDSSSSSRGIDRTPIVGLSQIPASPEDGTASTATTVQMFGGAFYQALSPYVLFTFVQARSGGSVLLVSGGGV	5	0.80
AF (2020)_MLG24	Africa	DT	PDSSSSSRGI	DR	TPIVGLSQIPA	SPEDGTASTATTVQM	FGG	AL	HQ	AL	SPYV	LFTFV	QASSGG	SVL	LVSGGGV	DTPDSSSSSRGIDRTPIVGLSQIPASPEDGTASTATTVQMFGGALHQALSPYVLFTFVQASSGGSVLLVSGGGV	4	0.70
AF (2020)_MLG25	Africa	DT	PDSSSSSKRI	DR	TPIVGLSQIPA	SPEDGTASTATSVQM	FGG	AL	YQ	AL	SPYV	LFTFV	QARSGG	SVL	LVSGGGV	DTPDSSSSSKRIDRTPIVGLSQIPASPEDGTASTATSVQMFGGALYQALSPYVLFTFVQARSGGSVLLVSGGGV	4	0.70
AF (2020)_MLG26	Africa	DT	PDSSSSSRGI	DR	TPIVGLSQIPA	SPEDGTASTATTVQM	FGG	AL	HQ	AL	SPYV	LFTFV	QARSGG	SVL	LVSGGGV	DTPDSSSSSRGIDRTPIVGLSQIPASPEDGTASTATTVQMFGGALHQALSPYVLFTFVQARSGGSVLLVSGGGV	3	0.50
AF (2020)_MLG27	Africa	DT	PDSSSSSKRI	DR	TPIVGLSQIPA	SPEDGIASTATTVQM	FGG	AL	YQ	AL	SPYV	LFTFV	QARSGG	SVL	LVSGGGV	DTPDSSSSSKRIDRTPIVGLSQIPASPEDGIASTATTVQMFGGALYQALSPYVLFTFVQARSGGSVLLVSGGGV	3	0.50
AF (2020)_MLG28	Africa	DT	PDSSSSSRGI	DR	TPIVGLSQVSA	SPEDGTASTATTVQM	FGG	AF	YQ	AP	SPYV	LFTFV	QARSGV	SVL	LVSGDGV	DTPDSSSSSRGIDRTPIVGLSQVSASPEDGTASTATTVQMFGGAFYQAPSPYVLFTFVQARSGVSVLLVSGDGV	3	0.50
AF (2020)_MLG29	Africa	DT	PDSSSSSRGI	DR	TPIVGLSQIPA	SPEDGTASTATTVQM	FGG	AL	YQ	AL	SPYV	LFTFV	QARSGD	SVL	LVSGGGV	DTPDSSSSSRGIDRTPIVGLSQIPASPEDGTASTATTVQMFGGALYQALSPYVLFTFVQARSGDSVLLVSGGGV	3	0.50
AF (2020)_MLG30	Africa	DT	PDSSLSSRGI	DR	TPIVGLSQIPA	SPEDGTASTATTVQM	FGG	AL	YQ	AL	SPYV	LFTFV	HARSGG	SVL	LVSGGGV	DTPDSSLSSRGIDRTPIVGLSQIPASPEDGTASTATTVQMFGGALYQALSPYVLFTFVHARSGGSVLLVSGGGV	2	0.30
AF (2020)_MLG31	Africa	DT	PDSSSSSKRI	DR	TPIVGLSQIPA	SPEDGIASTATTVQM	FSG	AF	YQ	AL	SPYV	LFTFV	QARSGG	SVL	LVSGGGV	DTPDSSSSSKRIDRTPIVGLSQIPASPEDGIASTATTVQMFSGAFYQALSPYVLFTFVQARSGGSVLLVSGGGV	2	0.30
AF (2020)_MLG32	Africa	DT	PDSSSSSKRI	DR	TPIVGLSQIPA	SPEDGTASTATTVQM	FGG	AL	YQ	AL	SPYV	LFTFV	QARSGG	SLL	LVSGGGV	DTPDSSSSSKRIDRTPIVGLSQIPASPEDGTASTATTVQMFGGALYQALSPYVLFTFVQARSGGSLLLVSGGGV	2	0.30
AF (2020)_MLG33	Africa	DT	LDSSSSSRGI	DR	TPIVGLSQIPA	SPEDGTASKATTVQM	FGG	AF	YQ	VP	SPYV	LFTFV	QARSGG	SVL	LVSGDGV	DTLDSSSSSRGIDRTPIVGLSQIPASPEDGTASKATTVQMFGGAFYQVPSPYVLFTFVQARSGGSVLLVSGDGV	2	0.30
AF (2020)_MLG34	Africa	DT	PDSSSSSRGI	DR	TPIVGLSQIPA	SPEDGTASTATSVQM	FGG	AL	YQ	AL	SPYV	LFTFV	QARSGG	SVL	LVSGGGV	DTPDSSSSSRGIDRTPIVGLSQIPASPEDGTASTATSVQMFGGALYQALSPYVLFTFVQARSGGSVLLVSGGGV	2	0.30
AF (2020)_MLG35	Africa	DT	PDSSSSSRGI	DR	IPIVGLSQIPA	SPEDGTASTATTVQM	FGG	AF	YQ	AL	SPYV	LFTFV	HARSGG	SVL	LVSGGGV	DTPDSSSSSRGIDRIPIVGLSQIPASPEDGTASTATTVQMFGGAFYQALSPYVLFTFVHARSGGSVLLVSGGGV	2	0.30
AF (2020)_MLG36	Africa	DT	PDSSSSSKRI	DR	TPIVGLSQVPA	SPEDGTASTATTVQM	FGG	AL	YQ	AL	SPYV	LFTFV	QASSGG	SVL	LVSGGGV	DTPDSSSSSKRIDRTPIVGLSQVPASPEDGTASTATTVQMFGGALYQALSPYVLFTFVQASSGGSVLLVSGGGV	2	0.30
AF (2020)_MLG37	Africa	DT	PDSSSSSRGI	DR	TPIVGLSQIPA	SPEDGTASTATTVQM	FGG	AL	YQ	AL	SPYV	LFTFV	HARSGG	SVL	LVRGGGV	DTPDSSSSSRGIDRTPIVGLSQIPASPEDGTASTATTVQMFGGALYQALSPYVLFTFVHARSGGSVLLVRGGGV	1	0.20
AF (2020)_MLG38	Africa	DT	PDSSSSSRGI	DR	TPIVGLSQIPA	SPEDGIASTATTVQM	FGG	AF	YQ	AP	SPYV	LFTFV	QARSGV	SVL	LVSGDGV	DTPDSSSSSRGIDRTPIVGLSQIPASPEDGIASTATTVQMFGGAFYQAPSPYVLFTFVQARSGVSVLLVSGDGV	1	0.20
AF (2020)_MLG39	Africa	DT	PDSSSSNRGI	DR	TPIVGLSQIPA	SPEYGTASTATTVQM	FGG	AL	YQ	AP	SPYV	LFTFV	QARSGG	SVS	LVSGDGV	DTPDSSSSNRGIDRTPIVGLSQIPASPEYGTASTATTVQMFGGALYQAPSPYVLFTFVQARSGGSVSLVSGDGV	1	0.20
AF (2020)_MLG40	Africa	DT	PDSLSSSRGI	DR	TPIVGLSQIPA	SPEDGTASTATTVQM	FGG	AL	YQ	AL	SPYV	LFTFV	QARFGG	SVL	LVSGGGV	DTPDSLSSSRGIDRTPIVGLSQIPASPEDGTASTATTVQMFGGALYQALSPYVLFTFVQARFGGSVLLVSGGGV	1	0.20
AF (2020)_MLG41	Africa	DT	PDSSSSSRGI	DR	TPIVGLSQIPA	SPEDGTASIATTVQM	FGG	AL	YQ	AL	SPYV	LFTFV	QARSGG	SVL	LVSGGGV	DTPDSSSSSRGIDRTPIVGLSQIPASPEDGTASIATTVQMFGGALYQALSPYVLFTFVQARSGGSVLLVSGGGV	1	0.20
AF (2020)_MLG42	Africa	DT	PDSSSSSRGI	DR	TPIVGLSQIPA	SPEDGTASTATTVQM	FGG	AL	YQ	AP	SPYV	LFTFV	QARSGG	SVL	LVSGDGV	DTPDSSSSSRGIDRTPIVGLSQIPASPEDGTASTATTVQMFGGALYQAPSPYVLFTFVQARSGGSVLLVSGDGV	1	0.20
AF (2020)_MLG43	Africa	DT	PDSSSSSRGI	DR	TPIVGLSQIPA	SPEDGTASTATTVQM	FGG	AL	YQ	AL	SPYV	LFTFV	HARSGG	SVL	LVSGGGF	DTPDSSSSSRGIDRTPIVGLSQIPASPEDGTASTATTVQMFGGALYQALSPYVLFTFVHARSGGSVLLVSGGGF	1	0.20
AF (2020)_MLG44	Africa	DT	PDSSSSNRGI	DR	TPIVGLSQIPA	SPEDGTASTATTVQM	FGG	AL	HQ	AL	SPYV	LFTFV	QASSGG	SVL	LVSGGGV	DTPDSSSSNRGIDRTPIVGLSQIPASPEDGTASTATTVQMFGGALHQALSPYVLFTFVQASSGGSVLLVSGGGV	1	0.20
AF (2020)_MLG45	Africa	DT	PDSSLSSRGI	DR	TPIVGLSQIPA	SPEDGTASTATTVQM	FGG	AL	YQ	AL	SPYV	LFTFV	QARSGG	SVL	LVSGGGV	DTPDSSLSSRGIDRTPIVGLSQIPASPEDGTASTATTVQMFGGALYQALSPYVLFTFVQARSGGSVLLVSGGGV	1	0.20
AF (2020)_MLG46	Africa	DT	PDSSSSSRGI	DR	TPIVGLSQIPA	SPEDGTASTATTVQL	FGG	AF	YQ	AL	SSYV	LFTFV	QARSGG	SVL	LVSGGGV	DTPDSSSSSRGIDRTPIVGLSQIPASPEDGTASTATTVQLFGGAFYQALSSYVLFTFVQARSGGSVLLVSGGGV	1	0.20
AF (2020)_MLG47	Africa	DT	PDSLSSSRGI	DR	TPIVGLSQIPA	SPEDGTASTATSVQM	FGG	AL	YQ	AL	SPYV	LFTFV	QARSGG	SVL	LVSGGGV	DTPDSLSSSRGIDRTPIVGLSQIPASPEDGTASTATSVQMFGGALYQALSPYVLFTFVQARSGGSVLLVSGGGV	1	0.20
AF (2020)_MLG48	Africa	DT	PDSSSSSRGI	DR	TPIVGLSQVSA	SPEDGTASTATTVQM	FGG	AL	YQ	AP	SPYV	LFTFV	QARSGV	SVL	LVSGDGV	DTPDSSSSSRGIDRTPIVGLSQVSASPEDGTASTATTVQMFGGALYQAPSPYVLFTFVQARSGVSVLLVSGDGV	1	0.20
AF (2020)_MLG49	Africa	DT	PDSSSLSRGI	DR	TPIVGLSQIPA	SPEDGTASTATTVQM	YGG	AL	YQ	AP	SPYV	LFTFV	QARSVG	SVS	LVSGDGV	DTPDSSSLSRGIDRTPIVGLSQIPASPEDGTASTATTVQMYGGALYQAPSPYVLFTFVQARSVGSVSLVSGDGV	1	0.20
AF (2020)_MLG50	Africa	DT	PDSSSSSRGI	DR	TPIVGLSQIPA	SPEDGTASTATTVQL	FGG	AL	YQ	AL	SPYV	LFTFV	QARSGG	SVL	LVSGGGV	DTPDSSSSSRGIDRTPIVGLSQIPASPEDGTASTATTVQLFGGALYQALSPYVLFTFVQARSGGSVLLVSGGGV	1	0.20
AF (2020)_MLG51	Africa	DT	PDSSSSSRGI	DR	IPIVGLSQIPA	SPEDGTASTATTVQM	FGG	AL	YQ	AL	SPYV	LFTFV	HARFGG	SVL	LVSGGGV	DTPDSSSSSRGIDRIPIVGLSQIPASPEDGTASTATTVQMFGGALYQALSPYVLFTFVHARFGGSVLLVSGGGV	1	0.20
AF (2020)_MLG52	Africa	DT	PDSSSSSKRI	DR	NPIVGLSQIPA	SPEDGTASTATTVQM	FGG	AL	YQ	AL	SPYV	LFTFV	QARSGG	SVL	LVSGGGV	DTPDSSSSSKRIDRNPIVGLSQIPASPEDGTASTATTVQMFGGALYQALSPYVLFTFVQARSGGSVLLVSGGGV	1	0.20
AF (2020)_MLG53	Africa	DT	PDSSSSSRGI	DR	TPIVDLSQIPT	SPEDGTASTATTVQM	FGG	AL	YQ	AP	SPYV	LFTFV	QARSGG	SVL	LVSGDGV	DTPDSSSSSRGIDRTPIVDLSQIPTSPEDGTASTATTVQMFGGALYQAPSPYVLFTFVQARSGGSVLLVSGDGV	1	0.20
AF (2020)_MLG54	Africa	DT	PDSSSSSKRI	DR	TPIVGLSQIPA	SPEDGTASTATSVQM	FGG	AL	YQ	AP	SPYV	LFTFV	QARSGG	SVL	LVSGGGV	DTPDSSSSSKRIDRTPIVGLSQIPASPEDGTASTATSVQMFGGALYQAPSPYVLFTFVQARSGGSVLLVSGGGV	1	0.20
AF (2020)_MLG55	Africa	DT	PDSSSSNRGI	DR	TPIVGLSQIPA	SPEDGTASTATTVQM	FSG	AL	YQ	AP	SPYV	LFTFV	QARSGG	SVS	LVSGDGV	DTPDSSSSNRGIDRTPIVGLSQIPASPEDGTASTATTVQMFSGALYQAPSPYVLFTFVQARSGGSVSLVSGDGV	1	0.20
AF (2020)_MLG56	Africa	DT	PDSSSSSRGI	DR	TPIVGLSQIPA	SPEDGTASTATSVHM	FGG	AL	YQ	AL	SPYV	LFTFV	HARSGG	SVL	LVSGGGV	DTPDSSSSSRGIDRTPIVGLSQIPASPEDGTASTATSVHMFGGALYQALSPYVLFTFVHARSGGSVLLVSGGGV	1	0.20
AF (2020)_MLG57	Africa	DT	PDSLSSSRGI	DR	TPIVGFSQIPA	SPEDGTASTATTVQM	FGG	AL	YQ	AL	SPYV	LFTFL	QARSGG	SVL	LVSGGGV	DTPDSLSSSRGIDRTPIVGFSQIPASPEDGTASTATTVQMFGGALYQALSPYVLFTFLQARSGGSVLLVSGGGV	1	0.20
AF (2020)_MLG58	Africa	GT	PDSSSSSRGI	ER	TPIVGLSQIPA	SPEDGTASTATTVQM	FGG	AL	YQ	AL	SPYV	LFTFV	QARSGG	SVL	LVSGGGV	GTPDSSSSSRGIERTPIVGLSQIPASPEDGTASTATTVQMFGGALYQALSPYVLFTFVQARSGGSVLLVSGGGV	1	0.20
AF (2020)_MLG59	Africa	DT	PDSSSSSRGI	DR	TPIVGLSQIPA	SPEDGTASTATTVQM	FGG	AL	YQ	AL	SPYV	LFTFI	QARSGG	SVL	LVSGGGV	DTPDSSSSSRGIDRTPIVGLSQIPASPEDGTASTATTVQMFGGALYQALSPYVLFTFIQARSGGSVLLVSGGGV	1	0.20
AF (2020)_MLG60	Africa	DT	PDSSSSSKRI	DR	TPIVGLSQIPA	SPEEGTASTATTVHM	FGG	AL	YQ	AL	SPYV	LFTFV	HARSGG	SVL	LVSGGGV	DTPDSSSSSKRIDRTPIVGLSQIPASPEEGTASTATTVHMFGGALYQALSPYVLFTFVHARSGGSVLLVSGGGV	1	0.20
AF (2020)_MLG61	Africa	DT	PDSSSSSKRI	DR	TPIVGLSQIPA	SPEEGTASTVTSVQM	FGG	AL	YQ	AL	SPYV	LFTFV	QARSGG	SVL	LVSGGGV	DTPDSSSSSKRIDRTPIVGLSQIPASPEEGTASTVTSVQMFGGALYQALSPYVLFTFVQARSGGSVLLVSGGGV	1	0.20
AF (2020)_MLG62	Africa	DT	PDSSSSSRGV	DR	TPIVGLSQIPA	SPEDGTASTATTVQM	FGG	AL	YQ	AL	SPYV	LFTFV	QARSGG	SVL	LVSGGGV	DTPDSSSSSRGVDRTPIVGLSQIPASPEDGTASTATTVQMFGGALYQALSPYVLFTFVQARSGGSVLLVSGGGV	1	0.20
AF (2020)_MLG63	Africa	DT	PDSSSSSRGI	DR	TPIVGLSQIPA	SPEDGTASTATTVQM	FGG	AL	YQ	AL	SPYV	LFTFV	QASSGG	SVL	LVSGGGV	DTPDSSSSSRGIDRTPIVGLSQIPASPEDGTASTATTVQMFGGALYQALSPYVLFTFVQASSGGSVLLVSGGGV	1	0.20

AF (2020)_MLG64	Africa	DT	PDSSSSSRGI	DR	TPIVGLSQVPA	SPEDGTASTATTVQM	FGG	AL	YQ	AL	SPYV	LFTFV	QARSGG	SLL	LVSGGGV	DTPDSSSSSRGIDRTPIVGLSQVPASPEDGTASTATTVQMFGGALYQALSPYVLFTFVQARSGGSLLLVSGGGV	1	0.20
AF (2020)_MLG65	Africa	GT	PDSSSSSKRI	DR	TPIVGLSQVPA	SPEDGTASTATTVQM	FGG	AL	YQ	AL	SPYV	LFTFV	QARSGG	SVL	LVSGGGV	GTPDSSSSSKRIDRTPIVGLSQVPASPEDGTASTATTVQMFGGALYQALSPYVLFTFVQARSGGSVLLVSGGGV	1	0.20
AF (2020)_MLG66	Africa	DT	PDSSSSSKRI	DR	TPIVGLSQVPA	SPEDGTASTATAVQM	FGG	AL	YQ	AL	SPYV	LFTFV	QARSGG	SVL	LVSGGGV	DTPDSSSSSKRIDRTPIVGLSQVPASPEDGTASTATAVQMFGGALYQALSPYVLFTFVQARSGGSVLLVSGGGV	1	0.20
AF (2020)_MLG67	Africa	DT	PDSSSSSRGI	DR	TPIVGLSQVPA	SPEDGTASTATTVQM	FGG	AL	HQ	AL	SPYV	LFTFV	QARSGG	SVL	LVSGGGV	DTPDSSSSSRGIDRTPIVGLSQVPASPEDGTASTATTVQMFGGALHQALSPYVLFTFVQARSGGSVLLVSGGGV	1	0.20
AF (2020)_MLG68	Africa	DT	PDSSSSSKRI	DR	TPIVGLSQVPA	SLEDGTASTATTVQM	FGG	AL	YQ	AL	SPYV	LFTFV	QARSGG	SVL	LVSGGGV	DTPDSSSSSKRIDRTPIVGLSQVPASLEDGTASTATTVQMFGGALYQALSPYVLFTFVQARSGGSVLLVSGGGV	1	0.20
AF (2020)_MLG69	Africa	DT	PDSSSSSRGI	DR	IPIVGLSQVPA	SPEDGTASTATTVQM	FGG	AL	YQ	AL	SPYV	LFTFV	HARSGG	SVL	LVSGGGV	DTPDSSSSSRGIDRIPIVGLSQVPASPEDGTASTATTVQMFGGALYQALSPYVLFTFVHARSGGSVLLVSGGGV	1	0.20
AF (2020)_MLG70	Africa	DT	PDSSSSSKRI	DR	IPIVGLSQVPA	SPEDGIASTATTVQM	FSG	AL	YQ	AL	SPYV	LFTFV	QARSGG	SVL	LVSGGGV	DTPDSSSSSKRIDRIPIVGLSQVPASPEDGIASTATTVQMFSGALYQALSPYVLFTFVQARSGGSVLLVSGGGV	1	0.20
AF (2020)_MLG71	Africa	DT	PDSSSSSKRI	DR	TPIVGLSQVPA	SPEDGTARTATTVQM	FGG	AL	YQ	AL	SPYV	LFTFV	QARSGG	SVL	LVSGGGV	DTPDSSSSSKRIDRTPIVGLSQVPASPEDGTARTATTVQMFGGALYQALSPYVLFTFVQARSGGSVLLVSGGGV	1	0.20
AF (2020)_MLG72	Africa	DT	PDSSSSSKRI	DR	TPIVGLSQVPA	SPEDGTASTATTVQM	FGG	AL	YQ	AL	SPYV	LFTFV	QARSGG	SVL	FVSGGGV	DTPDSSSSSKRIDRTPIVGLSQVPASPEDGTASTATTVQMFGGALYQALSPYVLFTFVQARSGGSVLFVSGGGV	1	0.20
AF (2020)_MLG73	Africa	DT	PDSSSSSKRI	DR	TPIVGLSQVPA	SPEDGTASTATTVQM	FGG	AL	HQ	AL	SPYV	LFTFV	QARSGG	SVL	LVSGDGV	DTPDSSSSSKRIDRTPIVGLSQVPASPEDGTASTATTVQMFGGALHQALSPYVLFTFVQARSGGSVLLVSGDGV	1	0.20
AF (2020)_MLG74	Africa	GT	PDSSSSSRGI	DR	TPIVGLSQVPA	SPEDGTASTATTVQM	FGG	AL	YQ	AL	SPYV	LFTFV	QARSGG	SVL	LVSGGGV	GTPDSSSSSRGIDRTPIVGLSQVPASPEDGTASTATTVQMFGGALYQALSPYVLFTFVQARSGGSVLLVSGGGV	1	0.20
AF (2021)_MLG75	Africa	DT	PDSSSSSRGI	DR	IPIVGLSQIDA	PEDGTASTATTVQM	FGG	AL	YQ	AL	SPYV	LFTFV	HARSGG	SVL	LVSGGGV	DTPDSSSSSRGIDRIPIVGLSQIDAPEDGTASTATTVQMFGGALYQALSPYVLFTFVHARSGGSVLLVSGGGV	412	38.72
AF (2021)_MLG76	Africa	DT	PDSSSSSKRI	DR	TPIVGLSQIDA	PEDGTASTATTVQM	FGG	AL	YQ	AL	SPYV	LFTFV	QARSGG	SVL	LVSGGGV	DTPDSSSSSKRIDRTPIVGLSQIDAPEDGTASTATTVQMFGGALYQALSPYVLFTFVQARSGGSVLLVSGGGV	171	16.07
AF (2021)_MLG77	Africa	DT	PDSSSSSRGI	DR	TPIVGLSQIDA	PEDGTASTATTVQM	FGG	AL	YQ	AL	SPYV	LFTFV	HARSGG	SVL	LVNGGGV	DTPDSSSSSRGIDRTPIVGLSQIDAPEDGTASTATTVQMFGGALYQALSPYVLFTFVHARSGGSVLLVNGGGV	121	11.37
AF (2021)_MLG78	Africa	DT	PDSSSSSRGI	DR	TPIVGLSQIDA	PEDGTASTATTVQM	FGG	AL	YQ	AL	SPYV	LFTFV	QARSGG	SVL	LVSGGGV	DTPDSSSSSRGIDRTPIVGLSQIDAPEDGTASTATTVQMFGGALYQALSPYVLFTFVQARSGGSVLLVSGGGV	59	5.55
AF (2021)_MLG79	Africa	DT	PDSSSSSRGI	DR	TPIVGLSQIDA	PEDGTASTATTVQM	FGG	AL	YQ	AF	SPYV	LFTFV	QARSGG	SVL	LVSGGGV	DTPDSSSSSRGIDRTPIVGLSQIDAPEDGTASTATTVQMFGGALYQAFSPYVLFTFVQARSGGSVLLVSGGGV	52	4.89
AF (2021)_MLG80	Africa	DT	PDSSSSSRGI	DR	TPIVGLSQIDA	PEDGTASTATTVQM	FGG	AL	YQ	AL	SPYV	LFTFV	HARSGG	SVL	LVSGGGV	DTPDSSSSSRGIDRTPIVGLSQIDAPEDGTASTATTVQMFGGALYQALSPYVLFTFVHARSGGSVLLVSGGGV	36	3.38
AF (2021)_MLG81	Africa	DT	PDSSSSSRGI	DR	TPIVGLSQIDA	PEDGTASTATTVQM	FGG	AL	YQ	AP	SPYV	LFTFV	QARSGG	SVL	LVSGDGV	DTPDSSSSSRGIDRTPIVGLSQIDAPEDGTASTATTVQMFGGALYQAPSPYVLFTFVQARSGGSVLLVSGDGV	28	2.63
AF (2021)_MLG82	Africa	DT	PDSLSSSRGI	DR	TPIVGLSQIDA	PEDGTASTATTVQM	FGG	AL	YQ	AL	SPYV	LFTFV	HARSGG	SVL	LVSGGGV	DTPDSLSSSRGIDRTPIVGLSQIDAPEDGTASTATTVQMFGGALYQALSPYVLFTFVHARSGGSVLLVSGGGV	27	2.54
AF (2021)_MLG83	Africa	DT	PDSLSSSRGI	DR	TPIVGLSQIDA	PEDGTASTATTVQM	FGG	AL	YQ	AL	SPYV	LFTFV	QARSGG	SVL	LVSGGGV	DTPDSLSSSRGIDRTPIVGLSQIDAPEDGTASTATTVQMFGGALYQALSPYVLFTFVQARSGGSVLLVSGGGV	25	2.35
AF (2021)_MLG84	Africa	DT	PDSSSSNRGI	DR	TPIVGLSQIDA	PEDGTASTATTVQM	FGG	AL	YQ	AP	SPYV	LFTFV	QARSGG	SVS	LVSGDGV	DTPDSSSSNRGIDRTPIVGLSQIDAPEDGTASTATTVQMFGGALYQAPSPYVLFTFVQARSGGSVSLVSGDGV	21	1.97
AF (2021)_MLG85	Africa	DT	SDSSSTSKRI	DR	TPIVGLSQIDA	PEDGTASTATTVQM	FGG	AL	YQ	AL	SPYV	LFTFV	QARSGG	SVL	LVSGGGV	DTSDSSSTSKRIDRTPIVGLSQIDAPEDGTASTATTVQMFGGALYQALSPYVLFTFVQARSGGSVLLVSGGGV	13	1.22
AF (2021)_MLG86	Africa	DT	PDSSSSSKRI	DR	TPIVGLSQIDA	PEDGIASTATTVQM	FSG	AL	YQ	AL	SPYV	LFTFV	QARSGG	SVL	LVSGGGV	DTPDSSSSSKRIDRTPIVGLSQIDAPEDGIASTATTVQMFSGALYQALSPYVLFTFVQARSGGSVLLVSGGGV	10	0.94
AF (2021)_MLG87	Africa	DT	SDSSSSSRGI	DR	IPIVGLSQIDA	PEDGTASTATTVQM	FGG	AL	YQ	AL	SPYV	LFTFV	HARSGG	SVL	LVSGGGV	DTSDSSSSSRGIDRIPIVGLSQIDAPEDGTASTATTVQMFGGALYQALSPYVLFTFVHARSGGSVLLVSGGGV	8	0.75
AF (2021)_MLG88	Africa	DT	PDSSSSSKRI	DR	TPIVGLSQIDA	PEDGTASTATTVQM	FGG	AF	YQ	AL	SPYV	LFTFV	QARSGG	SVL	LVSGGGV	DTPDSSSSSKRIDRTPIVGLSQIDAPEDGTASTATTVQMFGGAFYQALSPYVLFTFVQARSGGSVLLVSGGGV	7	0.66
AF (2021)_MLG89	Africa	DT	SDSSSSSRGI	DR	TPIVGLSQIDA	PEDGTASTATTVQM	FGG	AL	YQ	AL	SPYV	LFTFV	QARSGG	SVL	LVSGGGV	DTSDSSSSSRGIDRTPIVGLSQIDAPEDGTASTATTVQMFGGALYQALSPYVLFTFVQARSGGSVLLVSGGGV	5	0.47
AF (2021)_MLG90	Africa	DT	PDSSSSSRGI	DR	TPIVGLSQIDA	PEDSTASTATTVQM	FGG	AL	YQ	AL	SPYV	LFTFV	HARSGG	SVL	LVNGGGV	DTPDSSSSSRGIDRTPIVGLSQIDAPEDSTASTATTVQMFGGALYQALSPYVLFTFVHARSGGSVLLVNGGGV	4	0.38
AF (2021)_MLG91	Africa	DT	PDSSSSSKRI	DR	TPIVGLSQIDA	PEDGTASTATTVQM	FGG	AL	YQ	AL	SPYV	LFTFV	HARSGG	SVL	LVSGGGV	DTPDSSSSSKRIDRTPIVGLSQIDAPEDGTASTATTVQMFGGALYQALSPYVLFTFVHARSGGSVLLVSGGGV	4	0.38
AF (2021)_MLG92	Africa	DT	PDSSSSSRGI	DR	IPIVGLSQIDA	PEDGTASTATTVQM	FGG	AF	YQ	AL	SPYV	LFTFV	HARSGG	SVL	LVSGGGV	DTPDSSSSSRGIDRIPIVGLSQIDAPEDGTASTATTVQMFGGAFYQALSPYVLFTFVHARSGGSVLLVSGGGV	3	0.28
AF (2021)_MLG93	Africa	DT	PDSSSSSRGI	DR	TPIVGLSQIDA	PEDGTASTATTVQM	FGG	AF	YQ	AL	SPYV	LFTFV	QARSGG	SVL	LVSGGGV	DTPDSSSSSRGIDRTPIVGLSQIDAPEDGTASTATTVQMFGGAFYQALSPYVLFTFVQARSGGSVLLVSGGGV	3	0.28
AF (2021)_MLG94	Africa	DT	PDSSSSSRGI	DR	IPIVGLSQIDA	PEDGTASTATTVQM	FGG	AL	YQ	AH	SPYV	LFTFV	HARSGG	SVL	LVSGGGV	DTPDSSSSSRGIDRIPIVGLSQIDAPEDGTASTATTVQMFGGALYQAHSPYVLFTFVHARSGGSVLLVSGGGV	3	0.28
AF (2021)_MLG95	Africa	DT	LDSSSSSRGI	DR	TPIVGLSQIDA	PEDGTASTATTVQM	FGG	AL	YQ	AL	SPYV	LFTFV	QARSGG	SVL	LVSGGGV	DTLDSSSSSRGIDRTPIVGLSQIDAPEDGTASTATTVQMFGGALYQALSPYVLFTFVQARSGGSVLLVSGGGV	3	0.28
AF (2021)_MLG96	Africa	DT	SDSSSSSRGI	DR	TPIVGLSQIDA	PEDGTASTATTVQM	FGG	AL	YQ	AL	SPYV	LFTFV	QARSGG	SVL	LVSGDGV	DTSDSSSSSRGIDRTPIVGLSQIDAPEDGTASTATTVQMFGGALYQALSPYVLFTFVQARSGGSVLLVSGDGV	3	0.28
AF (2021)_MLG97	Africa	DT	PDSLSSSRGI	DR	TPIVGLSQIDA	PEDGTASTATTVQM	FGG	AL	YQ	AL	SPYV	LFTFV	HARSGG	SVL	LVNGGGV	DTPDSLSSSRGIDRTPIVGLSQIDAPEDGTASTATTVQMFGGALYQALSPYVLFTFVHARSGGSVLLVNGGGV	3	0.28
AF (2021)_MLG98	Africa	DT	PDSLSSSKRI	DR	TPIVGLSQIDA	PEDGTASTATTVQM	FGG	AL	YQ	AL	SPYV	LFTFV	QARSGG	SVL	LVSGGGV	DTPDSLSSSKRIDRTPIVGLSQIDAPEDGTASTATTVQMFGGALYQALSPYVLFTFVQARSGGSVLLVSGGGV	3	0.28
AF (2021)_MLG99	Africa	DT	PDSSSSSRGI	DR	TPIVGLSQIDA	PEDGTASTATTVQM	FGG	AL	YQ	AL	SPYV	LFTFV	QSRSGG	SVL	LVSGGGV	DTPDSSSSSRGIDRTPIVGLSQIDAPEDGTASTATTVQMFGGALYQALSPYVLFTFVQSRSGGSVLLVSGGGV	2	0.19
AF (2021)_MLG100	Africa	DT	PDSSSSNRGI	DR	TPIVGLSQIDA	PEDGTASTATTVQM	FGG	AF	YQ	AP	SPYV	LFTFV	QARSGG	SVS	LVSGDGV	DTPDSSSSNRGIDRTPIVGLSQIDAPEDGTASTATTVQMFGGAFYQAPSPYVLFTFVQARSGGSVSLVSGDGV	2	0.19
AF (2021)_MLG101	Africa	DT	PDSSSSSRGI	DR	IPIVGLSQIDA	PEDGTASTATSVQM	FGG	AL	YQ	AL	SPYV	LFTFV	HARSGG	LVL	LVSGGGV	DTPDSSSSSRGIDRIPIVGLSQIDAPEDGTASTATSVQMFGGALYQALSPYVLFTFVHARSGGLVLLVSGGGV	2	0.19
AF (2021)_MLG102	Africa	DT	PDSSSSSKRI	DR	TPIVGLSQIDA	LEDGTASTATTVQM	FGG	AL	YQ	AL	SPYV	LFTFV	QARSGG	SVL	LVSGGGV	DTPDSSSSSKRIDRTPIVGLSQIDALEDGTASTATTVQMFGGALYQALSPYVLFTFVQARSGGSVLLVSGGGV	2	0.19
AF (2021)_MLG103	Africa	DT	PDSSSSSKRI	DR	TRIVGLSQIDA	PEDGTASTATTVQM	FGG	AL	YQ	AL	SPYV	LFTFV	QARSGG	SVL	LVSGGGV	DTPDSSSSSKRIDRTRIVGLSQIDAPEDGTASTATTVQMFGGALYQALSPYVLFTFVQARSGGSVLLVSGGGV	2	0.19
AF (2021)_MLG104	Africa	DT	PDSSSSNKGI	DR	TPIVGLSQIDA	PEDGTASTATTVQM	FGG	AL	YQ	AP	SPYV	LFTFV	QARSGG	SVS	LVSGDGV	DTPDSSSSNKGIDRTPIVGLSQIDAPEDGTASTATTVQMFGGALYQAPSPYVLFTFVQARSGGSVSLVSGDGV	2	0.19
AF (2021)_MLG105	Africa	DT	LDSSSSSRGI	DR	IPIVGLSQIDA	PEDGTASTATTVQM	FGG	AL	YQ	AL	SPYV	LFTFV	HARSGG	SVL	LVSGGGV	DTLDSSSSSRGIDRIPIVGLSQIDAPEDGTASTATTVQMFGGALYQALSPYVLFTFVHARSGGSVLLVSGGGV	1	0.09
AF (2021)_MLG106	Africa	GT	PDSSSSSRGI	DR	TPIVGLSQIDA	PEDGTASTATTVQM	FGG	AL	YQ	AL	SPYV	LFTFV	QARSGG	SVL	LVSGGGV	GTPDSSSSSRGIDRTPIVGLSQIDAPEDGTASTATTVQMFGGALYQALSPYVLFTFVQARSGGSVLLVSGGGV	1	0.09
AF (2021)_MLG107	Africa	DT	PDSSSSSRGI	DR	IPIVGLSQIDA	PEDGTASTATTVQM	FGG	AL	YQ	AF	SPYV	LFTFV	HARSGG	SVL	LVSGGGV	DTPDSSSSSRGIDRIPIVGLSQIDAPEDGTASTATTVQMFGGALYQAFSPYVLFTFVHARSGGSVLLVSGGGV	1	0.09
AF (2021)_MLG108	Africa	DT	PDSLSSSRGI	DR	TPIVGLSQIDA	PEDGTASTATTVQM	FGG	AL	YQ	AL	SPYV	LFTFV	HARSGG	SVL	LVSGGCV	DTPDSLSSSRGIDRTPIVGLSQIDAPEDGTASTATTVQMFGGALYQALSPYVLFTFVHARSGGSVLLVSGGCV	1	0.09
AF (2021)_MLG109	Africa	DT	PDSSSSSRGI	DR	IPIVGLSQIDA	PEDGTASIATTVQM	FGG	AL	YQ	AL	SPYV	LFTFV	HARSGG	SVL	LVSGGGV	DTPDSSSSSRGIDRIPIVGLSQIDAPEDGTASIATTVQMFGGALYQALSPYVLFTFVHARSGGSVLLVSGGGV	1	0.09
AF (2021)_MLG110	Africa	DT	PDSSSSSRGI	DR	TPIVGLSQIDA	LEDGTASTATTVQM	FGG	AL	YQ	AL	SPYV	LFTFV	QARSGG	SVL	LVSGGGV	DTPDSSSSSRGIDRTPIVGLSQIDALEDGTASTATTVQMFGGALYQALSPYVLFTFVQARSGGSVLLVSGGGV	1	0.09
AF (2021)_MLG111	Africa	DT	PDSSSSSRGI	DR	IPIVGLSQIDA	PEDGTASTATTVQM	FGG	AL	YQ	AL	SPYV	LFTFV	YARSGG	SVL	LVSGGGV	DTPDSSSSSRGIDRIPIVGLSQIDAPEDGTASTATTVQMFGGALYQALSPYVLFTFVYARSGGSVLLVSGGGV	1	0.09
AF (2021)_MLG112	Africa	DT	PDSSSSSRGI	DR	IPIVGLSQIDA	PEDGTASTATTVQM	FGG	AL	YQ	AL	SPYV	LFTFV	HARSVG	SVL	LVSGGGV	DTPDSSSSSRGIDRIPIVGLSQIDAPEDGTASTATTVQMFGGALYQALSPYVLFTFVHARSVGSVLLVSGGGV	1	0.09
AF (2021)_MLG113	Africa	DT	PDSSSSSRGI	DR	TPIVGLSQIDA	PEDGTASTATTVQM	FGG	AL	YQ	AF	SPYV	LFTFV	QARSGG	SVL	FVSGGGV	DTPDSSSSSRGIDRTPIVGLSQIDAPEDGTASTATTVQMFGGALYQAFSPYVLFTFVQARSGGSVLFVSGGGV	1	0.09
AF (2021)_MLG114	Africa	DT	PDSSSSSRGI	DR	TPIVGLSQIDA	PEDGTASTATTVQM	FGG	AL	YQ	AL	SSYV	LFTFV	HARSGG	SVL	LVSGGGV	DTPDSSSSSRGIDRTPIVGLSQIDAPEDGTASTATTVQMFGGALYQALSSYVLFTFVHARSGGSVLLVSGGGV	1	0.09
AF (2021)_MLG115	Africa	DT	PDSSSSNRGI	DR	TPIVGLSQIDA	PEDGTASTATTVQM	FGG	AL	YQ	AL	SPYV	LFTFV	QARSGG	SVL	LVSGGGV	DTPDSSSSNRGIDRTPIVGLSQIDAPEDGTASTATTVQMFGGALYQALSPYVLFTFVQARSGGSVLLVSGGGV	1	0.09
AF (2021)_MLG116	Africa	DT	PDSSSSSRGI	DR	TPIVGLSQIDA	PEDGTASTATTVQM	FGG	AL	YQ	AF	SPYV	LFTFV	QARSGG	SVL	LVSGGVV	DTPDSSSSSRGIDRTPIVGLSQIDAPEDGTASTATTVQMFGGALYQAFSPYVLFTFVQARSGGSVLLVSGGVV	1	0.09
AF (2021)_MLG117	Africa	DT	PDSSSSSRGI	DR	TPIVGLSQIDA	PEDGTASTATTVQM	FGG	AF	YQ	AL	SPYV	LFTFV	HARSGG	SVL	LVNGGGV	DTPDSSSSSRGIDRTPIVGLSQIDAPEDGTASTATTVQMFGGAFYQALSPYVLFTFVHARSGGSVLLVNGGGV	1	0.09
AF (2021)_MLG118	Africa	DT	PDSSSSSRGN	DR	TPIVGLSQIDA	PEDGTASTATTVQM	FGG	AL	YQ	AF	SPYV	LFTFV	QARSGG	SVL	LVSGGGV	DTPDSSSSSRGNDRTPIVGLSQIDAPEDGTASTATTVQMFGGALYQAFSPYVLFTFVQARSGGSVLLVSGGGV	1	0.09
AF (2021)_MLG119	Africa	DT	PDSSSSSRGI	DR	IPIVGLSQIDA	PEDGTASTATTVQM	FGG	AL	YQ	AL	SPYV	LFTFV	HARSGG	SVL	LVGGGGV	DTPDSSSSSRGIDRIPIVGLSQIDAPEDGTASTATTVQMFGGALYQALSPYVLFTFVHARSGGSVLLVGGGGV	1	0.09
AF (2021)_MLG120	Africa	DT	TDSSSSSRGI	DR	TPIVGLSQIDA	PEDGTASTATTVQM	FGG	AL	YQ	AF	SPYV	LFTFV	QARSGG	SVL	LVSGGGV	DTTDSSSSSRGIDRTPIVGLSQIDAPEDGTASTATTVQMFGGALYQAFSPYVLFTFVQARSGGSVLLVSGGGV	1	0.09
AF (2021)_MLG121	Africa	DT	PDSSSSSRGI	DR	TPIVGLSQIDA	PEDGTASTATTVQL	FGG	AL	YQ	AF	SPYV	LFTFV	QARSGG	SVL	LVSGGGV	DTPDSSSSSRGIDRTPIVGLSQIDAPEDGTASTATTVQLFGGALYQAFSPYVLFTFVQARSGGSVLLVSGGGV	1	0.09
AF (2021)_MLG122	Africa	DT	PDSSSLSRGI	DR	TPIVGLSQIDA	PEDGTASTATTVQM	FGG	AL	YQ	AF	SPYV	LFTFV	QARSGG	SVL	LVSGGGV	DTPDSSSLSRGIDRTPIVGLSQIDAPEDGTASTATTVQMFGGALYQAFSPYVLFTFVQARSGGSVLLVSGGGV	1	0.09
AF (2021)_MLG123	Africa	DT	SDSSSSSRGI	DR	TPIVGLSQIDA	PEDGTASTATTVQM	FGG	AL	YQ	AL	SPYV	LFTFV	HARSGG	SVL	LVSGGGV	DTSDSSSSSRGIDRTPIVGLSQIDAPEDGTASTATTVQMFGGALYQALSPYVLFTFVHARSGGSVLLVSGGGV	1	0.09
AF (2021)_MLG124	Africa	DT	SDSSSSSRGI	DR	TPIVGLSQIDA	PEDGTASTATTVQM	FGG	AL	YQ	AP	SPYV	LFTFV	QARSGG	SVL	LVSGDGV	DTSDSSSSSRGIDRTPIVGLSQIDAPEDGTASTATTVQMFGGALYQAPSPYVLFTFVQARSGGSVLLVSGDGV	1	0.09
AF (2021)_MLG125	Africa	DT	PDSSSSSRGI	DR	TPIVGLSQIDA	PEDGTASTATTVQM	FGG	AL	YQ	AP	SPYV	LFTFV	QARSGG	SVL	LVSGGGV	DTPDSSSSSRGIDRTPIVGLSQIDAPEDGTASTATTVQMFGGALYQAPSPYVLFTFVQARSGGSVLLVSGGGV	1	0.09
AF (2021)_MLG126	Africa	DT	PDSSSSSKRI	DR	TPIVGLSQIDA	PEDGTASTATTVQM	FGG	AL	YQ	AL	SPYV	LFTFV	QARSGG	SVL	LVNGGGV	DTPDSSSSSKRIDRTPIVGLSQIDAPEDGTASTATTVQMFGGALYQALSPYVLFTFVQARSGGSVLLVNGGGV	1	0.09
AF (2021)_MLG127	Africa	DT	PYSSSSSKRI	DR	TPIVGLSQIDA	PEDGIASTATTVQM	FSG	AL	YQ	AL	SPYV	LFTFV	QARSGG	SVL	LVSGGGV	DTPYSSSSSKRIDRTPIVGLSQIDAPEDGIASTATTVQMFSGALYQALSPYVLFTFVQARSGGSVLLVSGGGV	1	0.09

AF (2021)_MLG128	Africa	DT	PDSSSSSKRI	DR	TPIVGLSQIDA	PEDGIASTATTVQM	FSG	AL	YQ	AL	SPYV	FFTFV	QARSGG	SVL	LVSGGGV	DTPDSSSSSKRIDRTPIVGLSQIDAPEDGIASTATTVQMFSGALYQALSPYVFFTFVQARSGGSVLLVSGGGV	1	0.09
AF (2021)_MLG129	Africa	DT	PDSSSSSKRI	DR	TPIVGLSQIDA	PEDGTASTAITVQM	FGG	AL	YQ	AL	SPYV	LFTFV	QARSGG	SVL	LVSGGGV	DTPDSSSSSKRIDRTPIVGLSQIDAPEDGTASTAITVQMFGGALYQALSPYVLFTFVQARSGGSVLLVSGGGV	1	0.09
AF (2021)_MLG130	Africa	DT	PDSSSSSKRI	DR	TPIVGLSQIDA	PEDGTASTATTVQM	FGG	AL	YQ	AL	SPYV	LFTFV	HSRSGG	SVL	LVSGGGV	DTPDSSSSSKRIDRTPIVGLSQIDAPEDGTASTATTVQMFGGALYQALSPYVLFTFVHSRSGGSVLLVSGGGV	1	0.09
AF (2021)_MLG131	Africa	DT	PDSSSSSKRI	DR	TPIVGLSQIDA	PEDGTDSTATTVQM	FGG	AL	YQ	AL	SPYV	LFTFV	QARSGG	SVL	LVSGGGV	DTPDSSSSSKRIDRTPIVGLSQIDAPEDGTDSTATTVQMFGGALYQALSPYVLFTFVQARSGGSVLLVSGGGV	1	0.09
AF (2021)_MLG132	Africa	DT	PDSSSSNKRI	DR	TPIVGLSQIDA	PEDGTASTATTVQM	FGG	AL	YQ	AL	SPYV	LFTFV	QARSGG	SVS	LVSGGGV	DTPDSSSSNKRIDRTPIVGLSQIDAPEDGTASTATTVQMFGGALYQALSPYVLFTFVQARSGGSVSLVSGGGV	1	0.09
AS (2020)_MLG1	Asia	DT	PDSSSSSRGI	DR	TPIVGLSQIPA	SPEDGTASTATTVQM	FGG	AL	YQ	AL	SPYV	LFTFV	QARSGG	SVL	LVSGGGV	DTPDSSSSSRGIDRTPIVGLSQIPASPEDGTASTATTVQMFGGALYQALSPYVLFTFVQARSGGSVLLVSGGGV	185	11.70
AS (2020)_MLG2	Asia	DT	PDSSSSSRGI	DR	TPIVGLSQIPA	SPEDGTASTATTVQM	FGG	AL	YQ	AP	SPYV	LFTFV	QARSGG	SVL	LVSGDGV	DTPDSSSSSRGIDRTPIVGLSQIPASPEDGTASTATTVQMFGGALYQAPSPYVLFTFVQARSGGSVLLVSGDGV	180	11.40
AS (2020)_MLG3	Asia	DT	PDSSSSSRGI	DR	TPIVGLSQIPA	SPEDGTASTATTVQM	FGG	AL	YQ	AL	SPYV	LFTFV	HARSGG	SVL	LVSGGGV	DTPDSSSSSRGIDRTPIVGLSQIPASPEDGTASTATTVQMFGGALYQALSPYVLFTFVHARSGGSVLLVSGGGV	110	7.00
AS (2020)_MLG4	Asia	DT	PDSSSSSKRI	DR	TPIVGLSQIPA	SPEDGTDSTATTVQM	FGG	AL	YQ	AL	SPYV	LFTFV	QARSGG	SVL	LVSGGGV	DTPDSSSSSKRIDRTPIVGLSQIPASPEDGTDSTATTVQMFGGALYQALSPYVLFTFVQARSGGSVLLVSGGGV	99	6.30
AS (2020)_MLG5	Asia	DT	PDSSLSSRGI	DR	TPIVGLSQIPA	SPEDGTASTATTVQM	FGG	AL	YQ	AL	SPYV	LFTFV	HARSGG	SVL	LVSGGGV	DTPDSSLSSRGIDRTPIVGLSQIPASPEDGTASTATTVQMFGGALYQALSPYVLFTFVHARSGGSVLLVSGGGV	81	5.10
AS (2020)_MLG6	Asia	DT	PDSSSSSKRI	DR	TPIVGLSQIPA	SPEDGTASTATTVQM	FGG	AL	YQ	AL	SPYV	LFTFV	QARSGG	SVL	LVSGGGV	DTPDSSSSSKRIDRTPIVGLSQIPASPEDGTASTATTVQMFGGALYQALSPYVLFTFVQARSGGSVLLVSGGGV	77	4.90
AS (2020)_MLG7	Asia	DT	LDSSSSSRGI	DR	TPIVGLSQIPA	SPEDGTARKATTVQM	FGG	AF	YQ	VP	SPYV	LFTFV	QARSGG	SVL	LVSGDGV	DTLDSSSSSRGIDRTPIVGLSQIPASPEDGTARKATTVQMFGGAFYQVPSPYVLFTFVQARSGGSVLLVSGDGV	70	4.40
AS (2020)_MLG8	Asia	DT	LDSSSSSRGI	DR	TPIVGLSQIPA	SPEDGTASKATTVQM	FGG	AF	YQ	VP	SPYV	LFTFV	QARSGG	SVL	LVSGDGV	DTLDSSSSSRGIDRTPIVGLSQIPASPEDGTASKATTVQMFGGAFYQVPSPYVLFTFVQARSGGSVLLVSGDGV	67	4.20
AS (2020)_MLG9	Asia	DT	PDSSSSSRGI	DR	IPIVGLSQIPA	SPEDGTASTATTVQM	FGG	AL	YQ	AL	SPYV	LFTFV	HARSGG	SVL	LVSGGGV	DTPDSSSSSRGIDRIPIVGLSQIPASPEDGTASTATTVQMFGGALYQALSPYVLFTFVHARSGGSVLLVSGGGV	61	3.90
AS (2020)_MLG10	Asia	DT	PDSSSSSRGI	DR	TPIVGLSQIPA	SPEDGTASTATTVQM	FGG	AL	YQ	AP	SPYV	LFTFV	QARSGG	SVS	LVSGDGV	DTPDSSSSSRGIDRTPIVGLSQIPASPEDGTASTATTVQMFGGALYQAPSPYVLFTFVQARSGGSVSLVSGDGV	56	3.50
AS (2020)_MLG11	Asia	DT	PDSSSSSRGI	DR	TPIVGLSQIPA	SPEDGTASTATTVQM	FGG	AL	YQ	AP	SPYV	LFTFV	QARSGV	SVL	LVSGDGV	DTPDSSSSSRGIDRTPIVGLSQIPASPEDGTASTATTVQMFGGALYQAPSPYVLFTFVQARSGVSVLLVSGDGV	40	2.50
AS (2020)_MLG12	Asia	DT	PDSSSSNRGI	DR	TPIVGLSQIPA	SPEDGTASTATTVQM	FGG	AL	YQ	AP	SPYV	LFTFV	QARSGG	SVS	LVSGDGV	DTPDSSSSNRGIDRTPIVGLSQIPASPEDGTASTATTVQMFGGALYQAPSPYVLFTFVQARSGGSVSLVSGDGV	39	2.50
AS (2020)_MLG13	Asia	DT	PDSSSSSKRI	DR	TPIVGLSQIPA	SPEDGTASTATTVQM	FGS	AL	YQ	AL	SPYV	LFTFV	QARSGG	SVL	LVSGGGV	DTPDSSSSSKRIDRTPIVGLSQIPASPEDGTASTATTVQMFGSALYQALSPYVLFTFVQARSGGSVLLVSGGGV	39	2.50
AS (2020)_MLG14	Asia	DT	LDSSSSSRGI	DR	TPIVGLFQIPA	SPEDGTARKATTVQM	FGG	AF	YH	VP	SPYV	LFTFV	QARSGG	SVL	LVSGDGV	DTLDSSSSSRGIDRTPIVGLFQIPASPEDGTARKATTVQMFGGAFYHVPSPYVLFTFVQARSGGSVLLVSGDGV	28	1.80
AS (2020)_MLG15	Asia	DT	PDSSSSSRGI	DR	TPIVGLSQIPA	SPEDGTASTATTVQM	FGG	AF	YQ	AP	SPYV	LFTFV	QARSGV	SVL	LVSGDGV	DTPDSSSSSRGIDRTPIVGLSQIPASPEDGTASTATTVQMFGGAFYQAPSPYVLFTFVQARSGVSVLLVSGDGV	22	1.40
AS (2020)_MLG16	Asia	DT	PDSSSSSRGI	DR	TPIVGLSQIPA	SPEDGTASTATTVQM	FGG	AL	YQ	AP	SPYV	LFTFV	QARSGG	SVL	LVSGGGV	DTPDSSSSSRGIDRTPIVGLSQIPASPEDGTASTATTVQMFGGALYQAPSPYVLFTFVQARSGGSVLLVSGGGV	18	1.10
AS (2020)_MLG17	Asia	DT	PDSSSSSRGI	DC	TPIIGLSQIPA	SPEDGTASTATTVQI	FGG	AF	YQ	AP	SPYV	LFTFV	QARSGG	SVL	LVSGDGV	DTPDSSSSSRGIDCTPIIGLSQIPASPEDGTASTATTVQIFGGAFYQAPSPYVLFTFVQARSGGSVLLVSGDGV	17	1.10
AS (2020)_MLG18	Asia	DT	PDSSSSSRGI	DR	TPIVGLSQIPA	SPEDGTASTATTGQM	FGG	AL	YQ	AL	SPYV	LFTFV	QARSGG	SVL	LVSGGGV	DTPDSSSSSRGIDRTPIVGLSQIPASPEDGTASTATTGQMFGGALYQALSPYVLFTFVQARSGGSVLLVSGGGV	14	0.90
AS (2020)_MLG19	Asia	DT	PDSSLSSRGI	DR	TPIVGLSQIPA	SPEDGTASTATTVQM	FGG	AL	YQ	AL	SPYV	LFTFV	HARSGG	SVL	FVSGGGV	DTPDSSLSSRGIDRTPIVGLSQIPASPEDGTASTATTVQMFGGALYQALSPYVLFTFVHARSGGSVLFVSGGGV	13	0.80
AS (2020)_MLG20	Asia	DT	PDSSSSSRGI	DR	TPIVGLSQVPA	SPEDGTASTATTVQM	FGG	AL	YQ	AL	SPYV	LFTFV	HARSGG	SVL	LVSGGGV	DTPDSSSSSRGIDRTPIVGLSQVPASPEDGTASTATTVQMFGGALYQALSPYVLFTFVHARSGGSVLLVSGGGV	13	0.80
AS (2020)_MLG21	Asia	DT	PDSSLSSRGI	DR	TPIVGLSPIPA	SPEDGTASTATTVQM	FGG	AL	YQ	AL	SPYV	FFIFV	HARSGG	SVL	FVSGGGV	DTPDSSLSSRGIDRTPIVGLSPIPASPEDGTASTATTVQMFGGALYQALSPYVFFIFVHARSGGSVLFVSGGGV	12	0.80
AS (2020)_MLG22	Asia	DT	PDSSLSSRGI	DR	TPIVGLSQIPA	SPEDGTASTATTVQM	FGG	AL	YQ	AL	SPYV	LFTFV	QARSGG	SVL	LVSGGGV	DTPDSSLSSRGIDRTPIVGLSQIPASPEDGTASTATTVQMFGGALYQALSPYVLFTFVQARSGGSVLLVSGGGV	11	0.70
AS (2020)_MLG23	Asia	DT	PDSSLSSRGI	DR	TPIVGLSPIPA	SPEDGTASTATTVQM	FGG	AL	YQ	AL	SPYV	FFTFV	HARSGG	SVL	FVSGGGV	DTPDSSLSSRGIDRTPIVGLSPIPASPEDGTASTATTVQMFGGALYQALSPYVFFTFVHARSGGSVLFVSGGGV	10	0.60
AS (2020)_MLG24	Asia	DT	PDSSSLSRGI	DR	TPIVGLSQIPA	SPEDGTASTATTVQM	YGG	AL	YQ	AP	SPYV	LFTFV	QARSVG	SVS	LVSGDGV	DTPDSSSLSRGIDRTPIVGLSQIPASPEDGTASTATTVQMYGGALYQAPSPYVLFTFVQARSVGSVSLVSGDGV	9	0.60
AS (2020)_MLG25	Asia	DT	PDSSSSSKRI	DR	TPIVGLSQVPA	SPEDGTASTATTVQM	FGG	AL	YQ	AL	SPYV	LFTFV	QARSGG	SVL	LVSGGGV	DTPDSSSSSKRIDRTPIVGLSQVPASPEDGTASTATTVQMFGGALYQALSPYVLFTFVQARSGGSVLLVSGGGV	9	0.60
AS (2020)_MLG26	Asia	DT	PDSSSSSKRI	DR	TPIVGLSQIPA	SPEDGTDSTATTVQM	FGG	AL	YQ	AP	SPYV	LFTFV	QARSGG	SVL	LVSGGGV	DTPDSSSSSKRIDRTPIVGLSQIPASPEDGTDSTATTVQMFGGALYQAPSPYVLFTFVQARSGGSVLLVSGGGV	8	0.50
AS (2020)_MLG27	Asia	DT	LDSSSSSRGI	DR	TPIVGLSQIPA	SPEDGTASKATTVQM	FGG	AL	YQ	VP	SPYV	LFTFV	QARSGG	SVL	LVSGDGV	DTLDSSSSSRGIDRTPIVGLSQIPASPEDGTASKATTVQMFGGALYQVPSPYVLFTFVQARSGGSVLLVSGDGV	8	0.50
AS (2020)_MLG28	Asia	DT	PDSSSSSRGI	DR	TPIVGLSPIPA	SPEDGTASTATTVQM	FGG	AL	YQ	AL	SPYV	FFIFV	HARSGG	SVL	FVSGGGV	DTPDSSSSSRGIDRTPIVGLSPIPASPEDGTASTATTVQMFGGALYQALSPYVFFIFVHARSGGSVLFVSGGGV	7	0.40
AS (2020)_MLG29	Asia	DT	PDSSSSSKRI	DR	TPFVGLSQIPA	SPEDGTASTATTVHM	FGG	AL	YQ	AL	SPYV	LFTFV	QARSGG	SVL	LVSGGGV	DTPDSSSSSKRIDRTPFVGLSQIPASPEDGTASTATTVHMFGGALYQALSPYVLFTFVQARSGGSVLLVSGGGV	7	0.40
AS (2020)_MLG30	Asia	DT	PDSSSSSRGI	DR	TPIIGLSQIPA	SPEDGTASTATTVQM	FGG	AF	YQ	AP	SPYV	LFTFV	QARSGG	SVL	LVSGDGV	DTPDSSSSSRGIDRTPIIGLSQIPASPEDGTASTATTVQMFGGAFYQAPSPYVLFTFVQARSGGSVLLVSGDGV	7	0.40
AS (2020)_MLG31	Asia	DT	PDSSSSSKRI	DR	TPIVGLSQIPA	SPEDGTASTATTVQM	FGS	AF	YQ	AL	SPYV	LFTFV	QARSGG	SVL	LVSGGGV	DTPDSSSSSKRIDRTPIVGLSQIPASPEDGTASTATTVQMFGSAFYQALSPYVLFTFVQARSGGSVLLVSGGGV	6	0.40
AS (2020)_MLG32	Asia	DT	PDSSSSSRGI	DR	TPIVGLSQIPA	SPEDGTASTATTVQM	FGG	AF	YQ	AL	SPYV	LFTFV	QARSGG	SVL	LVSGGGV	DTPDSSSSSRGIDRTPIVGLSQIPASPEDGTASTATTVQMFGGAFYQALSPYVLFTFVQARSGGSVLLVSGGGV	6	0.40
AS (2020)_MLG33	Asia	DT	PDSSSSSRGI	DR	TPIVGLSPIPA	SPEDGTASTATTVQM	FGG	AL	YQ	AL	SPYV	FFTFV	HARSGG	SVL	FVSGGGV	DTPDSSSSSRGIDRTPIVGLSPIPASPEDGTASTATTVQMFGGALYQALSPYVFFTFVHARSGGSVLFVSGGGV	6	0.40
AS (2020)_MLG34	Asia	DT	PDSSSSSRGI	DR	TPIVGLSQIPA	SPEDGTASTATTVQM	FGG	AL	YQ	AP	SLCV	LFTFV	QARSGG	SVS	LVSGDGV	DTPDSSSSSRGIDRTPIVGLSQIPASPEDGTASTATTVQMFGGALYQAPSLCVLFTFVQARSGGSVSLVSGDGV	6	0.40
AS (2020)_MLG35	Asia	DT	PDSSLSSRGI	DR	TPIVGLSPIPA	SPEDGTASTATTVQM	FGG	AL	YQ	AP	SPYV	FFTFV	HARSGG	SVL	FVSGGGV	DTPDSSLSSRGIDRTPIVGLSPIPASPEDGTASTATTVQMFGGALYQAPSPYVFFTFVHARSGGSVLFVSGGGV	6	0.40
AS (2020)_MLG36	Asia	DT	PDSSSSSRGI	DR	TPIVGLSQIPA	SPEDGTASTATTVQM	FGG	AF	YQ	AP	SPYV	LFTFV	QARSGG	SVL	LVSGDGV	DTPDSSSSSRGIDRTPIVGLSQIPASPEDGTASTATTVQMFGGAFYQAPSPYVLFTFVQARSGGSVLLVSGDGV	5	0.30
AS (2020)_MLG37	Asia	DT	LDSSSSSRGI	DR	TPIVGLSQIPA	SPEDGTASKATTGQM	FGG	AL	YQ	VP	SPYV	LFTFV	QARSGG	SVL	LVSGDGV	DTLDSSSSSRGIDRTPIVGLSQIPASPEDGTASKATTGQMFGGALYQVPSPYVLFTFVQARSGGSVLLVSGDGV	5	0.30
AS (2020)_MLG38	Asia	DT	PDSSSSSRGI	DR	TPIVGLSQVSA	SPEDGTASTATTVQM	FGG	AF	YQ	AP	SPYV	LFTFV	QARSGV	SVL	LVSGDGV	DTPDSSSSSRGIDRTPIVGLSQVSASPEDGTASTATTVQMFGGAFYQAPSPYVLFTFVQARSGVSVLLVSGDGV	5	0.30
AS (2020)_MLG39	Asia	DT	PDSSSSSRGI	DR	TPIVGLSPIPA	SPEDGTASTATTVQM	FGG	AL	YQ	AL	SPYV	FFTFV	QARSGG	SVL	FVSGGGV	DTPDSSSSSRGIDRTPIVGLSPIPASPEDGTASTATTVQMFGGALYQALSPYVFFTFVQARSGGSVLFVSGGGV	5	0.30
AS (2020)_MLG40	Asia	DT	PDSSSSSRGI	DR	TPIVGLSQIPA	SPEDGTASTATTVQM	FGG	AL	YQ	AL	SPYV	LFTFV	QARSGG	SVL	LVSGDGV	DTPDSSSSSRGIDRTPIVGLSQIPASPEDGTASTATTVQMFGGALYQALSPYVLFTFVQARSGGSVLLVSGDGV	4	0.30
AS (2020)_MLG41	Asia	DT	PDSSSSSRGI	ER	TPIVGLSQIPA	SLEDGTASTATTVQM	FGG	AL	YQ	AP	SPYV	LLTFV	QARSGG	SLS	LVSGDGV	DTPDSSSSSRGIERTPIVGLSQIPASLEDGTASTATTVQMFGGALYQAPSPYVLLTFVQARSGGSLSLVSGDGV	4	0.30
AS (2020)_MLG42	Asia	DT	PDSSLSSKRI	DR	TPIVGLSQIPA	SPEDGTASTATTVQM	FGS	AL	YQ	AL	SPYV	LFTFV	QARSGG	SVL	LVSGGGV	DTPDSSLSSKRIDRTPIVGLSQIPASPEDGTASTATTVQMFGSALYQALSPYVLFTFVQARSGGSVLLVSGGGV	4	0.30
AS (2020)_MLG43	Asia	DT	PDSSSSSRGI	DR	TPIVGLSQIPA	SPEDGTASTATTVQM	FGG	AL	YQ	AP	SPYV	LFTFV	QARSGG	SLS	LVSGDGV	DTPDSSSSSRGIDRTPIVGLSQIPASPEDGTASTATTVQMFGGALYQAPSPYVLFTFVQARSGGSLSLVSGDGV	4	0.30
AS (2020)_MLG44	Asia	DT	LDSSSSSRGI	DR	TPIVGLSQIPA	SPEDGTASTATTGQM	FGG	AL	YQ	VP	SPYV	LFTFV	QARSGG	SVL	LVSGDGV	DTLDSSSSSRGIDRTPIVGLSQIPASPEDGTASTATTGQMFGGALYQVPSPYVLFTFVQARSGGSVLLVSGDGV	3	0.20
AS (2020)_MLG45	Asia	GT	PDSSSSSRGI	DR	TPIVGLSQIPA	SPEDGTASTATTVQM	FGG	AL	YQ	AL	SPYV	LFTFV	QARSGG	SVL	LVSGGGV	GTPDSSSSSRGIDRTPIVGLSQIPASPEDGTASTATTVQMFGGALYQALSPYVLFTFVQARSGGSVLLVSGGGV	3	0.20
AS (2020)_MLG46	Asia	DT	PDSSSSSKRI	DR	TPIVGLSQIPA	SPEDGTASTATTVQM	FGG	AL	YQ	AP	SPYV	LFTFV	QARSGG	SVL	LVSGGGV	DTPDSSSSSKRIDRTPIVGLSQIPASPEDGTASTATTVQMFGGALYQAPSPYVLFTFVQARSGGSVLLVSGGGV	3	0.20
AS (2020)_MLG47	Asia	DT	LDSSSSSRGI	DR	TPIVGLFQIPA	SPEDGTARKATTVQM	FGG	AF	YQ	VP	SPYV	LFTFV	QARSGG	SVL	LVSGDGV	DTLDSSSSSRGIDRTPIVGLFQIPASPEDGTARKATTVQMFGGAFYQVPSPYVLFTFVQARSGGSVLLVSGDGV	3	0.20
AS (2020)_MLG48	Asia	DT	LDSSSSSRGI	DR	TPIVGLSQIPA	SPEDGTASKATTGQM	FGG	AF	YQ	VP	SPYV	LFTFV	QARSGG	SVL	LVSGDGV	DTLDSSSSSRGIDRTPIVGLSQIPASPEDGTASKATTGQMFGGAFYQVPSPYVLFTFVQARSGGSVLLVSGDGV	3	0.20
AS (2020)_MLG49	Asia	DT	PDSSSSSRGI	DR	TPIVGLSQIPA	SPEDGTASTATTVQM	FGG	AF	YQ	AP	SPYV	LFTFV	QARSGG	SVS	LVSGDGV	DTPDSSSSSRGIDRTPIVGLSQIPASPEDGTASTATTVQMFGGAFYQAPSPYVLFTFVQARSGGSVSLVSGDGV	3	0.20
AS (2020)_MLG50	Asia	DT	PDSSSSSRGI	DR	TPIVGLSQIPA	SPEDGTDSTATTGQM	FGG	AL	YQ	AL	SPYV	LFTFV	QARSGG	SVL	LVSGGGV	DTPDSSSSSRGIDRTPIVGLSQIPASPEDGTDSTATTGQMFGGALYQALSPYVLFTFVQARSGGSVLLVSGGGV	3	0.20
AS (2020)_MLG51	Asia	DT	PDSSSSSRGI	DR	TPIVGLSPIPA	SPEDGTASTATTVQM	FGG	AL	YQ	AL	SPYV	LFTFV	QARSGG	SVL	LVSGGGV	DTPDSSSSSRGIDRTPIVGLSPIPASPEDGTASTATTVQMFGGALYQALSPYVLFTFVQARSGGSVLLVSGGGV	3	0.20
AS (2020)_MLG52	Asia	DT	LDSSSSSRGI	DR	TPIVGLSQIPA	SPEDGTASKATTVQM	FGG	AF	YQ	VP	SPYV	LFTFV	QAKSGG	SVL	LVSGDGV	DTLDSSSSSRGIDRTPIVGLSQIPASPEDGTASKATTVQMFGGAFYQVPSPYVLFTFVQAKSGGSVLLVSGDGV	3	0.20
AS (2020)_MLG53	Asia	DT	LDSSSSSRGI	DR	TPIVGLSQIPA	SPEDGTARKATTVQM	FGG	AL	YQ	VL	SPYV	LFTFV	QARSGG	SVL	LVSGDGV	DTLDSSSSSRGIDRTPIVGLSQIPASPEDGTARKATTVQMFGGALYQVLSPYVLFTFVQARSGGSVLLVSGDGV	3	0.20
AS (2020)_MLG54	Asia	DT	LDSSSSSRGI	DR	TPIVGLSQIPA	SPEDGTARKATTVQM	FGG	AL	YQ	VP	SPYV	LFTFV	QARSGG	SVL	LVSGDGV	DTLDSSSSSRGIDRTPIVGLSQIPASPEDGTARKATTVQMFGGALYQVPSPYVLFTFVQARSGGSVLLVSGDGV	3	0.20
AS (2020)_MLG55	Asia	DT	PDSSLSSRGI	DR	TPIVGLSQIPA	SPEDGTASTATTVQM	FGG	AL	YQ	AL	SPYV	LFTFV	HARSGG	SLL	LVSGGGV	DTPDSSLSSRGIDRTPIVGLSQIPASPEDGTASTATTVQMFGGALYQALSPYVLFTFVHARSGGSLLLVSGGGV	2	0.10
AS (2020)_MLG56	Asia	DT	LDSSSSSRGI	DR	TPIVGLSQIPA	SPEDGTASTATTVQM	FGG	AF	YQ	VP	SPYV	LFTFV	QARSGG	SVL	LVSGDGV	DTLDSSSSSRGIDRTPIVGLSQIPASPEDGTASTATTVQMFGGAFYQVPSPYVLFTFVQARSGGSVLLVSGDGV	2	0.10
AS (2020)_MLG57	Asia	DT	LDSSSSSRGI	DR	TPIVGLSQIPA	SSEDGTASTATTVQM	FGG	AL	YQ	VP	SPYV	LFTFV	QARSGG	SVL	LVSGDGV	DTLDSSSSSRGIDRTPIVGLSQIPASSEDGTASTATTVQMFGGALYQVPSPYVLFTFVQARSGGSVLLVSGDGV	2	0.10
AS (2020)_MLG58	Asia	DT	PDSSSSSRGI	DR	TPIVGLSQIPA	SPEDGTASTATTVQM	FGG	AL	YQ	AL	SPYV	LFTFV	QARSGG	SVS	LVSGDGV	DTPDSSSSSRGIDRTPIVGLSQIPASPEDGTASTATTVQMFGGALYQALSPYVLFTFVQARSGGSVSLVSGDGV	2	0.10
AS (2020)_MLG59	Asia	DT	PDSSLSSRGI	DR	TPIVGLSQIPA	SPEDGTASTATTVQM	FGG	AL	YQ	AL	SPYV	LFIFV	QARSGG	SVL	LVSGGGV	DTPDSSLSSRGIDRTPIVGLSQIPASPEDGTASTATTVQMFGGALYQALSPYVLFIFVQARSGGSVLLVSGGGV	2	0.10

AS (2020)_MLG60	Asia	DT	PDSSLSSRGI	DR	TPIVGLSQIPA	SPEDGTASTATTVQM	FGG	AL	YQ	AL	SPYV	LFIFV	HARSGG	SVL	FVSGGGV	DTPDSSLSSRGIDRTPIVGLSQIPASPEDGTASTATTVQMFGGALYQALSPYVLFIFVHARSGGSVLFVSGGGV	2	0.10
AS (2020)_MLG61	Asia	DT	PDSSSSSRGI	DR	TPIVGLSQIPA	SPEDGTASTATTVQM	FGG	AL	YQ	AL	SPYV	FFTFV	QARSGG	SVL	LVSGGGV	DTPDSSSSSRGIDRTPIVGLSQIPASPEDGTASTATTVQMFGGALYQALSPYVFFTFVQARSGGSVLLVSGGGV	2	0.10
AS (2020)_MLG62	Asia	DT	PDSSSSNRGI	DR	TPIVGLSPIPA	SPEDGTASTATTVQM	FGG	AL	YQ	AP	SPYV	FFTFV	HARSGG	SVL	FVSGGGV	DTPDSSSSNRGIDRTPIVGLSPIPASPEDGTASTATTVQMFGGALYQAPSPYVFFTFVHARSGGSVLFVSGGGV	2	0.10
AS (2020)_MLG63	Asia	DT	PDSSSSSRGI	DR	IPIVGLSQIPA	SPEDGTASTATTGQM	FGG	AL	YQ	AL	SPYV	LFTFV	HARSGG	SVL	LVSGGGV	DTPDSSSSSRGIDRIPIVGLSQIPASPEDGTASTATTGQMFGGALYQALSPYVLFTFVHARSGGSVLLVSGGGV	2	0.10
AS (2020)_MLG64	Asia	DT	PDSSSSSKRI	DR	TPIVGLSQIPA	SPEDGTDSTATTVQM	FGG	AL	YQ	AL	SPYV	LFTFV	QARSGG	SVL	LVSGGVV	DTPDSSSSSKRIDRTPIVGLSQIPASPEDGTDSTATTVQMFGGALYQALSPYVLFTFVQARSGGSVLLVSGGVV	2	0.10
AS (2020)_MLG65	Asia	DT	PDSSSSSKRI	DR	TPIVGLSQIPA	SPEDGTASTATTVQM	FGG	AL	YQ	AL	SPYV	LFTFV	QARSGG	SVL	LVSGGGF	DTPDSSSSSKRIDRTPIVGLSQIPASPEDGTASTATTVQMFGGALYQALSPYVLFTFVQARSGGSVLLVSGGGF	2	0.10
AS (2020)_MLG66	Asia	DT	PDSSSSSRGI	DR	TPIVGLSQIPA	SPEDGTASTATTGQM	FGG	AL	YQ	AP	SPYV	LFTFV	QARSGG	SVL	LVSGDGV	DTPDSSSSSRGIDRTPIVGLSQIPASPEDGTASTATTGQMFGGALYQAPSPYVLFTFVQARSGGSVLLVSGDGV	2	0.10
AS (2020)_MLG67	Asia	DT	LDSSSSSRGI	DR	TPIVGLSQIPA	SPEDGTASTATTVQM	FGG	AL	YQ	VP	SPYV	LFTFV	QARSGG	SVL	LVSGDGV	DTLDSSSSSRGIDRTPIVGLSQIPASPEDGTASTATTVQMFGGALYQVPSPYVLFTFVQARSGGSVLLVSGDGV	2	0.10
AS (2020)_MLG68	Asia	DT	LDSSSSSRGI	DR	TPIVGLSQIPA	SPEDGTARKATTVQM	FGG	AF	YQ	AP	SPYV	LFTFV	QARSGG	SVL	LVSGDGV	DTLDSSSSSRGIDRTPIVGLSQIPASPEDGTARKATTVQMFGGAFYQAPSPYVLFTFVQARSGGSVLLVSGDGV	2	0.10
AS (2020)_MLG69	Asia	DT	PDSSSSSRGI	DR	TPIIGLSQIPA	SPEDGTASTATTGQM	FGG	AL	YQ	AP	SPYV	LFTFV	QARSGG	SVL	LVSGDGV	DTPDSSSSSRGIDRTPIIGLSQIPASPEDGTASTATTGQMFGGALYQAPSPYVLFTFVQARSGGSVLLVSGDGV	2	0.10
AS (2020)_MLG70	Asia	DT	PDSSSSSKRI	DR	TPIVGLSQIPA	SPEDGTASTATTGQM	FGG	AL	YQ	AL	SPYV	LFTFV	QARSGG	SVL	LVSGGGV	DTPDSSSSSKRIDRTPIVGLSQIPASPEDGTASTATTGQMFGGALYQALSPYVLFTFVQARSGGSVLLVSGGGV	2	0.10
AS (2020)_MLG71	Asia	DT	PDSSSSSRGI	DR	TPIVDLSQIPA	SPEDGTASTATTVQM	FGG	AL	YQ	AP	SPYV	LFTFV	QARSGG	SVL	LVSGDGV	DTPDSSSSSRGIDRTPIVDLSQIPASPEDGTASTATTVQMFGGALYQAPSPYVLFTFVQARSGGSVLLVSGDGV	2	0.10
AS (2020)_MLG72	Asia	DT	PDSSSSSRGI	DR	TPIVGLSQIPA	SPEDGTASTATTGQM	FGG	AL	YQ	AL	SPYV	FFTFV	QARSGG	SVL	LVSGGGV	DTPDSSSSSRGIDRTPIVGLSQIPASPEDGTASTATTGQMFGGALYQALSPYVFFTFVQARSGGSVLLVSGGGV	2	0.10
AS (2020)_MLG73	Asia	DT	PDSSSSNRGI	DR	TPIVGLSPIPA	SPEDGTASTATTVQM	FGG	AL	YQ	AP	SPYV	FFTFV	QARSGG	SVS	LVSGGGV	DTPDSSSSNRGIDRTPIVGLSPIPASPEDGTASTATTVQMFGGALYQAPSPYVFFTFVQARSGGSVSLVSGGGV	2	0.10
AS (2020)_MLG74	Asia	DT	PDSSSSSRGI	DR	TPIVGLSQIPA	SPEDGTASTATTVQM	FGG	AL	YQ	AP	SPYV	LFTFV	HARSGG	SVL	LVSGGGV	DTPDSSSSSRGIDRTPIVGLSQIPASPEDGTASTATTVQMFGGALYQAPSPYVLFTFVHARSGGSVLLVSGGGV	2	0.10
AS (2020)_MLG75	Asia	DT	PDSSSSSKRI	DR	TPIVGLSQIPA	SPEDGTDSTATTVQM	FGG	AF	YQ	AL	SPYV	LFTFV	QARSGG	SVL	LVSGGGV	DTPDSSSSSKRIDRTPIVGLSQIPASPEDGTDSTATTVQMFGGAFYQALSPYVLFTFVQARSGGSVLLVSGGGV	2	0.10
AS (2020)_MLG76	Asia	DT	PDSSLSSRGI	DR	TPIVGLSPIPA	SPEDGTASTATTVQM	FGG	AL	YQ	AP	SPYV	FFIFV	HARSGG	SVL	FVSGGGV	DTPDSSLSSRGIDRTPIVGLSPIPASPEDGTASTATTVQMFGGALYQAPSPYVFFIFVHARSGGSVLFVSGGGV	2	0.10
AS (2020)_MLG77	Asia	DT	PDSSSSSRGI	DR	TPIVGLSQIPA	SPEDGTASTATTVQM	FGG	AL	YQ	AL	SPYV	FFTFV	HARSGG	SVL	LVSGGGV	DTPDSSSSSRGIDRTPIVGLSQIPASPEDGTASTATTVQMFGGALYQALSPYVFFTFVHARSGGSVLLVSGGGV	2	0.10
AS (2020)_MLG78	Asia	DT	PDSSSSSRGI	DR	TPIVGLSQIPA	SPEDGTASTATTVQM	FGG	AL	YQ	AL	SPYV	FFTFV	HARSGG	SVL	FVSGGGV	DTPDSSSSSRGIDRTPIVGLSQIPASPEDGTASTATTVQMFGGALYQALSPYVFFTFVHARSGGSVLFVSGGGV	2	0.10
AS (2020)_MLG79	Asia	DT	PDSSLSSRGI	DR	TPIVGLSQIPA	SPEDGTASTATTVQM	FGG	AL	YQ	AL	SPYV	LFIFV	HARSGG	SVL	LVSGGGV	DTPDSSLSSRGIDRTPIVGLSQIPASPEDGTASTATTVQMFGGALYQALSPYVLFIFVHARSGGSVLLVSGGGV	2	0.10
AS (2020)_MLG80	Asia	DT	PDSSSSSKRI	DR	TPFVGLSQIPA	SPEDGTASTATTVQM	FGG	AL	YQ	AL	SPYV	LFTFV	QARSGG	SVL	LVSGGGV	DTPDSSSSSKRIDRTPFVGLSQIPASPEDGTASTATTVQMFGGALYQALSPYVLFTFVQARSGGSVLLVSGGGV	2	0.10
AS (2020)_MLG81	Asia	DT	PDSSSSSRGI	DR	TPIVGLSQIPA	SPEDGTASTATTVQM	FGG	AF	YQ	AL	SPYV	LFTFV	HARSGG	SVL	LVSGGGV	DTPDSSSSSRGIDRTPIVGLSQIPASPEDGTASTATTVQMFGGAFYQALSPYVLFTFVHARSGGSVLLVSGGGV	2	0.10
AS (2020)_MLG82	Asia	DT	PDSSSSSKRI	DR	TPIVGLSQIPA	SPEDGTASTATTVQM	FGG	AL	YQ	AL	SPYV	LFTFV	QARSGG	SVL	FVSGGGV	DTPDSSSSSKRIDRTPIVGLSQIPASPEDGTASTATTVQMFGGALYQALSPYVLFTFVQARSGGSVLFVSGGGV	2	0.10
AS (2020)_MLG83	Asia	DT	PDSSSSSRGI	DR	IPIVGLSQIPA	SPEDGTASTATTVQM	FGG	AF	YQ	AL	SPYV	LFTFV	HARSGG	SVL	LVSGGGV	DTPDSSSSSRGIDRIPIVGLSQIPASPEDGTASTATTVQMFGGAFYQALSPYVLFTFVHARSGGSVLLVSGGGV	2	0.10
AS (2020)_MLG84	Asia	DT	PDSSSSSRGI	DR	TPIVGLSQIPA	SPEDGTASKATTVQM	FGG	AL	YQ	AP	SPYV	LFTFV	QARSGG	SVL	LVSGDGV	DTPDSSSSSRGIDRTPIVGLSQIPASPEDGTASKATTVQMFGGALYQAPSPYVLFTFVQARSGGSVLLVSGDGV	2	0.10
AS (2020)_MLG85	Asia	DT	PDSSSSSKGI	DR	TPIVGLSQIPA	SPEDGTASTATTVQM	FGG	AL	YQ	AP	SPYV	LFTFV	QARSGG	SVL	LVSGGGV	DTPDSSSSSKGIDRTPIVGLSQIPASPEDGTASTATTVQMFGGALYQAPSPYVLFTFVQARSGGSVLLVSGGGV	2	0.10
AS (2020)_MLG86	Asia	DT	PDSSSSNRGI	DR	TPIVGLSQVPA	SPEDGTASTATTVQM	FGG	AL	YQ	AP	SPYV	LFTFV	QARSGG	SVS	LVSGDGV	DTPDSSSSNRGIDRTPIVGLSQVPASPEDGTASTATTVQMFGGALYQAPSPYVLFTFVQARSGGSVSLVSGDGV	2	0.10
AS (2020)_MLG87	Asia	DT	PDSSLSSRGI	DR	TPIVGLSQIPA	SPEDGTASTATTVQM	FGG	AL	YQ	AP	SPYV	LFTFV	HARSGG	SVL	LVSGGGV	DTPDSSLSSRGIDRTPIVGLSQIPASPEDGTASTATTVQMFGGALYQAPSPYVLFTFVHARSGGSVLLVSGGGV	1	0.10
AS (2020)_MLG88	Asia	DT	PDSSSSSKRI	DR	TPIVGLSQIPA	SPEDGTDSTATNVQM	FGG	AL	YQ	AL	SPYV	LFTFV	QARSGG	SVL	LVSGGGV	DTPDSSSSSKRIDRTPIVGLSQIPASPEDGTDSTATNVQMFGGALYQALSPYVLFTFVQARSGGSVLLVSGGGV	1	0.10
AS (2020)_MLG89	Asia	DT	PDSSSSSRGI	DR	TPIVGLSQIPA	SPEDGTAGTATTVQM	FGG	AL	YQ	AP	SPYV	LFTFV	QARSGG	SVS	LVSGDGV	DTPDSSSSSRGIDRTPIVGLSQIPASPEDGTAGTATTVQMFGGALYQAPSPYVLFTFVQARSGGSVSLVSGDGV	1	0.10
AS (2020)_MLG90	Asia	DT	PDSSSSSRGI	DR	IPIVGLSQIPA	SPEDGTASTATTVQM	FGG	AL	YQ	AL	SLYV	LFTFV	HARSGG	SVL	LVSGGGV	DTPDSSSSSRGIDRIPIVGLSQIPASPEDGTASTATTVQMFGGALYQALSLYVLFTFVHARSGGSVLLVSGGGV	1	0.10
AS (2020)_MLG91	Asia	DT	PYSSLSSRGI	DR	TPIVGLSQIPA	SPEDGTASTATTVQM	FGG	AL	YQ	AL	SPYV	LFTFV	HARSGG	SVL	LVSGGGV	DTPYSSLSSRGIDRTPIVGLSQIPASPEDGTASTATTVQMFGGALYQALSPYVLFTFVHARSGGSVLLVSGGGV	1	0.10
AS (2020)_MLG92	Asia	DT	LDSSSSSRGI	DR	TPIVGLSQIPA	SPEDGTASKATTVQM	FGG	AL	YQ	VL	SPYV	LFTFV	HARSGG	SVL	LVSGDGV	DTLDSSSSSRGIDRTPIVGLSQIPASPEDGTASKATTVQMFGGALYQVLSPYVLFTFVHARSGGSVLLVSGDGV	1	0.10
AS (2020)_MLG93	Asia	DT	PDFSSSSKRI	DR	TPIVGLSQIPA	SPEDGTDSTATTVQM	FGG	AL	YQ	AL	SPYV	LFTFV	QARSGG	SVL	LVSGGGV	DTPDFSSSSKRIDRTPIVGLSQIPASPEDGTDSTATTVQMFGGALYQALSPYVLFTFVQARSGGSVLLVSGGGV	1	0.10
AS (2020)_MLG94	Asia	DT	PDSSSSSKRI	DR	TPIVGLSPIPA	SPEDGTDSTATTVQM	FGG	AL	YQ	AL	SPYV	LFTFV	HARSGG	SVL	LVSGGGV	DTPDSSSSSKRIDRTPIVGLSPIPASPEDGTDSTATTVQMFGGALYQALSPYVLFTFVHARSGGSVLLVSGGGV	1	0.10
AS (2020)_MLG95	Asia	DT	PDSSLSSGRI	DR	TPIVGLSQIPA	SPEDGTASTATTVQM	FGG	AL	YQ	AL	SPYV	FFIFV	QARSGG	SVL	FVSGGGV	DTPDSSLSSGRIDRTPIVGLSQIPASPEDGTASTATTVQMFGGALYQALSPYVFFIFVQARSGGSVLFVSGGGV	1	0.10
AS (2020)_MLG96	Asia	DT	PDSSLSSRGI	DR	TPIVGLSPIPA	SPEDGTASTATTVQM	FGG	AL	YQ	AL	SPYV	LFTFV	HARSGG	SVL	LVSGGGV	DTPDSSLSSRGIDRTPIVGLSPIPASPEDGTASTATTVQMFGGALYQALSPYVLFTFVHARSGGSVLLVSGGGV	1	0.10
AS (2020)_MLG97	Asia	DT	PDSSLSSRGI	DR	TPIVGLSQIPA	SPEDGTASTATTVQM	FGG	AL	YQ	AL	SPYV	FFIFV	HARSGG	SVL	FVSGGGV	DTPDSSLSSRGIDRTPIVGLSQIPASPEDGTASTATTVQMFGGALYQALSPYVFFIFVHARSGGSVLFVSGGGV	1	0.10
AS (2020)_MLG98	Asia	DT	PDSSSSSRGI	DR	TPIVGLSPIPA	SPEDGTASTATTVQM	FGG	AL	YQ	AP	SPYV	FFTFV	HARSGG	SVL	FVSGGGV	DTPDSSSSSRGIDRTPIVGLSPIPASPEDGTASTATTVQMFGGALYQAPSPYVFFTFVHARSGGSVLFVSGGGV	1	0.10
AS (2020)_MLG99	Asia	DT	PDSSSSSRGI	DR	TPIVGLSPIPA	SPEDGTASTATTVQM	FGG	AL	YQ	AL	SPYV	LFIFV	HARSGG	SVL	FVSGGGV	DTPDSSSSSRGIDRTPIVGLSPIPASPEDGTASTATTVQMFGGALYQALSPYVLFIFVHARSGGSVLFVSGGGV	1	0.10
AS (2020)_MLG100	Asia	DT	PDSSLSSRGI	DR	TPIVGLSQIPA	SPEDGTASTATTVQM	FGG	AL	YQ	AL	SPYV	LFIFV	QARSGG	SVS	LVSGDGV	DTPDSSLSSRGIDRTPIVGLSQIPASPEDGTASTATTVQMFGGALYQALSPYVLFIFVQARSGGSVSLVSGDGV	1	0.10
AS (2020)_MLG101	Asia	DT	PDSSSSSRGI	DR	TPIVGLSQIPA	SPEDGTASTATTVQM	FGG	AF	YQ	VP	SPYV	LFTFV	QARSGG	SVL	LVSGDGV	DTPDSSSSSRGIDRTPIVGLSQIPASPEDGTASTATTVQMFGGAFYQVPSPYVLFTFVQARSGGSVLLVSGDGV	1	0.10
AS (2020)_MLG102	Asia	DT	PDSSSSSKRI	DR	TPIVGLSQIPA	SPEDGTASTATTVQM	FGG	AL	YQ	AL	SPYV	LFIFV	QARSGG	SVL	LVSGGGV	DTPDSSSSSKRIDRTPIVGLSQIPASPEDGTASTATTVQMFGGALYQALSPYVLFIFVQARSGGSVLLVSGGGV	1	0.10
AS (2020)_MLG103	Asia	DT	PDSSSSSKGI	DR	TPIVGLSPIPA	SPEDGTASTATTVQM	FGG	AL	YQ	AL	SPYV	FFTFV	HARSGG	SVL	FVSGGGV	DTPDSSSSSKGIDRTPIVGLSPIPASPEDGTASTATTVQMFGGALYQALSPYVFFTFVHARSGGSVLFVSGGGV	1	0.10
AS (2020)_MLG104	Asia	DT	PDSSLSSKTI	DR	TPIVGLSQIPA	SPEDGTASTATTVQM	FGG	AL	YQ	AP	SPYV	LFIFV	HARSGG	SVL	LVSGGGV	DTPDSSLSSKTIDRTPIVGLSQIPASPEDGTASTATTVQMFGGALYQAPSPYVLFIFVHARSGGSVLLVSGGGV	1	0.10
AS (2020)_MLG105	Asia	DT	LDSSSSSRGI	DR	TPIVGLSQIPA	SPEDGTASKATTVQM	FGG	AL	YQ	AP	SPYV	LFTFV	QARSGG	SVL	LVSGDGV	DTLDSSSSSRGIDRTPIVGLSQIPASPEDGTASKATTVQMFGGALYQAPSPYVLFTFVQARSGGSVLLVSGDGV	1	0.10
AS (2020)_MLG106	Asia	DT	PDSSSLSRGI	DR	TPIVGLSQIPA	SPEDGTASTATTVQM	YGG	AF	YQ	AP	SPYV	LFTFV	QARSVG	SVS	LVSGDGV	DTPDSSSLSRGIDRTPIVGLSQIPASPEDGTASTATTVQMYGGAFYQAPSPYVLFTFVQARSVGSVSLVSGDGV	1	0.10
AS (2020)_MLG107	Asia	DT	LDSSSSSRGI	DR	TPIVGLSQIPA	SPEDGTARKATTVQM	FGG	AF	YQ	AL	SPYV	LFTFV	QARSGG	SVL	LVSGDGV	DTLDSSSSSRGIDRTPIVGLSQIPASPEDGTARKATTVQMFGGAFYQALSPYVLFTFVQARSGGSVLLVSGDGV	1	0.10
AS (2020)_MLG108	Asia	DT	LDSSSSSRGI	DR	TPIVGLSQIPA	SPEDGTASKATTVQM	FGG	AF	YQ	VP	SPYV	LFTFV	QARSGG	SLL	LVSGDGV	DTLDSSSSSRGIDRTPIVGLSQIPASPEDGTASKATTVQMFGGAFYQVPSPYVLFTFVQARSGGSLLLVSGDGV	1	0.10
AS (2020)_MLG109	Asia	DT	PDSSSSNRGI	DR	TPIVGLSPIPA	SPEDGTASTATTVQM	FGG	AL	YQ	AP	SPYV	FFIFV	HARSGG	SVS	FVSGGGV	DTPDSSSSNRGIDRTPIVGLSPIPASPEDGTASTATTVQMFGGALYQAPSPYVFFIFVHARSGGSVSFVSGGGV	1	0.10
AS (2020)_MLG110	Asia	DT	PDSSSSSRGI	DR	IPIVGLSQIPA	SPEDGTASTATTVQM	FGG	AL	YQ	AP	SPYV	LFTFV	QARSGG	SVL	LVSGDGV	DTPDSSSSSRGIDRIPIVGLSQIPASPEDGTASTATTVQMFGGALYQAPSPYVLFTFVQARSGGSVLLVSGDGV	1	0.10
AS (2020)_MLG111	Asia	DT	PDSSSSSRGI	DR	IPIVGLSQIPA	SPEDGTASTATTVQM	FGG	AL	YQ	AL	SPYV	FFTFV	HARSGG	SVL	LVSGGGV	DTPDSSSSSRGIDRIPIVGLSQIPASPEDGTASTATTVQMFGGALYQALSPYVFFTFVHARSGGSVLLVSGGGV	1	0.10
AS (2020)_MLG112	Asia	DT	PDSSSSSRGI	DR	IPIIGLSQIPA	SPEDGTASTATTVQM	FGG	AF	YQ	AP	SPYV	LFTFV	QARSGG	SVL	LVSGDGV	DTPDSSSSSRGIDRIPIIGLSQIPASPEDGTASTATTVQMFGGAFYQAPSPYVLFTFVQARSGGSVLLVSGDGV	1	0.10
AS (2020)_MLG113	Asia	DT	PDSSLSSRGI	DR	TPIVGLSQIPA	SPEDGTASTATTGQM	FGG	AL	YQ	AL	SPYV	LFTFV	HARSGG	SVL	LVSGGGV	DTPDSSLSSRGIDRTPIVGLSQIPASPEDGTASTATTGQMFGGALYQALSPYVLFTFVHARSGGSVLLVSGGGV	1	0.10
AS (2020)_MLG114	Asia	DT	LDSSSSSRGI	DR	TPIVGLSQIPA	SPEDGTASKATTVQM	FGG	AF	YQ	AL	SPYV	LFTFV	QARSGG	SVL	LVSGDGV	DTLDSSSSSRGIDRTPIVGLSQIPASPEDGTASKATTVQMFGGAFYQALSPYVLFTFVQARSGGSVLLVSGDGV	1	0.10
AS (2020)_MLG115	Asia	DT	PDSSSSSKRI	DR	TPIVGLSQIPA	SPEDGTASTATTVHM	FGG	AL	YQ	AL	SPYV	LFTFV	QARSGG	SVL	LVSGGGV	DTPDSSSSSKRIDRTPIVGLSQIPASPEDGTASTATTVHMFGGALYQALSPYVLFTFVQARSGGSVLLVSGGGV	1	0.10
AS (2020)_MLG116	Asia	DT	PDSSSLSRGI	DR	TPIVGLSQIPA	SPEDGTASTATTVQM	FGG	AL	YQ	AP	SPYV	LFTFV	QARSVG	SVS	LVSGDGV	DTPDSSSLSRGIDRTPIVGLSQIPASPEDGTASTATTVQMFGGALYQAPSPYVLFTFVQARSVGSVSLVSGDGV	1	0.10
AS (2020)_MLG117	Asia	DT	LDSSSSSRGI	DR	TPIVGLFQIPA	SPEDGTANKATTVQM	FGG	AF	YQ	VP	SPYV	LFTFV	QARSGG	SVL	LVSGDGV	DTLDSSSSSRGIDRTPIVGLFQIPASPEDGTANKATTVQMFGGAFYQVPSPYVLFTFVQARSGGSVLLVSGDGV	1	0.10
AS (2020)_MLG118	Asia	DT	PDSSSSSRGI	DR	TPIVGLFQIPA	SPEDGTARKATTVQM	FGG	AF	YH	VP	SPYV	LFTFV	QARSGG	SVL	LVSGDGV	DTPDSSSSSRGIDRTPIVGLFQIPASPEDGTARKATTVQMFGGAFYHVPSPYVLFTFVQARSGGSVLLVSGDGV	1	0.10
AS (2020)_MLG119	Asia	DT	PDSSSSSRGI	DR	TPIVGLSQIPA	SPEDGTASTATTVQM	FGG	AL	YQ	AP	SPYV	LFTFV	QARSGG	SVF	LVSGDGV	DTPDSSSSSRGIDRTPIVGLSQIPASPEDGTASTATTVQMFGGALYQAPSPYVLFTFVQARSGGSVFLVSGDGV	1	0.10
AS (2020)_MLG120	Asia	DT	PDSSSSSRGI	DR	TPIVGLSQIPA	SPEDGTASTATTVQT	FGG	AL	YQ	AP	SPYV	LFTFV	QARSGG	SVS	LVSGDGV	DTPDSSSSSRGIDRTPIVGLSQIPASPEDGTASTATTVQTFGGALYQAPSPYVLFTFVQARSGGSVSLVSGDGV	1	0.10
AS (2020)_MLG121	Asia	DT	PGSSSSSRGI	DR	TPIVGLSQIPA	SPEDGTASTATTVQM	FGG	AL	YQ	AP	SPYV	LFTFV	QARSGV	SVL	LVSGDGV	DTPGSSSSSRGIDRTPIVGLSQIPASPEDGTASTATTVQMFGGALYQAPSPYVLFTFVQARSGVSVLLVSGDGV	1	0.10
AS (2020)_MLG122	Asia	DT	PDSSSSSRGI	DR	TPIVGLSPIPA	SPEDGTASTATTVQM	FGG	AL	YQ	AL	SPYV	FFTFV	QARSGG	SVL	LVSGGGV	DTPDSSSSSRGIDRTPIVGLSPIPASPEDGTASTATTVQMFGGALYQALSPYVFFTFVQARSGGSVLLVSGGGV	1	0.10
AS (2020)_MLG123	Asia	DT	PDSSLSSRGI	DR	TPIVGLSQIPA	SPEDGTASTATTVQM	FGG	AL	YQ	AP	SPYV	LFTFV	QARSGG	SVL	LVSGDGV	DTPDSSLSSRGIDRTPIVGLSQIPASPEDGTASTATTVQMFGGALYQAPSPYVLFTFVQARSGGSVLLVSGDGV	1	0.10

AS (2020)_MLG124	Asia	DT	LDSSSSSRGI	DR	TPIVGLSQIPA	SPEDGTASKATTVQM	FGG	AF	YQ	AP	SPYV	LFTFV	QARSGG	SVL	LVSGDGV	DTLDSSSSSRGIDRTPIVGLSQIPASPEDGTASKATTVQMFGGAFYQAPSPYVLFTFVQARSGGSVLLVSGDGV	1	0.10
AS (2020)_MLG125	Asia	DT	LDSSSSSKRI	DR	TPIVGLSQIPA	SPEDGTASTATTVQM	FGG	AL	YQ	VP	SPYV	LFTFV	QARSGG	SVL	LVSGDGV	DTLDSSSSSKRIDRTPIVGLSQIPASPEDGTASTATTVQMFGGALYQVPSPYVLFTFVQARSGGSVLLVSGDGV	1	0.10
AS (2020)_MLG126	Asia	DT	PDSSLSSRGI	DR	TPIVGLSPIPA	SPEDGTASTATTVQM	FGG	AL	YQ	AL	SPYV	LFIFV	HARSGG	SVL	FVSGGGV	DTPDSSLSSRGIDRTPIVGLSPIPASPEDGTASTATTVQMFGGALYQALSPYVLFIFVHARSGGSVLFVSGGGV	1	0.10
AS (2020)_MLG127	Asia	DT	PDSSSSSRGI	DR	TPIVGLSQIPA	SPEDGTASTATTVQM	FGG	AL	YQ	AP	SPYV	LFTLV	QARSGG	SVL	LVSGDGV	DTPDSSSSSRGIDRTPIVGLSQIPASPEDGTASTATTVQMFGGALYQAPSPYVLFTLVQARSGGSVLLVSGDGV	1	0.10
AS (2020)_MLG128	Asia	DT	LDSSSSSRGI	DR	TPIVGLFQIPA	SPEDGTASKATTVQM	FGG	AF	YQ	VP	SPYV	LFTFV	QARSGG	SVL	LVSGDGV	DTLDSSSSSRGIDRTPIVGLFQIPASPEDGTASKATTVQMFGGAFYQVPSPYVLFTFVQARSGGSVLLVSGDGV	1	0.10
AS (2020)_MLG129	Asia	DT	PDSSSSNRGI	DR	TPIVGLSQIPA	SPEDGTASTATTVQM	FGG	AL	YQ	AL	SPYV	LFTFV	QARSGG	SVS	LVSGDGV	DTPDSSSSNRGIDRTPIVGLSQIPASPEDGTASTATTVQMFGGALYQALSPYVLFTFVQARSGGSVSLVSGDGV	1	0.10
AS (2020)_MLG130	Asia	DT	PDSSLSSRGI	DR	TPIVGLSQIPA	SPEDGTASTATTVQM	FGG	AF	YQ	AL	SPYV	LFTFV	HARSGG	SVL	LVSGGGV	DTPDSSLSSRGIDRTPIVGLSQIPASPEDGTASTATTVQMFGGAFYQALSPYVLFTFVHARSGGSVLLVSGGGV	1	0.10
AS (2020)_MLG131	Asia	DT	PNSSLSSRGI	DR	TPIVGLSQIPA	SPEDGTASTATTVQM	FGG	AL	YQ	AL	SPYV	LFIFV	QARSGG	SVL	LVSGGGV	DTPNSSLSSRGIDRTPIVGLSQIPASPEDGTASTATTVQMFGGALYQALSPYVLFIFVQARSGGSVLLVSGGGV	1	0.10
AS (2020)_MLG132	Asia	DT	PDSSSSSRGI	DR	TPIVGLSQIPA	SPEDGTASTATTVQM	FGG	AL	YQ	AL	SPYV	LFIFV	QARSGG	SVL	LVSGGGV	DTPDSSSSSRGIDRTPIVGLSQIPASPEDGTASTATTVQMFGGALYQALSPYVLFIFVQARSGGSVLLVSGGGV	1	0.10
AS (2020)_MLG133	Asia	DT	PDSSSSSRGI	DR	TPIVGLSQIPA	SPEDGTASTATTVQM	FGG	AL	YQ	VP	SPYV	LFTFV	QARSGG	SVL	LVSGDGV	DTPDSSSSSRGIDRTPIVGLSQIPASPEDGTASTATTVQMFGGALYQVPSPYVLFTFVQARSGGSVLLVSGDGV	1	0.10
AS (2020)_MLG134	Asia	DT	PDSSLSSRGI	DR	TPIVGLSQIPA	SPEDGTASTATTVQM	FGG	AL	YQ	AP	SPYV	LFIFV	HARSGG	SVL	LVSGGGV	DTPDSSLSSRGIDRTPIVGLSQIPASPEDGTASTATTVQMFGGALYQAPSPYVLFIFVHARSGGSVLLVSGGGV	1	0.10
AS (2020)_MLG135	Asia	DT	PDSSSSSRGI	DR	TPIVGLSQIPA	SPEDGTASTATTVHM	FGG	AL	YQ	AL	SPYV	LFTFV	HARSGG	SVL	LVSGGGV	DTPDSSSSSRGIDRTPIVGLSQIPASPEDGTASTATTVHMFGGALYQALSPYVLFTFVHARSGGSVLLVSGGGV	1	0.10
AS (2020)_MLG136	Asia	DT	PDSSSSSRGI	DR	TPIVGLSQIPA	SPEDGTASTATTVQM	FGG	AL	YQ	AL	SPYV	LFTFV	HARSGG	SVL	LVNGGGV	DTPDSSSSSRGIDRTPIVGLSQIPASPEDGTASTATTVQMFGGALYQALSPYVLFTFVHARSGGSVLLVNGGGV	1	0.10
AS (2020)_MLG137	Asia	DT	PDSSSSSRGI	DR	TPIVGLSPIPA	SPEDGTASTATTVQM	FGG	AL	YQ	AP	SPYV	LFIFV	QARSGG	SVL	LVSGGGV	DTPDSSSSSRGIDRTPIVGLSPIPASPEDGTASTATTVQMFGGALYQAPSPYVLFIFVQARSGGSVLLVSGGGV	1	0.10
AS (2020)_MLG138	Asia	DT	LDSSSSSRGI	DR	TPIVGLSQIPA	SSEDGTASTATTVQM	FGG	AF	YQ	VP	SPYV	LFTFV	QARSGG	SVL	LVSGDGV	DTLDSSSSSRGIDRTPIVGLSQIPASSEDGTASTATTVQMFGGAFYQVPSPYVLFTFVQARSGGSVLLVSGDGV	1	0.10
AS (2020)_MLG139	Asia	DT	PDSSSSNRGI	DR	TLIVGLSQIPA	SPEDGTASTATTVQM	FGG	AL	YQ	AP	SPYV	LFTFV	QARSGG	SVS	LVSGDGV	DTPDSSSSNRGIDRTLIVGLSQIPASPEDGTASTATTVQMFGGALYQAPSPYVLFTFVQARSGGSVSLVSGDGV	1	0.10
AS (2020)_MLG140	Asia	DT	PDSSSSSRGI	DR	TPIVGLSQIPA	SPEDGTASTATTVQM	FGG	AF	YQ	AL	SPYV	LFTFV	QARSGG	SVL	LVSGDGV	DTPDSSSSSRGIDRTPIVGLSQIPASPEDGTASTATTVQMFGGAFYQALSPYVLFTFVQARSGGSVLLVSGDGV	1	0.10
AS (2020)_MLG141	Asia	DT	PDSSSSSRGI	DR	TPIVGLSQIPA	SPEDGTASTATTVQM	FGG	AL	YQ	AP	SLCV	LFTFV	QARSGG	SVL	LVSGDGV	DTPDSSSSSRGIDRTPIVGLSQIPASPEDGTASTATTVQMFGGALYQAPSLCVLFTFVQARSGGSVLLVSGDGV	1	0.10
AS (2020)_MLG142	Asia	DT	PDSSSSSRGI	DR	TPIVGLSQIPA	SQVDGTASTATTVQM	FGG	AL	YQ	AP	SPYV	LFTFV	QARSGG	SVL	LVSGDGV	DTPDSSSSSRGIDRTPIVGLSQIPASQVDGTASTATTVQMFGGALYQAPSPYVLFTFVQARSGGSVLLVSGDGV	1	0.10
AS (2020)_MLG143	Asia	DT	PDSSSSSRGI	DR	TPIVGLSQIPA	SPEDGTASTATTGQM	FGG	AL	YQ	AL	SPYV	LFTFV	HARSGG	SVL	LVSGGGV	DTPDSSSSSRGIDRTPIVGLSQIPASPEDGTASTATTGQMFGGALYQALSPYVLFTFVHARSGGSVLLVSGGGV	1	0.10
AS (2020)_MLG144	Asia	DT	LDSSSSSRGI	DR	TPIVGLSQIPA	SPEDGTASTATTVQM	FGG	AF	YQ	AP	SPYV	LFTFV	QARSGG	SVL	LVSGDGV	DTLDSSSSSRGIDRTPIVGLSQIPASPEDGTASTATTVQMFGGAFYQAPSPYVLFTFVQARSGGSVLLVSGDGV	1	0.10
AS (2020)_MLG145	Asia	DT	PDSSSSSRGI	DR	TPIVGLSQIPA	SPEGGTASTATTVQM	FGG	AL	YQ	AL	SPYV	LFTFV	QARSGG	SVL	LVSGGGV	DTPDSSSSSRGIDRTPIVGLSQIPASPEGGTASTATTVQMFGGALYQALSPYVLFTFVQARSGGSVLLVSGGGV	1	0.10
AS (2020)_MLG146	Asia	DT	PDSSLSNRGI	DR	TPIVGLSQIPA	SPEDGTASTATTVQM	FGG	AL	YQ	AL	SPYV	LFIFV	HARSGG	SVL	LVSGDGV	DTPDSSLSNRGIDRTPIVGLSQIPASPEDGTASTATTVQMFGGALYQALSPYVLFIFVHARSGGSVLLVSGDGV	1	0.10
AS (2020)_MLG147	Asia	DT	PDSSSSSRGI	DR	TPIVGLSPIPA	SPEDGTASTATTVQM	FGG	AL	YQ	AL	SPYV	LFTFV	HARSGG	SVL	LVSGGGV	DTPDSSSSSRGIDRTPIVGLSPIPASPEDGTASTATTVQMFGGALYQALSPYVLFTFVHARSGGSVLLVSGGGV	1	0.10
AS (2020)_MLG148	Asia	DT	PDSSSSSRGI	DR	TPIVGLSQIPA	SPEDGTASTATTVQM	FGG	AL	YQ	AL	SPYV	LFIFV	HARSGG	SVL	LVSGGGV	DTPDSSSSSRGIDRTPIVGLSQIPASPEDGTASTATTVQMFGGALYQALSPYVLFIFVHARSGGSVLLVSGGGV	1	0.10
AS (2020)_MLG149	Asia	DT	PDSSSSSRGI	DR	TPIVGLSPIPA	SPEDGTASTATTVQM	FGG	AL	YQ	AP	SPYV	FFTFV	QARSGG	SVL	LVSGGGV	DTPDSSSSSRGIDRTPIVGLSPIPASPEDGTASTATTVQMFGGALYQAPSPYVFFTFVQARSGGSVLLVSGGGV	1	0.10
AS (2020)_MLG150	Asia	DT	PDSSLSSRGI	DR	TPIVGLSQIPA	SPEDGTASTATTVQM	FGG	AL	YQ	AP	SPYV	LFTFV	HARSGG	SVL	FVSGGGV	DTPDSSLSSRGIDRTPIVGLSQIPASPEDGTASTATTVQMFGGALYQAPSPYVLFTFVHARSGGSVLFVSGGGV	1	0.10
AS (2020)_MLG151	Asia	DT	PDSSLSSRGI	DR	TPIVGLSQIPA	SPEDGTASTATTVQM	FGG	AL	YQ	AL	SPYV	FFTFV	HARSGG	SVL	LVSGGGV	DTPDSSLSSRGIDRTPIVGLSQIPASPEDGTASTATTVQMFGGALYQALSPYVFFTFVHARSGGSVLLVSGGGV	1	0.10
AS (2020)_MLG152	Asia	DT	PDSSSSSKRI	DR	TPIVGLSPIPA	SPEDGTASTATTVQM	FGG	AL	YQ	AL	SPYV	LFIFV	HARSGG	SVL	FVSGGGV	DTPDSSSSSKRIDRTPIVGLSPIPASPEDGTASTATTVQMFGGALYQALSPYVLFIFVHARSGGSVLFVSGGGV	1	0.10
AS (2020)_MLG153	Asia	DT	PDSSSSNRGI	DR	TPIVGLSQIPA	SPEDGTASTATTVQM	FGG	AF	YQ	AP	SPYV	LFTFV	QARSGG	SVS	LVSGDGV	DTPDSSSSNRGIDRTPIVGLSQIPASPEDGTASTATTVQMFGGAFYQAPSPYVLFTFVQARSGGSVSLVSGDGV	1	0.10
AS (2020)_MLG154	Asia	DT	PDSSSSSRGI	DR	TPIVGLSQIPA	SPEDGTASTATTVQM	FGG	AL	YQ	AP	SPYV	LFTFV	QARSGV	SVS	LVSGDGV	DTPDSSSSSRGIDRTPIVGLSQIPASPEDGTASTATTVQMFGGALYQAPSPYVLFTFVQARSGVSVSLVSGDGV	1	0.10
AS (2020)_MLG155	Asia	DT	PDSSSSSRGI	DR	TPIVGLSQIPA	SPEDGTASTATTGQM	FGG	AL	YQ	AP	SPYV	LFTFV	QARSGG	SVS	LVSGDGV	DTPDSSSSSRGIDRTPIVGLSQIPASPEDGTASTATTGQMFGGALYQAPSPYVLFTFVQARSGGSVSLVSGDGV	1	0.10
AS (2020)_MLG156	Asia	DT	PDSSLSSRGI	DR	TPIVGLSPIPA	SPEDGTASTATTVQM	FGG	AL	YQ	AL	SPYV	LFIFV	QARSGG	SVL	FVSGGGV	DTPDSSLSSRGIDRTPIVGLSPIPASPEDGTASTATTVQMFGGALYQALSPYVLFIFVQARSGGSVLFVSGGGV	1	0.10
AS (2020)_MLG157	Asia	DT	PDSSSSSRGI	DR	TPIVGLSPIPA	SPEDGTASTATTVQM	FGG	AL	YQ	AP	SPYV	FFIFV	HARSGG	SVL	FVSGGGV	DTPDSSSSSRGIDRTPIVGLSPIPASPEDGTASTATTVQMFGGALYQAPSPYVFFIFVHARSGGSVLFVSGGGV	1	0.10
AS (2020)_MLG158	Asia	DT	PDSSLSNRGI	DR	TPIVGLSPIPA	SPEDGTASTATTVQM	FGG	AL	YQ	AP	SPYV	FFTFV	HARSGG	SVL	FVSGGGV	DTPDSSLSNRGIDRTPIVGLSPIPASPEDGTASTATTVQMFGGALYQAPSPYVFFTFVHARSGGSVLFVSGGGV	1	0.10
AS (2020)_MLG159	Asia	DT	PDSSSSSRGI	DR	TPIVGLSPIPA	SPEDGTASTATTVQM	FGG	AL	YQ	AL	SPYV	FFIFV	QARSGG	SVS	FVSGGGV	DTPDSSSSSRGIDRTPIVGLSPIPASPEDGTASTATTVQMFGGALYQALSPYVFFIFVQARSGGSVSFVSGGGV	1	0.10
AS (2020)_MLG160	Asia	DT	LDSSSSSRGI	DR	TPIVGLSQIPA	SPEDGTASKATTVQM	FGG	AL	YQ	VL	SPYV	LFTFV	QARSGG	SVL	LVSGDGV	DTLDSSSSSRGIDRTPIVGLSQIPASPEDGTASKATTVQMFGGALYQVLSPYVLFTFVQARSGGSVLLVSGDGV	1	0.10
AS (2020)_MLG161	Asia	DT	PDSSSSSRGI	DR	TPIVGLSQIPA	SPEDGTASTATTGQM	FGG	AF	YQ	VP	SPYV	LFTFV	QARSGG	SVL	LVSGDGV	DTPDSSSSSRGIDRTPIVGLSQIPASPEDGTASTATTGQMFGGAFYQVPSPYVLFTFVQARSGGSVLLVSGDGV	1	0.10
AS (2020)_MLG162	Asia	DT	PDSSSSSRGI	DC	TPIIGLSQIPA	SPEDGTASTATTGQI	FGG	AF	YQ	AP	SPYV	LFTFV	QARSGG	SVL	LVSGDGV	DTPDSSSSSRGIDCTPIIGLSQIPASPEDGTASTATTGQIFGGAFYQAPSPYVLFTFVQARSGGSVLLVSGDGV	1	0.10
AS (2020)_MLG163	Asia	DT	PDYSSSSRGI	DR	IPIVGLSQIPA	SPEDGTASTATTVQM	FGG	AL	YQ	AL	SPYV	LFTFV	HARSGG	SVL	LVSGGGV	DTPDYSSSSRGIDRIPIVGLSQIPASPEDGTASTATTVQMFGGALYQALSPYVLFTFVHARSGGSVLLVSGGGV	1	0.10
AS (2020)_MLG164	Asia	DT	PDSSSSSRGI	DR	TPIVGLSQIPA	SPEDGTASTATTVQI	FGG	AL	YQ	AL	SPYV	LFTFV	HARSGG	SVL	LVSGGGV	DTPDSSSSSRGIDRTPIVGLSQIPASPEDGTASTATTVQIFGGALYQALSPYVLFTFVHARSGGSVLLVSGGGV	1	0.10
AS (2020)_MLG165	Asia	DT	PDSSSSSRGI	DC	TPIIGLSQIPA	SPEDGTASTATTGQI	FGG	AL	YQ	AP	SPYV	LFTFV	QARSGG	SVL	LVSGDGV	DTPDSSSSSRGIDCTPIIGLSQIPASPEDGTASTATTGQIFGGALYQAPSPYVLFTFVQARSGGSVLLVSGDGV	1	0.10
AS (2020)_MLG166	Asia	DT	PDSSSSSKRI	DR	TPIVGLSQIPA	SPEDGTASTATTVQM	FGG	AL	YQ	AP	SPYV	LFTFV	QARSGG	SVL	LVSGDGV	DTPDSSSSSKRIDRTPIVGLSQIPASPEDGTASTATTVQMFGGALYQAPSPYVLFTFVQARSGGSVLLVSGDGV	1	0.10
AS (2020)_MLG167	Asia	DT	PDSSSSSRGI	DR	TPIVGLSQIPA	SPEDGTASTATTGQM	FGG	AL	YQ	AL	SPYL	LFTFV	QARSGG	SVL	LVSGGGV	DTPDSSSSSRGIDRTPIVGLSQIPASPEDGTASTATTGQMFGGALYQALSPYLLFTFVQARSGGSVLLVSGGGV	1	0.10
AS (2020)_MLG168	Asia	DT	PDSSSSSRGI	DR	TPIVGLSQISA	SPEDGTASTATTVQM	FGG	AF	YQ	AP	SPYV	LFTFV	QARSGV	SVL	LVSGDGV	DTPDSSSSSRGIDRTPIVGLSQISASPEDGTASTATTVQMFGGAFYQAPSPYVLFTFVQARSGVSVLLVSGDGV	1	0.10
AS (2020)_MLG169	Asia	DT	LDSSSSSKRI	DR	TPIVGLFQIPA	SPEDGTASTATTVQM	FGG	AL	YQ	VL	SPYV	LFTFV	QARSGG	SVL	LVSGGGV	DTLDSSSSSKRIDRTPIVGLFQIPASPEDGTASTATTVQMFGGALYQVLSPYVLFTFVQARSGGSVLLVSGGGV	1	0.10
AS (2020)_MLG170	Asia	DT	LDSSSSSKRI	DR	TPIVGLFQIPA	SPEDGTARKATTVQM	FGG	AF	YH	VP	SPYV	LFTFV	QARSGG	SVL	LVSGDGV	DTLDSSSSSKRIDRTPIVGLFQIPASPEDGTARKATTVQMFGGAFYHVPSPYVLFTFVQARSGGSVLLVSGDGV	1	0.10
AS (2020)_MLG171	Asia	DT	LDSSSSSRGI	DR	TPIVGLSQIPA	SPEDGTARKATTVQM	FGG	AF	YQ	VP	SPYV	LFTFV	QSRSGG	SVL	LVSGDGV	DTLDSSSSSRGIDRTPIVGLSQIPASPEDGTARKATTVQMFGGAFYQVPSPYVLFTFVQSRSGGSVLLVSGDGV	1	0.10
AS (2020)_MLG172	Asia	DT	PDSSSSSRGI	DR	TPIVGLSQIPA	SPEDGTASTATTGQM	FGG	AF	YQ	AL	SPYV	LFTFV	QARSGG	SVL	LVSGGGV	DTPDSSSSSRGIDRTPIVGLSQIPASPEDGTASTATTGQMFGGAFYQALSPYVLFTFVQARSGGSVLLVSGGGV	1	0.10
AS (2020)_MLG173	Asia	DT	PDSSSSSKRI	DR	TPIVGLSQIPA	SPEDGIASTATTVQM	FSG	AL	YQ	AL	SPYV	LFTFV	QARSGG	SVL	LVSGGGV	DTPDSSSSSKRIDRTPIVGLSQIPASPEDGIASTATTVQMFSGALYQALSPYVLFTFVQARSGGSVLLVSGGGV	1	0.10
AS (2020)_MLG174	Asia	DT	PDSSSSSRGI	DR	TPIVGLSQIPA	SPEEGTASTATTVQM	FGG	AL	YQ	AL	SPYV	LFTFV	QARSGG	SVL	LVSGGGV	DTPDSSSSSRGIDRTPIVGLSQIPASPEEGTASTATTVQMFGGALYQALSPYVLFTFVQARSGGSVLLVSGGGV	1	0.10
AS (2020)_MLG175	Asia	DT	PDSSSSSRGI	DR	TPIVGLSQIPA	SPEDGTASTATTVQM	FGG	AL	YQ	AL	SPYV	LFTFV	HARSGG	SVL	LVSGDGV	DTPDSSSSSRGIDRTPIVGLSQIPASPEDGTASTATTVQMFGGALYQALSPYVLFTFVHARSGGSVLLVSGDGV	1	0.10
AS (2020)_MLG176	Asia	DT	PDSSSSSKRI	DR	TPIVGLSQIPA	SPEDGTASTATTVQM	FGG	AL	YQ	AL	SPYV	LFTFV	QARSGV	SVL	LVSGGGV	DTPDSSSSSKRIDRTPIVGLSQIPASPEDGTASTATTVQMFGGALYQALSPYVLFTFVQARSGVSVLLVSGGGV	1	0.10
AS (2020)_MLG177	Asia	DT	PDSSSSNRGI	DR	TPIVGLSQIPA	SPEDGTASTATTGQM	FGG	AF	YQ	AP	SPYV	LFTFV	QARSGG	SVS	LVSGDGV	DTPDSSSSNRGIDRTPIVGLSQIPASPEDGTASTATTGQMFGGAFYQAPSPYVLFTFVQARSGGSVSLVSGDGV	1	0.10
AS (2020)_MLG178	Asia	DT	PDSSSSNRGI	DR	TPIVGLSQIPA	SPEDGTASTATTGQM	FGG	AL	YQ	AP	SPYV	LFTFV	QARSGG	SVS	LVSGDGV	DTPDSSSSNRGIDRTPIVGLSQIPASPEDGTASTATTGQMFGGALYQAPSPYVLFTFVQARSGGSVSLVSGDGV	1	0.10
AS (2020)_MLG179	Asia	DT	PDSSSSSRGI	DR	TPIVGLSQIPA	SPEDGTASTATTVQM	FGG	AL	YQ	AL	LPYV	LFTFV	HARSGG	SVL	LVSGGGV	DTPDSSSSSRGIDRTPIVGLSQIPASPEDGTASTATTVQMFGGALYQALLPYVLFTFVHARSGGSVLLVSGGGV	1	0.10
AS (2020)_MLG180	Asia	DT	LDSSSSSRGI	DR	TPIVGLSQIPA	SPEDGTASTATTVQM	FGG	AL	YQ	AL	SPYV	LFTFV	HARSGG	SVL	LVSGGGV	DTLDSSSSSRGIDRTPIVGLSQIPASPEDGTASTATTVQMFGGALYQALSPYVLFTFVHARSGGSVLLVSGGGV	1	0.10
AS (2020)_MLG181	Asia	DT	PDSSSSSRGI	DC	TPIIGLSQIPP	SPEDGTASTATTVQM	FGG	AF	YQ	AP	SPYV	FFTFV	QARSGG	SVL	LVSGDGV	DTPDSSSSSRGIDCTPIIGLSQIPPSPEDGTASTATTVQMFGGAFYQAPSPYVFFTFVQARSGGSVLLVSGDGV	1	0.10
AS (2020)_MLG182	Asia	DT	LDSSSSSRGI	DR	TPIVGLSQIPA	SSEDGTASKATTGQM	FGG	AF	YQ	VP	SPYV	LFTFV	QARSGG	SVL	LVSGDGV	DTLDSSSSSRGIDRTPIVGLSQIPASSEDGTASKATTGQMFGGAFYQVPSPYVLFTFVQARSGGSVLLVSGDGV	1	0.10
AS (2020)_MLG183	Asia	DT	PDSSSSSRGI	DR	TPIVGLSQVPA	SPEDGTASTATTVQM	FGG	AL	YQ	AL	SPYV	LFTFV	HSRSGG	SVL	LVSGGGV	DTPDSSSSSRGIDRTPIVGLSQVPASPEDGTASTATTVQMFGGALYQALSPYVLFTFVHSRSGGSVLLVSGGGV	1	0.10
AS (2020)_MLG184	Asia	DT	PDSSSSSRGI	DR	TPIVGLSQVPA	SPEDGTASTATTVQM	FGG	AL	YQ	AL	SPYV	LFTFV	QARSGG	SVL	LVSGGGV	DTPDSSSSSRGIDRTPIVGLSQVPASPEDGTASTATTVQMFGGALYQALSPYVLFTFVQARSGGSVLLVSGGGV	1	0.10
AS (2020)_MLG185	Asia	DT	PDSSSSSRGI	DR	TPIVGLSQVPA	SPEDGTASTATTVQM	FGG	AL	YQ	AL	SPYV	LFTFV	HARSGG	SVL	LVIGGGV	DTPDSSSSSRGIDRTPIVGLSQVPASPEDGTASTATTVQMFGGALYQALSPYVLFTFVHARSGGSVLLVIGGGV	1	0.10
AS (2021)_MLG186	Asia	DT	PDSSSSSKRI	DR	TPIVGLSQIDA	PEDGTASTATTVQM	FGG	AL	YQ	AL	SPYV	LFTFV	QARSGG	SVL	LVSGGGV	DTPDSSSSSKRIDRTPIVGLSQIDAPEDGTASTATTVQMFGGALYQALSPYVLFTFVQARSGGSVLLVSGGGV	676	32.18
AS (2021)_MLG187	Asia	DT	PDSSSSSRGI	DR	TPIVGLSQIDA	PEDGTASTATTVQM	FGG	AL	YQ	AL	SPYV	LFTFV	HARSGG	SVL	LVSGGGV	DTPDSSSSSRGIDRTPIVGLSQIDAPEDGTASTATTVQMFGGALYQALSPYVLFTFVHARSGGSVLLVSGGGV	465	22.13

AS (2021)_MLG188	Asia	DT	PDSSSSSRGI	DR	IPIVGLSQIDA	PEDGTASTATTVQM	FGG	AL	YQ	AL	SPYV	LFTFV	HARSGG	SVL	LVSGGGV	DTPDSSSSSRGIDRIPIVGLSQIDAPEDGTASTATTVQMFGGALYQALSPYVLFTFVHARSGGSVLLVSGGGV	237	11.28
AS (2021)_MLG189	Asia	DT	PDSSSSSKRI	DR	TPIVGLSQIDA	PEDGTASTATTVQM	FGG	AL	YQ	AL	SPYV	LFTFV	QARSGG	SVL	LVSGGGF	DTPDSSSSSKRIDRTPIVGLSQIDAPEDGTASTATTVQMFGGALYQALSPYVLFTFVQARSGGSVLLVSGGGF	140	6.66
AS (2021)_MLG190	Asia	DT	PDSSSSSKRI	DR	TPIVGLSQIDA	PEDGTASTATTVQM	FGG	AF	YQ	AL	SPYV	LFTFV	QARSGG	SVL	LVSGGGV	DTPDSSSSSKRIDRTPIVGLSQIDAPEDGTASTATTVQMFGGAFYQALSPYVLFTFVQARSGGSVLLVSGGGV	98	4.66
AS (2021)_MLG191	Asia	DT	PDSSLSSRGI	DR	TPIVGLSQIDA	PEDGTASIATTVQM	FGG	AL	YQ	AL	SPYV	LFTFV	QARSGG	SVL	LVSGGGV	DTPDSSLSSRGIDRTPIVGLSQIDAPEDGTASIATTVQMFGGALYQALSPYVLFTFVQARSGGSVLLVSGGGV	60	2.86
AS (2021)_MLG192	Asia	DT	PDSSLSSRGI	DR	TPIVGLSQIDA	PEDGTASTATTVQM	FGG	AL	YQ	AL	SPYV	LFTFV	HARSGG	SVL	LVSGGGV	DTPDSSLSSRGIDRTPIVGLSQIDAPEDGTASTATTVQMFGGALYQALSPYVLFTFVHARSGGSVLLVSGGGV	59	2.81
AS (2021)_MLG193	Asia	DT	PDSSSSSMGI	DR	TPIVGLSQIDA	PEDGTASTATTVQM	FGG	AL	YQ	AL	SPYV	LFTFV	QARSGG	SVL	LVSGGGV	DTPDSSSSSMGIDRTPIVGLSQIDAPEDGTASTATTVQMFGGALYQALSPYVLFTFVQARSGGSVLLVSGGGV	30	1.43
AS (2021)_MLG194	Asia	DT	PDSSSSSKRI	DR	TPIVGLSQIDA	PEDGTASTATTVQM	FGG	AF	YQ	AL	SPYV	LFTFV	QARSGG	SLL	LVSGGGV	DTPDSSSSSKRIDRTPIVGLSQIDAPEDGTASTATTVQMFGGAFYQALSPYVLFTFVQARSGGSLLLVSGGGV	25	1.19
AS (2021)_MLG195	Asia	DT	PDSSSSSKRI	DR	TPIVGLSQIDA	PEDGTASTATTVQM	FGG	AL	YQ	AL	SSYV	LFTFV	QARSGG	SVL	LVSGGGV	DTPDSSSSSKRIDRTPIVGLSQIDAPEDGTASTATTVQMFGGALYQALSSYVLFTFVQARSGGSVLLVSGGGV	18	0.86
AS (2021)_MLG196	Asia	DT	PDSSSSSRGI	DR	TPIVGLSQIDA	PEDGTASTATTVQM	FGG	AF	YQ	AL	SPYV	LFTFV	HARSGG	SVL	LVSGGGV	DTPDSSSSSRGIDRTPIVGLSQIDAPEDGTASTATTVQMFGGAFYQALSPYVLFTFVHARSGGSVLLVSGGGV	13	0.62
AS (2021)_MLG197	Asia	DT	PDSSLSSRGI	DR	TPIVGLSQIDA	PEDGTASTATTVQM	FGG	AF	YQ	AL	SPYV	LFTFV	HARSGG	SVL	LVSGGGV	DTPDSSLSSRGIDRTPIVGLSQIDAPEDGTASTATTVQMFGGAFYQALSPYVLFTFVHARSGGSVLLVSGGGV	13	0.62
AS (2021)_MLG198	Asia	DT	PDSSLSSRGI	DR	TPIVGLSQIDA	PEDGTASIATTVQM	FGG	AL	YQ	AL	SPYV	FFTFV	QARSGG	SVL	LVSGGGV	DTPDSSLSSRGIDRTPIVGLSQIDAPEDGTASIATTVQMFGGALYQALSPYVFFTFVQARSGGSVLLVSGGGV	13	0.62
AS (2021)_MLG199	Asia	DT	PDSSLSSRGI	DR	TPIVGLSQIDA	PEDGTASIATTVQM	FGG	AF	YQ	AL	SPYV	LFTFV	QARSGG	SVL	LVSGGGV	DTPDSSLSSRGIDRTPIVGLSQIDAPEDGTASIATTVQMFGGAFYQALSPYVLFTFVQARSGGSVLLVSGGGV	13	0.62
AS (2021)_MLG200	Asia	DT	PDSSSSSRGI	DR	TPIVGLSQIDA	PEDGTASTATTVQM	FGG	AL	YQ	AL	SPYV	LFTFV	QARSGG	SVL	LVSGGGV	DTPDSSSSSRGIDRTPIVGLSQIDAPEDGTASTATTVQMFGGALYQALSPYVLFTFVQARSGGSVLLVSGGGV	12	0.57
AS (2021)_MLG201	Asia	DT	PDSLSSSKRI	DR	TPIVGLSQIDA	PEDGTASTATTVQM	FGG	AL	YQ	AL	SPYV	LFTFV	QARSGG	SVL	LVSGGGV	DTPDSLSSSKRIDRTPIVGLSQIDAPEDGTASTATTVQMFGGALYQALSPYVLFTFVQARSGGSVLLVSGGGV	12	0.57
AS (2021)_MLG202	Asia	DT	LDSSSSSRGI	DR	TPIVGLSQIDA	PEDGTASKATTVQM	FGG	AF	YQ	VP	SPYV	LFTFV	QARSGG	SVL	LVSGGGV	DTLDSSSSSRGIDRTPIVGLSQIDAPEDGTASKATTVQMFGGAFYQVPSPYVLFTFVQARSGGSVLLVSGGGV	11	0.52
AS (2021)_MLG203	Asia	DT	LDSSSSSKRI	DR	TPIVGLSQIDA	PEDGTASTATTVQM	FGG	AL	YQ	AL	SPYV	LFTFV	QARSGG	SVL	LVSGGGF	DTLDSSSSSKRIDRTPIVGLSQIDAPEDGTASTATTVQMFGGALYQALSPYVLFTFVQARSGGSVLLVSGGGF	11	0.52
AS (2021)_MLG204	Asia	DT	PDSSSSSKRI	DR	TPIVGLSQIDA	PEDGTASTATTVHM	FGG	AL	YQ	AL	SPYV	LFTFV	QARSGG	SVL	LVSGGGV	DTPDSSSSSKRIDRTPIVGLSQIDAPEDGTASTATTVHMFGGALYQALSPYVLFTFVQARSGGSVLLVSGGGV	9	0.43
AS (2021)_MLG205	Asia	DT	PDSSSSSKRI	DR	TPIVGLSQIDA	PEDGTASTATTVQM	FGG	AL	YQ	AL	SPYV	LFTFV	QARSGG	SVL	LVNGGGV	DTPDSSSSSKRIDRTPIVGLSQIDAPEDGTASTATTVQMFGGALYQALSPYVLFTFVQARSGGSVLLVNGGGV	9	0.43
AS (2021)_MLG206	Asia	DT	PDSSSSSRGI	DR	TPIVGLSQIDT	PEDGTASTATTVQM	FGG	AL	YQ	AL	SPYV	LFTFV	HARSGG	SLL	LVSGGGV	DTPDSSSSSRGIDRTPIVGLSQIDTPEDGTASTATTVQMFGGALYQALSPYVLFTFVHARSGGSLLLVSGGGV	8	0.38
AS (2021)_MLG207	Asia	DT	SDSSSSSRGI	DR	IPIVGLSQIDA	PEDGTASTATTVQM	FGG	AL	YQ	AL	SPYV	LFTFV	HARSGG	SVL	LVSGGGV	DTSDSSSSSRGIDRIPIVGLSQIDAPEDGTASTATTVQMFGGALYQALSPYVLFTFVHARSGGSVLLVSGGGV	8	0.38
AS (2021)_MLG208	Asia	DT	PDSSSSSKRI	DR	TPIVGLSQIDA	PEDGTASTATTVQM	FGG	AL	YQ	AL	SPYV	LFTFV	HARSGG	SVL	LVSGGGV	DTPDSSSSSKRIDRTPIVGLSQIDAPEDGTASTATTVQMFGGALYQALSPYVLFTFVHARSGGSVLLVSGGGV	6	0.29
AS (2021)_MLG209	Asia	DT	PDSSSSSKRI	DR	TPIVGLSQIDA	LEDGTASTATTVQM	FGG	AL	YQ	AL	SPYV	LFTFV	QARSGG	SVL	LVSGGGV	DTPDSSSSSKRIDRTPIVGLSQIDALEDGTASTATTVQMFGGALYQALSPYVLFTFVQARSGGSVLLVSGGGV	6	0.29
AS (2021)_MLG210	Asia	DT	PDSSSSNKGI	DR	TPIVGLSQIDA	PEDGTASTATTVQM	FGG	AL	YQ	AP	SPYV	LFTFV	QARSGG	SVS	LVSGDGV	DTPDSSSSNKGIDRTPIVGLSQIDAPEDGTASTATTVQMFGGALYQAPSPYVLFTFVQARSGGSVSLVSGDGV	6	0.29
AS (2021)_MLG211	Asia	DT	PDSSSSSKRI	DR	TPIVGLSQIDA	PEDGTASTATTVQM	FGG	AL	YQ	AL	SPYV	LFTFV	QARSGG	SLL	LVSGGGV	DTPDSSSSSKRIDRTPIVGLSQIDAPEDGTASTATTVQMFGGALYQALSPYVLFTFVQARSGGSLLLVSGGGV	5	0.24
AS (2021)_MLG212	Asia	DT	PDSSSSSKRI	DR	TPIVGLSQIDA	PEDGTDSTATTVQM	FGG	AL	YQ	AL	SPYV	LFTFV	QARSGG	SVL	LVSGGGV	DTPDSSSSSKRIDRTPIVGLSQIDAPEDGTDSTATTVQMFGGALYQALSPYVLFTFVQARSGGSVLLVSGGGV	5	0.24
AS (2021)_MLG213	Asia	DT	PDSSSSSRGI	DR	TPIVGLSQIDA	PEDGTATTATTVQM	FGG	AL	YQ	AL	SPYV	LFTFV	HARSGG	SVL	LVSGGGV	DTPDSSSSSRGIDRTPIVGLSQIDAPEDGTATTATTVQMFGGALYQALSPYVLFTFVHARSGGSVLLVSGGGV	4	0.19
AS (2021)_MLG214	Asia	DT	PDSSLSSRGI	DC	TPIVGFSQIDA	PEDGTASIATTVQM	FGG	AL	YQ	AL	SPYV	LFTFV	QARSGG	SVL	LVSGGGV	DTPDSSLSSRGIDCTPIVGFSQIDAPEDGTASIATTVQMFGGALYQALSPYVLFTFVQARSGGSVLLVSGGGV	4	0.19
AS (2021)_MLG215	Asia	DT	PDSSSSNRGI	DR	TPIVGLSQIDA	PEDGTASTATTVQM	FGG	AL	YQ	AP	SPYV	LFTFV	QARSGG	SVS	LVSGDGV	DTPDSSSSNRGIDRTPIVGLSQIDAPEDGTASTATTVQMFGGALYQAPSPYVLFTFVQARSGGSVSLVSGDGV	4	0.19
AS (2021)_MLG216	Asia	DT	PDSSSSSRGI	DR	TPIVGLSQIDA	PEDGTASIATTVQM	FGG	AF	YQ	AL	SPYV	LFTFV	QARSGG	SVL	LVSGGGV	DTPDSSSSSRGIDRTPIVGLSQIDAPEDGTASIATTVQMFGGAFYQALSPYVLFTFVQARSGGSVLLVSGGGV	3	0.14
AS (2021)_MLG217	Asia	DT	PDSSSSSRGI	DR	TPIVGLSQIDA	PEDGTASTATTVQM	FGG	AL	YQ	AL	SSYV	LFTFV	HARSGG	SVL	LVSGGGV	DTPDSSSSSRGIDRTPIVGLSQIDAPEDGTASTATTVQMFGGALYQALSSYVLFTFVHARSGGSVLLVSGGGV	3	0.14
AS (2021)_MLG218	Asia	DT	PDSSSSSRGI	DR	TPIVGLSQIDA	PEDGTASTATTVQM	FGG	AL	YQ	AF	SPYV	LFTFV	QARSGG	SVL	LVSGGGV	DTPDSSSSSRGIDRTPIVGLSQIDAPEDGTASTATTVQMFGGALYQAFSPYVLFTFVQARSGGSVLLVSGGGV	3	0.14
AS (2021)_MLG219	Asia	DT	PDSSSSSRGI	DR	TPIVGLSQIDA	LEDGTASTATTVQM	FGG	AL	YQ	AL	SPYV	LFTFV	HARSGG	SVL	LVSGGGV	DTPDSSSSSRGIDRTPIVGLSQIDALEDGTASTATTVQMFGGALYQALSPYVLFTFVHARSGGSVLLVSGGGV	3	0.14
AS (2021)_MLG220	Asia	DT	PDSSSSNRGI	DR	TPIVGLSQIDA	PEDGTASTATTVQM	FGG	AL	YQ	AP	SPYV	LFTFV	QSRSGG	SVS	LVSGNGV	DTPDSSSSNRGIDRTPIVGLSQIDAPEDGTASTATTVQMFGGALYQAPSPYVLFTFVQSRSGGSVSLVSGNGV	3	0.14
AS (2021)_MLG221	Asia	DT	PDSSSSSKRI	DR	TPIVGLSQIDA	SEDGTASTATTVQM	FGG	AL	YQ	AL	SPYV	LFTFV	QARSGG	SVL	LVSGGGV	DTPDSSSSSKRIDRTPIVGLSQIDASEDGTASTATTVQMFGGALYQALSPYVLFTFVQARSGGSVLLVSGGGV	3	0.14
AS (2021)_MLG222	Asia	DT	PDSSSSSKRI	DR	TPIVGLSQIDA	PEDGTASTATTVQM	FGG	AL	YQ	AP	SPYV	LFTFV	QARSGG	SVL	LVSGGGV	DTPDSSSSSKRIDRTPIVGLSQIDAPEDGTASTATTVQMFGGALYQAPSPYVLFTFVQARSGGSVLLVSGGGV	3	0.14
AS (2021)_MLG223	Asia	DT	SDSSSSSKRI	DR	TPIVGLSQIDA	PEDGTASTATTVQM	FGG	AL	YQ	AL	SPYV	LFTFV	QARSGG	SVL	LVSGGGF	DTSDSSSSSKRIDRTPIVGLSQIDAPEDGTASTATTVQMFGGALYQALSPYVLFTFVQARSGGSVLLVSGGGF	3	0.14
AS (2021)_MLG224	Asia	DT	PDSSSSSKRI	DR	TPIVGLSQIDA	PEDGTASTATTVQM	FGG	AF	YQ	AL	SPYV	LFTFV	QARSGG	SVL	LVSGGGF	DTPDSSSSSKRIDRTPIVGLSQIDAPEDGTASTATTVQMFGGAFYQALSPYVLFTFVQARSGGSVLLVSGGGF	3	0.14
AS (2021)_MLG225	Asia	DT	PDSSLSSRGI	DR	TPIVGLSQIDA	PEDGTASTATTVQM	FGG	AL	YQ	AL	SPYV	LFTFV	QARSGG	SVL	LVSGGGV	DTPDSSLSSRGIDRTPIVGLSQIDAPEDGTASTATTVQMFGGALYQALSPYVLFTFVQARSGGSVLLVSGGGV	2	0.10
AS (2021)_MLG226	Asia	DT	PDSSSSSRGI	DR	TPIVGLSQIDA	SEDGTASTATTVQM	FGG	AL	YQ	AL	SPYV	LFTFV	HARSGG	SVL	LVSGGGV	DTPDSSSSSRGIDRTPIVGLSQIDASEDGTASTATTVQMFGGALYQALSPYVLFTFVHARSGGSVLLVSGGGV	2	0.10
AS (2021)_MLG227	Asia	DT	PYSSSSSRGI	DR	TPIVGLSQIDA	PEDGTASTATTVQM	FGG	AL	YQ	AL	SPYV	LFTFV	HARSGG	SVL	LVSGGGV	DTPYSSSSSRGIDRTPIVGLSQIDAPEDGTASTATTVQMFGGALYQALSPYVLFTFVHARSGGSVLLVSGGGV	2	0.10
AS (2021)_MLG228	Asia	DT	PDSSSSSRGI	DR	TPIVGLSQIDA	PEDGTASTATTVQM	FGG	AL	YQ	AL	SPYV	LFTFV	HARSGG	SVL	LVSGGGF	DTPDSSSSSRGIDRTPIVGLSQIDAPEDGTASTATTVQMFGGALYQALSPYVLFTFVHARSGGSVLLVSGGGF	2	0.10
AS (2021)_MLG229	Asia	DT	PDSSSSGRGI	DR	TPIVGLSQIDA	PEDGTASIATTVQM	FGG	AL	YQ	AL	SPYV	LFTFV	QARSGG	SVL	LVSGGGV	DTPDSSSSGRGIDRTPIVGLSQIDAPEDGTASIATTVQMFGGALYQALSPYVLFTFVQARSGGSVLLVSGGGV	2	0.10
AS (2021)_MLG230	Asia	DT	PDSSSSSRGI	DR	TPIVGLSQIDA	PEDGTASTATTVQM	FGG	AL	YQ	AL	SPYV	LFTFV	HARSGG	SLL	LVSGGGV	DTPDSSSSSRGIDRTPIVGLSQIDAPEDGTASTATTVQMFGGALYQALSPYVLFTFVHARSGGSLLLVSGGGV	2	0.10
AS (2021)_MLG231	Asia	DT	PDSSLSSRGI	DR	TPIVGLSQIDA	PEDGTASIATTVQM	FGG	AL	YQ	AL	SPYV	LFTFV	QARSVG	SVL	LVSGGGV	DTPDSSLSSRGIDRTPIVGLSQIDAPEDGTASIATTVQMFGGALYQALSPYVLFTFVQARSVGSVLLVSGGGV	2	0.10
AS (2021)_MLG232	Asia	DT	PDSSSSSRGI	DR	TPIVGLSQIDA	PEDGTASTVTTVQM	FGG	AL	YQ	AL	SPYV	LFTFV	HARSGG	SVL	LVSGGGV	DTPDSSSSSRGIDRTPIVGLSQIDAPEDGTASTVTTVQMFGGALYQALSPYVLFTFVHARSGGSVLLVSGGGV	2	0.10
AS (2021)_MLG233	Asia	DT	PDSSLSSRGI	DR	TPIVGLSQIDA	PEDGTASIATTVQM	FGG	AL	YQ	AL	SPYV	LFTFV	QASSGG	SVL	LVSGGGV	DTPDSSLSSRGIDRTPIVGLSQIDAPEDGTASIATTVQMFGGALYQALSPYVLFTFVQASSGGSVLLVSGGGV	2	0.10
AS (2021)_MLG234	Asia	DT	PDSSSSSRGI	DR	IPIVGLSQIDA	PEDGTASTATTVQM	FGG	AL	YQ	AL	SPYV	LFTFV	HARSGG	LVL	LVSGGGV	DTPDSSSSSRGIDRIPIVGLSQIDAPEDGTASTATTVQMFGGALYQALSPYVLFTFVHARSGGLVLLVSGGGV	2	0.10
AS (2021)_MLG235	Asia	DT	PDSSLSSRGI	DR	TPIVGLSQIDA	PEDGTASTATTVQM	FGG	AL	YQ	AL	SPYV	LFTFV	HARSGG	SVL	FVSGGGV	DTPDSSLSSRGIDRTPIVGLSQIDAPEDGTASTATTVQMFGGALYQALSPYVLFTFVHARSGGSVLFVSGGGV	2	0.10
AS (2021)_MLG236	Asia	DT	PDSSSSSKRI	DR	TPIVGLSQIDA	LEDGTASTATTVQM	FGG	AL	YQ	AL	SPYV	LFTFV	QARSGG	SVL	LVSGGGF	DTPDSSSSSKRIDRTPIVGLSQIDALEDGTASTATTVQMFGGALYQALSPYVLFTFVQARSGGSVLLVSGGGF	2	0.10
AS (2021)_MLG237	Asia	DT	PDSSSSSKRI	DR	TPIVGLSQIDA	PEDGTASTATTVQM	FGG	AL	YQ	AL	SSYV	LFTFV	QARSGG	SVL	LVSGGGF	DTPDSSSSSKRIDRTPIVGLSQIDAPEDGTASTATTVQMFGGALYQALSSYVLFTFVQARSGGSVLLVSGGGF	2	0.10
AS (2021)_MLG238	Asia	DT	PDSSSSSKRI	DR	TPIVGLSQIDA	PEDGTASTATTVQM	FGS	AL	YQ	AL	SPYV	LFTFV	QARSGG	SVL	LVSGGGV	DTPDSSSSSKRIDRTPIVGLSQIDAPEDGTASTATTVQMFGSALYQALSPYVLFTFVQARSGGSVLLVSGGGV	2	0.10
AS (2021)_MLG239	Asia	DT	PDSSSSSKRI	DR	TPIVGLSQIDA	PEDGTASTATTVQM	FGG	AF	YQ	AL	SPYV	LFTFV	HARSGG	SLL	LVSGGGV	DTPDSSSSSKRIDRTPIVGLSQIDAPEDGTASTATTVQMFGGAFYQALSPYVLFTFVHARSGGSLLLVSGGGV	2	0.10
AS (2021)_MLG240	Asia	DT	PDSSSSSKRI	DR	TPIVGLSQIDA	PEDGTASTATTVQM	FGG	AL	YQ	AL	SPYV	LFTFV	QARSGG	SVL	FVSGGGV	DTPDSSSSSKRIDRTPIVGLSQIDAPEDGTASTATTVQMFGGALYQALSPYVLFTFVQARSGGSVLFVSGGGV	2	0.10
AS (2021)_MLG241	Asia	DT	PDSSSSSKRI	DR	TPFVGLSQIDA	PEDGTASTATTVQM	FGG	AL	YQ	AL	SPYV	LFTFV	QARSGG	SVL	LVSGGGV	DTPDSSSSSKRIDRTPFVGLSQIDAPEDGTASTATTVQMFGGALYQALSPYVLFTFVQARSGGSVLLVSGGGV	2	0.10
AS (2021)_MLG242	Asia	DT	PDSSSSSMGI	DR	TPIVGLSQIDA	PEDGTASTATTGQM	FGG	AL	YQ	AL	SPYV	LFTFV	QARSGG	SVL	LVSGGGV	DTPDSSSSSMGIDRTPIVGLSQIDAPEDGTASTATTGQMFGGALYQALSPYVLFTFVQARSGGSVLLVSGGGV	2	0.10
AS (2021)_MLG243	Asia	DT	PDFSSSSRGI	DR	TPIVGLSQIDA	PEDGTASTATTVQM	FGG	AL	YQ	AL	SPYV	LFTFV	QARSGG	SVL	LVSGGGV	DTPDFSSSSRGIDRTPIVGLSQIDAPEDGTASTATTVQMFGGALYQALSPYVLFTFVQARSGGSVLLVSGGGV	1	0.05
AS (2021)_MLG244	Asia	DT	PDSSSSSRGI	DR	IPIVGLSQIDA	PEDGTASTATTVQM	FGG	AL	YQ	AL	SPYV	LFTFV	YARSGG	SVL	LVSGGGV	DTPDSSSSSRGIDRIPIVGLSQIDAPEDGTASTATTVQMFGGALYQALSPYVLFTFVYARSGGSVLLVSGGGV	1	0.05
AS (2021)_MLG245	Asia	DT	PNSSSSSRGI	DR	TPIVGLSQIDA	PEDGTASTATTVQM	FGG	AL	YQ	AL	SPYV	LFTFV	HARSGG	SVL	LVSGGGV	DTPNSSSSSRGIDRTPIVGLSQIDAPEDGTASTATTVQMFGGALYQALSPYVLFTFVHARSGGSVLLVSGGGV	1	0.05
AS (2021)_MLG246	Asia	DT	PYSSSSSRGI	DR	TPIVGLSQIDT	SEDGTASTATTVQM	FGG	AL	YQ	AL	SPYV	LFTFV	HARSGG	SLL	LVSGGGV	DTPYSSSSSRGIDRTPIVGLSQIDTSEDGTASTATTVQMFGGALYQALSPYVLFTFVHARSGGSLLLVSGGGV	1	0.05
AS (2021)_MLG247	Asia	DT	PDSSSSNRGI	DR	TPIVGLSQIDA	PEDGTASTATTVQM	FGG	TL	YQ	AP	SPYV	LFTFV	QTRSGG	SVS	LVSGDGV	DTPDSSSSNRGIDRTPIVGLSQIDAPEDGTASTATTVQMFGGTLYQAPSPYVLFTFVQTRSGGSVSLVSGDGV	1	0.05
AS (2021)_MLG248	Asia	DT	PDSSLSSRGI	DR	TPIVGLSQIDA	PEDGTASTATTVQM	FGG	AL	YQ	AL	SPYV	LFTFV	HARSGG	SVL	LVSGGGF	DTPDSSLSSRGIDRTPIVGLSQIDAPEDGTASTATTVQMFGGALYQALSPYVLFTFVHARSGGSVLLVSGGGF	1	0.05
AS (2021)_MLG249	Asia	DT	PDSSSSSRGI	DR	TPIVGLSQIDA	PEDGTASTATTVQM	FGG	AL	YQ	AL	SPYV	LFTFV	HARSGG	SVL	LVSGGVV	DTPDSSSSSRGIDRTPIVGLSQIDAPEDGTASTATTVQMFGGALYQALSPYVLFTFVHARSGGSVLLVSGGVV	1	0.05
AS (2021)_MLG250	Asia	DT	PDSSSSSRGI	DR	TPIVGLSQIDA	PEDGTAGTATTVQM	FGG	AL	YQ	AL	SPYV	LFTFV	HARSGG	SVL	LVSGGGV	DTPDSSSSSRGIDRTPIVGLSQIDAPEDGTAGTATTVQMFGGALYQALSPYVLFTFVHARSGGSVLLVSGGGV	1	0.05
AS (2021)_MLG251	Asia	DT	PDSSSSSRGI	DR	TPIVGLSQIDA	PEDGTASTATTVQM	FGG	AF	YQ	AL	SPYV	LFTFV	QARSGG	SVL	LVSGGGV	DTPDSSSSSRGIDRTPIVGLSQIDAPEDGTASTATTVQMFGGAFYQALSPYVLFTFVQARSGGSVLLVSGGGV	1	0.05

AS (2021)_MLG252	Asia	DT	PDSSSSSRGI	DR	TPIVGLSQIDA	PEDGTASTATTVQM	FGG	AL	YQ	AL	SPYL	LFTFV	HARSGG	SVL	LVSGGGV	DTPDSSSSSRGIDRTPIVGLSQIDAPEDGTASTATTVQMFGGALYQALSPYLLFTFVHARSGGSVLLVSGGGV	1	0.05
AS (2021)_MLG253	Asia	DT	PDSSSSSRGI	DR	TPIVGLSQIDA	PEDGTASTATTVQM	FGG	AL	YQ	AL	SPYV	LFTFV	NARSGG	SVL	LVSGGGV	DTPDSSSSSRGIDRTPIVGLSQIDAPEDGTASTATTVQMFGGALYQALSPYVLFTFVNARSGGSVLLVSGGGV	1	0.05
AS (2021)_MLG254	Asia	DT	PDSSLSSRGI	DR	TPIVCLSQIDA	PEDGTASTATTVQM	FGG	AL	YQ	AL	SPYV	LFTFV	HARSGG	SVL	LVSGGGV	DTPDSSLSSRGIDRTPIVCLSQIDAPEDGTASTATTVQMFGGALYQALSPYVLFTFVHARSGGSVLLVSGGGV	1	0.05
AS (2021)_MLG255	Asia	DT	PDSSSSSRGI	DR	TPIVGLSQIDA	PEDGTASTATTVQM	FGG	AL	YQ	AP	SPYV	LFTFV	HARSGG	SVL	LVSGGGV	DTPDSSSSSRGIDRTPIVGLSQIDAPEDGTASTATTVQMFGGALYQAPSPYVLFTFVHARSGGSVLLVSGGGV	1	0.05
AS (2021)_MLG256	Asia	DT	PDSSSSSRGI	DR	IPIVGLSQIDA	PEDGTASTATTVQM	LGG	AL	YQ	AL	SPYV	LFTFV	HARSGG	SVL	LVSGGGV	DTPDSSSSSRGIDRIPIVGLSQIDAPEDGTASTATTVQMLGGALYQALSPYVLFTFVHARSGGSVLLVSGGGV	1	0.05
AS (2021)_MLG257	Asia	DT	TDSSSSSRGI	DR	TPIVGLSQIDA	PEDGTASTATTVQM	FGG	AL	YQ	AL	SPYV	LFTFV	HARSGG	SVL	LVSGGGV	DTTDSSSSSRGIDRTPIVGLSQIDAPEDGTASTATTVQMFGGALYQALSPYVLFTFVHARSGGSVLLVSGGGV	1	0.05
AS (2021)_MLG258	Asia	DT	PDSSLSSRGI	DR	TPIVGLSHIDA	PEDGTASIATTVQM	FGG	AL	YQ	AL	SPYV	LFTFV	HARSGG	SVL	LVSGGGV	DTPDSSLSSRGIDRTPIVGLSHIDAPEDGTASIATTVQMFGGALYQALSPYVLFTFVHARSGGSVLLVSGGGV	1	0.05
AS (2021)_MLG259	Asia	DT	PDSSSSSRGI	DR	TPIVGLSQIDA	PEDGTASTATTVQM	FGG	AL	YQ	AL	SPYV	LFIFV	HARSGG	SVL	LVSGGGV	DTPDSSSSSRGIDRTPIVGLSQIDAPEDGTASTATTVQMFGGALYQALSPYVLFIFVHARSGGSVLLVSGGGV	1	0.05
AS (2021)_MLG260	Asia	DT	PDSSSSSRGI	DR	TPIVSLSQIDA	PEDGTASTATTVQM	FGG	AF	YQ	AL	SPYV	LFTFV	HARSGG	SVL	LVSGGGV	DTPDSSSSSRGIDRTPIVSLSQIDAPEDGTASTATTVQMFGGAFYQALSPYVLFTFVHARSGGSVLLVSGGGV	1	0.05
AS (2021)_MLG261	Asia	DT	PDSSLSSRGI	DR	TPIVGLSQIDA	PEDGTASTATTGQM	FGG	AL	YQ	AL	SPYV	LFTFV	HARSGG	SLL	LVSGGGV	DTPDSSLSSRGIDRTPIVGLSQIDAPEDGTASTATTGQMFGGALYQALSPYVLFTFVHARSGGSLLLVSGGGV	1	0.05
AS (2021)_MLG262	Asia	DT	PDSSSSSRGI	DR	TPIVCLSQIDA	PEDGTASTATTVQM	FGG	AL	YQ	AL	SPYV	LFTFV	HARSGG	SVL	LVSGGGV	DTPDSSSSSRGIDRTPIVCLSQIDAPEDGTASTATTVQMFGGALYQALSPYVLFTFVHARSGGSVLLVSGGGV	1	0.05
AS (2021)_MLG263	Asia	DT	PDSSSSSRGI	DR	IPIVGLSQIDA	PEDGTASTATTVQM	FGG	AF	YQ	AL	SPYV	LFTFV	HARSGG	SVL	LVSGGGV	DTPDSSSSSRGIDRIPIVGLSQIDAPEDGTASTATTVQMFGGAFYQALSPYVLFTFVHARSGGSVLLVSGGGV	1	0.05
AS (2021)_MLG264	Asia	DT	PDSSSSSRGI	DR	TPIVGLSQIDT	PEDGTASTATTVQM	FGG	AL	YQ	AL	SPYV	LFTFV	HARSGG	SVL	LVSGGGV	DTPDSSSSSRGIDRTPIVGLSQIDTPEDGTASTATTVQMFGGALYQALSPYVLFTFVHARSGGSVLLVSGGGV	1	0.05
AS (2021)_MLG265	Asia	DT	PDSSSSSRGI	DR	TPIVGLSQIDA	PEDGTASTATTVQM	FGG	AL	YQ	AL	SPYV	LFTFL	HARSGG	SVL	LVSGGGV	DTPDSSSSSRGIDRTPIVGLSQIDAPEDGTASTATTVQMFGGALYQALSPYVLFTFLHARSGGSVLLVSGGGV	1	0.05
AS (2021)_MLG266	Asia	DT	PDSSSSSRGI	DR	IPIVGLSQIDA	PEDGTASTATTVQM	FGG	AL	YQ	AP	SPYV	LFTFV	HARSGG	SVL	LVSGGGV	DTPDSSSSSRGIDRIPIVGLSQIDAPEDGTASTATTVQMFGGALYQAPSPYVLFTFVHARSGGSVLLVSGGGV	1	0.05
AS (2021)_MLG267	Asia	DT	PDSSSSSRGI	DR	TSIVGLSQIDA	PEDGTASTATTVQM	FGG	AL	YQ	AL	SPYV	LFTFV	HARSGG	SVL	LVSGGGV	DTPDSSSSSRGIDRTSIVGLSQIDAPEDGTASTATTVQMFGGALYQALSPYVLFTFVHARSGGSVLLVSGGGV	1	0.05
AS (2021)_MLG268	Asia	DT	PDSSSSSRGI	DR	IPIVGLSQIDA	PEDGTASTATTVQM	FGG	AL	YQ	AL	SPYV	LFTFV	HARSGG	SLL	LVSGGGV	DTPDSSSSSRGIDRIPIVGLSQIDAPEDGTASTATTVQMFGGALYQALSPYVLFTFVHARSGGSLLLVSGGGV	1	0.05
AS (2021)_MLG269	Asia	DT	LDSSSSSRGI	DR	TPIVGLSQIDA	PEDGTASTATTVQM	FGG	AL	YQ	AL	SPYV	LFTFV	HARSGG	SVL	LVSGGGV	DTLDSSSSSRGIDRTPIVGLSQIDAPEDGTASTATTVQMFGGALYQALSPYVLFTFVHARSGGSVLLVSGGGV	1	0.05
AS (2021)_MLG270	Asia	DT	PDSSSSSRGI	DR	TPIVGLSQIDA	PEDGTASTATTVQM	FGG	AL	YQ	AL	SPYV	LFTFV	QARSGG	SVL	LVSGDGV	DTPDSSSSSRGIDRTPIVGLSQIDAPEDGTASTATTVQMFGGALYQALSPYVLFTFVQARSGGSVLLVSGDGV	1	0.05
AS (2021)_MLG271	Asia	DT	PDSSSSSRGI	DR	TPIVGLSQIDA	PEDGTASTATTVQM	FGG	AL	YQ	AL	LPYV	LFTFV	HARSGG	SVL	LVSGGGV	DTPDSSSSSRGIDRTPIVGLSQIDAPEDGTASTATTVQMFGGALYQALLPYVLFTFVHARSGGSVLLVSGGGV	1	0.05
AS (2021)_MLG272	Asia	DT	PDSSSSSKLI	DR	TPIVGLSQIDA	PEDGTASTATTVQM	FGG	AL	YQ	AL	SPYV	LFTFV	QARSGG	SVL	LVSGGGV	DTPDSSSSSKLIDRTPIVGLSQIDAPEDGTASTATTVQMFGGALYQALSPYVLFTFVQARSGGSVLLVSGGGV	1	0.05
AS (2021)_MLG273	Asia	DT	PDSSSSSKLI	DR	TPIVGLSQIDA	PEDGTASTATTVHM	FGG	AL	YQ	AL	SPYV	LFTFV	QARSGG	SVL	LVSGGGV	DTPDSSSSSKLIDRTPIVGLSQIDAPEDGTASTATTVHMFGGALYQALSPYVLFTFVQARSGGSVLLVSGGGV	1	0.05
AS (2021)_MLG274	Asia	DT	LDSSSSSKRI	DR	TPIVGLSQIDA	PEDGTASTATTVQM	FGG	AF	YQ	AL	SPYV	LFTFV	QARSGG	SVL	LVSGGGV	DTLDSSSSSKRIDRTPIVGLSQIDAPEDGTASTATTVQMFGGAFYQALSPYVLFTFVQARSGGSVLLVSGGGV	1	0.05
AS (2021)_MLG275	Asia	DT	PDSSSSSKRI	DR	TPIVGLSQIDA	PEDGTASIATTVQM	FGG	AL	YQ	AL	SPYV	LFTFV	QARSGG	SVL	LVSGGGV	DTPDSSSSSKRIDRTPIVGLSQIDAPEDGTASIATTVQMFGGALYQALSPYVLFTFVQARSGGSVLLVSGGGV	1	0.05
AS (2021)_MLG276	Asia	DT	PDSSSSSKRI	DR	TLIVGLSQIDA	PEDGTASTATTVQM	FGG	AF	YQ	AL	SSYV	LFTFV	QARSGG	SVL	LVSGGGV	DTPDSSSSSKRIDRTLIVGLSQIDAPEDGTASTATTVQMFGGAFYQALSSYVLFTFVQARSGGSVLLVSGGGV	1	0.05
AS (2021)_MLG277	Asia	DM	PDSSSSSKRI	DR	TPIVGLSQIDA	PEDGTASTATTVQM	FGG	AL	YQ	AL	SPYV	LFTFV	QARSGG	SVL	LVSGGGF	DMPDSSSSSKRIDRTPIVGLSQIDAPEDGTASTATTVQMFGGALYQALSPYVLFTFVQARSGGSVLLVSGGGF	1	0.05
AS (2021)_MLG278	Asia	GT	PDSSSSSKRI	DR	TPIVGLSQIDA	PEDGTASTATTVQM	FGG	AL	YQ	AL	SPYV	LFTFV	QARSGG	SVL	LVSGGGV	GTPDSSSSSKRIDRTPIVGLSQIDAPEDGTASTATTVQMFGGALYQALSPYVLFTFVQARSGGSVLLVSGGGV	1	0.05
AS (2021)_MLG279	Asia	DT	PYSSSSSKRI	DR	TPIVGLSQIDA	PEDGTASTATTVQM	FGG	AF	YQ	AL	SPYV	LFTFV	QARSGG	SVL	LVSGGGV	DTPYSSSSSKRIDRTPIVGLSQIDAPEDGTASTATTVQMFGGAFYQALSPYVLFTFVQARSGGSVLLVSGGGV	1	0.05
AS (2021)_MLG280	Asia	DT	PDSSSSSKRI	DR	TPIVGLSQIDA	PEDGTASTATTVQM	FGG	AL	YQ	AL	SPYV	LFTFV	QARSGG	SVL	LVGGGGV	DTPDSSSSSKRIDRTPIVGLSQIDAPEDGTASTATTVQMFGGALYQALSPYVLFTFVQARSGGSVLLVGGGGV	1	0.05
AS (2021)_MLG281	Asia	DT	PDSSSSSKRI	DR	TPIVGFSQIDA	PEDGTASTATTVQM	FGG	AL	YQ	AL	SPYV	LFTFV	QARSGG	SVL	LVSGGGV	DTPDSSSSSKRIDRTPIVGFSQIDAPEDGTASTATTVQMFGGALYQALSPYVLFTFVQARSGGSVLLVSGGGV	1	0.05
AS (2021)_MLG282	Asia	DT	PDSSSSSKRI	DR	TPIVGLSQIDA	PEDGTASTATTVQM	FGG	AL	YQ	AL	SPYV	LFTFI	QARSGG	SVL	LVSGGGV	DTPDSSSSSKRIDRTPIVGLSQIDAPEDGTASTATTVQMFGGALYQALSPYVLFTFIQARSGGSVLLVSGGGV	1	0.05
AS (2021)_MLG283	Asia	DT	PDSSSSSKRI	DR	TPIVGLSQIDA	PEDGTASTATTVQM	FGG	AL	YQ	AL	SPYV	LFTFV	QARSGG	SVL	LVSGDGV	DTPDSSSSSKRIDRTPIVGLSQIDAPEDGTASTATTVQMFGGALYQALSPYVLFTFVQARSGGSVLLVSGDGV	1	0.05
AS (2021)_MLG284	Asia	DT	PDSSSSSKRI	DR	TPIVGLSQIDA	PEDGTASTATTVQM	FGG	AL	YQ	AL	SPYV	LFTFV	QARFGG	SVL	LVNGGGV	DTPDSSSSSKRIDRTPIVGLSQIDAPEDGTASTATTVQMFGGALYQALSPYVLFTFVQARFGGSVLLVNGGGV	1	0.05
AS (2021)_MLG285	Asia	DT	PDSSLSSKRI	DR	TPIVGLSQIDA	PEDGTASTATTVQM	FGG	AL	YQ	AL	SPYV	LFTFV	QARSGG	SVL	LVSGGGV	DTPDSSLSSKRIDRTPIVGLSQIDAPEDGTASTATTVQMFGGALYQALSPYVLFTFVQARSGGSVLLVSGGGV	1	0.05
AS (2021)_MLG286	Asia	DT	LDSSSSSKRI	DR	TPIVGLSQIDA	PEDGTASTATTVQM	FGG	AL	YQ	AL	SPYV	LFTFV	QARSGG	SVL	LVSGGGV	DTLDSSSSSKRIDRTPIVGLSQIDAPEDGTASTATTVQMFGGALYQALSPYVLFTFVQARSGGSVLLVSGGGV	1	0.05
AS (2021)_MLG287	Asia	DT	PDSSSSSKRI	DR	TPIVGLSQIDA	PEDGTASTATTVQM	FGG	AF	YQ	AL	SPYV	LFTFV	QARSGG	SVL	FVSGGGV	DTPDSSSSSKRIDRTPIVGLSQIDAPEDGTASTATTVQMFGGAFYQALSPYVLFTFVQARSGGSVLFVSGGGV	1	0.05
AS (2021)_MLG288	Asia	DT	PDSSSSSKRI	DR	TPIVGLSQIDA	PEDGTASTATTVQM	FGG	AL	YQ	AL	SPYV	LFTFV	QARSGS	SVL	LVSGGGF	DTPDSSSSSKRIDRTPIVGLSQIDAPEDGTASTATTVQMFGGALYQALSPYVLFTFVQARSGSSVLLVSGGGF	1	0.05
AS (2021)_MLG289	Asia	DT	PDSSSSSKRI	DR	TPIVGLSQIDA	PEDGTASTATTVQM	FGG	AL	YQ	AL	SSYV	LFTFV	QARSGG	SVL	FVSGGGV	DTPDSSSSSKRIDRTPIVGLSQIDAPEDGTASTATTVQMFGGALYQALSSYVLFTFVQARSGGSVLFVSGGGV	1	0.05
AS (2021)_MLG290	Asia	DT	PDSSSSSKRI	DR	TPIVGLSQIDA	PEDGTASTATTVQM	FGG	AL	YQ	VL	SPYV	LFTFV	QARSGG	SVL	LVSGGGV	DTPDSSSSSKRIDRTPIVGLSQIDAPEDGTASTATTVQMFGGALYQVLSPYVLFTFVQARSGGSVLLVSGGGV	1	0.05
AS (2021)_MLG291	Asia	DT	PDSSSSSKRI	DR	TPIVGLSQIDA	PEDGTDSTATTVQM	FGG	AF	YQ	AL	SPYV	LFTFV	QARSGG	SVL	LVSGGGV	DTPDSSSSSKRIDRTPIVGLSQIDAPEDGTDSTATTVQMFGGAFYQALSPYVLFTFVQARSGGSVLLVSGGGV	1	0.05
AS (2021)_MLG292	Asia	DT	PDSSSSSKRI	DR	TPIVGLSQIDA	PEDGTASTATTVQM	FGG	AF	YQ	AL	SPYV	LFTFV	HARSGG	SLL	LVIGGGV	DTPDSSSSSKRIDRTPIVGLSQIDAPEDGTASTATTVQMFGGAFYQALSPYVLFTFVHARSGGSLLLVIGGGV	1	0.05
AS (2021)_MLG293	Asia	DT	PDSSSSSKRI	DR	TPIVGLSQIDA	PEYGTASTATTVQM	FGG	AF	YQ	AL	SPYV	LFTFV	QARSGG	SLL	LVSGGGV	DTPDSSSSSKRIDRTPIVGLSQIDAPEYGTASTATTVQMFGGAFYQALSPYVLFTFVQARSGGSLLLVSGGGV	1	0.05
AS (2021)_MLG294	Asia	DT	PDSSSSSKRI	DR	TPIVGLSQIDA	PEDGTASKATTVQM	FGG	AL	YQ	AL	SPYV	LFTFV	QARSGG	SVL	LVSGGGV	DTPDSSSSSKRIDRTPIVGLSQIDAPEDGTASKATTVQMFGGALYQALSPYVLFTFVQARSGGSVLLVSGGGV	1	0.05
AS (2021)_MLG295	Asia	DT	PDSSSSSKRI	DR	TPIVGLSQIDA	PEDGTASTATTVQM	FGG	AL	YQ	AL	SPYV	LFTFV	QARSGG	SVL	LVSGGVV	DTPDSSSSSKRIDRTPIVGLSQIDAPEDGTASTATTVQMFGGALYQALSPYVLFTFVQARSGGSVLLVSGGVV	1	0.05
AS (2021)_MLG296	Asia	DT	PDASSSSKRI	DR	TPIVGLSQIDA	PEDGTASTATTVQM	FGG	AL	YQ	AL	SPYV	LFTFV	QARSGG	SVL	LVSGGGV	DTPDASSSSKRIDRTPIVGLSQIDAPEDGTASTATTVQMFGGALYQALSPYVLFTFVQARSGGSVLLVSGGGV	1	0.05
AS (2021)_MLG297	Asia	DT	PDSSSLSSGI	DR	TPIVGLSQIDA	PEDGTASTATTVQM	FGG	AF	YQ	AL	SPYV	LFTFV	HARSGG	SVL	LVSGGGV	DTPDSSSLSSGIDRTPIVGLSQIDAPEDGTASTATTVQMFGGAFYQALSPYVLFTFVHARSGGSVLLVSGGGV	1	0.05
AS (2021)_MLG298	Asia	DT	PDSSSSSKGI	DR	TPIVGLSQIDA	PEDGTASTATTVQM	FGG	AL	YQ	AL	SPYV	LFTFV	QARSGG	SVL	LVSGDGV	DTPDSSSSSKGIDRTPIVGLSQIDAPEDGTASTATTVQMFGGALYQALSPYVLFTFVQARSGGSVLLVSGDGV	1	0.05
AS (2021)_MLG299	Asia	DT	PDSSSSSSGI	DR	TPIVGLSQIDA	PEDGTASTATTVQM	FGG	AL	YQ	AL	SPYV	LFTFV	HARSGG	SVL	LVSGGGF	DTPDSSSSSSGIDRTPIVGLSQIDAPEDGTASTATTVQMFGGALYQALSPYVLFTFVHARSGGSVLLVSGGGF	1	0.05
AS (2021)_MLG300	Asia	DT	LDSSSSSMGI	DR	TPIVGLSQIDA	PEDGTASTATTVQM	FGG	AL	YQ	AL	SPYV	LFTFV	QARSGG	SVL	LVSGGGV	DTLDSSSSSMGIDRTPIVGLSQIDAPEDGTASTATTVQMFGGALYQALSPYVLFTFVQARSGGSVLLVSGGGV	1	0.05
EU (2020)_MLG1	Europe	DT	PDSSSSSKRI	DR	TPIVGLSQIPA	SPEDGTASTATTVQM	FGG	AL	YQ	AL	SPYV	LFTFV	QARSGG	SVL	LVSGGGV	DTPDSSSSSKRIDRTPIVGLSQIPASPEDGTASTATTVQMFGGALYQALSPYVLFTFVQARSGGSVLLVSGGGV	272	22.90
EU (2020)_MLG2	Europe	DT	PDSSSSSRGI	DR	TPIVGLSQIPA	SPEDGTASTATTVQM	FGG	AL	YQ	AL	SPYV	LFTFV	QARSGG	SVL	LVSGGGV	DTPDSSSSSRGIDRTPIVGLSQIPASPEDGTASTATTVQMFGGALYQALSPYVLFTFVQARSGGSVLLVSGGGV	272	22.90
EU (2020)_MLG3	Europe	DT	PDSSSSSRGI	DR	IPIVGLSQIPA	SPEDGTASTATTVQM	FGG	AL	YQ	AL	SPYV	LFTFV	HARSGG	SVL	LVSGGGV	DTPDSSSSSRGIDRIPIVGLSQIPASPEDGTASTATTVQMFGGALYQALSPYVLFTFVHARSGGSVLLVSGGGV	117	9.85
EU (2020)_MLG4	Europe	DT	PDSSSSSRGI	DR	TPIVGLSQIPA	SPEDGTASTATTVQM	FGG	AL	YQ	AL	SPYV	LFTFV	HARSGG	SVL	LVSGGGV	DTPDSSSSSRGIDRTPIVGLSQIPASPEDGTASTATTVQMFGGALYQALSPYVLFTFVHARSGGSVLLVSGGGV	44	3.70
EU (2020)_MLG5	Europe	DT	PDSSSSSRGI	DR	TPIVGLSQIPA	SPEDGTASTATTVQM	FGG	AL	YQ	AL	LPYV	LFTFV	HARSGG	SVL	LVSGGGV	DTPDSSSSSRGIDRTPIVGLSQIPASPEDGTASTATTVQMFGGALYQALLPYVLFTFVHARSGGSVLLVSGGGV	37	3.11
EU (2020)_MLG6	Europe	DT	PDSSSLSRGI	DR	TPIVGLSQIPA	SPEDGTASTATTVQM	YGG	AL	YQ	AP	SPYV	LFTFV	QARSVG	SVS	LVSGDGV	DTPDSSSLSRGIDRTPIVGLSQIPASPEDGTASTATTVQMYGGALYQAPSPYVLFTFVQARSVGSVSLVSGDGV	32	2.69
EU (2020)_MLG7	Europe	DT	PDSSSSSRGI	DR	IPIVGLSQIPA	SPEDGTASTATTVQM	FGG	AL	YQ	AL	SPYV	LFTFV	HARSGG	SVL	LGSGGGV	DTPDSSSSSRGIDRIPIVGLSQIPASPEDGTASTATTVQMFGGALYQALSPYVLFTFVHARSGGSVLLGSGGGV	29	2.44
EU (2020)_MLG8	Europe	DT	PDSSSSSKRI	DR	TPIVGLSQIPA	SPEDGTASTATTVQM	FGG	AL	YQ	AL	SPYV	LFTFV	QARSGG	SVL	LGSGGGV	DTPDSSSSSKRIDRTPIVGLSQIPASPEDGTASTATTVQMFGGALYQALSPYVLFTFVQARSGGSVLLGSGGGV	29	2.44
EU (2020)_MLG9	Europe	DT	PDSSSSSRGI	DR	TPIVGLSQIPA	SPEDGTASTATTVQM	FGG	AL	YQ	AL	SPYV	LFTFV	QARSGG	SVL	LGSGGGV	DTPDSSSSSRGIDRTPIVGLSQIPASPEDGTASTATTVQMFGGALYQALSPYVLFTFVQARSGGSVLLGSGGGV	29	2.44
EU (2020)_MLG10	Europe	GT	PDSSSSSRGI	DR	TPIVGLSQIPA	SPEDGTASTATTVQM	FGG	AL	YQ	AL	SPYV	LFTFV	QARSGG	SVL	LVSGGGV	GTPDSSSSSRGIDRTPIVGLSQIPASPEDGTASTATTVQMFGGALYQALSPYVLFTFVQARSGGSVLLVSGGGV	28	2.36
EU (2020)_MLG11	Europe	DT	PDSSSSSRGI	DR	TPIVGLSQIPA	SPEDGTASTATTVQM	FGG	AF	YQ	AP	SPYV	LFTFV	QARSGV	SVL	LVSGDGV	DTPDSSSSSRGIDRTPIVGLSQIPASPEDGTASTATTVQMFGGAFYQAPSPYVLFTFVQARSGVSVLLVSGDGV	21	1.77
EU (2020)_MLG12	Europe	DM	PDSSSSSKRI	DR	TPIVGLSQIPA	SPEDGTASTATTVQM	FGG	AL	YQ	AL	SPYV	LFTFV	QARSGG	SVL	LVSGGGV	DMPDSSSSSKRIDRTPIVGLSQIPASPEDGTASTATTVQMFGGALYQALSPYVLFTFVQARSGGSVLLVSGGGV	19	1.60
EU (2020)_MLG13	Europe	DT	PDSSSSSRGI	DR	IPIVGLSQIPA	SPEDGTASTATTVQM	FGG	AL	YQ	AL	SPYV	LFTFV	HVRSGG	SVL	LVSGGGV	DTPDSSSSSRGIDRIPIVGLSQIPASPEDGTASTATTVQMFGGALYQALSPYVLFTFVHVRSGGSVLLVSGGGV	18	1.52
EU (2020)_MLG14	Europe	DT	PDSSSSSRGI	DR	TPIVGLSQIPA	SPEDGTASTATTVQM	FGG	AL	YQ	AP	SPYV	LFTFV	QARSGG	SVS	LVSGDGV	DTPDSSSSSRGIDRTPIVGLSQIPASPEDGTASTATTVQMFGGALYQAPSPYVLFTFVQARSGGSVSLVSGDGV	17	1.43
EU (2020)_MLG15	Europe	DM	PDSSSSSKRI	DR	TPIVGLSQIPA	SPEDGTASTATTVQM	FGG	AL	YQ	AL	SPYV	LFTFV	QARSGG	SVL	LGSGGGV	DMPDSSSSSKRIDRTPIVGLSQIPASPEDGTASTATTVQMFGGALYQALSPYVLFTFVQARSGGSVLLGSGGGV	16	1.35

EU (2020)_MLG16	Europe	DT	PDSSSSSKRI	DR	TPIVGLSQIPA	SPEDGTASTATSVQM	FGG	AL	YQ	AL	SPYV	LFTFV	QARSGG	SVL	LVSGGGV	DTPDSSSSSKRIDRTPIVGLSQIPASPEDGTASTATSVQMFGGALYQALSPYVLFTFVQARSGGSVLLVSGGGV	13	1.09
EU (2020)_MLG17	Europe	DT	PDSSSSSRGI	DR	TPIVGLSQIPA	SPEDGTASTATTVQM	FGG	AL	YQ	AP	SPYV	LFTFV	QARSGG	SVL	LVSGDGV	DTPDSSSSSRGIDRTPIVGLSQIPASPEDGTASTATTVQMFGGALYQAPSPYVLFTFVQARSGGSVLLVSGDGV	12	1.01
EU (2020)_MLG18	Europe	DT	PDSSSSSRGI	DR	TPIVGLSQIPA	SPEDGTASTATTVHM	FGG	AL	YQ	AL	SPYV	LFTFV	HARSGG	SVL	LVSGGGV	DTPDSSSSSRGIDRTPIVGLSQIPASPEDGTASTATTVHMFGGALYQALSPYVLFTFVHARSGGSVLLVSGGGV	11	0.93
EU (2020)_MLG19	Europe	DT	PDSSSSSRGI	DR	IPIVGLSQIPA	SPEDGTASTATTVQM	FGG	AF	YQ	AL	SPYV	LFTFV	HARSGG	SVL	LVSGGGV	DTPDSSSSSRGIDRIPIVGLSQIPASPEDGTASTATTVQMFGGAFYQALSPYVLFTFVHARSGGSVLLVSGGGV	10	0.84
EU (2020)_MLG20	Europe	DT	PDSSSSSKRI	DR	TPIVGLSQIPA	SPEDGIASTATTVQM	FSG	AL	YQ	AL	SPYV	LFTFV	QARSGG	SVL	LVSGGGV	DTPDSSSSSKRIDRTPIVGLSQIPASPEDGIASTATTVQMFSGALYQALSPYVLFTFVQARSGGSVLLVSGGGV	10	0.84
EU (2020)_MLG21	Europe	DT	PDSSSSSKRI	DR	TPIVGLSQVPA	SPEDGTASTATTVQM	FGG	AL	YQ	AL	SPYV	LFTFV	QARSGG	SVL	LVSGGGV	DTPDSSSSSKRIDRTPIVGLSQVPASPEDGTASTATTVQMFGGALYQALSPYVLFTFVQARSGGSVLLVSGGGV	8	0.67
EU (2020)_MLG22	Europe	DT	PDSSSSSRGI	DR	TPIVDLSQIPT	SPEDGTASTATTVQM	FGG	AL	YQ	AP	SPYV	LFTFV	QARSGG	SVL	LVSGDGV	DTPDSSSSSRGIDRTPIVDLSQIPTSPEDGTASTATTVQMFGGALYQAPSPYVLFTFVQARSGGSVLLVSGDGV	7	0.59
EU (2020)_MLG23	Europe	DT	PDSSSSSKRI	DR	TPIVGLSQIPA	SPEEGTASTATTVQM	FGG	AL	YQ	AL	SPYV	LFTFV	QARSGG	SVL	LVSGGGV	DTPDSSSSSKRIDRTPIVGLSQIPASPEEGTASTATTVQMFGGALYQALSPYVLFTFVQARSGGSVLLVSGGGV	7	0.59
EU (2020)_MLG24	Europe	DT	PDSSSSSRGI	DR	TPIVGLSQIPA	SPEDGTASTATSVQM	FGG	AL	YQ	AL	SPYV	LFTFV	QARSGG	SVL	LVSGGGV	DTPDSSSSSRGIDRTPIVGLSQIPASPEDGTASTATSVQMFGGALYQALSPYVLFTFVQARSGGSVLLVSGGGV	5	0.42
EU (2020)_MLG25	Europe	DT	PDSSLSSRGI	DR	TPIVGLSQIPA	SPEDGTASTATTVQM	FGG	AL	YQ	AL	SPYV	LFTFV	QARSGG	SVL	LVSGGGV	DTPDSSLSSRGIDRTPIVGLSQIPASPEDGTASTATTVQMFGGALYQALSPYVLFTFVQARSGGSVLLVSGGGV	5	0.42
EU (2020)_MLG26	Europe	DT	PDSSSSSRGI	DR	TPIVGLSQIPA	SPEDGTASTATTVQM	FGG	AL	YQ	AP	SPYV	LFTFV	QARSGG	SVL	LVSGGGV	DTPDSSSSSRGIDRTPIVGLSQIPASPEDGTASTATTVQMFGGALYQAPSPYVLFTFVQARSGGSVLLVSGGGV	5	0.42
EU (2020)_MLG27	Europe	DT	PDSSSSSKRI	DR	TPIVGLSQIPA	SPEDGTASTATTVQM	FGG	AF	YQ	AL	SPYV	LFTFV	QARSGG	SVL	LVSGGGV	DTPDSSSSSKRIDRTPIVGLSQIPASPEDGTASTATTVQMFGGAFYQALSPYVLFTFVQARSGGSVLLVSGGGV	5	0.42
EU (2020)_MLG28	Europe	DT	PDSSSSSRGI	DR	TPIVGLSQIPA	SPEDGTASTATTVQM	FGG	AL	YQ	AP	SPYV	LFTFV	QARSGG	SVL	LGSGDGV	DTPDSSSSSRGIDRTPIVGLSQIPASPEDGTASTATTVQMFGGALYQAPSPYVLFTFVQARSGGSVLLGSGDGV	5	0.42
EU (2020)_MLG29	Europe	DT	PDSSSSSRGI	DR	TPIVGLSQIPA	SPEDGTASTATTVQM	FGG	AL	YQ	AP	SPYV	LFTFV	QARSGV	SVL	LVSGDGV	DTPDSSSSSRGIDRTPIVGLSQIPASPEDGTASTATTVQMFGGALYQAPSPYVLFTFVQARSGVSVLLVSGDGV	4	0.34
EU (2020)_MLG30	Europe	DT	PDSSSSSKRI	DR	TPIVGLSQIPA	SPEDGTASTATTVQM	FGS	AL	YQ	AL	SPYV	LFTFV	QARSGG	SVL	LVSGGGV	DTPDSSSSSKRIDRTPIVGLSQIPASPEDGTASTATTVQMFGSALYQALSPYVLFTFVQARSGGSVLLVSGGGV	4	0.34
EU (2020)_MLG31	Europe	DT	PDSSSSNRGI	DR	TPIVGLSQIPA	SPEDGTASTATTVQM	FGG	AL	YQ	AP	SPYV	LFTFV	QARSGG	SVS	LVSGDGV	DTPDSSSSNRGIDRTPIVGLSQIPASPEDGTASTATTVQMFGGALYQAPSPYVLFTFVQARSGGSVSLVSGDGV	4	0.34
EU (2020)_MLG32	Europe	DT	PDSSSSSKRI	DR	TPIVGLSQVPA	SPEDGIASTATTVQM	FSG	AL	YQ	AL	SPYV	LFTFV	QARSGG	SVL	LVSGGGV	DTPDSSSSSKRIDRTPIVGLSQVPASPEDGIASTATTVQMFSGALYQALSPYVLFTFVQARSGGSVLLVSGGGV	4	0.34
EU (2020)_MLG33	Europe	DT	PDSSSSSKRI	DR	TPIVGLSQIPA	SPEEGTASTATTVQM	FGG	AL	YQ	AL	SPYV	LFTFV	QARSGG	SVL	LGSGGGV	DTPDSSSSSKRIDRTPIVGLSQIPASPEEGTASTATTVQMFGGALYQALSPYVLFTFVQARSGGSVLLGSGGGV	3	0.25
EU (2020)_MLG34	Europe	DT	PDSSSSSRGI	DR	TPIVGLSQIPA	SPEDGTASTATTVQM	FGG	AF	YQ	AP	SPYV	LFTFV	QARSGV	SVL	LGSGDGV	DTPDSSSSSRGIDRTPIVGLSQIPASPEDGTASTATTVQMFGGAFYQAPSPYVLFTFVQARSGVSVLLGSGDGV	3	0.25
EU (2020)_MLG35	Europe	DT	PDSSSSSRGI	DR	TPIVGLSQIPA	SPEDGTASTATTVQM	FGG	AL	YQ	AP	SPYV	LFTFV	QARSGG	SVS	LGSGDGV	DTPDSSSSSRGIDRTPIVGLSQIPASPEDGTASTATTVQMFGGALYQAPSPYVLFTFVQARSGGSVSLGSGDGV	3	0.25
EU (2020)_MLG36	Europe	DT	PDSSSSSRGI	DR	TPIVDLSQIPT	SPEDGTASTATTVQM	FGG	AL	YQ	AP	SPYV	LFTFV	QARSGG	SVL	LGSGDGV	DTPDSSSSSRGIDRTPIVDLSQIPTSPEDGTASTATTVQMFGGALYQAPSPYVLFTFVQARSGGSVLLGSGDGV	3	0.25
EU (2020)_MLG37	Europe	DT	PDSSSSSRGI	DR	TPIVGLSQIPA	SPEDGTASTATTVQM	FGG	AF	YQ	AP	SPYV	LFTFV	QARSGG	SVL	LVSGDGV	DTPDSSSSSRGIDRTPIVGLSQIPASPEDGTASTATTVQMFGGAFYQAPSPYVLFTFVQARSGGSVLLVSGDGV	3	0.25
EU (2020)_MLG38	Europe	DT	PDSSSSSRGI	DR	TPIIGLSQIPA	SPEDGTASTATTVQM	FGG	AF	YQ	AP	SPYV	LFTFV	QARSGG	SVL	LVSGDGV	DTPDSSSSSRGIDRTPIIGLSQIPASPEDGTASTATTVQMFGGAFYQAPSPYVLFTFVQARSGGSVLLVSGDGV	3	0.25
EU (2020)_MLG39	Europe	DT	PDSSSLSRGI	DR	TPIVGLSQIPA	SPEDGTASTATTVQM	YGG	AL	YQ	AP	SPYV	LFTFV	QARSGG	SVS	LVSGDGV	DTPDSSSLSRGIDRTPIVGLSQIPASPEDGTASTATTVQMYGGALYQAPSPYVLFTFVQARSGGSVSLVSGDGV	3	0.25
EU (2020)_MLG40	Europe	DT	PDSSLSSRGI	DR	TPIVGLSQIPA	SPEDGTASTATTVQM	FGG	AL	YQ	AL	SPYV	LFTFV	HARSGG	SVL	LVSGGGV	DTPDSSLSSRGIDRTPIVGLSQIPASPEDGTASTATTVQMFGGALYQALSPYVLFTFVHARSGGSVLLVSGGGV	3	0.25
EU (2020)_MLG41	Europe	DT	PDSSSSSRGI	DR	TPIVGLSQVPA	SPEDGTASTATTVQM	FGG	AL	YQ	AL	SPYV	LFTFV	QARSGG	SVL	LVSGGGV	DTPDSSSSSRGIDRTPIVGLSQVPASPEDGTASTATTVQMFGGALYQALSPYVLFTFVQARSGGSVLLVSGGGV	3	0.25
EU (2020)_MLG42	Europe	DT	PDSSSSSRGI	DR	TPIVGLSQVSA	SPEDGTASTATTVQM	FGG	AF	YQ	AP	SPYV	LFTFV	QARSGV	SVL	LVSGDGV	DTPDSSSSSRGIDRTPIVGLSQVSASPEDGTASTATTVQMFGGAFYQAPSPYVLFTFVQARSGVSVLLVSGDGV	2	0.17
EU (2020)_MLG43	Europe	DT	PDSSSSSRGI	DR	TPIVGLSQIPA	SPEDGTASTATTVQM	FGG	AF	YQ	AL	SPYV	LFTFV	HARSGG	SVL	LVSGGGV	DTPDSSSSSRGIDRTPIVGLSQIPASPEDGTASTATTVQMFGGAFYQALSPYVLFTFVHARSGGSVLLVSGGGV	2	0.17
EU (2020)_MLG44	Europe	DT	PDSSSSSRGI	DR	TPIVCLSQIPA	SPEDGTASTATTVQM	FGG	AL	YQ	AL	SPYV	LFTFV	QARSGG	SVL	LVSGGGV	DTPDSSSSSRGIDRTPIVCLSQIPASPEDGTASTATTVQMFGGALYQALSPYVLFTFVQARSGGSVLLVSGGGV	2	0.17
EU (2020)_MLG45	Europe	DT	PDSSSSSRGI	DR	IPIVGLSQIPA	SPEDGTASTATTVQM	FGG	AF	YQ	AL	SPYV	LFTFV	HVRSGG	SVL	LVSGGGV	DTPDSSSSSRGIDRIPIVGLSQIPASPEDGTASTATTVQMFGGAFYQALSPYVLFTFVHVRSGGSVLLVSGGGV	2	0.17
EU (2020)_MLG46	Europe	DT	PDSSSSSKRI	DR	IPIVGLSQIPA	SPEEGTASTATTVQM	FGG	AL	YQ	AL	SPYV	LFTFV	HARSGG	SVL	LVSGGGV	DTPDSSSSSKRIDRIPIVGLSQIPASPEEGTASTATTVQMFGGALYQALSPYVLFTFVHARSGGSVLLVSGGGV	2	0.17
EU (2020)_MLG47	Europe	DT	PDSSSSSKRI	DR	TPIVGLSQIPA	SPEDGTASTATTVQM	FGG	AL	YQ	AL	SPYV	LFTFV	QARSGG	SVL	FVSGGGV	DTPDSSSSSKRIDRTPIVGLSQIPASPEDGTASTATTVQMFGGALYQALSPYVLFTFVQARSGGSVLFVSGGGV	2	0.17
EU (2020)_MLG48	Europe	DT	PDSSSSSKRI	DR	TPIVGLSQIPA	LPEDGTASTATSVQM	FGG	AL	YQ	AL	SPYV	LFTFV	QARSGG	SVL	LVSGGGV	DTPDSSSSSKRIDRTPIVGLSQIPALPEDGTASTATSVQMFGGALYQALSPYVLFTFVQARSGGSVLLVSGGGV	1	0.08
EU (2020)_MLG49	Europe	DT	PDSSSSSKRI	DR	TPIVGLSQIPA	SPEDGTASTATTVQM	FGG	AL	YQ	AL	SSYV	LFTFV	QARSGG	SVL	LVSGGGV	DTPDSSSSSKRIDRTPIVGLSQIPASPEDGTASTATTVQMFGGALYQALSSYVLFTFVQARSGGSVLLVSGGGV	1	0.08
EU (2020)_MLG50	Europe	DT	PDSSSSSKRI	DR	TPIVGLSQIPA	SPEDGTASTATTVQM	FGG	AF	YQ	AP	SPYV	LFTFV	QARSGG	SVL	LVSGGGV	DTPDSSSSSKRIDRTPIVGLSQIPASPEDGTASTATTVQMFGGAFYQAPSPYVLFTFVQARSGGSVLLVSGGGV	1	0.08
EU (2020)_MLG51	Europe	DT	PDSSSSSRGI	DR	TPIVDLSQIPT	SPEDGTASTATTVQM	FGG	AF	YQ	AP	SPYV	LFTFV	QARSGG	SVL	LGSGDGV	DTPDSSSSSRGIDRTPIVDLSQIPTSPEDGTASTATTVQMFGGAFYQAPSPYVLFTFVQARSGGSVLLGSGDGV	1	0.08
EU (2020)_MLG52	Europe	GT	PDSSSSSRGI	DC	TPIVGLSQIPA	SPEDGTASTATTVQM	FGG	AL	YQ	AL	SPYV	LFTFV	QARSGG	SVL	LVSGGGV	GTPDSSSSSRGIDCTPIVGLSQIPASPEDGTASTATTVQMFGGALYQALSPYVLFTFVQARSGGSVLLVSGGGV	1	0.08
EU (2020)_MLG53	Europe	DT	PDSSSSSKRI	DR	TPIVGLSQIPA	SPEDGTASTATTVQM	FGG	AF	YQ	AL	SPYV	LFTFV	QARSGG	SVL	FVSGGGV	DTPDSSSSSKRIDRTPIVGLSQIPASPEDGTASTATTVQMFGGAFYQALSPYVLFTFVQARSGGSVLFVSGGGV	1	0.08
EU (2020)_MLG54	Europe	DT	PDSSSSSKRI	DR	TPIVGLSQIPA	LPEDGTASTATTVQM	FGG	AL	YQ	AL	SPYV	LFTFV	QARSGG	SVL	LVSGGGV	DTPDSSSSSKRIDRTPIVGLSQIPALPEDGTASTATTVQMFGGALYQALSPYVLFTFVQARSGGSVLLVSGGGV	1	0.08
EU (2020)_MLG55	Europe	DT	PDSSSSCRGI	DR	TPIVGLSQIPA	SPEDGTASTATTVQM	FGG	AL	YQ	AL	LPYV	LFTFV	HARSGG	SVL	LVSGGGV	DTPDSSSSCRGIDRTPIVGLSQIPASPEDGTASTATTVQMFGGALYQALLPYVLFTFVHARSGGSVLLVSGGGV	1	0.08
EU (2020)_MLG56	Europe	DT	PDSSSSSRGI	DR	TPIVGLSQIPA	SPEDGTASTATTVQM	FGG	AF	YQ	AL	SPYV	LFTFV	HARSGG	SVL	LGSGGGV	DTPDSSSSSRGIDRTPIVGLSQIPASPEDGTASTATTVQMFGGAFYQALSPYVLFTFVHARSGGSVLLGSGGGV	1	0.08
EU (2020)_MLG57	Europe	DT	PDSSSSSRGI	DR	TPIVGLSQIPA	SPEDGTASTATSVQM	FGG	AF	YQ	AL	SPYV	LFTFV	QARSGG	SVL	LVSGGGV	DTPDSSSSSRGIDRTPIVGLSQIPASPEDGTASTATSVQMFGGAFYQALSPYVLFTFVQARSGGSVLLVSGGGV	1	0.08
EU (2020)_MLG58	Europe	DT	PDSSLSSRGI	DR	TPIVGLSQIPA	SPEDGTASTATTVQM	FGG	AL	YQ	AP	SPYV	LFTFV	QARSGG	SVL	LVSGDGV	DTPDSSLSSRGIDRTPIVGLSQIPASPEDGTASTATTVQMFGGALYQAPSPYVLFTFVQARSGGSVLLVSGDGV	1	0.08
EU (2020)_MLG59	Europe	DT	PDSSSSSKRI	DR	TPIVGLSQIPV	SPEDGTASTATTVQM	FGG	AL	YQ	AL	SPYV	LFTFV	QARSGG	SVL	LVSGGGV	DTPDSSSSSKRIDRTPIVGLSQIPVSPEDGTASTATTVQMFGGALYQALSPYVLFTFVQARSGGSVLLVSGGGV	1	0.08
EU (2020)_MLG60	Europe	DT	LDSSSSSKRI	DR	TPIVGLSQIPA	SPEDGTASTATSVQM	FGG	AL	YQ	AL	SPYV	LFTFV	QARSGG	SVL	LVSGGGV	DTLDSSSSSKRIDRTPIVGLSQIPASPEDGTASTATSVQMFGGALYQALSPYVLFTFVQARSGGSVLLVSGGGV	1	0.08
EU (2020)_MLG61	Europe	DT	PDSSSSSKRI	DR	TPIVGLSQIPA	SPEDGTASTATTVQM	FGG	AL	YQ	AL	SPYV	LFTFV	QVRSGG	SVL	LVSGGGV	DTPDSSSSSKRIDRTPIVGLSQIPASPEDGTASTATTVQMFGGALYQALSPYVLFTFVQVRSGGSVLLVSGGGV	1	0.08
EU (2020)_MLG62	Europe	DT	PDSSSSIKRI	DR	TPIVGLSQIPA	SPEDGTASTATTVQM	FGG	AL	YQ	AL	SPYV	LFTFV	QARSGG	SVL	LGSGGGV	DTPDSSSSIKRIDRTPIVGLSQIPASPEDGTASTATTVQMFGGALYQALSPYVLFTFVQARSGGSVLLGSGGGV	1	0.08
EU (2020)_MLG63	Europe	DT	LDSSSSSRGI	DR	TPIVGLSQIPA	SPEDGTASKATTVQM	FGG	AF	YQ	VP	SPYV	LFTFV	QARSGG	SVL	LVSGDGV	DTLDSSSSSRGIDRTPIVGLSQIPASPEDGTASKATTVQMFGGAFYQVPSPYVLFTFVQARSGGSVLLVSGDGV	1	0.08
EU (2020)_MLG64	Europe	DT	PDSSSSSRGI	DR	TPIIGLSQIPA	SPEDGTASTATTVQM	FGG	AL	YQ	AT	SPYV	LFTFV	QARSGG	SVL	LVSGDGV	DTPDSSSSSRGIDRTPIIGLSQIPASPEDGTASTATTVQMFGGALYQATSPYVLFTFVQARSGGSVLLVSGDGV	1	0.08
EU (2020)_MLG65	Europe	DT	PDSSSSSRGI	DR	TPIVGLSQIPA	SPEDGTASTATTVQM	FGG	AF	YQ	AP	SPYV	LFTFV	QARSGG	SVL	LVSGGGV	DTPDSSSSSRGIDRTPIVGLSQIPASPEDGTASTATTVQMFGGAFYQAPSPYVLFTFVQARSGGSVLLVSGGGV	1	0.08
EU (2020)_MLG66	Europe	DT	PDSSSSSRGI	DR	TPIVGLSQIPA	SPEDGTASTATTVQM	YGG	AL	YQ	AP	SPYV	LFTFV	QARSGG	SVL	LVSGDGV	DTPDSSSSSRGIDRTPIVGLSQIPASPEDGTASTATTVQMYGGALYQAPSPYVLFTFVQARSGGSVLLVSGDGV	1	0.08
EU (2020)_MLG67	Europe	DT	PDSSSSSKRI	DR	TPIVGLSQIPA	SPEEGTASTATTVQM	FGG	AL	YQ	AL	SPYV	LFTFV	HARSGG	SVL	LGSGGGV	DTPDSSSSSKRIDRTPIVGLSQIPASPEEGTASTATTVQMFGGALYQALSPYVLFTFVHARSGGSVLLGSGGGV	1	0.08
EU (2020)_MLG68	Europe	DT	PDSSSSSRGI	DR	TPIVDLSQIPT	SPEDGTASTATTVQM	FGG	AL	YQ	AP	SPYV	LFTFV	QARSGG	SVL	LVGGDGV	DTPDSSSSSRGIDRTPIVDLSQIPTSPEDGTASTATTVQMFGGALYQAPSPYVLFTFVQARSGGSVLLVGGDGV	1	0.08
EU (2020)_MLG69	Europe	DT	PDSSSLSRGI	DR	TPIVGLSQIPA	SPEDGTASTATTVQM	FGG	AL	YQ	AP	SPYV	LFTFV	QARSVG	SVS	LVSGDGV	DTPDSSSLSRGIDRTPIVGLSQIPASPEDGTASTATTVQMFGGALYQAPSPYVLFTFVQARSVGSVSLVSGDGV	1	0.08
EU (2020)_MLG70	Europe	GT	PDSSSSSRGI	DR	TPIVGLSQIPA	SPEDGTASTATTVQM	FGG	AL	YQ	AS	SPYV	LFTFV	QARSGG	SVL	LVSGGGV	GTPDSSSSSRGIDRTPIVGLSQIPASPEDGTASTATTVQMFGGALYQASSPYVLFTFVQARSGGSVLLVSGGGV	1	0.08
EU (2020)_MLG71	Europe	DM	PDSSSSSKGI	DR	TPIVGLSQIPA	SPEDGTASTATTVQM	FGG	AL	YQ	AP	SPYV	LFTFV	QARSGG	SVL	LVSGGGV	DMPDSSSSSKGIDRTPIVGLSQIPASPEDGTASTATTVQMFGGALYQAPSPYVLFTFVQARSGGSVLLVSGGGV	1	0.08
EU (2020)_MLG72	Europe	DT	PDSSSSSRGI	DR	TPIVGLSQIPA	SPEDGTASTATTVQM	FGG	AL	YQ	AL	SPYV	LFTFV	QARSGV	SVL	LVSGGGV	DTPDSSSSSRGIDRTPIVGLSQIPASPEDGTASTATTVQMFGGALYQALSPYVLFTFVQARSGVSVLLVSGGGV	1	0.08
EU (2020)_MLG73	Europe	DT	PDSSSSSRGI	DR	TPIVGLSQIPA	SPEDGTASTATTVQM	YGG	AL	YQ	AP	SPYV	LFTFV	QARSGG	SVS	LVSGDGV	DTPDSSSSSRGIDRTPIVGLSQIPASPEDGTASTATTVQMYGGALYQAPSPYVLFTFVQARSGGSVSLVSGDGV	1	0.08
EU (2020)_MLG74	Europe	DT	PDSSSLSRGI	DR	TLIVGLSQIPA	SPEDGTASTATTVQM	YGG	AL	YQ	AP	SPYV	LFTFV	QARSVG	SVS	LVSGDGV	DTPDSSSLSRGIDRTLIVGLSQIPASPEDGTASTATTVQMYGGALYQAPSPYVLFTFVQARSVGSVSLVSGDGV	1	0.08
EU (2020)_MLG75	Europe	DT	PDSSSSSKRI	DR	TPIVGLSQIPA	SPEDGIASTATTVQM	FSG	AL	YQ	AL	SPYV	LFTFV	QARSGG	SVL	LGSGGGV	DTPDSSSSSKRIDRTPIVGLSQIPASPEDGIASTATTVQMFSGALYQALSPYVLFTFVQARSGGSVLLGSGGGV	1	0.08
EU (2020)_MLG76	Europe	DT	PDSSSSSRGI	DR	IPIVGLSQIPA	SPEDGTASTATTVQV	FGG	AL	YQ	AL	SPYV	LFTFV	HARSGG	SVL	LVSGGGV	DTPDSSSSSRGIDRIPIVGLSQIPASPEDGTASTATTVQVFGGALYQALSPYVLFTFVHARSGGSVLLVSGGGV	1	0.08
EU (2020)_MLG77	Europe	DT	PDSSSSSRGI	DR	IPIVGLSQIPA	SLEDGTASTATTVQM	FGG	AL	YQ	AL	SPYV	LFTFV	HARSGG	SVL	LVSGGGV	DTPDSSSSSRGIDRIPIVGLSQIPASLEDGTASTATTVQMFGGALYQALSPYVLFTFVHARSGGSVLLVSGGGV	1	0.08
EU (2020)_MLG78	Europe	DT	PDSSSSSRGI	DR	TPIVGLSQIPA	SPEDGIASTATTVQM	FSG	AL	YQ	AL	SPYV	LFTFV	QARSGG	SVL	LVSGGGV	DTPDSSSSSRGIDRTPIVGLSQIPASPEDGIASTATTVQMFSGALYQALSPYVLFTFVQARSGGSVLLVSGGGV	1	0.08
EU (2020)_MLG79	Europe	DT	PDSSSSSRGI	DR	TPIVGLSQIPA	SPEDGTASTATSVQM	FGG	AF	YQ	AP	SPYV	LFTFV	QARSGV	SVL	LVSGDGV	DTPDSSSSSRGIDRTPIVGLSQIPASPEDGTASTATSVQMFGGAFYQAPSPYVLFTFVQARSGVSVLLVSGDGV	1	0.08

EU (2020)_MLG80	Europe	DT	PDSSSSSRGI	DR	TPIVGLSQIPA	SPEDGTASTATTVHM	FGG	AL	YQ	AL	LPYV	LFTFV	HARSGG	SVL	LVSGGGV	DTPDSSSSSRGIDRTPIVGLSQIPASPEDGTASTATTVHMFGGALYQALLPYVLFTFVHARSGGSVLLVSGGGV	1	0.08
EU (2020)_MLG81	Europe	DT	PDSSSSSRGI	DR	TPIVDLSQIPT	SPEDGTASTATTVQM	FGG	AL	YQ	AL	SPYV	LFTFV	QARSGG	SVL	LVSGGGV	DTPDSSSSSRGIDRTPIVDLSQIPTSPEDGTASTATTVQMFGGALYQALSPYVLFTFVQARSGGSVLLVSGGGV	1	0.08
EU (2020)_MLG82	Europe	DT	PDSSSSSRGI	DR	TPIVGLSQIPA	SPEDGTASTATTVQM	FGG	AL	YQ	AP	SPYV	LFTFV	QARSVG	SVS	LVSGDGV	DTPDSSSSSRGIDRTPIVGLSQIPASPEDGTASTATTVQMFGGALYQAPSPYVLFTFVQARSVGSVSLVSGDGV	1	0.08
EU (2020)_MLG83	Europe	DT	PDSSSSSRGI	DR	TPIVGLSQIPA	SLEDGTASTATTVQM	FGG	AL	YQ	AL	SPYV	LFTFV	QARSGG	SVL	LVSGGGV	DTPDSSSSSRGIDRTPIVGLSQIPASLEDGTASTATTVQMFGGALYQALSPYVLFTFVQARSGGSVLLVSGGGV	1	0.08
EU (2020)_MLG84	Europe	DT	PDSSSSSRGI	DG	TPIVGLSQIPA	SPEDGTASTATTVQM	FGG	AL	YQ	AL	SPYV	LFTFV	QARSGG	SVL	LVSGGGV	DTPDSSSSSRGIDGTPIVGLSQIPASPEDGTASTATTVQMFGGALYQALSPYVLFTFVQARSGGSVLLVSGGGV	1	0.08
EU (2020)_MLG85	Europe	DT	PDSSSSSRGI	DC	TPIIGLSQIPA	SPEDGTASTATTVQI	FGG	AF	YQ	AP	SPYV	LFTFV	QARSGG	SVL	LVSGDGV	DTPDSSSSSRGIDCTPIIGLSQIPASPEDGTASTATTVQIFGGAFYQAPSPYVLFTFVQARSGGSVLLVSGDGV	1	0.08
EU (2020)_MLG86	Europe	DT	PDSSSSSKRI	DR	TPIVGLSQIPA	SPEDGTASTATTVQM	FGG	AL	YQ	AP	SPYV	LFTFV	QARSGG	SVL	LVSGGGV	DTPDSSSSSKRIDRTPIVGLSQIPASPEDGTASTATTVQMFGGALYQAPSPYVLFTFVQARSGGSVLLVSGGGV	1	0.08
EU (2020)_MLG87	Europe	DT	PDSSSSSRGI	DR	TPIVGLSQIPA	SPEDGTASTATTVQM	FGG	AL	YQ	AL	SPYV	LFTFV	HARSGG	SVL	LGSGGGV	DTPDSSSSSRGIDRTPIVGLSQIPASPEDGTASTATTVQMFGGALYQALSPYVLFTFVHARSGGSVLLGSGGGV	1	0.08
EU (2020)_MLG88	Europe	DT	PDSSSSSRGI	DR	TPIVDLSQIPA	SPEDGTASTATTVQM	FGG	AL	YQ	AP	SPYV	LFTFV	QARSGG	SVL	LVSGDGV	DTPDSSSSSRGIDRTPIVDLSQIPASPEDGTASTATTVQMFGGALYQAPSPYVLFTFVQARSGGSVLLVSGDGV	1	0.08
EU (2020)_MLG89	Europe	DT	PDSSSSSRGI	DR	TPIVGLSQIPA	SPEDGTASTATTVQM	FGG	AL	YQ	AL	SPYV	LFTFV	QARSGV	SVL	LGSGGGV	DTPDSSSSSRGIDRTPIVGLSQIPASPEDGTASTATTVQMFGGALYQALSPYVLFTFVQARSGVSVLLGSGGGV	1	0.08
EU (2020)_MLG90	Europe	DT	PDSSSSSRGT	DR	TPIVGLSQIPA	SPEDGTASTATTVQM	FGG	AL	YQ	AL	SPYV	LFTFV	QARSGG	SVL	LVSGGGV	DTPDSSSSSRGTDRTPIVGLSQIPASPEDGTASTATTVQMFGGALYQALSPYVLFTFVQARSGGSVLLVSGGGV	1	0.08
EU (2020)_MLG91	Europe	DT	PDSSSLSRGI	DR	TPIVGLSQIPA	SPEDGTASTATTVQM	FGG	AL	YQ	AP	SPYV	LFTFV	QARSVG	SVS	LFSGDVV	DTPDSSSLSRGIDRTPIVGLSQIPASPEDGTASTATTVQMFGGALYQAPSPYVLFTFVQARSVGSVSLFSGDVV	1	0.08
EU (2020)_MLG92	Europe	DT	PDSSSSSKGI	DR	TPIVGLSQIPA	SPEDGTASTATTVQM	FGG	AL	YQ	AL	SPYV	LFTFV	QARSGG	SVL	LVSGGGV	DTPDSSSSSKGIDRTPIVGLSQIPASPEDGTASTATTVQMFGGALYQALSPYVLFTFVQARSGGSVLLVSGGGV	1	0.08
EU (2020)_MLG93	Europe	DT	PDSSSSSKLI	DR	TPIVGLSQIPA	SPEDGTASTATTVQM	FGG	AL	YQ	AL	SPYV	LFTFV	QARSGG	SVL	LVSGGGV	DTPDSSSSSKLIDRTPIVGLSQIPASPEDGTASTATTVQMFGGALYQALSPYVLFTFVQARSGGSVLLVSGGGV	1	0.08
EU (2020)_MLG94	Europe	DT	PDSSSSSKRI	DR	TPIVGLSQIPA	SPEDGIASTATTVQM	FSG	AF	YQ	AL	SPYV	LFTFV	QARSGG	SVL	LVSGGGV	DTPDSSSSSKRIDRTPIVGLSQIPASPEDGIASTATTVQMFSGAFYQALSPYVLFTFVQARSGGSVLLVSGGGV	1	0.08
EU (2020)_MLG95	Europe	DT	PDSSSSSMGI	DR	TPIVGLSQIPA	SPEDGTASTATTVQM	FGG	AL	YQ	AL	SPYV	LFTFV	QARSGG	SVL	LVSGGGV	DTPDSSSSSMGIDRTPIVGLSQIPASPEDGTASTATTVQMFGGALYQALSPYVLFTFVQARSGGSVLLVSGGGV	1	0.08
EU (2020)_MLG96	Europe	DT	PDSSSSSKRI	DR	TPIVGLSQVPA	LPEDGTASTATTVQM	FGG	AL	YQ	AL	SPYV	LFTFV	QARSGG	SVL	LVSGGGV	DTPDSSSSSKRIDRTPIVGLSQVPALPEDGTASTATTVQMFGGALYQALSPYVLFTFVQARSGGSVLLVSGGGV	1	0.08
EU (2020)_MLG97	Europe	DT	PDSSLSSRGI	DR	TPIVGLSQVPA	SPEDGTASTATTVHM	FGG	AL	YQ	AL	SPYV	LFTFV	HARSGG	SVL	LVSGGGV	DTPDSSLSSRGIDRTPIVGLSQVPASPEDGTASTATTVHMFGGALYQALSPYVLFTFVHARSGGSVLLVSGGGV	1	0.08
EU (2021)_MLG98	Europe	DT	PDSSSSSKRI	DR	TPIVGLSQIDA	PEDGTASTATTVQM	FGG	AL	YQ	AL	SPYV	LFTFV	QARSGG	SVL	LVSGGGV	DTPDSSSSSKRIDRTPIVGLSQIDAPEDGTASTATTVQMFGGALYQALSPYVLFTFVQARSGGSVLLVSGGGV	4273	53.56
EU (2021)_MLG99	Europe	DT	PDSSSSSRGI	DR	TPIVGLSQIDA	PEDGTASTATTVQM	FGG	AL	YQ	AL	SPYV	LFTFV	QARSGG	SVL	LVSGGGV	DTPDSSSSSRGIDRTPIVGLSQIDAPEDGTASTATTVQMFGGALYQALSPYVLFTFVQARSGGSVLLVSGGGV	1314	16.47
EU (2021)_MLG100	Europe	DT	PDSSSSSRGI	DR	TPIVGLSQIDA	PEDGTASTATTVQM	FGG	AL	YQ	AL	SPYV	LFTFV	HARSGG	SVL	LVNGGGV	DTPDSSSSSRGIDRTPIVGLSQIDAPEDGTASTATTVQMFGGALYQALSPYVLFTFVHARSGGSVLLVNGGGV	460	5.77
EU (2021)_MLG101	Europe	DT	PDSSSSSKPI	DR	TPIVGLSQIDA	PEDGTASTATTVQM	FGG	AL	YQ	AL	SPYV	LFTFV	QARSGG	SVL	LVSGGGV	DTPDSSSSSKPIDRTPIVGLSQIDAPEDGTASTATTVQMFGGALYQALSPYVLFTFVQARSGGSVLLVSGGGV	272	3.41
EU (2021)_MLG102	Europe	DT	PDSSSSSRGI	DR	IPIVGLSQIDA	PEDGTASTATTVQM	FGG	AL	YQ	AL	SPYV	LFTFV	HARSGG	SVL	LVSGGGV	DTPDSSSSSRGIDRIPIVGLSQIDAPEDGTASTATTVQMFGGALYQALSPYVLFTFVHARSGGSVLLVSGGGV	260	3.26
EU (2021)_MLG103	Europe	DT	PDSSSSSKRI	DR	TPIVGLSQIDA	LEDGTASTATTVQM	FGG	AL	YQ	AL	SPYV	LFTFV	QARSGG	SVL	LVSGGGV	DTPDSSSSSKRIDRTPIVGLSQIDALEDGTASTATTVQMFGGALYQALSPYVLFTFVQARSGGSVLLVSGGGV	175	2.19
EU (2021)_MLG104	Europe	DT	PDSSSSSKRI	DR	TPIVGLSQIDA	PEDGTASTATTVQM	FGG	AL	YQ	AL	SPYV	LFTFV	QARSGG	SVL	LVSGGGF	DTPDSSSSSKRIDRTPIVGLSQIDAPEDGTASTATTVQMFGGALYQALSPYVLFTFVQARSGGSVLLVSGGGF	160	2.01
EU (2021)_MLG105	Europe	DT	PDSSSSSRGI	DR	TPIVGLSQIDA	PEDGTASTATTVQM	FGG	AF	YQ	AL	SPYV	LFTFV	QARSGG	SVL	LVSGGGV	DTPDSSSSSRGIDRTPIVGLSQIDAPEDGTASTATTVQMFGGAFYQALSPYVLFTFVQARSGGSVLLVSGGGV	63	0.79
EU (2021)_MLG106	Europe	DT	PDSSLSSRGI	DR	TPIVGLSQIDA	PEDGTASTATTVQM	FGG	AL	YQ	AL	SPYV	LFTFV	QARSGG	SVL	LVSGGGV	DTPDSSLSSRGIDRTPIVGLSQIDAPEDGTASTATTVQMFGGALYQALSPYVLFTFVQARSGGSVLLVSGGGV	61	0.76
EU (2021)_MLG107	Europe	DT	PDSSSSSKRI	DR	TPIVGLSQIDA	PEDGTASTATTVQM	FGG	AF	YQ	AL	SPYV	LFTFV	QARSGG	SVL	LVSGGGV	DTPDSSSSSKRIDRTPIVGLSQIDAPEDGTASTATTVQMFGGAFYQALSPYVLFTFVQARSGGSVLLVSGGGV	56	0.70
EU (2021)_MLG108	Europe	DT	PDSSSSSKRI	DR	TPIVGLSQIDA	PEDGTASTATTVQM	FGG	AL	YQ	AL	SPYV	LFTFV	QARSGG	SLL	LVSGGGV	DTPDSSSSSKRIDRTPIVGLSQIDAPEDGTASTATTVQMFGGALYQALSPYVLFTFVQARSGGSLLLVSGGGV	51	0.64
EU (2021)_MLG109	Europe	DT	PDSSSSNRGI	DR	TPIVGLSQIDA	PEDGTASTATTVQM	FGG	TL	YQ	AP	SPYV	LFTFV	QARSGG	SVS	LVSGDGV	DTPDSSSSNRGIDRTPIVGLSQIDAPEDGTASTATTVQMFGGTLYQAPSPYVLFTFVQARSGGSVSLVSGDGV	49	0.61
EU (2021)_MLG110	Europe	DT	PDSSSSSKRI	DC	TPIVGLSQIDA	PEDGTASTATTVQM	FGG	AL	YQ	AL	SPYV	LFTFV	QARSGG	SVL	LVSGGGV	DTPDSSSSSKRIDCTPIVGLSQIDAPEDGTASTATTVQMFGGALYQALSPYVLFTFVQARSGGSVLLVSGGGV	42	0.53
EU (2021)_MLG111	Europe	DT	PDSSSSNRGI	DR	TPIVGLSQIDA	PEDGTASTATTVQM	FGG	AL	YQ	AP	SPYV	LFTFV	QARSGG	SVS	LVSGDGV	DTPDSSSSNRGIDRTPIVGLSQIDAPEDGTASTATTVQMFGGALYQAPSPYVLFTFVQARSGGSVSLVSGDGV	31	0.39
EU (2021)_MLG112	Europe	DT	PDSLSSSKRI	DR	TPIVGLSQIDA	PEDGTASTATTVQM	FGG	AL	YQ	AL	SPYV	LFTFV	QARSGG	SVL	LVSGGGV	DTPDSLSSSKRIDRTPIVGLSQIDAPEDGTASTATTVQMFGGALYQALSPYVLFTFVQARSGGSVLLVSGGGV	28	0.35
EU (2021)_MLG113	Europe	DT	PDSSSSSKRI	DR	TPIVGLSQIDA	PEDGIASTATTVQM	FSG	AL	YQ	AL	SPYV	LFTFV	QARSGG	SVL	LVSGGGV	DTPDSSSSSKRIDRTPIVGLSQIDAPEDGIASTATTVQMFSGALYQALSPYVLFTFVQARSGGSVLLVSGGGV	27	0.34
EU (2021)_MLG114	Europe	DT	SDSSSTSKRI	DR	TPIVGLSQIDA	PEDGTASTATTVQM	FGG	AL	YQ	AL	SPYV	LFTFV	QARSGG	SVL	LVSGGGV	DTSDSSSTSKRIDRTPIVGLSQIDAPEDGTASTATTVQMFGGALYQALSPYVLFTFVQARSGGSVLLVSGGGV	23	0.29
EU (2021)_MLG115	Europe	DT	PDSSSSSRGI	DR	TPIVGLSQIDA	PEDGTASTATTVQM	FGG	AL	YQ	AL	SPYV	LFTFV	QARSGG	SVL	LVSGGGF	DTPDSSSSSRGIDRTPIVGLSQIDAPEDGTASTATTVQMFGGALYQALSPYVLFTFVQARSGGSVLLVSGGGF	21	0.26
EU (2021)_MLG116	Europe	DT	PDSSSSSKRI	DR	TPIVGLSQIDA	PEDGTASTATTVQM	FGG	AL	YQ	AL	SPYV	LFTFL	QARSGG	SVL	LVSGGGV	DTPDSSSSSKRIDRTPIVGLSQIDAPEDGTASTATTVQMFGGALYQALSPYVLFTFLQARSGGSVLLVSGGGV	20	0.25
EU (2021)_MLG117	Europe	DT	PDSSSSSRGI	DR	TPIVGLSQIDA	PEDGTASTATTVQM	FGG	AL	YQ	AL	SPYV	LFTFV	QARFGG	SVL	LVSGGGV	DTPDSSSSSRGIDRTPIVGLSQIDAPEDGTASTATTVQMFGGALYQALSPYVLFTFVQARFGGSVLLVSGGGV	17	0.21
EU (2021)_MLG118	Europe	DT	PDSSSSSKLI	DR	TPIVGLSQIDA	PEDGTASTATTVQM	FGG	AL	YQ	AL	SPYV	LFTFV	QARSGG	SVL	LVSGGGV	DTPDSSSSSKLIDRTPIVGLSQIDAPEDGTASTATTVQMFGGALYQALSPYVLFTFVQARSGGSVLLVSGGGV	17	0.21
EU (2021)_MLG119	Europe	DT	PDSSSSSKRI	DR	TPIVGLSQIDA	PEDGTASTATTVQM	FGG	AL	YQ	AL	SPYV	FFTFV	QARSGG	SVL	LVSGGGV	DTPDSSSSSKRIDRTPIVGLSQIDAPEDGTASTATTVQMFGGALYQALSPYVFFTFVQARSGGSVLLVSGGGV	17	0.21
EU (2021)_MLG120	Europe	DT	PDSSLSSRGI	DR	TPIVGLSQIDA	PEDGTASTATTVQM	FGG	AL	YQ	AL	SPYV	LFTFV	HARSGG	SVL	LVSGGGV	DTPDSSLSSRGIDRTPIVGLSQIDAPEDGTASTATTVQMFGGALYQALSPYVLFTFVHARSGGSVLLVSGGGV	16	0.20
EU (2021)_MLG121	Europe	DT	PDSSSSSKRI	DR	TPIVGLSQIDA	PEDGTASTATTVQM	FGG	AL	YQ	AL	SPYV	LFTFV	HARSGG	SVL	LVSGGGV	DTPDSSSSSKRIDRTPIVGLSQIDAPEDGTASTATTVQMFGGALYQALSPYVLFTFVHARSGGSVLLVSGGGV	16	0.20
EU (2021)_MLG122	Europe	DT	PDSSSSSKRI	DR	TPIVGLSQIDA	PEDGTASTATTVQM	FGG	AL	YQ	AL	SPYV	LFTFV	QARSGG	SVL	LVNGGGV	DTPDSSSSSKRIDRTPIVGLSQIDAPEDGTASTATTVQMFGGALYQALSPYVLFTFVQARSGGSVLLVNGGGV	16	0.20
EU (2021)_MLG123	Europe	DT	PDSSSSSRGI	DR	TPIVGLSHIDA	PEDGTASTATTVQM	FGG	AL	YQ	AL	SPYV	LFTFV	QARSGG	SVL	LVSGGGV	DTPDSSSSSRGIDRTPIVGLSHIDAPEDGTASTATTVQMFGGALYQALSPYVLFTFVQARSGGSVLLVSGGGV	15	0.19
EU (2021)_MLG124	Europe	DT	PDSSSSSRGI	DR	TPIVGLSQIDA	PEDGTASTATTVQM	FGG	AF	YQ	AL	SPYV	LFTFV	HARSGG	SVL	LVNGGGV	DTPDSSSSSRGIDRTPIVGLSQIDAPEDGTASTATTVQMFGGAFYQALSPYVLFTFVHARSGGSVLLVNGGGV	14	0.18
EU (2021)_MLG125	Europe	DT	PDSSSSSRGI	DR	TPIVGLSQIDA	PEDGTASTATTVQM	FGG	AL	YQ	AF	SPYV	LFTFV	QARSGG	SVL	LVSGGGV	DTPDSSSSSRGIDRTPIVGLSQIDAPEDGTASTATTVQMFGGALYQAFSPYVLFTFVQARSGGSVLLVSGGGV	14	0.18
EU (2021)_MLG126	Europe	DT	PDSSSSSKRI	DR	TPIVGLSQIDA	PEDGTASTATTVQM	FGG	AL	YQ	AL	SPYV	LFTFV	QARSGS	SVL	LVSGGGV	DTPDSSSSSKRIDRTPIVGLSQIDAPEDGTASTATTVQMFGGALYQALSPYVLFTFVQARSGSSVLLVSGGGV	14	0.18
EU (2021)_MLG127	Europe	DT	PDSSSSSRGI	DC	TPIVGLSQIDA	PEDGTASTATTVQM	FGG	AL	YQ	AL	SPYV	LFTFV	QARSGG	SVL	LVSGGGV	DTPDSSSSSRGIDCTPIVGLSQIDAPEDGTASTATTVQMFGGALYQALSPYVLFTFVQARSGGSVLLVSGGGV	13	0.16
EU (2021)_MLG128	Europe	DT	PDSSSSSRGI	DR	TPIVGLSQIDA	PEDGTASTATTVQM	FGG	AL	YQ	AL	SPYV	LFTFV	HARSGG	SVL	LVSGGGV	DTPDSSSSSRGIDRTPIVGLSQIDAPEDGTASTATTVQMFGGALYQALSPYVLFTFVHARSGGSVLLVSGGGV	13	0.16
EU (2021)_MLG129	Europe	DT	PDSSSSSRGI	DR	IPIVGLSQIDA	PEDGTASTATTVQM	FGG	AL	YQ	AL	SPYV	LFTFV	HARSGS	SVL	LVSGGGV	DTPDSSSSSRGIDRIPIVGLSQIDAPEDGTASTATTVQMFGGALYQALSPYVLFTFVHARSGSSVLLVSGGGV	11	0.14
EU (2021)_MLG130	Europe	DT	TDSSSSSRGI	DR	TPIVGLSQIDA	PEDGTASTATTVQM	FGG	AL	YQ	AL	SPYV	LFTFV	HARSGG	SVL	LVNGGGV	DTTDSSSSSRGIDRTPIVGLSQIDAPEDGTASTATTVQMFGGALYQALSPYVLFTFVHARSGGSVLLVNGGGV	9	0.11
EU (2021)_MLG131	Europe	DT	PDSLSSSRGI	DR	TPIVGLSQIDA	PEDGTASTATTVQM	FGG	AL	YQ	AL	SPYV	LFTFV	QARSGG	SVL	LVSGGGV	DTPDSLSSSRGIDRTPIVGLSQIDAPEDGTASTATTVQMFGGALYQALSPYVLFTFVQARSGGSVLLVSGGGV	9	0.11
EU (2021)_MLG132	Europe	DT	PDSSSSNRGI	DR	TPIVGLSQIDA	PEDGTASTATTVQM	FGG	AL	YQ	AP	SPYV	LFTFV	QSRSGG	SVS	LVSGNGV	DTPDSSSSNRGIDRTPIVGLSQIDAPEDGTASTATTVQMFGGALYQAPSPYVLFTFVQSRSGGSVSLVSGNGV	9	0.11
EU (2021)_MLG133	Europe	DT	PDSLSSSRGI	DR	TPIVGFSQIDA	PEDGTASTATTVQM	FGG	AL	YQ	AL	SPYV	LFTFV	QARFGG	SVL	LVSGGGV	DTPDSLSSSRGIDRTPIVGFSQIDAPEDGTASTATTVQMFGGALYQALSPYVLFTFVQARFGGSVLLVSGGGV	9	0.11
EU (2021)_MLG134	Europe	DT	PDSSSSSKRI	DR	TPIVGLSQIDA	PEEGTASTATTVQM	FGG	AF	YQ	AL	SPYV	LFTFV	QARSGG	SVL	LVSGGGV	DTPDSSSSSKRIDRTPIVGLSQIDAPEEGTASTATTVQMFGGAFYQALSPYVLFTFVQARSGGSVLLVSGGGV	9	0.11
EU (2021)_MLG135	Europe	DT	PDSSSSSRGI	DR	TPIVGLSQIDA	PEDGTVSTATTVQM	FGG	AL	YQ	AL	SPYV	LFTFV	QARSGG	SVL	LVSGGGV	DTPDSSSSSRGIDRTPIVGLSQIDAPEDGTVSTATTVQMFGGALYQALSPYVLFTFVQARSGGSVLLVSGGGV	8	0.10
EU (2021)_MLG136	Europe	DT	PDSLSSSRGI	DR	TPIVGLSQIDA	PEDGTASTATTVQM	FGG	AL	YQ	AL	SPYV	LFTFV	QARSGG	SVL	LVNGGGV	DTPDSLSSSRGIDRTPIVGLSQIDAPEDGTASTATTVQMFGGALYQALSPYVLFTFVQARSGGSVLLVNGGGV	8	0.10
EU (2021)_MLG137	Europe	DT	PDSSSSSKRI	DR	TPIVGLSQIDA	PEDGTASTATTVQM	FGG	AL	YQ	AL	SPYV	LFTFV	QARSGG	SVL	FVSGGGV	DTPDSSSSSKRIDRTPIVGLSQIDAPEDGTASTATTVQMFGGALYQALSPYVLFTFVQARSGGSVLFVSGGGV	8	0.10
EU (2021)_MLG138	Europe	DT	PDSSSSSKRI	DR	TPIVGLSQIDA	PEDGTASTATTVQM	FGS	AL	YQ	AL	SPYV	LFTFV	QARSGG	SVL	LVSGGGV	DTPDSSSSSKRIDRTPIVGLSQIDAPEDGTASTATTVQMFGSALYQALSPYVLFTFVQARSGGSVLLVSGGGV	8	0.10
EU (2021)_MLG139	Europe	DT	PDSSSSSKRI	DR	TPIVGLSQIDA	PEDGTASTATTVQM	FGG	AL	YQ	AL	SSYV	LFTFV	QARSGG	SVL	LVSGGGV	DTPDSSSSSKRIDRTPIVGLSQIDAPEDGTASTATTVQMFGGALYQALSSYVLFTFVQARSGGSVLLVSGGGV	8	0.10
EU (2021)_MLG140	Europe	DT	PDSSLSSRGI	DR	TPIVGLSQIDA	PEDGTASTATTVQM	FGG	AF	YQ	AL	SPYV	LFTFV	QARSGG	SVL	LVSGGGV	DTPDSSLSSRGIDRTPIVGLSQIDAPEDGTASTATTVQMFGGAFYQALSPYVLFTFVQARSGGSVLLVSGGGV	7	0.09
EU (2021)_MLG141	Europe	DT	PDSSSSSRGI	DR	TPIVGLSQIDA	PEDGTASTATTVQM	FGG	AL	YQ	AL	SPYV	LFTFV	QSRSGG	SVL	LVSGGGV	DTPDSSSSSRGIDRTPIVGLSQIDAPEDGTASTATTVQMFGGALYQALSPYVLFTFVQSRSGGSVLLVSGGGV	7	0.09
EU (2021)_MLG142	Europe	DT	PDSSSSSKRI	DR	TPIVGLSQIDA	PEDGTASTATTVQM	FGG	AL	YQ	AL	SPYV	LFTFI	QARSGG	SVL	LVSGGGV	DTPDSSSSSKRIDRTPIVGLSQIDAPEDGTASTATTVQMFGGALYQALSPYVLFTFIQARSGGSVLLVSGGGV	7	0.09
EU (2021)_MLG143	Europe	DT	PDSSSSSRGI	DR	TPIVGLSQIDA	PEDGTASTATTVQM	FGG	AL	YQ	AL	STYV	LFTFV	QARSGG	SVL	LVSGGGV	DTPDSSSSSRGIDRTPIVGLSQIDAPEDGTASTATTVQMFGGALYQALSTYVLFTFVQARSGGSVLLVSGGGV	6	0.08

EU (2021)_MLG144	Europe	DT	SDSSSSSRGI	DR	IPIVGLSQIDA	PEDGTASTATTVQM	FGG	AL	YQ	AL	SPYV	LFTFV	HARSGG	SVL	LVSGGGV	DTSDSSSSSRGIDRIPIVGLSQIDAPEDGTASTATTVQMFGGALYQALSPYVLFTFVHARSGGSVLLVSGGGV	6	0.08
EU (2021)_MLG145	Europe	DT	PDSSSSSRGI	DR	IPIVGLSQIDA	PEDGTASTATTVQM	FGG	AL	YQ	AL	SPYV	LFTFV	HARSGG	LVL	LVSGGGV	DTPDSSSSSRGIDRIPIVGLSQIDAPEDGTASTATTVQMFGGALYQALSPYVLFTFVHARSGGLVLLVSGGGV	6	0.08
EU (2021)_MLG146	Europe	DT	PDSSSSSEPI	DR	TPIVGLSQIDA	PEDGTASTATTVQM	FGG	AL	YQ	AL	SPYV	LFTFV	QARSGG	SVL	LVSGGGV	DTPDSSSSSEPIDRTPIVGLSQIDAPEDGTASTATTVQMFGGALYQALSPYVLFTFVQARSGGSVLLVSGGGV	6	0.08
EU (2021)_MLG147	Europe	DT	PDSSSSSKRI	DR	TPIVGLSQIDA	SEDGTASTATTVQM	FGG	AL	YQ	AL	SPYV	LFTFV	QARSGG	SVL	LVSGGGV	DTPDSSSSSKRIDRTPIVGLSQIDASEDGTASTATTVQMFGGALYQALSPYVLFTFVQARSGGSVLLVSGGGV	6	0.08
EU (2021)_MLG148	Europe	DT	PDSSSSSRGI	DR	IPIVGLSQIDA	PEDGTASTATTVQM	FGG	AL	YQ	AH	SPYV	LFTFV	HARSGG	SVL	LVSGGGV	DTPDSSSSSRGIDRIPIVGLSQIDAPEDGTASTATTVQMFGGALYQAHSPYVLFTFVHARSGGSVLLVSGGGV	5	0.06
EU (2021)_MLG149	Europe	DT	PDSSSSSRGI	DR	TPIVGLSQIDA	PEDGIASTATTVQM	FGG	AL	YQ	AL	SPYV	LFTFV	QARSGG	SVL	LVSGGGV	DTPDSSSSSRGIDRTPIVGLSQIDAPEDGIASTATTVQMFGGALYQALSPYVLFTFVQARSGGSVLLVSGGGV	5	0.06
EU (2021)_MLG150	Europe	DT	PDSSSSNRGI	DR	TPIVGLSQIDA	PEDGTASTATTVQM	FGG	TF	YQ	AP	SPYV	LFTFV	QARSGG	SVS	LVSGDGV	DTPDSSSSNRGIDRTPIVGLSQIDAPEDGTASTATTVQMFGGTFYQAPSPYVLFTFVQARSGGSVSLVSGDGV	5	0.06
EU (2021)_MLG151	Europe	DT	LDSSSSSRGI	DR	TPIVGLSQIDA	PEDGTASTATTVQM	FGG	AL	YQ	AL	SPYV	LFTFV	QARSGG	SVL	LVSGGGV	DTLDSSSSSRGIDRTPIVGLSQIDAPEDGTASTATTVQMFGGALYQALSPYVLFTFVQARSGGSVLLVSGGGV	5	0.06
EU (2021)_MLG152	Europe	DT	PDSSSSSRGI	DR	TPIVGLSQIDA	PEDGTASTATTVQM	FGG	AL	YQ	AL	SPYV	LFTFV	QVRSGG	SVL	LVSGGGV	DTPDSSSSSRGIDRTPIVGLSQIDAPEDGTASTATTVQMFGGALYQALSPYVLFTFVQVRSGGSVLLVSGGGV	5	0.06
EU (2021)_MLG153	Europe	DT	PDSSSSSRGI	DR	TPIVGLSQIDA	PEDGTASTATTVQM	FGG	AL	YQ	AP	SPYV	LFTFV	QARSGG	SVL	LVSGGGV	DTPDSSSSSRGIDRTPIVGLSQIDAPEDGTASTATTVQMFGGALYQAPSPYVLFTFVQARSGGSVLLVSGGGV	5	0.06
EU (2021)_MLG154	Europe	DT	PDSSLSSRGI	DR	TPIVGLSQIDA	PEDGTASTATTVQM	FGG	AL	YQ	AL	SPYV	LFTFV	QARSGG	SVL	LVSGGGF	DTPDSSLSSRGIDRTPIVGLSQIDAPEDGTASTATTVQMFGGALYQALSPYVLFTFVQARSGGSVLLVSGGGF	5	0.06
EU (2021)_MLG155	Europe	DT	PDSSSSSRGI	DR	IPIVGLSQIDA	PEDGTASTATTVQM	FGG	AF	YQ	AL	SPYV	LFTFV	HARSGG	SVL	LVSGGGV	DTPDSSSSSRGIDRIPIVGLSQIDAPEDGTASTATTVQMFGGAFYQALSPYVLFTFVHARSGGSVLLVSGGGV	5	0.06
EU (2021)_MLG156	Europe	DT	PDSLSSSRGI	DR	TPIVGLSQIDA	PEDGTASTATTVQM	FGG	AL	YQ	AL	SPYV	LFTFV	HARSGG	SVL	LVSGGGV	DTPDSLSSSRGIDRTPIVGLSQIDAPEDGTASTATTVQMFGGALYQALSPYVLFTFVHARSGGSVLLVSGGGV	4	0.05
EU (2021)_MLG157	Europe	DT	LDSSSSSRGI	DR	IPIVGLSQIDA	PEDGTASTATTVQM	FGG	AL	YQ	AL	SPYV	LFTFV	HARSGG	SVL	LVSGGGV	DTLDSSSSSRGIDRIPIVGLSQIDAPEDGTASTATTVQMFGGALYQALSPYVLFTFVHARSGGSVLLVSGGGV	4	0.05
EU (2021)_MLG158	Europe	DT	PDSSSSSRGI	DR	TPIVGLSQIDA	PEDGTASTATTVQM	FGG	AL	YQ	AL	SPYV	LFTFV	QARSGG	SVL	LVIGGGV	DTPDSSSSSRGIDRTPIVGLSQIDAPEDGTASTATTVQMFGGALYQALSPYVLFTFVQARSGGSVLLVIGGGV	4	0.05
EU (2021)_MLG159	Europe	DT	PDSSSSSRGI	DH	TPIVGLSQIDA	PEDGTASTATTVQM	FGG	AL	YQ	AL	SPYV	LFTFV	QARSGG	SVL	LVSGGGV	DTPDSSSSSRGIDHTPIVGLSQIDAPEDGTASTATTVQMFGGALYQALSPYVLFTFVQARSGGSVLLVSGGGV	4	0.05
EU (2021)_MLG160	Europe	DT	PDSSSSSRGI	DR	IPIVGLSQIDA	PEDGTASTATTVQM	FGG	AL	YQ	AL	SPYV	LFTFV	QARSGG	SVL	LVSGGGV	DTPDSSSSSRGIDRIPIVGLSQIDAPEDGTASTATTVQMFGGALYQALSPYVLFTFVQARSGGSVLLVSGGGV	4	0.05
EU (2021)_MLG161	Europe	DT	PDSSSSSKPI	DR	TPIVGLSQIDA	PEDGTASTATTVQM	FGG	AF	YQ	AL	SPYV	LFTFV	QARSGG	SVL	LVSGGGV	DTPDSSSSSKPIDRTPIVGLSQIDAPEDGTASTATTVQMFGGAFYQALSPYVLFTFVQARSGGSVLLVSGGGV	4	0.05
EU (2021)_MLG162	Europe	DT	PDSSSSSKRI	DR	TPIVGLSQIDA	PEDGTASTATTVQM	FGG	AL	YQ	AP	SPYV	LFTFV	QARSGG	SVL	LVSGGGV	DTPDSSSSSKRIDRTPIVGLSQIDAPEDGTASTATTVQMFGGALYQAPSPYVLFTFVQARSGGSVLLVSGGGV	4	0.05
EU (2021)_MLG163	Europe	DT	PDSSSSSKRI	DR	TPIVGLSQIDA	PEDGTASTATTVQM	FGG	AL	YQ	AL	SPYV	LFTFV	QARSGG	SAL	LVSGGGV	DTPDSSSSSKRIDRTPIVGLSQIDAPEDGTASTATTVQMFGGALYQALSPYVLFTFVQARSGGSALLVSGGGV	4	0.05
EU (2021)_MLG164	Europe	DT	PDSSSSSRGI	DR	TPIVGLSQIDA	PEDGTASTATTVQM	FGG	AL	YQ	AL	SSYV	LFTFV	HARSGG	SVL	LVSGGGV	DTPDSSSSSRGIDRTPIVGLSQIDAPEDGTASTATTVQMFGGALYQALSSYVLFTFVHARSGGSVLLVSGGGV	3	0.04
EU (2021)_MLG165	Europe	DT	PDSSSSSRGI	DR	TPIVGLSQIDA	PEDGTASTATTVQM	FGG	AL	YQ	VL	SPYV	LFTFV	QARSGG	SVL	LVSGGGV	DTPDSSSSSRGIDRTPIVGLSQIDAPEDGTASTATTVQMFGGALYQVLSPYVLFTFVQARSGGSVLLVSGGGV	3	0.04
EU (2021)_MLG166	Europe	DT	PDSSSSSRGI	DR	TPIVGLSQIDA	PEDGTASTVTTVQM	FGG	AL	YQ	AL	SPYV	LFTFV	QARSGG	SVL	LVSGGGV	DTPDSSSSSRGIDRTPIVGLSQIDAPEDGTASTVTTVQMFGGALYQALSPYVLFTFVQARSGGSVLLVSGGGV	3	0.04
EU (2021)_MLG167	Europe	DT	PDSSSSSRGI	DR	TPIVGLSQIDA	PEDGTASTATTVQM	FGG	AL	YQ	AL	SPYV	LFTFV	QARSGG	SVL	LVSGGVV	DTPDSSSSSRGIDRTPIVGLSQIDAPEDGTASTATTVQMFGGALYQALSPYVLFTFVQARSGGSVLLVSGGVV	3	0.04
EU (2021)_MLG168	Europe	DT	TDSSSSSRGI	DR	TPIVGLSQIDA	PEDGTASTATTVQM	FGG	AL	YQ	AL	SPYV	LFTFV	QARSGG	SVL	LVSGGGV	DTTDSSSSSRGIDRTPIVGLSQIDAPEDGTASTATTVQMFGGALYQALSPYVLFTFVQARSGGSVLLVSGGGV	3	0.04
EU (2021)_MLG169	Europe	DT	PDSSSSSRGI	DR	TPIVGLSQIDA	SEDGTASTATTVQM	FGG	AL	YQ	AL	SPYV	LFTFV	QARSGG	SVL	LVSGGGV	DTPDSSSSSRGIDRTPIVGLSQIDASEDGTASTATTVQMFGGALYQALSPYVLFTFVQARSGGSVLLVSGGGV	3	0.04
EU (2021)_MLG170	Europe	DT	PDSSSSSRGI	DR	TPIVGLSQIDA	PEDGTASTATTVQM	FGG	AL	YQ	AP	SPYV	LFTFV	QARSGG	SVL	LVSGDGV	DTPDSSSSSRGIDRTPIVGLSQIDAPEDGTASTATTVQMFGGALYQAPSPYVLFTFVQARSGGSVLLVSGDGV	3	0.04
EU (2021)_MLG171	Europe	DT	PDSSSSSKPI	DR	TPIVGLSQIDA	PEDGTASTATTVQM	FGG	AL	YQ	AL	SPYV	LFTFV	QARSGG	SVL	LVRGGGV	DTPDSSSSSKPIDRTPIVGLSQIDAPEDGTASTATTVQMFGGALYQALSPYVLFTFVQARSGGSVLLVRGGGV	3	0.04
EU (2021)_MLG172	Europe	DT	PDSSSSSKRI	DR	TPIVGLSQIDA	PEDGTASTATTVQM	FGG	AL	YQ	AL	SPYV	LFTFV	QARFGG	SVL	LVSGGGV	DTPDSSSSSKRIDRTPIVGLSQIDAPEDGTASTATTVQMFGGALYQALSPYVLFTFVQARFGGSVLLVSGGGV	3	0.04
EU (2021)_MLG173	Europe	DT	PDSSSSSKRI	DR	TPIVGLSQIDA	PEDGTASTATTVQI	FGG	AL	YQ	AL	SPYV	LFTFV	QARSGG	SVL	LVSGGGV	DTPDSSSSSKRIDRTPIVGLSQIDAPEDGTASTATTVQIFGGALYQALSPYVLFTFVQARSGGSVLLVSGGGV	3	0.04
EU (2021)_MLG174	Europe	DT	PDSSSSSKRI	DR	TPIVGLSQIDA	PEDGTASTATTVQM	FGG	AL	YQ	AL	SPYV	FFTFI	QARSGG	SVL	LVSGGGV	DTPDSSSSSKRIDRTPIVGLSQIDAPEDGTASTATTVQMFGGALYQALSPYVFFTFIQARSGGSVLLVSGGGV	3	0.04
EU (2021)_MLG175	Europe	DT	PDSSSSSRGI	DR	TPIVGLSQIDA	PEDGTASTATTVQM	FGG	AF	YQ	AL	SPYV	LFTFV	QARFGG	SVL	LVSGGGV	DTPDSSSSSRGIDRTPIVGLSQIDAPEDGTASTATTVQMFGGAFYQALSPYVLFTFVQARFGGSVLLVSGGGV	2	0.03
EU (2021)_MLG176	Europe	DT	PDSSSLSRGI	DR	TPIVGLSQIDA	PEDGTASTATTVQM	YGG	AL	YQ	AP	SPYV	LFTFV	QARSVG	SVS	LVSGGGV	DTPDSSSLSRGIDRTPIVGLSQIDAPEDGTASTATTVQMYGGALYQAPSPYVLFTFVQARSVGSVSLVSGGGV	2	0.03
EU (2021)_MLG177	Europe	DT	PDSSSSSRGI	DR	TPIVGLSQIDA	PEDGTASTATTVQM	FGG	AL	YQ	AL	SLYV	LFTFV	QARSGG	SVL	LVSGGGV	DTPDSSSSSRGIDRTPIVGLSQIDAPEDGTASTATTVQMFGGALYQALSLYVLFTFVQARSGGSVLLVSGGGV	2	0.03
EU (2021)_MLG178	Europe	DT	PDSSSSSRGI	DR	TPIVGLSQIDA	LEDGTASTATTVQM	FGG	AL	YQ	AL	SPYV	LFTFV	QARSGG	SVL	LVSGGGF	DTPDSSSSSRGIDRTPIVGLSQIDALEDGTASTATTVQMFGGALYQALSPYVLFTFVQARSGGSVLLVSGGGF	2	0.03
EU (2021)_MLG179	Europe	DT	SDSSSSSRGI	DR	TPIVGLSQIDA	PEDGTASTATTVQM	FGG	AL	YQ	AL	SPYV	LFTFV	QARSGG	SVL	LVSGGGV	DTSDSSSSSRGIDRTPIVGLSQIDAPEDGTASTATTVQMFGGALYQALSPYVLFTFVQARSGGSVLLVSGGGV	2	0.03
EU (2021)_MLG180	Europe	DT	PDSSSSSRGI	DR	TPIVGLSQIDA	PEDGTASTATTVQM	FGG	AL	YQ	AL	SPYV	LFTFV	QARSGG	SVL	LFSGGGV	DTPDSSSSSRGIDRTPIVGLSQIDAPEDGTASTATTVQMFGGALYQALSPYVLFTFVQARSGGSVLLFSGGGV	2	0.03
EU (2021)_MLG181	Europe	DT	PDSSSSSRGI	DR	TPIVGLSQIDA	LEDGTASTATTVQM	FGG	AL	YQ	AL	SPYV	LFTFV	QARSGG	SVL	LVSGGGV	DTPDSSSSSRGIDRTPIVGLSQIDALEDGTASTATTVQMFGGALYQALSPYVLFTFVQARSGGSVLLVSGGGV	2	0.03
EU (2021)_MLG182	Europe	DT	PDSSSSKRRGI	DR	TPIVGLSQIDA	LEDGTASTATTVQM	FGG	AL	YQ	AL	SPYV	LFTFV	QARSGG	SVL	LVSGGGV	DTPDSSSSKRRGIDRTPIVGLSQIDALEDGTASTATTVQMFGGALYQALSPYVLFTFVQARSGGSVLLVSGGGV	2	0.03
EU (2021)_MLG183	Europe	DT	PDSSSSNRGI	DR	TPIVGLSQIDA	PEDGTASTATTVQM	FGG	AF	YQ	AP	SPYV	LFTFV	QARSGG	SVS	LVSGDGV	DTPDSSSSNRGIDRTPIVGLSQIDAPEDGTASTATTVQMFGGAFYQAPSPYVLFTFVQARSGGSVSLVSGDGV	2	0.03
EU (2021)_MLG184	Europe	DT	PDSSSPSRGI	DR	TPIVGLSQIDA	PEDGTASTATTVQM	FGG	AL	YQ	AL	SPYV	LFTFV	QARSGG	SVL	LVSGGGV	DTPDSSSPSRGIDRTPIVGLSQIDAPEDGTASTATTVQMFGGALYQALSPYVLFTFVQARSGGSVLLVSGGGV	2	0.03
EU (2021)_MLG185	Europe	DT	PDSSSSSRGI	DR	IPIVGLSQIDA	PEDGTASTATTVQM	FGG	AL	YQ	AL	SPYV	LFTFV	HARSGG	SVL	LVNGGGV	DTPDSSSSSRGIDRIPIVGLSQIDAPEDGTASTATTVQMFGGALYQALSPYVLFTFVHARSGGSVLLVNGGGV	2	0.03
EU (2021)_MLG186	Europe	DT	PDSSSSSRGI	DR	TPIVGLSQIDA	PEDGTASIATTVQM	FGG	AL	YQ	AL	SPYV	LFTFV	QARSGG	SVL	LVSGGGV	DTPDSSSSSRGIDRTPIVGLSQIDAPEDGTASIATTVQMFGGALYQALSPYVLFTFVQARSGGSVLLVSGGGV	2	0.03
EU (2021)_MLG187	Europe	DT	PDSSSSSRGI	DR	TPIVGLSQIDA	PEDGTASTATTVQM	FGG	AL	YQ	AP	SPYV	LFTFV	QARSGG	SLL	LVSGGGV	DTPDSSSSSRGIDRTPIVGLSQIDAPEDGTASTATTVQMFGGALYQAPSPYVLFTFVQARSGGSLLLVSGGGV	2	0.03
EU (2021)_MLG188	Europe	DT	PDSSSSIRGI	DR	TPIVGLSQIDA	PEDGTASTATTVQM	FGG	AL	YQ	AL	SPYV	LFTFV	QARSGG	SVL	LVSGDGV	DTPDSSSSIRGIDRTPIVGLSQIDAPEDGTASTATTVQMFGGALYQALSPYVLFTFVQARSGGSVLLVSGDGV	2	0.03
EU (2021)_MLG189	Europe	DT	PDSSLSSRGI	DR	TPIVGLSQIDA	PEDGTASTATTVQM	FGG	AF	YQ	AL	SPYV	LFTFV	HARSGG	SVL	LVSGGGV	DTPDSSLSSRGIDRTPIVGLSQIDAPEDGTASTATTVQMFGGAFYQALSPYVLFTFVHARSGGSVLLVSGGGV	2	0.03
EU (2021)_MLG190	Europe	DT	PDSSSSNRGI	DR	TPIVGLSQIDA	PEDGTASTATTVQM	FGG	AL	YQ	AP	SPYV	LFTFV	QARSGG	SVS	LVSGGGV	DTPDSSSSNRGIDRTPIVGLSQIDAPEDGTASTATTVQMFGGALYQAPSPYVLFTFVQARSGGSVSLVSGGGV	2	0.03
EU (2021)_MLG191	Europe	DT	PDSSSSSELI	DR	TPIVGLSQIDA	LEDGTASTATTVQM	FGG	AL	YQ	AL	SPYV	LFTFV	QARSGG	SVL	LVSGGGV	DTPDSSSSSELIDRTPIVGLSQIDALEDGTASTATTVQMFGGALYQALSPYVLFTFVQARSGGSVLLVSGGGV	2	0.03
EU (2021)_MLG192	Europe	DT	PDSSSSSKPI	DR	TPIVGLSQIDA	PEDSTASTATTVQM	FGG	AL	YQ	AL	SPYV	LFTFV	QARSGG	SVL	LVSGGGV	DTPDSSSSSKPIDRTPIVGLSQIDAPEDSTASTATTVQMFGGALYQALSPYVLFTFVQARSGGSVLLVSGGGV	2	0.03
EU (2021)_MLG193	Europe	DT	PDSSSSSKPI	DR	TPIVGLSQIDA	PEDGTASTAITVQM	FGG	AL	YQ	AL	SPYV	LFTFV	QARSGG	SVL	LVSGGGV	DTPDSSSSSKPIDRTPIVGLSQIDAPEDGTASTAITVQMFGGALYQALSPYVLFTFVQARSGGSVLLVSGGGV	2	0.03
EU (2021)_MLG194	Europe	DT	PDSSSSSKPI	DR	TPIVGLSQVDA	PEDGTASTATTVQM	FGG	AL	YQ	AL	SPYV	LFTFV	QARSGG	SVL	LVSGGGV	DTPDSSSSSKPIDRTPIVGLSQVDAPEDGTASTATTVQMFGGALYQALSPYVLFTFVQARSGGSVLLVSGGGV	2	0.03
EU (2021)_MLG195	Europe	DT	PDSSSSSKRI	DR	TPIVGLSQIDA	PEDGTASIATTVQM	FGG	AL	YQ	AL	SPYV	LFTFV	QARSGG	SVL	LVSGGGF	DTPDSSSSSKRIDRTPIVGLSQIDAPEDGTASIATTVQMFGGALYQALSPYVLFTFVQARSGGSVLLVSGGGF	2	0.03
EU (2021)_MLG196	Europe	DT	PDSSSSSKRI	DR	TPFVGLSQIDA	PEDGTASTATTVQM	FGG	AF	YQ	AL	SPYV	LFTFV	QARSGG	SVL	LVSGGGV	DTPDSSSSSKRIDRTPFVGLSQIDAPEDGTASTATTVQMFGGAFYQALSPYVLFTFVQARSGGSVLLVSGGGV	2	0.03
EU (2021)_MLG197	Europe	DT	PDSSSSSKRI	DR	IPIVGLSQIDA	PEDGTASTATTVQM	FGG	AL	YQ	AL	SPYV	FFTFV	QARSGG	SVL	LVSGGGV	DTPDSSSSSKRIDRIPIVGLSQIDAPEDGTASTATTVQMFGGALYQALSPYVFFTFVQARSGGSVLLVSGGGV	2	0.03
EU (2021)_MLG198	Europe	DT	PDSSSSSKRI	DR	TPIVGLSQIDA	PEDVTASTATTVQM	FGG	AL	YQ	AL	SPYV	LFTFV	QARSGG	SVL	LVSGGGV	DTPDSSSSSKRIDRTPIVGLSQIDAPEDVTASTATTVQMFGGALYQALSPYVLFTFVQARSGGSVLLVSGGGV	2	0.03
EU (2021)_MLG199	Europe	DT	LDSSSSSKRI	DR	TPIVGLSQIDA	PEDGTASTATTVQM	FGG	AL	YQ	AL	SPYV	LFTFV	QARSGG	SVL	LVSGGGV	DTLDSSSSSKRIDRTPIVGLSQIDAPEDGTASTATTVQMFGGALYQALSPYVLFTFVQARSGGSVLLVSGGGV	2	0.03
EU (2021)_MLG200	Europe	DT	PDSSSSSKRI	DR	TPIVGLSQIDA	PEDGTASTATTVQM	FGG	AL	YQ	VL	SPYV	LFTFV	QARSGG	SVL	LVSGGGV	DTPDSSSSSKRIDRTPIVGLSQIDAPEDGTASTATTVQMFGGALYQVLSPYVLFTFVQARSGGSVLLVSGGGV	2	0.03
EU (2021)_MLG201	Europe	DT	PDSSSSSKRI	DR	TPIVGLSQIDA	PEDGTASTATTVQM	FGG	AL	YQ	AL	SLYV	LFTFV	QARSGG	SVL	LVSGGGV	DTPDSSSSSKRIDRTPIVGLSQIDAPEDGTASTATTVQMFGGALYQALSLYVLFTFVQARSGGSVLLVSGGGV	2	0.03
EU (2021)_MLG202	Europe	DT	PDSSSSSKRI	DR	TPIVGLSQIDA	PEDGTASTATTVQM	FGG	AL	YQ	AL	SPYV	LFTFV	QTRSGG	SVL	LVSGGGV	DTPDSSSSSKRIDRTPIVGLSQIDAPEDGTASTATTVQMFGGALYQALSPYVLFTFVQTRSGGSVLLVSGGGV	2	0.03
EU (2021)_MLG203	Europe	DT	PDSSSSSKRT	DR	TPIVGLSQIDA	PEDGTASTATTVQM	FGG	AL	YQ	AL	SPYV	LFTFV	QARSGG	SVL	LVSGGGV	DTPDSSSSSKRTDRTPIVGLSQIDAPEDGTASTATTVQMFGGALYQALSPYVLFTFVQARSGGSVLLVSGGGV	2	0.03
EU (2021)_MLG204	Europe	DT	PDSSSSSKRI	DR	TPIVGLSQIDA	PEDGIASTATTVQM	FSG	AF	YQ	AL	SPYV	LFTFV	QARSGG	SVL	LVSGGGV	DTPDSSSSSKRIDRTPIVGLSQIDAPEDGIASTATTVQMFSGAFYQALSPYVLFTFVQARSGGSVLLVSGGGV	2	0.03
EU (2021)_MLG205	Europe	DT	PDSASSSKRI	DR	TPIVGLSQIDA	PEDGIASTATTVQM	FSG	AF	YQ	AL	SPYV	LFTFV	QARSGG	SVL	LVSGGGV	DTPDSASSSKRIDRTPIVGLSQIDAPEDGIASTATTVQMFSGAFYQALSPYVLFTFVQARSGGSVLLVSGGGV	2	0.03
EU (2021)_MLG206	Europe	DT	PDSSSSSKRI	DR	TPIVGLSQIDA	PEDGTASTATTVQM	FGG	AL	YQ	AP	SPYV	LFTFV	QARSGG	SVL	LVSGDGV	DTPDSSSSSKRIDRTPIVGLSQIDAPEDGTASTATTVQMFGGALYQAPSPYVLFTFVQARSGGSVLLVSGDGV	2	0.03
EU (2021)_MLG207	Europe	DT	PDSSSSSKRI	DR	TPIVGLSQIDA	PEDGTASTATTVQM	FGG	AF	YQ	AL	SPYV	FFTFV	QARSGG	SVL	LVSGGGV	DTPDSSSSSKRIDRTPIVGLSQIDAPEDGTASTATTVQMFGGAFYQALSPYVFFTFVQARSGGSVLLVSGGGV	2	0.03

EU (2021)_MLG208	Europe	DT	PDSSSSSMGI	DR	TPIVGLSQIDA	PEDGTASTATTVQM	FGG	AL	YQ	AL	SPYV	LFTFV	QARSGG	SVL	LVSGGGV	DTPDSSSSSMGIDRTPIVGLSQIDAPEDGTASTATTVQMFGGALYQALSPYVLFTFVQARSGGSVLLVSGGGV	2	0.03
EU (2021)_MLG209	Europe	DT	PDSSSSSKGI	DR	TPIVGLSQIDA	PEDGTASTATTVQM	FGG	AL	YQ	AL	SPYV	LFTFV	QARSGG	SVL	LVSGGGV	DTPDSSSSSKGIDRTPIVGLSQIDAPEDGTASTATTVQMFGGALYQALSPYVLFTFVQARSGGSVLLVSGGGV	2	0.03
EU (2021)_MLG210	Europe	DT	PDSSSSSKAI	DR	TPIVGLSQIDA	PEDGTASTATTVQM	FGG	AL	YQ	AL	SPYV	LFTFV	QARSGG	SVL	LVSGGGV	DTPDSSSSSKAIDRTPIVGLSQIDAPEDGTASTATTVQMFGGALYQALSPYVLFTFVQARSGGSVLLVSGGGV	2	0.03
EU (2021)_MLG211	Europe	DT	PDSSSSNRGI	DR	TPIVGLSQIDA	PEDGTASTATTVQM	FGG	AL	YQ	AP	SPYV	LFTFV	HARSGG	SVS	LVSGDGV	DTPDSSSSNRGIDRTPIVGLSQIDAPEDGTASTATTVQMFGGALYQAPSPYVLFTFVHARSGGSVSLVSGDGV	1	0.01
EU (2021)_MLG212	Europe	DT	PDSSSSSRGI	DC	TPIVGLSQIDA	PEDGTVSTATTVQM	FGG	AL	YQ	AL	SPYV	LFTFV	QARSGG	SVL	LVSGGGV	DTPDSSSSSRGIDCTPIVGLSQIDAPEDGTVSTATTVQMFGGALYQALSPYVLFTFVQARSGGSVLLVSGGGV	1	0.01
EU (2021)_MLG213	Europe	DT	PDSSSSSRGI	DR	TPIIGLSQIDA	PEDGTASTATTVQM	FGG	AL	YQ	AL	SPYV	LFTFV	QARSGG	SVL	LVSGGGV	DTPDSSSSSRGIDRTPIIGLSQIDAPEDGTASTATTVQMFGGALYQALSPYVLFTFVQARSGGSVLLVSGGGV	1	0.01
EU (2021)_MLG214	Europe	DT	PDSSSSSRGI	DR	TPIVGLSQIDA	PEDGTVSTATTVQM	FGG	AL	YQ	AL	SPYV	LFTFV	HARSGG	SVL	LVNGGGV	DTPDSSSSSRGIDRTPIVGLSQIDAPEDGTVSTATTVQMFGGALYQALSPYVLFTFVHARSGGSVLLVNGGGV	1	0.01
EU (2021)_MLG215	Europe	DT	PDSSSSSRGI	DR	TPIVGLSQIDA	PEDGTASTATTVQM	FGG	AL	YQ	AL	SPYV	LFTFV	HARSGG	SVL	FVNGGGV	DTPDSSSSSRGIDRTPIVGLSQIDAPEDGTASTATTVQMFGGALYQALSPYVLFTFVHARSGGSVLFVNGGGV	1	0.01
EU (2021)_MLG216	Europe	DT	PDSLSSSRGI	DR	TPIVGLSQIDA	PEDGTASTATTVQM	FGG	AF	YQ	AL	SPYV	LFTFV	HARSGG	SLL	LVSGGGV	DTPDSLSSSRGIDRTPIVGLSQIDAPEDGTASTATTVQMFGGAFYQALSPYVLFTFVHARSGGSLLLVSGGGV	1	0.01
EU (2021)_MLG217	Europe	DT	SDSLSSSRGI	DR	TPIVGLSQIDA	PEDGTASTATTVQM	FGG	AL	YQ	AL	SPYV	LFTFV	HARSGG	SVL	LVSGGGV	DTSDSLSSSRGIDRTPIVGLSQIDAPEDGTASTATTVQMFGGALYQALSPYVLFTFVHARSGGSVLLVSGGGV	1	0.01
EU (2021)_MLG218	Europe	DT	PDSSSSSRGI	DR	TPIVGLSQIDA	SEDGTASTATTVQM	FGG	AL	YQ	AL	SPYV	LFTFV	HARSGG	SVL	LVNGGGV	DTPDSSSSSRGIDRTPIVGLSQIDASEDGTASTATTVQMFGGALYQALSPYVLFTFVHARSGGSVLLVNGGGV	1	0.01
EU (2021)_MLG219	Europe	DT	PDSSSSSRGI	DR	TPIVGLSQIDA	PEDGTASTATTVQM	FGG	AL	YQ	AL	SPYV	LFTFL	HARSGG	SVL	LVSGGGV	DTPDSSSSSRGIDRTPIVGLSQIDAPEDGTASTATTVQMFGGALYQALSPYVLFTFLHARSGGSVLLVSGGGV	1	0.01
EU (2021)_MLG220	Europe	DT	PNSSSSSRGI	DR	TPIVGLSQIDA	PEDGTASTATTVQM	FGG	AF	YQ	AL	SPYV	LFTFV	QARSGG	SVL	LVSGGGV	DTPNSSSSSRGIDRTPIVGLSQIDAPEDGTASTATTVQMFGGAFYQALSPYVLFTFVQARSGGSVLLVSGGGV	1	0.01
EU (2021)_MLG221	Europe	DT	PDSSSSSRGI	DR	TPIVGLSQIDA	PEDGTASTATTVQM	FGG	AL	YQ	AL	SPYV	LFTFV	QARSGG	SVL	FVSGGGV	DTPDSSSSSRGIDRTPIVGLSQIDAPEDGTASTATTVQMFGGALYQALSPYVLFTFVQARSGGSVLFVSGGGV	1	0.01
EU (2021)_MLG222	Europe	DT	PDSSSSSRGI	DR	TPIVGLSQIDA	PEDGTASKATTVQM	FGG	AL	YQ	AL	SPYV	LFTFV	QARSGG	SVL	LVSGGGV	DTPDSSSSSRGIDRTPIVGLSQIDAPEDGTASKATTVQMFGGALYQALSPYVLFTFVQARSGGSVLLVSGGGV	1	0.01
EU (2021)_MLG223	Europe	DT	PDSSSSSRGI	DR	TPIVGLSQIDA	PEDGTASTATTVQM	FGG	AL	YQ	AP	SPYV	LFTFV	HARSGG	SVL	LVNGGGV	DTPDSSSSSRGIDRTPIVGLSQIDAPEDGTASTATTVQMFGGALYQAPSPYVLFTFVHARSGGSVLLVNGGGV	1	0.01
EU (2021)_MLG224	Europe	DT	PDSSSSSRGI	DR	TPIVGLSQIDA	PEDGTASTATTVQM	FGG	AL	YQ	AL	SPYV	LFTFV	QAKSGG	SVL	LVSGGGV	DTPDSSSSSRGIDRTPIVGLSQIDAPEDGTASTATTVQMFGGALYQALSPYVLFTFVQAKSGGSVLLVSGGGV	1	0.01
EU (2021)_MLG225	Europe	DT	PDSSSSSRGI	DR	TPIVGLSQIDA	PEDGTASTATTVQM	FGG	AL	YQ	AL	SSYV	LFTFV	QARSGG	SVL	LVSGGGV	DTPDSSSSSRGIDRTPIVGLSQIDAPEDGTASTATTVQMFGGALYQALSSYVLFTFVQARSGGSVLLVSGGGV	1	0.01
EU (2021)_MLG226	Europe	DT	PDSSSSSRGI	DR	TPIVGLSQIDA	PEDGTASTATTVQI	FGG	AL	YQ	AL	SPYV	LFTFV	HARSGG	SVL	LVNGGGV	DTPDSSSSSRGIDRTPIVGLSQIDAPEDGTASTATTVQIFGGALYQALSPYVLFTFVHARSGGSVLLVNGGGV	1	0.01
EU (2021)_MLG227	Europe	DT	PDSSSSSRGI	DR	TPIVGLSQIDA	PEDGTASTATTVQM	FGG	AL	YQ	AL	SPYV	LFTFV	QGRSGG	SVL	LVSGGGV	DTPDSSSSSRGIDRTPIVGLSQIDAPEDGTASTATTVQMFGGALYQALSPYVLFTFVQGRSGGSVLLVSGGGV	1	0.01
EU (2021)_MLG228	Europe	DT	SDSSLSSRGI	DR	TPIVGLSQIDA	PEDGTASTATTVQM	FGG	AL	YQ	AL	SPYV	LFTFV	HARSGG	SVL	LVSGGGV	DTSDSSLSSRGIDRTPIVGLSQIDAPEDGTASTATTVQMFGGALYQALSPYVLFTFVHARSGGSVLLVSGGGV	1	0.01
EU (2021)_MLG229	Europe	DT	PDSSSSSRGI	DR	TPIVGLSQIDA	PEDGTASTATTVQM	FGG	AL	YQ	AL	SPYV	LFTFV	HARSGS	SVL	LVNGGGV	DTPDSSSSSRGIDRTPIVGLSQIDAPEDGTASTATTVQMFGGALYQALSPYVLFTFVHARSGSSVLLVNGGGV	1	0.01
EU (2021)_MLG230	Europe	DT	PDSSSSSRGI	DR	TPIVGLSQIDA	PEDGTASTATTVQM	FGG	AL	YQ	AL	SPYV	LFTFV	QARSGG	SVL	LVSGDGV	DTPDSSSSSRGIDRTPIVGLSQIDAPEDGTASTATTVQMFGGALYQALSPYVLFTFVQARSGGSVLLVSGDGV	1	0.01
EU (2021)_MLG231	Europe	DT	PDSSSSSRGI	DR	IPIVGLSQIDA	PEDGTASTATTVQM	FGG	AL	YQ	AP	SPYV	LFTFV	HARSGG	SVL	LVSGGGV	DTPDSSSSSRGIDRIPIVGLSQIDAPEDGTASTATTVQMFGGALYQAPSPYVLFTFVHARSGGSVLLVSGGGV	1	0.01
EU (2021)_MLG232	Europe	DT	PDSSSSNRGI	DR	TPIVGLSQIDA	PEDGTASTATTVQM	FGG	AL	YQ	AL	SPYV	LFTFV	QARSGG	SVL	LVNGGGV	DTPDSSSSNRGIDRTPIVGLSQIDAPEDGTASTATTVQMFGGALYQALSPYVLFTFVQARSGGSVLLVNGGGV	1	0.01
EU (2021)_MLG233	Europe	DT	PDSSSSSRGI	DR	TPIVGFSQIDA	PEDGTASTATTVQM	FGG	AL	YQ	AL	SPYV	LFTFV	HARSGG	SVL	LVNGGGV	DTPDSSSSSRGIDRTPIVGFSQIDAPEDGTASTATTVQMFGGALYQALSPYVLFTFVHARSGGSVLLVNGGGV	1	0.01
EU (2021)_MLG234	Europe	DT	PDSSSSSRGI	DR	TPIVGLSQIDA	PEDGTASTATTVQM	FGG	AL	YQ	AL	SPYV	LFTFV	QARSGG	LVL	LVSGGGV	DTPDSSSSSRGIDRTPIVGLSQIDAPEDGTASTATTVQMFGGALYQALSPYVLFTFVQARSGGLVLLVSGGGV	1	0.01
EU (2021)_MLG235	Europe	DT	PDSSLSSRGI	DR	TPIVGLSQIDA	PEDGTASTATTVQM	FGG	AL	YQ	AL	SLYV	LFTFV	HARSGG	SVL	LVSGGGV	DTPDSSLSSRGIDRTPIVGLSQIDAPEDGTASTATTVQMFGGALYQALSLYVLFTFVHARSGGSVLLVSGGGV	1	0.01
EU (2021)_MLG236	Europe	DT	RDSSSSSRGI	DR	TPIVGLSQIDA	PEDGTASTATTVQM	FGG	AL	YQ	AL	SPYV	LFTFV	QARSGG	SVL	LVSGGGV	DTRDSSSSSRGIDRTPIVGLSQIDAPEDGTASTATTVQMFGGALYQALSPYVLFTFVQARSGGSVLLVSGGGV	1	0.01
EU (2021)_MLG237	Europe	DT	PDSSSSSRGI	DR	IPIVCLSQIDA	PEDGTASTATTVQM	FGG	AL	YQ	AL	SPYV	LFTFV	HARSGG	SVL	LVSGGGV	DTPDSSSSSRGIDRIPIVCLSQIDAPEDGTASTATTVQMFGGALYQALSPYVLFTFVHARSGGSVLLVSGGGV	1	0.01
EU (2021)_MLG238	Europe	DT	PDSSSSSRGI	DR	TPIVGLSQIDA	PEDGTASTATTVQM	FGG	AL	YQ	AL	SSYV	LFTFV	HARSGG	SVL	LVNGGGV	DTPDSSSSSRGIDRTPIVGLSQIDAPEDGTASTATTVQMFGGALYQALSSYVLFTFVHARSGGSVLLVNGGGV	1	0.01
EU (2021)_MLG239	Europe	DT	PDSSSSSRGI	DR	TPIVGLSQIDA	PEDGTASTATIVQM	FGG	AL	YQ	AL	SPYV	LFTFV	HARSGG	SVL	LVNGGGV	DTPDSSSSSRGIDRTPIVGLSQIDAPEDGTASTATIVQMFGGALYQALSPYVLFTFVHARSGGSVLLVNGGGV	1	0.01
EU (2021)_MLG240	Europe	DT	PDSSSSNRGI	DR	TPIVGLSQIDA	PEDGTASTATTVQM	FGG	TL	YQ	AP	SPYV	LFTFV	QARSGG	SVS	LVSGGGV	DTPDSSSSNRGIDRTPIVGLSQIDAPEDGTASTATTVQMFGGTLYQAPSPYVLFTFVQARSGGSVSLVSGGGV	1	0.01
EU (2021)_MLG241	Europe	DT	PDSSLSSRGI	DR	TPIVGLSQIDA	PEDGTASTVTTVQM	FGG	AL	YQ	AL	SPYV	LFTFV	QARSGG	SVL	LVSGGGV	DTPDSSLSSRGIDRTPIVGLSQIDAPEDGTASTVTTVQMFGGALYQALSPYVLFTFVQARSGGSVLLVSGGGV	1	0.01
EU (2021)_MLG242	Europe	DT	PDSSSSSRGI	DR	TPIVGLSQIDA	PEDGTASTATTVQM	FGG	AL	YQ	AL	SPYV	LFTFV	QARSGG	SLL	LVSGGGV	DTPDSSSSSRGIDRTPIVGLSQIDAPEDGTASTATTVQMFGGALYQALSPYVLFTFVQARSGGSLLLVSGGGV	1	0.01
EU (2021)_MLG243	Europe	DT	PDSSSSSRGI	DR	TPIVGLSQIDA	PEDGTASTATTVQM	FGG	AL	YQ	AL	SPYV	LFTFV	HARSGG	LVL	LVNGGGV	DTPDSSSSSRGIDRTPIVGLSQIDAPEDGTASTATTVQMFGGALYQALSPYVLFTFVHARSGGLVLLVNGGGV	1	0.01
EU (2021)_MLG244	Europe	DT	PDSSLSSRGI	DR	TPIVGLSQIDA	PEDGTASTATTVQM	FGG	VL	YQ	AL	SPYV	LFTFV	HARSGG	SVL	LVSGGGV	DTPDSSLSSRGIDRTPIVGLSQIDAPEDGTASTATTVQMFGGVLYQALSPYVLFTFVHARSGGSVLLVSGGGV	1	0.01
EU (2021)_MLG245	Europe	DM	PDSSSSSRGI	DR	TPIVGLSQIDA	PEDGTASTATTVQM	FGG	AL	YQ	AL	SPYV	LFTFV	QARSGG	SVL	LVSGGGV	DMPDSSSSSRGIDRTPIVGLSQIDAPEDGTASTATTVQMFGGALYQALSPYVLFTFVQARSGGSVLLVSGGGV	1	0.01
EU (2021)_MLG246	Europe	DT	PDSLSSSRGI	DR	TLIVGLSQIDA	PEDGTASTATTVQM	FGG	AL	YQ	AL	SPYV	LFTFV	HARSGG	SVL	LVSGGGV	DTPDSLSSSRGIDRTLIVGLSQIDAPEDGTASTATTVQMFGGALYQALSPYVLFTFVHARSGGSVLLVSGGGV	1	0.01
EU (2021)_MLG247	Europe	DT	PDSSSSSRGI	DR	TPIVGLSQIDA	PEDGTASTATTVQM	FGG	AL	YQ	AL	SPYV	LFTFV	HARSGG	SVL	LVSGGGF	DTPDSSSSSRGIDRTPIVGLSQIDAPEDGTASTATTVQMFGGALYQALSPYVLFTFVHARSGGSVLLVSGGGF	1	0.01
EU (2021)_MLG248	Europe	DT	PDSSSSSRGI	DC	IPIVGLSQIDA	PEDGTASTATTVQM	FGG	AL	YQ	AL	SPYV	LFTFV	HARSGG	LVL	LVSGGGV	DTPDSSSSSRGIDCIPIVGLSQIDAPEDGTASTATTVQMFGGALYQALSPYVLFTFVHARSGGLVLLVSGGGV	1	0.01
EU (2021)_MLG249	Europe	DT	PDSSSSSRGI	DR	TPIVGLSQIDA	PEDGTASTAITVQM	FGG	AL	YQ	AL	SPYV	LFTFV	QARSGG	SVL	LVSGGGV	DTPDSSSSSRGIDRTPIVGLSQIDAPEDGTASTAITVQMFGGALYQALSPYVLFTFVQARSGGSVLLVSGGGV	1	0.01
EU (2021)_MLG250	Europe	DT	PDSLSSSRGI	DR	TPIVGLSQIDA	PEDGTASTATTVQM	FGG	AF	YQ	AL	SPYV	LFTFV	HARSGG	SVL	LVSGGGV	DTPDSLSSSRGIDRTPIVGLSQIDAPEDGTASTATTVQMFGGAFYQALSPYVLFTFVHARSGGSVLLVSGGGV	1	0.01
EU (2021)_MLG251	Europe	DT	PDSSSSSRGI	DR	TPIVGLSQIDA	PEDGTASTATTVQM	FGG	AF	YQ	AL	SPYV	LFTFV	HARSGG	SVL	LVSGGGV	DTPDSSSSSRGIDRTPIVGLSQIDAPEDGTASTATTVQMFGGAFYQALSPYVLFTFVHARSGGSVLLVSGGGV	1	0.01
EU (2021)_MLG252	Europe	DT	PDSSSSSRGI	DR	TPIVGLSQIDA	PEDGTASTATTVQM	FGG	AL	YQ	AL	SPYV	LFTFV	QARSGG	SVL	LVNGGGV	DTPDSSSSSRGIDRTPIVGLSQIDAPEDGTASTATTVQMFGGALYQALSPYVLFTFVQARSGGSVLLVNGGGV	1	0.01
EU (2021)_MLG253	Europe	DT	PDSSLSSRGI	DR	TPIVGLSQIDA	PEDGTAITATTVQM	FGG	AL	YQ	AL	SPYV	LFTFV	QARSGG	SVL	LVSGGGV	DTPDSSLSSRGIDRTPIVGLSQIDAPEDGTAITATTVQMFGGALYQALSPYVLFTFVQARSGGSVLLVSGGGV	1	0.01
EU (2021)_MLG254	Europe	DT	PDSLASSRGI	DR	TPIVGLSQIDA	PEDGTASTATTVQM	FGG	AL	YQ	AL	SPYV	LFTFV	QARSGG	SVL	LVSGGGV	DTPDSLASSRGIDRTPIVGLSQIDAPEDGTASTATTVQMFGGALYQALSPYVLFTFVQARSGGSVLLVSGGGV	1	0.01
EU (2021)_MLG255	Europe	DT	PDSSSSSELI	DR	TPIVGLSQIDA	PEDGTASTATTVQM	FGG	AL	YQ	AL	SPYV	LFTFV	QARSGG	SVL	LVSGGGV	DTPDSSSSSELIDRTPIVGLSQIDAPEDGTASTATTVQMFGGALYQALSPYVLFTFVQARSGGSVLLVSGGGV	1	0.01
EU (2021)_MLG256	Europe	DT	PDSSSSSKLI	DR	TPIVGLSQIDA	PEDGTASTATTVQM	FGG	AF	YQ	AL	SPYV	LFTFV	QARSGG	SVL	LVSGGGV	DTPDSSSSSKLIDRTPIVGLSQIDAPEDGTASTATTVQMFGGAFYQALSPYVLFTFVQARSGGSVLLVSGGGV	1	0.01
EU (2021)_MLG257	Europe	DT	PDSSSSSKLI	DR	TPIVGLSQIDA	PEEGTASTATTVQM	FGG	AL	YQ	AL	SPYV	LFTFV	QARSGG	SVL	LVSGGGV	DTPDSSSSSKLIDRTPIVGLSQIDAPEEGTASTATTVQMFGGALYQALSPYVLFTFVQARSGGSVLLVSGGGV	1	0.01
EU (2021)_MLG258	Europe	DT	PDSSSSSKPI	DR	TPIVGLSQIDA	PEDGTASTATTVQM	FGG	AL	YQ	AL	SPYV	LFTFV	QARSGS	SVL	LVSGGGV	DTPDSSSSSKPIDRTPIVGLSQIDAPEDGTASTATTVQMFGGALYQALSPYVLFTFVQARSGSSVLLVSGGGV	1	0.01
EU (2021)_MLG259	Europe	DT	PDSSSSSKPI	DC	TPIVGLSQIDA	PEDGTASTATTVQM	FGG	AL	YQ	AL	SPYV	LFTFV	QARSGG	SVL	LVSGGGV	DTPDSSSSSKPIDCTPIVGLSQIDAPEDGTASTATTVQMFGGALYQALSPYVLFTFVQARSGGSVLLVSGGGV	1	0.01
EU (2021)_MLG260	Europe	DT	PDSSSSSKPI	DR	TPIVGLSQIDA	PEDGTASTATTVQM	FGG	AF	YQ	AL	SPYV	LFTFL	QARSGG	SVL	LVSGGGV	DTPDSSSSSKPIDRTPIVGLSQIDAPEDGTASTATTVQMFGGAFYQALSPYVLFTFLQARSGGSVLLVSGGGV	1	0.01
EU (2021)_MLG261	Europe	DT	PDSSSSSKPI	DR	TPIVGLSQIDA	PEDGTASTATTVQM	FGG	AL	YQ	AL	SPYV	LFTFV	HARSGG	SVL	LVNGGGV	DTPDSSSSSKPIDRTPIVGLSQIDAPEDGTASTATTVQMFGGALYQALSPYVLFTFVHARSGGSVLLVNGGGV	1	0.01
EU (2021)_MLG262	Europe	DT	PDSSSSSKRI	DR	TPIVGLSQIDA	PEDGTASTATTVQM	FGG	AL	YQ	AL	SPYV	LFTFV	QARSVG	SVL	LVSGGGV	DTPDSSSSSKRIDRTPIVGLSQIDAPEDGTASTATTVQMFGGALYQALSPYVLFTFVQARSVGSVLLVSGGGV	1	0.01
EU (2021)_MLG263	Europe	DT	PDSSSSSKRI	DR	TSIVGLSQIDA	PEDGTASTATTVQM	FGG	AL	YQ	AL	SPYV	LFTFV	QARSGG	SVL	LVSGGGV	DTPDSSSSSKRIDRTSIVGLSQIDAPEDGTASTATTVQMFGGALYQALSPYVLFTFVQARSGGSVLLVSGGGV	1	0.01
EU (2021)_MLG264	Europe	DT	SVSSSTSKRI	DR	TPIVGLSQIDA	PEDGTASTATTVQM	FGG	AL	YQ	AL	SPYV	LFTFV	QARSGG	SVL	LVSGGGV	DTSVSSSTSKRIDRTPIVGLSQIDAPEDGTASTATTVQMFGGALYQALSPYVLFTFVQARSGGSVLLVSGGGV	1	0.01
EU (2021)_MLG265	Europe	DT	PDSSSSSKRI	DR	TPIVGLSQIDA	PEDGIASTATTVQM	FGG	AL	YQ	AL	SPYV	LFTFV	QARSGG	SVL	LVSGGGV	DTPDSSSSSKRIDRTPIVGLSQIDAPEDGIASTATTVQMFGGALYQALSPYVLFTFVQARSGGSVLLVSGGGV	1	0.01
EU (2021)_MLG266	Europe	DT	PDSSSSSKRI	DH	TPIVGLSQIDA	PEDGTASTATTVQM	FGG	AL	YQ	AL	SPYV	LFTFV	QARSGG	SVL	LVSGGGV	DTPDSSSSSKRIDHTPIVGLSQIDAPEDGTASTATTVQMFGGALYQALSPYVLFTFVQARSGGSVLLVSGGGV	1	0.01
EU (2021)_MLG267	Europe	DT	SDSSSTSKRI	NR	TPIVGLSQIDA	PEDGTASTATTVQM	FGG	AL	YQ	AL	SPYV	LFTFV	QARSGG	SVL	LVSGGGV	DTSDSSSTSKRINRTPIVGLSQIDAPEDGTASTATTVQMFGGALYQALSPYVLFTFVQARSGGSVLLVSGGGV	1	0.01
EU (2021)_MLG268	Europe	DT	PDSSSSSKRI	DR	TPIVGLSQIDA	PEDGTASTATTVQM	FGG	AL	YQ	AL	SPYV	LFTFV	QARSGG	SVL	SVSGGGV	DTPDSSSSSKRIDRTPIVGLSQIDAPEDGTASTATTVQMFGGALYQALSPYVLFTFVQARSGGSVLSVSGGGV	1	0.01
EU (2021)_MLG269	Europe	DT	PDSSSSSKRI	DR	TPIVGLSQIDA	PEDGTASTATTVQM	FGG	AL	YQ	AL	SPYV	LFTFV	HARSGG	SVL	LVNGGGV	DTPDSSSSSKRIDRTPIVGLSQIDAPEDGTASTATTVQMFGGALYQALSPYVLFTFVHARSGGSVLLVNGGGV	1	0.01
EU (2021)_MLG270	Europe	DT	PDSSSSSKRI	DR	TPIVGLSQIDA	LEDGTASTATTVQM	FGG	AF	YQ	AL	SPYV	LFTFV	QARSGG	SVL	LVSGGGV	DTPDSSSSSKRIDRTPIVGLSQIDALEDGTASTATTVQMFGGAFYQALSPYVLFTFVQARSGGSVLLVSGGGV	1	0.01
EU (2021)_MLG271	Europe	DT	PDSSSSSKRI	DR	TPIVGLSQIDA	PEDGTASTATTVQM	FGG	AL	YQ	AL	SPYV	LFTFV	QSRSGG	SVL	LVSGGGV	DTPDSSSSSKRIDRTPIVGLSQIDAPEDGTASTATTVQMFGGALYQALSPYVLFTFVQSRSGGSVLLVSGGGV	1	0.01

EU (2021)_MLG272	Europe	DT	PDSSSSSKRI	DR	TPIVGLSQIDA	PEDGTASTATTVQM	FGG	AL	YQ	SL	SPYV	LFTFV	QARSGG	SVL	LVSGGGV	DTPDSSSSSKRIDRTPIVGLSQIDAPEDGTASTATTVQMFGGALYQSLSPYVLFTFVQARSGGSVLLVSGGGV	1	0.01
EU (2021)_MLG273	Europe	DT	PDSSSSSKRI	DR	TPIVGLSQIDA	PEDGTASTATTVQM	FGG	AL	YQ	AL	SPYV	LFTFV	EARSGG	SVL	LVSGGGV	DTPDSSSSSKRIDRTPIVGLSQIDAPEDGTASTATTVQMFGGALYQALSPYVLFTFVEARSGGSVLLVSGGGV	1	0.01
EU (2021)_MLG274	Europe	DT	SDSSSTSKRI	DR	TPIVGLSQIDA	PEDGTASTATTVQM	FGG	AL	YQ	AL	SPYV	LFTFV	HARSGG	SVL	LVSGGGV	DTSDSSSTSKRIDRTPIVGLSQIDAPEDGTASTATTVQMFGGALYQALSPYVLFTFVHARSGGSVLLVSGGGV	1	0.01
EU (2021)_MLG275	Europe	DT	PDSSSSSKRI	DR	TPIVGLSQIDA	PEDGTASTATTVQM	FGG	AF	YQ	AL	SPYV	LFTFV	QARSGS	SVL	LVSGGGV	DTPDSSSSSKRIDRTPIVGLSQIDAPEDGTASTATTVQMFGGAFYQALSPYVLFTFVQARSGSSVLLVSGGGV	1	0.01
EU (2021)_MLG276	Europe	DT	PDSSSSSKRI	DR	TPIVGLSQIDA	PEEGTASTATTVQM	FGG	AL	YQ	AL	SPYV	LFTFV	QARSGG	SLL	LVSGGGV	DTPDSSSSSKRIDRTPIVGLSQIDAPEEGTASTATTVQMFGGALYQALSPYVLFTFVQARSGGSLLLVSGGGV	1	0.01
EU (2021)_MLG277	Europe	DT	PDSSSSSKRI	DR	TPIVGLSQIDA	LEDGTASTATTVQM	FGG	AL	YQ	AL	SPYV	LFTFV	QARFGG	SVL	LVSGGGV	DTPDSSSSSKRIDRTPIVGLSQIDALEDGTASTATTVQMFGGALYQALSPYVLFTFVQARFGGSVLLVSGGGV	1	0.01
EU (2021)_MLG278	Europe	DT	PDSSSSSRRI	DR	TPIVGLSQIDA	PEDGTASTATTVQM	FGG	AL	YQ	AL	SPYV	LFTFV	QARSGG	SVL	LVSGGGF	DTPDSSSSSRRIDRTPIVGLSQIDAPEDGTASTATTVQMFGGALYQALSPYVLFTFVQARSGGSVLLVSGGGF	1	0.01
EU (2021)_MLG279	Europe	DT	PDSSSSSKRI	DR	TPIVGLSQIDA	PEDGTASTATTVHM	FGG	AL	YQ	AL	SPYV	LFTFV	QARSGG	SVL	LVSGGGV	DTPDSSSSSKRIDRTPIVGLSQIDAPEDGTASTATTVHMFGGALYQALSPYVLFTFVQARSGGSVLLVSGGGV	1	0.01
EU (2021)_MLG280	Europe	DT	PDSSSSSKRI	DR	TPIVGLSQIDA	PEDGTASTATTVQM	FGS	AL	YQ	AL	SPYV	LFTFV	QARSGG	SFL	LVSGGGV	DTPDSSSSSKRIDRTPIVGLSQIDAPEDGTASTATTVQMFGSALYQALSPYVLFTFVQARSGGSFLLVSGGGV	1	0.01
EU (2021)_MLG281	Europe	DT	PDSSLSSKRI	DR	TPIVGLSQIDA	PEDGTASTATTVQM	FGG	AL	YQ	AL	SPYV	LFTFV	QARSGG	SML	LVSGGGV	DTPDSSLSSKRIDRTPIVGLSQIDAPEDGTASTATTVQMFGGALYQALSPYVLFTFVQARSGGSMLLVSGGGV	1	0.01
EU (2021)_MLG282	Europe	DT	PDSSSSSKRI	DR	TLIVGLSQIDA	PEDGTASTATTVQM	FGG	AL	YQ	AL	SPYV	LFTFV	QARSGG	SVL	LVSGGGF	DTPDSSSSSKRIDRTLIVGLSQIDAPEDGTASTATTVQMFGGALYQALSPYVLFTFVQARSGGSVLLVSGGGF	1	0.01
EU (2021)_MLG283	Europe	DM	PDSSSSSKRI	DR	TPIVGLSQIDA	PEDGTASTATTVQM	FGG	AL	YQ	AL	SPYV	LFTFV	QARSGG	SVL	LVSGGGV	DMPDSSSSSKRIDRTPIVGLSQIDAPEDGTASTATTVQMFGGALYQALSPYVLFTFVQARSGGSVLLVSGGGV	1	0.01
EU (2021)_MLG284	Europe	DT	PDSSSSSKRI	DR	TPIVGLSQIDA	PEDGTASTATTVQM	FDG	AL	YQ	AL	SPYV	LFTFV	QARSGG	SVL	LVSGGGV	DTPDSSSSSKRIDRTPIVGLSQIDAPEDGTASTATTVQMFDGALYQALSPYVLFTFVQARSGGSVLLVSGGGV	1	0.01
EU (2021)_MLG285	Europe	DT	PDSSSSSKRI	DR	TPIVGLSQIDA	PEDGIASTATTVQM	FGG	AL	YQ	AL	SPYV	LFTFV	QARSGG	SVL	LVSGGGF	DTPDSSSSSKRIDRTPIVGLSQIDAPEDGIASTATTVQMFGGALYQALSPYVLFTFVQARSGGSVLLVSGGGF	1	0.01
EU (2021)_MLG286	Europe	DT	PDSSSSSKRI	DR	TPIVGLSQIDA	PEDGTASTATTVQM	FGG	AL	YQ	AL	SPYV	LFTFV	QARSGG	SML	LVSGGGV	DTPDSSSSSKRIDRTPIVGLSQIDAPEDGTASTATTVQMFGGALYQALSPYVLFTFVQARSGGSMLLVSGGGV	1	0.01
EU (2021)_MLG287	Europe	DT	PDSSSSSKRI	DR	TPIVGLSQIDA	PEDGTASTATTVQM	FGG	AL	YQ	AL	LPYV	LFTFV	QARSGG	SVL	LVSGGGV	DTPDSSSSSKRIDRTPIVGLSQIDAPEDGTASTATTVQMFGGALYQALLPYVLFTFVQARSGGSVLLVSGGGV	1	0.01
EU (2021)_MLG288	Europe	AT	PDSSSSSKRI	DR	TPIVGLSQIDA	PEDGTASTATTVQM	FGG	AL	YQ	AL	SPYV	LFTFV	QARSGG	SVL	LVSGGGV	ATPDSSSSSKRIDRTPIVGLSQIDAPEDGTASTATTVQMFGGALYQALSPYVLFTFVQARSGGSVLLVSGGGV	1	0.01
EU (2021)_MLG289	Europe	DT	PDSSSSSKRI	DR	TPIVGLSRIDA	PEDGTASTATTVQM	FGG	AL	YQ	AL	SPYV	LFTFV	QARSGG	SVL	LVSGGGV	DTPDSSSSSKRIDRTPIVGLSRIDAPEDGTASTATTVQMFGGALYQALSPYVLFTFVQARSGGSVLLVSGGGV	1	0.01
EU (2021)_MLG290	Europe	DT	PDSSSSSKRI	DR	TPIVGLSQIDA	PEDGTASTATTVQM	FGG	AL	YQ	AL	SPYV	LFTFV	QVRSGG	SVL	LVSGGGV	DTPDSSSSSKRIDRTPIVGLSQIDAPEDGTASTATTVQMFGGALYQALSPYVLFTFVQVRSGGSVLLVSGGGV	1	0.01
EU (2021)_MLG291	Europe	DT	PDSSSSSKRI	DR	TPIVGLSQIDA	PEDGTTSTATTVQM	FGG	AL	YQ	AL	SPYV	LFTFV	QARSGG	SVL	LVSGGGV	DTPDSSSSSKRIDRTPIVGLSQIDAPEDGTTSTATTVQMFGGALYQALSPYVLFTFVQARSGGSVLLVSGGGV	1	0.01
EU (2021)_MLG292	Europe	DT	PDSSSSSKRI	DR	TPIVGLSQIDA	PEEGTASTATTVQM	FGG	AL	YQ	AL	SPYV	LFTFV	QARSGG	SVL	LVSGGGV	DTPDSSSSSKRIDRTPIVGLSQIDAPEEGTASTATTVQMFGGALYQALSPYVLFTFVQARSGGSVLLVSGGGV	1	0.01
EU (2021)_MLG293	Europe	DT	PDSSSSFKRI	DR	TPIVGLSQIDA	PEDGTASTATTVQM	FGG	AL	YQ	AL	SPYV	LFTFV	QARSGG	SVL	LVSGGGF	DTPDSSSSFKRIDRTPIVGLSQIDAPEDGTASTATTVQMFGGALYQALSPYVLFTFVQARSGGSVLLVSGGGF	1	0.01
EU (2021)_MLG294	Europe	DT	PDSSSSSKRI	DR	TPIVGLSQIDA	PEDGTASTATTVQM	FGG	AF	YQ	AL	SPYV	LFTFV	QARSGG	SLL	LVSGGGV	DTPDSSSSSKRIDRTPIVGLSQIDAPEDGTASTATTVQMFGGAFYQALSPYVLFTFVQARSGGSLLLVSGGGV	1	0.01
EU (2021)_MLG295	Europe	DT	PDSSSSNKGI	DR	TPIVGLSQIDA	PEDGTASTATTVQM	FGG	AL	YQ	AP	SPYV	LFTFV	QARSGG	SVS	LVSGDGV	DTPDSSSSNKGIDRTPIVGLSQIDAPEDGTASTATTVQMFGGALYQAPSPYVLFTFVQARSGGSVSLVSGDGV	1	0.01
EU (2021)_MLG296	Europe	DT	PDSSSSSMGI	DR	TPIVGLSQIDA	PEDGTASTATTVQM	FGG	AL	YQ	AL	SLYV	LFTFV	QARSGG	SVL	LVSGGGV	DTPDSSSSSMGIDRTPIVGLSQIDAPEDGTASTATTVQMFGGALYQALSLYVLFTFVQARSGGSVLLVSGGGV	1	0.01
NA (2020)_MLG1	N America	DT	PDSSSLSRGI	DR	TPIVGLSQIPA	SPEDGTASTATTVQM	YGG	AL	YQ	AP	SPYV	LFTFV	QARSVG	SVS	LVSGDGV	DTPDSSSLSRGIDRTPIVGLSQIPASPEDGTASTATTVQMYGGALYQAPSPYVLFTFVQARSVGSVSLVSGDGV	169	28.30
NA (2020)_MLG2	N America	DT	PDSSSSSRGI	DR	IPIVGLSQIPA	SPEDGTASTATTVQM	FGG	AL	YQ	AL	SPYV	LFTFV	HARSGG	SVL	LVSGGGV	DTPDSSSSSRGIDRIPIVGLSQIPASPEDGTASTATTVQMFGGALYQALSPYVLFTFVHARSGGSVLLVSGGGV	105	17.60
NA (2020)_MLG3	N America	DT	PDSSSSSRGI	DR	IPIVGLSQIPA	SPEDGTASTATTVQM	FGG	AL	YQ	AL	SPYV	LFTFV	HARSGG	LVL	LVSGGGV	DTPDSSSSSRGIDRIPIVGLSQIPASPEDGTASTATTVQMFGGALYQALSPYVLFTFVHARSGGLVLLVSGGGV	55	9.20
NA (2020)_MLG4	N America	DT	PDSSSSSRGI	DR	TPIVGLSQIPA	SPEDGTASTATTVQM	FGG	AL	YQ	AL	SPYV	LFTFV	QARSGG	SVL	LVSGGGV	DTPDSSSSSRGIDRTPIVGLSQIPASPEDGTASTATTVQMFGGALYQALSPYVLFTFVQARSGGSVLLVSGGGV	39	6.50
NA (2020)_MLG5	N America	DT	PDSSSSSRGI	DR	TPIVGLSQIPA	SPEDGTASTATTVQM	FGG	AL	YQ	AL	SPYV	LFTFV	QARSGS	SVL	LVSGGGV	DTPDSSSSSRGIDRTPIVGLSQIPASPEDGTASTATTVQMFGGALYQALSPYVLFTFVQARSGSSVLLVSGGGV	29	4.90
NA (2020)_MLG6	N America	DT	PDSSSSSRGI	DR	TPIVGLSQIPA	SPEDGTASTATTVQM	FGG	AL	YQ	AL	SPYV	LFTFV	HARSGG	SVL	LVSGGGV	DTPDSSSSSRGIDRTPIVGLSQIPASPEDGTASTATTVQMFGGALYQALSPYVLFTFVHARSGGSVLLVSGGGV	27	4.50
NA (2020)_MLG7	N America	DT	PDSSSSSRGI	DR	TPIVGLSQIPA	SPEDGTASTATTVQM	FGG	AL	YQ	AP	SLCV	LFTFV	QARSGG	SVS	LVSGDGV	DTPDSSSSSRGIDRTPIVGLSQIPASPEDGTASTATTVQMFGGALYQAPSLCVLFTFVQARSGGSVSLVSGDGV	23	3.90
NA (2020)_MLG8	N America	DT	PDSSSSSKRI	DR	TPIVGLSQIPA	SPEDGTASTATTVQM	FGG	AL	YQ	AL	SPYV	LFTFV	QARSGG	SVL	LVSGGGV	DTPDSSSSSKRIDRTPIVGLSQIPASPEDGTASTATTVQMFGGALYQALSPYVLFTFVQARSGGSVLLVSGGGV	21	3.50
NA (2020)_MLG9	N America	DT	PDSSSSSRGI	ER	TPIVGLSQIPA	SLEDGTASTATTVQM	FGG	AL	YQ	AP	SPYV	LLTFV	QARSGG	SLS	LVSGDGV	DTPDSSSSSRGIERTPIVGLSQIPASLEDGTASTATTVQMFGGALYQAPSPYVLLTFVQARSGGSLSLVSGDGV	16	2.70
NA (2020)_MLG10	N America	DT	PDSSSSSKRI	DR	TPIVGLSQVPA	SPEDGTASTATTVQM	FGG	AL	YQ	AL	SPYV	LFTFV	QARSGG	SVL	LVSGGGV	DTPDSSSSSKRIDRTPIVGLSQVPASPEDGTASTATTVQMFGGALYQALSPYVLFTFVQARSGGSVLLVSGGGV	16	2.70
NA (2020)_MLG11	N America	DT	PDSSLSSRGI	DR	TPIVGLSQVPA	SPEDGTASTATTVQM	FGG	AL	YQ	AL	SPYV	LFTFV	QARSGG	SVL	LVSGGGV	DTPDSSLSSRGIDRTPIVGLSQVPASPEDGTASTATTVQMFGGALYQALSPYVLFTFVQARSGGSVLLVSGGGV	11	1.80
NA (2020)_MLG12	N America	DT	PDSSSSSRGI	DR	TPIVGLSQIPA	SPEDGTASTATTVQM	FGG	AL	YQ	AP	SPYV	LFTFV	QARSGG	SVL	LVSGDGV	DTPDSSSSSRGIDRTPIVGLSQIPASPEDGTASTATTVQMFGGALYQAPSPYVLFTFVQARSGGSVLLVSGDGV	8	1.30
NA (2020)_MLG13	N America	DT	PDSSLSSRGI	DR	TPIVGLSQIPA	SPEDGTASTATTVQM	FGG	AL	YQ	AL	SPYV	LFTFV	QARSGG	SVL	LVSGGGV	DTPDSSLSSRGIDRTPIVGLSQIPASPEDGTASTATTVQMFGGALYQALSPYVLFTFVQARSGGSVLLVSGGGV	6	1.00
NA (2020)_MLG14	N America	DT	PDSSSSSRGI	DR	IPIVGLSQIPA	SPEDGTASTATTVQM	FGG	AL	YQ	AL	SPYV	LFTFV	QARSGG	SVL	LVSGGGV	DTPDSSSSSRGIDRIPIVGLSQIPASPEDGTASTATTVQMFGGALYQALSPYVLFTFVQARSGGSVLLVSGGGV	5	0.80
NA (2020)_MLG15	N America	DT	PDSSSSSRGI	DR	TPIVGLSQIPA	SPEDGTASTATTVQM	FGG	AL	YQ	AP	SPYV	LFTFV	QARSGG	SVS	LVSGDGV	DTPDSSSSSRGIDRTPIVGLSQIPASPEDGTASTATTVQMFGGALYQAPSPYVLFTFVQARSGGSVSLVSGDGV	4	0.70
NA (2020)_MLG16	N America	DT	PDSSSSSKRI	DR	TPIVGLSQIPA	SPEDGTASTATTVQM	FGG	AF	YQ	AL	SPYV	LFTFV	QARSGG	SVL	LVSGGGV	DTPDSSSSSKRIDRTPIVGLSQIPASPEDGTASTATTVQMFGGAFYQALSPYVLFTFVQARSGGSVLLVSGGGV	2	0.30
NA (2020)_MLG17	N America	DT	PDSSSSSRGI	DR	TPIVGLSQIPA	SPEDGTASTATTVQM	FGG	AL	YQ	AL	SPYV	LFTFV	QARSGG	SVL	LVSGDGV	DTPDSSSSSRGIDRTPIVGLSQIPASPEDGTASTATTVQMFGGALYQALSPYVLFTFVQARSGGSVLLVSGDGV	2	0.30
NA (2020)_MLG18	N America	GT	PDSSSSSRGI	DR	TPIVGLSQIPA	SPEDGTASTATTVQM	FGG	AL	YQ	AL	SPYV	LFTFV	QARSGG	SVL	LVSGGGV	GTPDSSSSSRGIDRTPIVGLSQIPASPEDGTASTATTVQMFGGALYQALSPYVLFTFVQARSGGSVLLVSGGGV	2	0.30
NA (2020)_MLG19	N America	DT	PDSSSSSRGI	DR	IPIVGLSQIPA	SPEDGTASTATTVQM	FGG	AL	YQ	AP	SPYV	LFTFV	HARSGG	SVL	LVSGGGV	DTPDSSSSSRGIDRIPIVGLSQIPASPEDGTASTATTVQMFGGALYQAPSPYVLFTFVHARSGGSVLLVSGGGV	2	0.30
NA (2020)_MLG20	N America	DT	PDSSSSSRGI	ER	TPIVGLSQIPA	SLEDGTASTATTVQM	FGG	AL	YQ	AP	SPYV	LLTFL	QARSGG	SLS	LVSGDGV	DTPDSSSSSRGIERTPIVGLSQIPASLEDGTASTATTVQMFGGALYQAPSPYVLLTFLQARSGGSLSLVSGDGV	2	0.30
NA (2020)_MLG21	N America	DT	PDSSSSSRGI	DR	TPIVGLSQIPA	SPEDGTASTATTVQM	FGG	AL	YQ	AP	SPYV	LFTFV	HARSGG	SVL	LVSGGGV	DTPDSSSSSRGIDRTPIVGLSQIPASPEDGTASTATTVQMFGGALYQAPSPYVLFTFVHARSGGSVLLVSGGGV	2	0.30
NA (2020)_MLG22	N America	DT	PDSSSSSKRI	DR	TPIVGLSQIPA	SPEDGIASTATTVQM	FSG	AL	YQ	AL	SPYV	LFTFV	QARSGG	SVL	LVSGGGV	DTPDSSSSSKRIDRTPIVGLSQIPASPEDGIASTATTVQMFSGALYQALSPYVLFTFVQARSGGSVLLVSGGGV	2	0.30
NA (2020)_MLG23	N America	DT	PDSSSSSRGI	DR	TPIIGLSQIPA	SPEDGTASTATTVQM	FGG	AF	YQ	AP	SPYV	LFTFV	QARSGG	SVL	LVSGDGV	DTPDSSSSSRGIDRTPIIGLSQIPASPEDGTASTATTVQMFGGAFYQAPSPYVLFTFVQARSGGSVLLVSGDGV	2	0.30
NA (2020)_MLG24	N America	DT	PDSSSLSRGI	DR	TPIVGLSQIPA	SPEDGTASTATTVQM	YGG	AF	YQ	AP	SPYV	LFTFV	QARSVG	SVS	LVSGDGV	DTPDSSSLSRGIDRTPIVGLSQIPASPEDGTASTATTVQMYGGAFYQAPSPYVLFTFVQARSVGSVSLVSGDGV	2	0.30
NA (2020)_MLG25	N America	DT	PDSSSSSSGI	DR	IPIVGLSQIPA	SPEDGTASTATTVQM	FGG	AL	YQ	AL	SPYV	LFTFV	HARSGG	SVL	LVSGGGV	DTPDSSSSSSGIDRIPIVGLSQIPASPEDGTASTATTVQMFGGALYQALSPYVLFTFVHARSGGSVLLVSGGGV	1	0.20
NA (2020)_MLG26	N America	DT	LDSSSSSRGI	DR	TPIVGLSQIPA	SPEDGTASTATTVQM	FGG	AL	YQ	AP	SPYV	LFTFV	QARSGG	SVL	LVSGDGV	DTLDSSSSSRGIDRTPIVGLSQIPASPEDGTASTATTVQMFGGALYQAPSPYVLFTFVQARSGGSVLLVSGDGV	1	0.20
NA (2020)_MLG27	N America	DT	PDSSSSSRGI	DR	TPIVGLSQIPA	SPEDGTASTATTVQM	FGG	AL	YQ	AP	SPYV	LFTFV	QARSGG	SVL	LVSGGGV	DTPDSSSSSRGIDRTPIVGLSQIPASPEDGTASTATTVQMFGGALYQAPSPYVLFTFVQARSGGSVLLVSGGGV	1	0.20
NA (2020)_MLG28	N America	DT	PDSSSLSRGI	DR	TPIVGLSQIPA	SPEDGTASTATTVQM	YGG	AL	YQ	AP	SPYV	LFTFV	QARSGG	SVS	LVSGDGV	DTPDSSSLSRGIDRTPIVGLSQIPASPEDGTASTATTVQMYGGALYQAPSPYVLFTFVQARSGGSVSLVSGDGV	1	0.20
NA (2020)_MLG29	N America	DT	PDSSSSSRGI	ER	TPIVGLSQIPA	SLEDGTASTATTVQM	FGG	AF	YQ	AP	SPYV	LLTFV	QARSGG	SLS	LVSGDGV	DTPDSSSSSRGIERTPIVGLSQIPASLEDGTASTATTVQMFGGAFYQAPSPYVLLTFVQARSGGSLSLVSGDGV	1	0.20
NA (2020)_MLG30	N America	DT	PDSSSSSRGI	DR	IPIVGLSQIPA	SPEDGTASTATTVQM	FGG	AL	YQ	AL	SPYV	LFTFV	QARSGG	LVL	LVSGGGV	DTPDSSSSSRGIDRIPIVGLSQIPASPEDGTASTATTVQMFGGALYQALSPYVLFTFVQARSGGLVLLVSGGGV	1	0.20
NA (2020)_MLG31	N America	DT	PDSSSSSRGI	DR	TPIVGLSQIPA	SPEDGTASTATTVQM	FGG	AL	YQ	AP	SLYV	LFTFV	QARSGG	SVS	LVSGDGV	DTPDSSSSSRGIDRTPIVGLSQIPASPEDGTASTATTVQMFGGALYQAPSLYVLFTFVQARSGGSVSLVSGDGV	1	0.20
NA (2020)_MLG32	N America	DT	PDSSSSSRGI	DR	TPIVGLSQIPA	SPEDGTASTATTVQM	FGG	AL	YQ	AP	SPYV	LFTFV	HARSGG	SVL	LVSGDGV	DTPDSSSSSRGIDRTPIVGLSQIPASPEDGTASTATTVQMFGGALYQAPSPYVLFTFVHARSGGSVLLVSGDGV	1	0.20
NA (2020)_MLG33	N America	DT	PDSSSSSRGI	DR	TPIVGLSQIPA	SPEDGTASTATTVQM	FGG	AL	YQ	VL	SPYV	LFTFV	HARSGG	SVL	LVSGGGV	DTPDSSSSSRGIDRTPIVGLSQIPASPEDGTASTATTVQMFGGALYQVLSPYVLFTFVHARSGGSVLLVSGGGV	1	0.20
NA (2020)_MLG34	N America	DT	PDSSSLSRGI	DR	TPIVGLSQIPA	SPEDGTASTATTVQM	YGG	AL	YQ	AP	SPYV	LFTFV	QARSVG	SES	LVSGDGV	DTPDSSSLSRGIDRTPIVGLSQIPASPEDGTASTATTVQMYGGALYQAPSPYVLFTFVQARSVGSESLVSGDGV	1	0.20
NA (2020)_MLG35	N America	DT	PDSSSSSRGI	DR	TPIVGLSQIPA	SPEDGTASTATTVQM	FGG	AF	YQ	AL	SPYV	LFTFV	HARSGG	SVL	LVSGGGV	DTPDSSSSSRGIDRTPIVGLSQIPASPEDGTASTATTVQMFGGAFYQALSPYVLFTFVHARSGGSVLLVSGGGV	1	0.20
NA (2020)_MLG36	N America	DT	PDSSSLSRGI	DR	TPIVGLSQIPA	SPEDGTASTATTVQM	YGG	AL	YQ	AP	SPFV	LFTFV	QARSVG	SVS	LVSGDGV	DTPDSSSLSRGIDRTPIVGLSQIPASPEDGTASTATTVQMYGGALYQAPSPFVLFTFVQARSVGSVSLVSGDGV	1	0.20
NA (2020)_MLG37	N America	DM	PDSSSSSKRI	DR	TPIVGLSQIPA	SPEDGTASTATTVQM	FGG	AL	YQ	AL	SPYV	LFTFV	QARSGG	SVL	LVSGGGV	DMPDSSSSSKRIDRTPIVGLSQIPASPEDGTASTATTVQMFGGALYQALSPYVLFTFVQARSGGSVLLVSGGGV	1	0.20
NA (2020)_MLG38	N America	DT	PDSSSLSRGI	DR	TPIVGLSQIPA	SPEDGTASTATTVQV	YGG	AL	YQ	AP	SPYV	LFTFV	QARSVG	SVS	LVSGDGV	DTPDSSSLSRGIDRTPIVGLSQIPASPEDGTASTATTVQVYGGALYQAPSPYVLFTFVQARSVGSVSLVSGDGV	1	0.20
NA (2020)_MLG39	N America	DT	PDSSSSSKRI	DR	TPIVGLSQIPA	SPEEGTASTATTVQM	FGG	AL	YQ	AL	SPYV	LFTFV	QARSGG	SVL	LVSGGGV	DTPDSSSSSKRIDRTPIVGLSQIPASPEEGTASTATTVQMFGGALYQALSPYVLFTFVQARSGGSVLLVSGGGV	1	0.20

NA (2020)_MLG40	N America	DT	PDSSSSSRGI	DR	IPIVGLSQIPA	SPEDGTASTATTVQM	FGG	AF	YQ	AL	SPYV	LFTFV	HARSGG	SVL	LVSGGGV	DTPDSSSSSRGIDRIPIVGLSQIPASPEDGTASTATTVQMFGGAFYQALSPYVLFTFVHARSGGSVLLVSGGGV	1	0.20
NA (2020)_MLG41	N America	DT	PDSSSSSRGI	DR	TPIVGLSQIPA	SPEDGTASIATTVQM	FGG	AL	YQ	AP	SLCV	LFTFV	QARSGG	SVS	LVSGDGV	DTPDSSSSSRGIDRTPIVGLSQIPASPEDGTASIATTVQMFGGALYQAPSLCVLFTFVQARSGGSVSLVSGDGV	1	0.20
NA (2020)_MLG42	N America	DT	PDSSSSSRGI	DC	TPIIGLSQIPA	SPEDGTASTATTVQI	FGG	AF	YQ	AP	SPYV	LFTFV	QARSGG	SVL	LVSGDGV	DTPDSSSSSRGIDCTPIIGLSQIPASPEDGTASTATTVQIFGGAFYQAPSPYVLFTFVQARSGGSVLLVSGDGV	1	0.20
NA (2020)_MLG43	N America	DT	PDYSSSSRGI	DR	IPIVGLSQIPA	SPEDGTASTATTVQM	FGG	AL	YQ	AL	SPYV	LFTFV	HARSGG	SVL	LVSGGGV	DTPDYSSSSRGIDRIPIVGLSQIPASPEDGTASTATTVQMFGGALYQALSPYVLFTFVHARSGGSVLLVSGGGV	1	0.20
NA (2020)_MLG44	N America	DT	PDSSSSSKRI	DR	TPIVGLSQIPA	SPEDGTASTATTVQM	FGG	AL	YQ	AP	SPYV	LFTFV	QARSGG	SVL	LVSGGGV	DTPDSSSSSKRIDRTPIVGLSQIPASPEDGTASTATTVQMFGGALYQAPSPYVLFTFVQARSGGSVLLVSGGGV	1	0.20
NA (2020)_MLG45	N America	DT	PDSSSSNRGI	DR	TPIVGLSQIPA	SPEDGTASTATTVQM	FGG	AL	YQ	AP	SPYV	LFTFV	QARSGG	SVS	LVSGDGV	DTPDSSSSNRGIDRTPIVGLSQIPASPEDGTASTATTVQMFGGALYQAPSPYVLFTFVQARSGGSVSLVSGDGV	1	0.20
NA (2020)_MLG46	N America	DT	PDSSSSSRGI	DR	IPIVGLSQIPA	SPEDGTASTATTVQM	FGG	AL	YQ	AL	SPYV	LFTFV	HARSGG	LVL	FVSGGGV	DTPDSSSSSRGIDRIPIVGLSQIPASPEDGTASTATTVQMFGGALYQALSPYVLFTFVHARSGGLVLFVSGGGV	1	0.20
NA (2020)_MLG47	N America	DT	PDSSSSSKRI	DR	TPIVGLSQIPA	SPEDGTASTATTVQM	FGG	AL	YQ	AP	SPYV	LFTFV	QARSGG	SVL	LVSGDGV	DTPDSSSSSKRIDRTPIVGLSQIPASPEDGTASTATTVQMFGGALYQAPSPYVLFTFVQARSGGSVLLVSGDGV	1	0.20
NA (2020)_MLG48	N America	DT	PDSSSSSRGI	DR	TPIVGLSQVSA	SPEDGTASTATTVQM	FGG	AF	YQ	AP	SPYV	LFTFV	QARSGV	SVL	LVSGDGV	DTPDSSSSSRGIDRTPIVGLSQVSASPEDGTASTATTVQMFGGAFYQAPSPYVLFTFVQARSGVSVLLVSGDGV	1	0.20
NA (2020)_MLG49	N America	DT	PDSSSLSRGI	ER	TPIVGLSQIPA	SLEDGTASTATTVQM	YGG	AL	YQ	AP	SPYV	LLTFV	QARSVG	SLS	LVSGDGV	DTPDSSSLSRGIERTPIVGLSQIPASLEDGTASTATTVQMYGGALYQAPSPYVLLTFVQARSVGSLSLVSGDGV	1	0.20
NA (2020)_MLG50	N America	DT	PDSSSLSRGI	DR	TPIVGLSQIPA	SPEDGTASTATTVQM	YGG	AL	YQ	AP	SPYV	LFTFV	QAKSVG	SVS	LVSGDGV	DTPDSSSLSRGIDRTPIVGLSQIPASPEDGTASTATTVQMYGGALYQAPSPYVLFTFVQAKSVGSVSLVSGDGV	1	0.20
NA (2020)_MLG51	N America	DT	PDSSSLSRGI	DR	TPIVGLSQIPA	SPEDGTASTATTVQM	YGG	AL	YQ	AP	SPYV	LFTFL	QARSVG	SVS	LVSGDGV	DTPDSSSLSRGIDRTPIVGLSQIPASPEDGTASTATTVQMYGGALYQAPSPYVLFTFLQARSVGSVSLVSGDGV	1	0.20
NA (2020)_MLG52	N America	DM	PDSSSSSKRI	DR	TPIVGLSQIPA	SPEDGTASTATTVQM	FGG	AL	YQ	AL	SPYV	LLTFV	QARSGG	SVL	LVSGGGV	DMPDSSSSSKRIDRTPIVGLSQIPASPEDGTASTATTVQMFGGALYQALSPYVLLTFVQARSGGSVLLVSGGGV	1	0.20
NA (2020)_MLG53	N America	DT	LDSSSSSRGI	DR	IPIVGLSQIPA	SPEDGTASTATTVQM	FGG	AL	YQ	AL	SPYV	LFTFV	HARSGG	SVL	LVSGGGV	DTLDSSSSSRGIDRIPIVGLSQIPASPEDGTASTATTVQMFGGALYQALSPYVLFTFVHARSGGSVLLVSGGGV	1	0.20
NA (2020)_MLG54	N America	DT	PDSSSSSRGI	DR	TPIVGLSQIPA	SPEDGTASTATTVQM	FGG	AF	YQ	AP	SPYV	LFTFV	QARSGV	SVL	LVSGDGV	DTPDSSSSSRGIDRTPIVGLSQIPASPEDGTASTATTVQMFGGAFYQAPSPYVLFTFVQARSGVSVLLVSGDGV	1	0.20
NA (2020)_MLG55	N America	DT	PDSSSSSRGI	DR	TPIVGLSQIPA	SPEDGTASTATSVQM	FGG	AL	YQ	AL	SPYV	LFTFV	QARSGS	SVL	LVSGGGV	DTPDSSSSSRGIDRTPIVGLSQIPASPEDGTASTATSVQMFGGALYQALSPYVLFTFVQARSGSSVLLVSGGGV	1	0.20
NA (2020)_MLG56	N America	DT	PDSSSLSRGI	DR	TPIVGLSQIPA	SPEDGTASTGTTVQM	YGG	AL	YQ	AP	SPYV	LFTFV	QARSVG	SVS	LVSGDGV	DTPDSSSLSRGIDRTPIVGLSQIPASPEDGTASTGTTVQMYGGALYQAPSPYVLFTFVQARSVGSVSLVSGDGV	1	0.20
NA (2020)_MLG57	N America	DT	PDSSSSSRGI	DR	TPIVGLSQIPA	SPEDGTASTATTVQM	FGG	AL	YQ	AL	SPYV	LFTFV	QARSGS	AVL	LVSGGGV	DTPDSSSSSRGIDRTPIVGLSQIPASPEDGTASTATTVQMFGGALYQALSPYVLFTFVQARSGSAVLLVSGGGV	1	0.20
NA (2020)_MLG58	N America	DT	PDSSSSSRGI	DR	TPIVGLSQIPA	SPEDGTASTATTVQM	FGG	AL	YQ	AL	SPYV	LFTFV	QARSGS	SVL	LISGGGV	DTPDSSSSSRGIDRTPIVGLSQIPASPEDGTASTATTVQMFGGALYQALSPYVLFTFVQARSGSSVLLISGGGV	1	0.20
NA (2020)_MLG59	N America	DT	PDSSSLSRGI	DR	TPIVGLSQIPA	SPEDGTASTATTVQM	YGG	AL	YQ	AP	SPYV	LFTFV	QARSVG	PVS	LVSGDGV	DTPDSSSLSRGIDRTPIVGLSQIPASPEDGTASTATTVQMYGGALYQAPSPYVLFTFVQARSVGPVSLVSGDGV	1	0.20
NA (2020)_MLG60	N America	DT	PDSSSLSRGI	DR	TPIVGLSRIPA	SPEDGTASTATTVQM	YGG	AL	YQ	AP	SPYV	LFTFV	QARSVG	SVS	LVSGDGV	DTPDSSSLSRGIDRTPIVGLSRIPASPEDGTASTATTVQMYGGALYQAPSPYVLFTFVQARSVGSVSLVSGDGV	1	0.20
NA (2020)_MLG61	N America	DT	PDSSSSSRGI	DR	TPIVGLSQVPA	SPEDGTASTATTVQM	FGG	AL	YQ	AP	SPYV	LFTFV	QARSGG	SVS	LVSGDGV	DTPDSSSSSRGIDRTPIVGLSQVPASPEDGTASTATTVQMFGGALYQAPSPYVLFTFVQARSGGSVSLVSGDGV	1	0.20
NA (2020)_MLG62	N America	DT	PDYSSSSRGI	DR	IPIVGLSQVPA	SPEDGTASTATTVQM	FGG	AL	YQ	AL	SPYV	LFTFV	HARSGG	SVL	LVSGGGV	DTPDYSSSSRGIDRIPIVGLSQVPASPEDGTASTATTVQMFGGALYQALSPYVLFTFVHARSGGSVLLVSGGGV	1	0.20
NA (2020)_MLG63	N America	DT	PDSSLSSRGI	DR	TLIVGLSQVPA	SPEDGTASTATTVQM	FGG	AL	YQ	AL	SPYV	LFTFV	QARSGG	SVL	LVSGGGV	DTPDSSLSSRGIDRTLIVGLSQVPASPEDGTASTATTVQMFGGALYQALSPYVLFTFVQARSGGSVLLVSGGGV	1	0.20
NA (2020)_MLG64	N America	DT	PDSSSSSKRI	DR	TPIVGLSQVPA	SPEDGIASTATTVQM	FSG	AL	YQ	AL	SPYV	LFTFV	QARSGG	SVL	LVSGGGV	DTPDSSSSSKRIDRTPIVGLSQVPASPEDGIASTATTVQMFSGALYQALSPYVLFTFVQARSGGSVLLVSGGGV	1	0.20
NA (2020)_MLG65	N America	DT	PDSSSSSRGI	DR	TPIVGLSQVPA	SPEDGTASTATTVQM	FGG	AL	YQ	AL	SPYV	LFTFV	QARSGG	SVL	LVSGGGV	DTPDSSSSSRGIDRTPIVGLSQVPASPEDGTASTATTVQMFGGALYQALSPYVLFTFVQARSGGSVLLVSGGGV	1	0.20
NA (2020)_MLG66	N America	DT	PDSSLSSRGI	DR	TPIVGLSQVPA	SPEDGTASTATTVQM	FGG	AF	YQ	AL	SPYV	LFTFV	QARSGG	SVL	LVSGGGV	DTPDSSLSSRGIDRTPIVGLSQVPASPEDGTASTATTVQMFGGAFYQALSPYVLFTFVQARSGGSVLLVSGGGV	1	0.20
NA (2020)_MLG67	N America	DT	PDSSSSSKRI	DR	TPIVGLSQVPA	SPEDGIASTATTVQM	FSG	AL	YQ	AL	SPYV	LFTFV	QASSGG	SVL	LVSGGGV	DTPDSSSSSKRIDRTPIVGLSQVPASPEDGIASTATTVQMFSGALYQALSPYVLFTFVQASSGGSVLLVSGGGV	1	0.20
NA (2020)_MLG68	N America	DT	PDSSSSSRGI	DR	IPIVGLSQVPA	SPEDGTASTATTVQM	FGG	AL	YQ	AL	SPYV	LFTFV	HARSGG	SVL	LVSGGGV	DTPDSSSSSRGIDRIPIVGLSQVPASPEDGTASTATTVQMFGGALYQALSPYVLFTFVHARSGGSVLLVSGGGV	1	0.20
NA (2020)_MLG69	N America	DT	PDSSSSSRGI	DR	IPIVGLSQVPA	SPEDGTASTATTVQM	FGG	AL	YQ	AL	SPYV	LFTFV	HARSGG	LVL	LVSGGGV	DTPDSSSSSRGIDRIPIVGLSQVPASPEDGTASTATTVQMFGGALYQALSPYVLFTFVHARSGGLVLLVSGGGV	1	0.20
NA (2021)_MLG70	N America	DT	PDSSSSSKRI	DR	TPIVGLSQIDA	PEDGTASTATTVQM	FGG	AL	YQ	AL	SPYV	LFTFV	QARSGG	SVL	LVSGGGV	DTPDSSSSSKRIDRTPIVGLSQIDAPEDGTASTATTVQMFGGALYQALSPYVLFTFVQARSGGSVLLVSGGGV	1809	44.33
NA (2021)_MLG71	N America	DT	PDSSSSSRGI	DR	IPIVGLSQIDA	PEDGTASTATTVQM	FGG	AL	YQ	AL	SPYV	LFTFV	HARSGG	LVL	LVSGGGV	DTPDSSSSSRGIDRIPIVGLSQIDAPEDGTASTATTVQMFGGALYQALSPYVLFTFVHARSGGLVLLVSGGGV	926	22.69
NA (2021)_MLG72	N America	DT	PDSSSSSRGI	DR	IPIVGLSQIDA	PEDGTASTATTVQM	FGG	AL	YQ	AL	SPYV	LFTFV	HARSGG	SVL	LVSGGGV	DTPDSSSSSRGIDRIPIVGLSQIDAPEDGTASTATTVQMFGGALYQALSPYVLFTFVHARSGGSVLLVSGGGV	379	9.29
NA (2021)_MLG73	N America	DT	PDSSLSSRGI	DR	TPIVGLSQIDA	PEDGTASTATTVQM	FGG	AL	YQ	AL	SPYV	LFTFV	QARSGG	SVL	LVSGGGV	DTPDSSLSSRGIDRTPIVGLSQIDAPEDGTASTATTVQMFGGALYQALSPYVLFTFVQARSGGSVLLVSGGGV	225	5.51
NA (2021)_MLG74	N America	DT	PDSSSLSRGI	DR	TPIVGLSQIDA	PEDGTASTATTVQM	YGG	AL	YQ	AP	SPYV	LFTFV	QARSVG	SVS	LVSGGGV	DTPDSSSLSRGIDRTPIVGLSQIDAPEDGTASTATTVQMYGGALYQAPSPYVLFTFVQARSVGSVSLVSGGGV	99	2.43
NA (2021)_MLG75	N America	DT	PDSSSSSRGI	DR	TPIVGLSQIDA	PEDGTASTATTVQM	FGG	AL	YQ	AL	SPYV	LFTFV	QARSGG	SVL	LVSGGGV	DTPDSSSSSRGIDRTPIVGLSQIDAPEDGTASTATTVQMFGGALYQALSPYVLFTFVQARSGGSVLLVSGGGV	47	1.15
NA (2021)_MLG76	N America	DT	PDSSSSSRGI	DR	IPIVGLSQIDA	PEDGTASTATTVQM	FGG	AL	YQ	AL	SPYV	LFTFL	HARSGG	LVL	LVSGGGV	DTPDSSSSSRGIDRIPIVGLSQIDAPEDGTASTATTVQMFGGALYQALSPYVLFTFLHARSGGLVLLVSGGGV	43	1.05
NA (2021)_MLG77	N America	DT	PDSSSSSKRI	DR	TPIVGLSQIDA	PEDGTASTATTVQM	FGG	AF	YQ	AL	SPYV	LFTFV	QARSGG	SVL	LVSGGGV	DTPDSSSSSKRIDRTPIVGLSQIDAPEDGTASTATTVQMFGGAFYQALSPYVLFTFVQARSGGSVLLVSGGGV	43	1.05
NA (2021)_MLG78	N America	DT	PDSSSSSKRI	DR	TPIVGLSQIDA	PEDGTASTATTVQM	FGG	AL	YQ	AL	SPYV	LFTFV	QARSGG	SVL	LVSGGGF	DTPDSSSSSKRIDRTPIVGLSQIDAPEDGTASTATTVQMFGGALYQALSPYVLFTFVQARSGGSVLLVSGGGF	38	0.93
NA (2021)_MLG79	N America	DT	PDSLSSSRGI	DR	IPIVGLSQIDA	PEDGTASTATTVQM	FGG	AL	YQ	AL	SPYV	LFTFV	HARSGG	LVL	LVSGGGV	DTPDSLSSSRGIDRIPIVGLSQIDAPEDGTASTATTVQMFGGALYQALSPYVLFTFVHARSGGLVLLVSGGGV	25	0.61
NA (2021)_MLG80	N America	DT	PDSSSSSKRI	DR	TPIVGLSQIDA	PEDGTASTATTVQM	FGG	AL	YQ	AL	SPYV	LFTFV	QARSGG	SLL	LVSGGGV	DTPDSSSSSKRIDRTPIVGLSQIDAPEDGTASTATTVQMFGGALYQALSPYVLFTFVQARSGGSLLLVSGGGV	23	0.56
NA (2021)_MLG81	N America	DT	PDSSSSSRGI	DR	IPIVGLSQIDA	SEDGTASTATTVQM	FGG	AL	YQ	AL	SPYV	LFTFV	HARSGG	SVL	LVSGGGV	DTPDSSSSSRGIDRIPIVGLSQIDASEDGTASTATTVQMFGGALYQALSPYVLFTFVHARSGGSVLLVSGGGV	20	0.49
NA (2021)_MLG82	N America	DT	PDSSSSSRGI	DR	TPIVGLSQIDA	PEDGTASTATTVQM	FGG	AL	YQ	AL	SPYV	LFTFV	HARSGG	SVL	LVNGGGV	DTPDSSSSSRGIDRTPIVGLSQIDAPEDGTASTATTVQMFGGALYQALSPYVLFTFVHARSGGSVLLVNGGGV	18	0.44
NA (2021)_MLG83	N America	DT	PDSSSSSMGI	DR	TPIVGLSQIDA	PEDGTASTATTVQM	FGG	AL	YQ	AL	SPYV	LFTFV	QARSGG	SVL	LVSGGGV	DTPDSSSSSMGIDRTPIVGLSQIDAPEDGTASTATTVQMFGGALYQALSPYVLFTFVQARSGGSVLLVSGGGV	13	0.32
NA (2021)_MLG84	N America	DT	PDSSSSSRGI	DR	IPIVGLSQIDA	PEDGTASTATTVQM	FGG	AF	YQ	AL	SPYV	LFTFV	HARSGG	LVL	LVSGGGV	DTPDSSSSSRGIDRIPIVGLSQIDAPEDGTASTATTVQMFGGAFYQALSPYVLFTFVHARSGGLVLLVSGGGV	12	0.29
NA (2021)_MLG85	N America	DT	PDSSSSSRGI	DR	TPIVGLSQIDA	PEDGTASTATTVQM	FGG	AL	YQ	AL	SPYV	LFTFV	QARSGG	SVL	LVNGGGV	DTPDSSSSSRGIDRTPIVGLSQIDAPEDGTASTATTVQMFGGALYQALSPYVLFTFVQARSGGSVLLVNGGGV	12	0.29
NA (2021)_MLG86	N America	DT	PDSSLSSRGI	DR	TPIVGLSQIDA	PEDGTASTATTVQM	FGG	AF	YQ	AL	SPYV	LFTFV	QARSGG	SVL	LVSGGGV	DTPDSSLSSRGIDRTPIVGLSQIDAPEDGTASTATTVQMFGGAFYQALSPYVLFTFVQARSGGSVLLVSGGGV	12	0.29
NA (2021)_MLG87	N America	DT	PDSSSSSRGI	DR	TPIVGLSQIDA	PEDGTASTATTVQM	FGG	AL	YQ	AL	SPYV	LFTFV	HARSGG	SVL	LVSGGGV	DTPDSSSSSRGIDRTPIVGLSQIDAPEDGTASTATTVQMFGGALYQALSPYVLFTFVHARSGGSVLLVSGGGV	10	0.25
NA (2021)_MLG88	N America	DT	PDSSSSSRGI	DR	IPIVGLSQIDA	PEDGTASTATTVQM	FGG	AL	YQ	AL	SPYV	LFTFV	HARSGG	SVL	LVNGGGV	DTPDSSSSSRGIDRIPIVGLSQIDAPEDGTASTATTVQMFGGALYQALSPYVLFTFVHARSGGSVLLVNGGGV	8	0.20
NA (2021)_MLG89	N America	DT	PDSSSSSRGI	DR	IPIVGLSQIDA	PEDGTASTATTVQM	FGG	AF	YQ	AL	SPYV	LFTFV	HARSGG	SVL	LVSGGGV	DTPDSSSSSRGIDRIPIVGLSQIDAPEDGTASTATTVQMFGGAFYQALSPYVLFTFVHARSGGSVLLVSGGGV	8	0.20
NA (2021)_MLG90	N America	DT	PDSSSSSRGI	DR	IPIVGLSQIDA	LEDGTASTATTVQM	FGG	AL	YQ	AL	SPYV	LFTFV	HARSGG	SVL	LVSGGGV	DTPDSSSSSRGIDRIPIVGLSQIDALEDGTASTATTVQMFGGALYQALSPYVLFTFVHARSGGSVLLVSGGGV	8	0.20
NA (2021)_MLG91	N America	DT	PDSSSSSRGI	DR	IPIVGLSQIDA	PEDGTASTATTVQM	FGG	AL	YQ	AL	SPYV	LFTFV	QARSGG	SVL	LVSGGGV	DTPDSSSSSRGIDRIPIVGLSQIDAPEDGTASTATTVQMFGGALYQALSPYVLFTFVQARSGGSVLLVSGGGV	7	0.17
NA (2021)_MLG92	N America	DT	PDSSSSSRGI	DR	IPIVGLSQIDA	PEDGTASTATTVQM	FGG	AL	YQ	AF	SPYV	LFTFV	HARSGG	LVL	LVSGGGV	DTPDSSSSSRGIDRIPIVGLSQIDAPEDGTASTATTVQMFGGALYQAFSPYVLFTFVHARSGGLVLLVSGGGV	7	0.17
NA (2021)_MLG93	N America	DT	PDSLSSSRGI	DR	IPIVGLSQIDA	PEDGTASTATTVQM	FGG	AL	YQ	AL	SPYV	LFTFV	HARSGG	SVL	LVSGGGV	DTPDSLSSSRGIDRIPIVGLSQIDAPEDGTASTATTVQMFGGALYQALSPYVLFTFVHARSGGSVLLVSGGGV	7	0.17
NA (2021)_MLG94	N America	DT	PDSSSSNRGI	DR	TPIVGLSQIDA	PEDGTASTATTVQM	FGG	AL	YQ	AP	SPYV	LFTFV	QARSGG	SVS	LVSGDGV	DTPDSSSSNRGIDRTPIVGLSQIDAPEDGTASTATTVQMFGGALYQAPSPYVLFTFVQARSGGSVSLVSGDGV	6	0.15
NA (2021)_MLG95	N America	DT	LDSSLSSRGI	DR	TPIVGLSQIDA	PEDGTASTATTVQM	FGG	AL	YQ	AL	SPYV	LFTFV	QARSGG	SVL	LVSGGGV	DTLDSSLSSRGIDRTPIVGLSQIDAPEDGTASTATTVQMFGGALYQALSPYVLFTFVQARSGGSVLLVSGGGV	6	0.15
NA (2021)_MLG96	N America	DT	PDSSSSSRGI	DR	IPIVGLSQIDA	PEDGTASTATTVQM	FGG	AL	YQ	AL	SPYV	LFTFV	HDRSGG	SVL	LVSGGGV	DTPDSSSSSRGIDRIPIVGLSQIDAPEDGTASTATTVQMFGGALYQALSPYVLFTFVHDRSGGSVLLVSGGGV	6	0.15
NA (2021)_MLG97	N America	DT	PDSSLSSRGI	DR	TPIVGLSQIDA	PEDGTASTATTVQM	FGG	AL	YQ	AL	SPYV	LFTFV	QARSGG	SVL	LVNGGGV	DTPDSSLSSRGIDRTPIVGLSQIDAPEDGTASTATTVQMFGGALYQALSPYVLFTFVQARSGGSVLLVNGGGV	6	0.15
NA (2021)_MLG98	N America	DT	PDSSLSSRGI	DR	TPIVGLSHIDA	PEDGTASTATTVQM	FGG	AL	YQ	AL	SPYV	LFTFV	QARSGG	SVL	LVSGGGV	DTPDSSLSSRGIDRTPIVGLSHIDAPEDGTASTATTVQMFGGALYQALSPYVLFTFVQARSGGSVLLVSGGGV	6	0.15
NA (2021)_MLG99	N America	DT	LDSSSSSRGI	DR	IPIVGLSQIDA	PEDGTASTATTVQM	FGG	AL	YQ	AL	SPYV	LFTFV	HARSGG	LVL	LVSGGGV	DTLDSSSSSRGIDRIPIVGLSQIDAPEDGTASTATTVQMFGGALYQALSPYVLFTFVHARSGGLVLLVSGGGV	5	0.12
NA (2021)_MLG100	N America	DT	PDSSSSSRGI	DR	TPIVGLSQIDA	PEDGTASTATTVQM	FGG	AL	YQ	AL	SPYV	LFTFV	QARSGG	LVL	LVSGGGV	DTPDSSSSSRGIDRTPIVGLSQIDAPEDGTASTATTVQMFGGALYQALSPYVLFTFVQARSGGLVLLVSGGGV	5	0.12
NA (2021)_MLG101	N America	DT	PDSSSSSRGI	DR	IPIVGLSQIDA	PEDGTASTATTVQM	FGG	AL	YQ	AL	SPYL	LFTFV	HARSGG	LVL	LVSGGGV	DTPDSSSSSRGIDRIPIVGLSQIDAPEDGTASTATTVQMFGGALYQALSPYLLFTFVHARSGGLVLLVSGGGV	5	0.12
NA (2021)_MLG102	N America	DT	PDSSLSSRGI	DR	IPIVGLSQIDA	PEDGTASTATTVQM	FGG	AL	YQ	AL	SPYV	LFTFV	HARSGG	SVL	LVSGGGV	DTPDSSLSSRGIDRIPIVGLSQIDAPEDGTASTATTVQMFGGALYQALSPYVLFTFVHARSGGSVLLVSGGGV	5	0.12
NA (2021)_MLG103	N America	DT	PDSSSSSKRI	DR	TPIVGLSQIDA	PEDGIASTATTVQM	FGG	AL	YQ	AL	SPYV	LFTFV	QARSGG	SVL	LVSGGGV	DTPDSSSSSKRIDRTPIVGLSQIDAPEDGIASTATTVQMFGGALYQALSPYVLFTFVQARSGGSVLLVSGGGV	5	0.12

NA (2021)_MLG104	N America	DT	PYSSSSSKRI	DR	TPIVGLSQIDA	PEDGTASTATTVQM	FGG	AL	YQ	AL	SPYV	LFTFV	QARSGG	SVL	LVSGGGV	DTPYSSSSSKRIDRTPIVGLSQIDAPEDGTASTATTVQMFGGALYQALSPYVLFTFVQARSGGSVLLVSGGGV	5	0.12
NA (2021)_MLG105	N America	DT	PDSLSSSKRI	DR	TPIVGLSQIDA	PEDGTASTATTVQM	FGG	AL	YQ	AL	SPYV	LFTFV	QARSGG	SVL	LVSGGGV	DTPDSLSSSKRIDRTPIVGLSQIDAPEDGTASTATTVQMFGGALYQALSPYVLFTFVQARSGGSVLLVSGGGV	5	0.12
NA (2021)_MLG106	N America	DT	PDSSSSSKRI	DR	TPIVGLSQIDA	LEDGTASTATTVQM	FGG	AL	YQ	AL	SPYV	LFTFV	QARSGG	SVL	LVSGGGV	DTPDSSSSSKRIDRTPIVGLSQIDALEDGTASTATTVQMFGGALYQALSPYVLFTFVQARSGGSVLLVSGGGV	5	0.12
NA (2021)_MLG107	N America	DT	PDSSSPSRGI	DR	IPIVGLSQIDA	PEDGTASTATTVQM	FGG	AL	YQ	AL	SPYV	LFTFV	HARSGG	LVL	LVSGGGV	DTPDSSSPSRGIDRIPIVGLSQIDAPEDGTASTATTVQMFGGALYQALSPYVLFTFVHARSGGLVLLVSGGGV	4	0.10
NA (2021)_MLG108	N America	DT	PDSSSSSRGI	DR	TPIVGLSQIDA	PEDGTASTATTVQM	FGG	AL	YQ	AL	SPYV	LFTFV	HARSGG	LVL	LVSGGGV	DTPDSSSSSRGIDRTPIVGLSQIDAPEDGTASTATTVQMFGGALYQALSPYVLFTFVHARSGGLVLLVSGGGV	4	0.10
NA (2021)_MLG109	N America	DT	PDSSSLSRGI	DR	TPIVGLSQIDA	LEDGTASTATTVQM	YGG	AL	YQ	AP	SPYV	LFTFV	QARSVG	SVS	LVSGGGV	DTPDSSSLSRGIDRTPIVGLSQIDALEDGTASTATTVQMYGGALYQAPSPYVLFTFVQARSVGSVSLVSGGGV	4	0.10
NA (2021)_MLG110	N America	DT	PDSSSSSRGI	DR	TPIVGLSQIDA	PEDGTASTATTVQM	FGG	AL	YQ	AP	SPYV	LFTFV	QARSGG	SVL	LVSGGGV	DTPDSSSSSRGIDRTPIVGLSQIDAPEDGTASTATTVQMFGGALYQAPSPYVLFTFVQARSGGSVLLVSGGGV	4	0.10
NA (2021)_MLG111	N America	DT	PDSSSSSKRI	DR	TPIVGLSQIDA	PEDGTASTATTVQM	FGG	AL	YQ	AL	SPYV	LFTFV	HARSGG	SVL	LVSGGGV	DTPDSSSSSKRIDRTPIVGLSQIDAPEDGTASTATTVQMFGGALYQALSPYVLFTFVHARSGGSVLLVSGGGV	4	0.10
NA (2021)_MLG112	N America	DT	PDSSSSSKRT	DR	TPIVGLSQIDA	PEDGTASTATTVQM	FGG	AL	YQ	AL	SPYV	LFTFV	QARSGG	SVL	LVSGGGV	DTPDSSSSSKRTDRTPIVGLSQIDAPEDGTASTATTVQMFGGALYQALSPYVLFTFVQARSGGSVLLVSGGGV	4	0.10
NA (2021)_MLG113	N America	DT	PDSSLSSKRI	DR	TPIVGLSQIDA	PEDGTASTATTVQM	FGG	AL	YQ	AL	SPYV	LFTFV	QARSGG	SVL	LVSGGGV	DTPDSSLSSKRIDRTPIVGLSQIDAPEDGTASTATTVQMFGGALYQALSPYVLFTFVQARSGGSVLLVSGGGV	4	0.10
NA (2021)_MLG114	N America	DT	PDSSSSSRGI	DR	IPIVGLSQIDA	PEDGTASTATTVQM	FGG	AL	YQ	AL	SPYV	LFTFV	QARSGG	LVL	LVSGGGV	DTPDSSSSSRGIDRIPIVGLSQIDAPEDGTASTATTVQMFGGALYQALSPYVLFTFVQARSGGLVLLVSGGGV	3	0.07
NA (2021)_MLG115	N America	DT	PDSSSSSRGI	DR	TPIVGLSQIDA	PEDGTASTATTVQM	FGG	AL	YQ	AP	SPYV	LFTFV	QARSGG	SVL	LVSGDGV	DTPDSSSSSRGIDRTPIVGLSQIDAPEDGTASTATTVQMFGGALYQAPSPYVLFTFVQARSGGSVLLVSGDGV	3	0.07
NA (2021)_MLG116	N America	DT	PDSSSSSRGI	DR	IPIVGLSQIDA	PEDGTASTATTVQM	FGG	AL	YQ	AL	SPYV	LFTFV	HARSGG	LVL	FVSGGGV	DTPDSSSSSRGIDRIPIVGLSQIDAPEDGTASTATTVQMFGGALYQALSPYVLFTFVHARSGGLVLFVSGGGV	3	0.07
NA (2021)_MLG117	N America	DT	PDYSSSSRGI	DR	IPIVGLSQIDA	PEDGTASTATTVQM	FGG	AL	YQ	AL	SPYV	LFTFV	HARSGG	SVL	LVSGGGV	DTPDYSSSSRGIDRIPIVGLSQIDAPEDGTASTATTVQMFGGALYQALSPYVLFTFVHARSGGSVLLVSGGGV	3	0.07
NA (2021)_MLG118	N America	DT	PDSSLSSRGI	DR	TPIVGLSQIDA	PEDGTASTATTVQM	FGG	AL	YQ	AL	SPYV	LFTFV	QASSGG	SVL	LVSGGGV	DTPDSSLSSRGIDRTPIVGLSQIDAPEDGTASTATTVQMFGGALYQALSPYVLFTFVQASSGGSVLLVSGGGV	3	0.07
NA (2021)_MLG119	N America	DT	PDSSSSSRGI	DR	IPIVGLSQIDA	PEDGTASTATTVQM	FGG	AL	YQ	AL	SPYV	FFTFV	HARSGG	SVL	LVSGGGV	DTPDSSSSSRGIDRIPIVGLSQIDAPEDGTASTATTVQMFGGALYQALSPYVFFTFVHARSGGSVLLVSGGGV	3	0.07
NA (2021)_MLG120	N America	DT	PDSSSLSRGI	DR	TPIVGLSQIDA	PEDGTASTATTVQM	YGG	AF	YQ	AP	SPYV	LFTFV	QARSVG	SVS	LVSGGGV	DTPDSSSLSRGIDRTPIVGLSQIDAPEDGTASTATTVQMYGGAFYQAPSPYVLFTFVQARSVGSVSLVSGGGV	3	0.07
NA (2021)_MLG121	N America	DT	PDSSLSSRGI	DR	TPIVGLSQIDA	PEDGTASTATTVQM	FGG	AL	YQ	AL	SPYV	LFTFV	HARSGG	SVL	LVSGGGV	DTPDSSLSSRGIDRTPIVGLSQIDAPEDGTASTATTVQMFGGALYQALSPYVLFTFVHARSGGSVLLVSGGGV	3	0.07
NA (2021)_MLG122	N America	DT	PDSSSSSRGI	DR	IPIVGLSQIDA	PEDGTASTATTVQM	FGG	AL	YQ	AP	SPYV	LFTFV	HARSGG	LVL	LVSGGGV	DTPDSSSSSRGIDRIPIVGLSQIDAPEDGTASTATTVQMFGGALYQAPSPYVLFTFVHARSGGLVLLVSGGGV	3	0.07
NA (2021)_MLG123	N America	DT	PDSSSSSKRI	DR	TPIVGLSQIDA	PEDGTASTATTVQM	FGG	AL	YQ	AP	SPYV	LFTFV	QARSGG	SVL	LVSGDGV	DTPDSSSSSKRIDRTPIVGLSQIDAPEDGTASTATTVQMFGGALYQAPSPYVLFTFVQARSGGSVLLVSGDGV	3	0.07
NA (2021)_MLG124	N America	DT	PDSSSSSKRI	DR	TPIVGLSQIDA	PEDGTASTATTVQM	FGG	AL	YQ	AL	SPYV	LFTFL	QARSGG	SVL	LVSGGGV	DTPDSSSSSKRIDRTPIVGLSQIDAPEDGTASTATTVQMFGGALYQALSPYVLFTFLQARSGGSVLLVSGGGV	3	0.07
NA (2021)_MLG125	N America	DT	PDSSSSSKRI	DR	TPIVGLSQVDA	PEDGTASTATTVQM	FGG	AL	YQ	AL	SPYV	LFTFV	QARSGG	SVL	LVSGGGV	DTPDSSSSSKRIDRTPIVGLSQVDAPEDGTASTATTVQMFGGALYQALSPYVLFTFVQARSGGSVLLVSGGGV	3	0.07
NA (2021)_MLG126	N America	DT	PDSSSSSKRI	DR	TPIVGLSQIDV	PEDGTASTATTVQM	FGG	AL	YQ	AL	SPYV	LFTFV	QARSGG	SVL	LVSGGGV	DTPDSSSSSKRIDRTPIVGLSQIDVPEDGTASTATTVQMFGGALYQALSPYVLFTFVQARSGGSVLLVSGGGV	3	0.07
NA (2021)_MLG127	N America	DT	PDSSSSSKRI	DR	TPIVGLSQIDA	PEDGTASTATTVQM	FGG	AL	YQ	AL	SPYV	LFTFV	QARSGG	SVL	FVSGGGV	DTPDSSSSSKRIDRTPIVGLSQIDAPEDGTASTATTVQMFGGALYQALSPYVLFTFVQARSGGSVLFVSGGGV	3	0.07
NA (2021)_MLG128	N America	DT	PDSSSSSKRI	DR	TPIVGLSQIDA	PEDGTASTVTTVQM	FGG	AL	YQ	AL	SPYV	LFTFV	QARSGG	SVL	LVSGGGV	DTPDSSSSSKRIDRTPIVGLSQIDAPEDGTASTVTTVQMFGGALYQALSPYVLFTFVQARSGGSVLLVSGGGV	3	0.07
NA (2021)_MLG129	N America	DT	PDSSSSSKRI	DR	TPIVGLSQIDA	SEDGTASTATTVQM	FGG	AL	YQ	AL	SPYV	LFTFV	QARSGG	SVL	LVSGGGV	DTPDSSSSSKRIDRTPIVGLSQIDASEDGTASTATTVQMFGGALYQALSPYVLFTFVQARSGGSVLLVSGGGV	3	0.07
NA (2021)_MLG130	N America	DT	PDSSSSSRGI	DR	IPIVGLSQIDA	PEDGTASTATTVQM	FGG	AL	YQ	AF	SSYV	LFTFV	HARSGG	LVL	LVSGGGV	DTPDSSSSSRGIDRIPIVGLSQIDAPEDGTASTATTVQMFGGALYQAFSSYVLFTFVHARSGGLVLLVSGGGV	2	0.05
NA (2021)_MLG131	N America	DT	PDSSSSSRGI	DR	IPIVGLSQIDA	PEDGTASIATTVQM	FGG	AL	YQ	AL	SPYV	LFTFV	HARSGG	LVL	LVSGGGV	DTPDSSSSSRGIDRIPIVGLSQIDAPEDGTASIATTVQMFGGALYQALSPYVLFTFVHARSGGLVLLVSGGGV	2	0.05
NA (2021)_MLG132	N America	DT	PDSSLSSRGI	DR	TPIVGLSQIDA	LEDGTASTATTVQM	FGG	AL	YQ	AL	SPYV	LFTFV	QARSGG	SVL	LVSGGGV	DTPDSSLSSRGIDRTPIVGLSQIDALEDGTASTATTVQMFGGALYQALSPYVLFTFVQARSGGSVLLVSGGGV	2	0.05
NA (2021)_MLG133	N America	DT	PDPSLSSRGI	DR	TPIVGLSQIDA	PEDGTASTATTVQM	FGG	AL	YQ	AL	SPYV	LFTFV	QARSGG	SVL	LVSGGGV	DTPDPSLSSRGIDRTPIVGLSQIDAPEDGTASTATTVQMFGGALYQALSPYVLFTFVQARSGGSVLLVSGGGV	2	0.05
NA (2021)_MLG134	N America	DT	PDSSSLSRGI	DR	TPIVGLSQIDA	PEDGTASTATTVQM	YGG	AL	YQ	AP	SPYV	LFTFV	QARSVG	SVS	LVSGGGF	DTPDSSSLSRGIDRTPIVGLSQIDAPEDGTASTATTVQMYGGALYQAPSPYVLFTFVQARSVGSVSLVSGGGF	2	0.05
NA (2021)_MLG135	N America	DT	PDSSSSSRGI	DR	IPIVGLSQIDA	PEDGTASTATTVQM	FGG	AL	YQ	VL	SPYV	LFTFV	HARSGG	LVL	LVSGGGV	DTPDSSSSSRGIDRIPIVGLSQIDAPEDGTASTATTVQMFGGALYQVLSPYVLFTFVHARSGGLVLLVSGGGV	2	0.05
NA (2021)_MLG136	N America	DT	PDSSSSSRGI	DR	TPIVGLSQIDA	PEDGTASTATTVQM	FGG	AL	YQ	AL	SPYV	LFTFV	QSRSGG	SVL	LVSGGGV	DTPDSSSSSRGIDRTPIVGLSQIDAPEDGTASTATTVQMFGGALYQALSPYVLFTFVQSRSGGSVLLVSGGGV	2	0.05
NA (2021)_MLG137	N America	DT	PDSSSSSRGI	DR	IPIVGLSQIDA	PKDGTASTATTVQM	FGG	AL	YQ	AL	SPYV	LFTFV	HARSGG	LVL	LVSGGGV	DTPDSSSSSRGIDRIPIVGLSQIDAPKDGTASTATTVQMFGGALYQALSPYVLFTFVHARSGGLVLLVSGGGV	2	0.05
NA (2021)_MLG138	N America	DT	PDSSSSSRGI	DR	TPIVGLSQIDA	PEDGTASTATTVQM	FGG	AL	YQ	AL	SPYV	LFTFV	HDRSGG	SVL	LVSGGGV	DTPDSSSSSRGIDRTPIVGLSQIDAPEDGTASTATTVQMFGGALYQALSPYVLFTFVHDRSGGSVLLVSGGGV	2	0.05
NA (2021)_MLG139	N America	DT	PDSSSSSRGI	DR	IPIVGLSQIDA	SEDGTASTATTVQM	FGG	AL	YQ	AL	SPYV	LFTFV	HARSGG	LVL	LVSGGGV	DTPDSSSSSRGIDRIPIVGLSQIDASEDGTASTATTVQMFGGALYQALSPYVLFTFVHARSGGLVLLVSGGGV	2	0.05
NA (2021)_MLG140	N America	DT	PDSSSLSRGI	DR	TPIVGLSQIDA	PEDGTASTATTVQM	YGG	AL	YQ	AP	SPYV	LFTFV	QARSVG	SVS	LVSGGVV	DTPDSSSLSRGIDRTPIVGLSQIDAPEDGTASTATTVQMYGGALYQAPSPYVLFTFVQARSVGSVSLVSGGVV	2	0.05
NA (2021)_MLG141	N America	DT	PDSSSSSRGI	DR	TPIVGLSQIDA	PEDGTASTATTVQM	FGG	AF	YQ	AL	SPYV	LFTFV	QARSGG	SVL	LVSGGGV	DTPDSSSSSRGIDRTPIVGLSQIDAPEDGTASTATTVQMFGGAFYQALSPYVLFTFVQARSGGSVLLVSGGGV	2	0.05
NA (2021)_MLG142	N America	DT	PDSSSSCRGI	DR	IPIVGLSQIDA	PEDGTASTATTVQM	FGG	AL	YQ	VL	SPYV	LFTFV	HARSGG	LVL	LVSGGGV	DTPDSSSSCRGIDRIPIVGLSQIDAPEDGTASTATTVQMFGGALYQVLSPYVLFTFVHARSGGLVLLVSGGGV	2	0.05
NA (2021)_MLG143	N America	DT	PDSSSSCRGI	DR	TPIVGLSQIDA	PEDGTASTATTVQM	FGG	AL	YQ	AL	SPYV	LFTFV	HARSGG	SVL	LVSGGGV	DTPDSSSSCRGIDRTPIVGLSQIDAPEDGTASTATTVQMFGGALYQALSPYVLFTFVHARSGGSVLLVSGGGV	2	0.05
NA (2021)_MLG144	N America	DT	PDSSSSSRGI	DR	IPIVGLSQIDA	PEDGTASTATTVQM	FGG	AL	YQ	AL	SSYV	LFTFV	HARSGG	LVL	LVSGGGV	DTPDSSSSSRGIDRIPIVGLSQIDAPEDGTASTATTVQMFGGALYQALSSYVLFTFVHARSGGLVLLVSGGGV	2	0.05
NA (2021)_MLG145	N America	DT	PDSSSSSRGI	DR	TPIVGLSQIDA	PEDGTASTATTVQM	FGG	AL	YQ	AL	SPYV	LFTFV	QARSGG	SVL	LVSGGGF	DTPDSSSSSRGIDRTPIVGLSQIDAPEDGTASTATTVQMFGGALYQALSPYVLFTFVQARSGGSVLLVSGGGF	2	0.05
NA (2021)_MLG146	N America	DT	PDSSSSSKRI	DR	TPIVGLSQIDA	PEDGTASTATTVQM	FGG	AL	YQ	AL	SPYL	LFTFV	QARSGG	SVL	LVSGGGV	DTPDSSSSSKRIDRTPIVGLSQIDAPEDGTASTATTVQMFGGALYQALSPYLLFTFVQARSGGSVLLVSGGGV	2	0.05
NA (2021)_MLG147	N America	DT	PDSSSSSKRI	DR	IPIVGLSQIDA	PEDGTASTATTVQM	FGG	AL	YQ	AL	SPYV	LFTFV	QARSGG	SVL	LVSGGGV	DTPDSSSSSKRIDRIPIVGLSQIDAPEDGTASTATTVQMFGGALYQALSPYVLFTFVQARSGGSVLLVSGGGV	2	0.05
NA (2021)_MLG148	N America	DT	PDSSSSSKRI	DC	TPIVGLSQIDA	PEDGTASTATTVQM	FGG	AL	YQ	AL	SPYV	LFTFV	QARSGG	SVL	LVSGGGV	DTPDSSSSSKRIDCTPIVGLSQIDAPEDGTASTATTVQMFGGALYQALSPYVLFTFVQARSGGSVLLVSGGGV	2	0.05
NA (2021)_MLG149	N America	DT	PDSSSLSKRI	DR	TPIVGLSQIDA	PEDGTASTATTVQM	FGG	AL	YQ	AL	SPYV	LFTFV	QARSGG	SVL	LVSGGGV	DTPDSSSLSKRIDRTPIVGLSQIDAPEDGTASTATTVQMFGGALYQALSPYVLFTFVQARSGGSVLLVSGGGV	2	0.05
NA (2021)_MLG150	N America	DT	PDSSSSSKRI	DR	TPIVGLSQIDA	PEDGTASTATTVQM	FGS	AF	YQ	AL	SPYV	LFTFV	QARSGG	SVL	LVSGGGV	DTPDSSSSSKRIDRTPIVGLSQIDAPEDGTASTATTVQMFGSAFYQALSPYVLFTFVQARSGGSVLLVSGGGV	2	0.05
NA (2021)_MLG151	N America	DT	PDSSSSSKRI	DR	TPIVGLSQIDA	PEDGTASTATTVQM	FGG	AF	YQ	AL	SPYV	LFTFV	QARSGG	SVL	LVSGGGF	DTPDSSSSSKRIDRTPIVGLSQIDAPEDGTASTATTVQMFGGAFYQALSPYVLFTFVQARSGGSVLLVSGGGF	2	0.05
NA (2021)_MLG152	N America	DT	PDSSSSSKRI	DR	TPIVGLSQIDA	PEDGTASTATTVQM	FGG	AL	YQ	AL	SSYV	LFTFV	QARSGG	SVL	LVSGGGV	DTPDSSSSSKRIDRTPIVGLSQIDAPEDGTASTATTVQMFGGALYQALSSYVLFTFVQARSGGSVLLVSGGGV	2	0.05
NA (2021)_MLG153	N America	DT	PDSSSSSKRI	DR	TPIVGLSQIDA	PEDGTASTATTVQM	FGG	AL	YQ	AL	SPYV	LFTFV	QARSGG	SVL	LVNGGGV	DTPDSSSSSKRIDRTPIVGLSQIDAPEDGTASTATTVQMFGGALYQALSPYVLFTFVQARSGGSVLLVNGGGV	2	0.05
NA (2021)_MLG154	N America	DT	PDSSSSSKRI	DR	TPIVGLSQIDA	PEDGTASTATTVQM	FGG	AL	YQ	VL	SPYV	LFTFV	QARSGG	SVL	LVSGGGV	DTPDSSSSSKRIDRTPIVGLSQIDAPEDGTASTATTVQMFGGALYQVLSPYVLFTFVQARSGGSVLLVSGGGV	2	0.05
NA (2021)_MLG155	N America	DT	SDSSSSSKRI	DR	TPIVGLSQIDA	PEDGTASTATTVQM	FGG	AL	YQ	AL	SPYV	LFTFV	QARSGG	SVL	LVSGGGV	DTSDSSSSSKRIDRTPIVGLSQIDAPEDGTASTATTVQMFGGALYQALSPYVLFTFVQARSGGSVLLVSGGGV	2	0.05
NA (2021)_MLG156	N America	DT	PDSSSSSKRI	DR	TPIVGLSQIDA	PEDGTASTATTVQM	FGG	AL	YQ	AL	SPYV	LFTFV	QARSGG	SML	LVSGGGV	DTPDSSSSSKRIDRTPIVGLSQIDAPEDGTASTATTVQMFGGALYQALSPYVLFTFVQARSGGSMLLVSGGGV	2	0.05
NA (2021)_MLG157	N America	DT	PDSSSSSRGI	DR	IPIVGLSQIDA	PEDGTASTATTVQM	FGG	AL	YQ	AL	SPYV	LFTFV	HARSGG	LVL	LVSGDGV	DTPDSSSSSRGIDRIPIVGLSQIDAPEDGTASTATTVQMFGGALYQALSPYVLFTFVHARSGGLVLLVSGDGV	1	0.02
NA (2021)_MLG158	N America	DT	SDSSLSSRGI	DR	TPIVGLSQIDA	PEDGTASTATTVQM	FGG	AL	YQ	AL	SPYV	LFTFV	QARSGG	SLL	LVSGGGV	DTSDSSLSSRGIDRTPIVGLSQIDAPEDGTASTATTVQMFGGALYQALSPYVLFTFVQARSGGSLLLVSGGGV	1	0.02
NA (2021)_MLG159	N America	DT	PDSSSSSRGI	DR	TPIVGLSQIDA	PEDGTASTATTVQM	FGG	AL	YQ	AP	SPYV	LFTFV	QARSGG	LVL	LVSGGGV	DTPDSSSSSRGIDRTPIVGLSQIDAPEDGTASTATTVQMFGGALYQAPSPYVLFTFVQARSGGLVLLVSGGGV	1	0.02
NA (2021)_MLG160	N America	DT	PDSSSSSRGI	DR	IPIVGLSQIDA	PEDGTASTATTVQM	FGG	AF	YQ	AF	SPYV	LFTFV	HARSGG	LVL	LVSGGGV	DTPDSSSSSRGIDRIPIVGLSQIDAPEDGTASTATTVQMFGGAFYQAFSPYVLFTFVHARSGGLVLLVSGGGV	1	0.02
NA (2021)_MLG161	N America	DT	PDSLSSSRGI	DR	IPIVGLSQIDA	LEDGTASTATTVQM	FGG	AL	YQ	AL	SPYV	LFTFV	HARSGG	SVL	LVSGGGV	DTPDSLSSSRGIDRIPIVGLSQIDALEDGTASTATTVQMFGGALYQALSPYVLFTFVHARSGGSVLLVSGGGV	1	0.02
NA (2021)_MLG162	N America	DT	PDSSSSSRGI	DR	IPIVGLSQIDA	PEDGTASTATTVQM	FGG	AL	YQ	AL	SLYV	LFTFV	HARSGG	SVL	LVSGGGV	DTPDSSSSSRGIDRIPIVGLSQIDAPEDGTASTATTVQMFGGALYQALSLYVLFTFVHARSGGSVLLVSGGGV	1	0.02
NA (2021)_MLG163	N America	DT	PDSSSSSRGI	DR	IPIVGLSQIDV	PEDGTASTATTVQM	FGG	AL	YQ	AL	SPYV	LFTFV	HARSGG	LVL	LVSGGGV	DTPDSSSSSRGIDRIPIVGLSQIDVPEDGTASTATTVQMFGGALYQALSPYVLFTFVHARSGGLVLLVSGGGV	1	0.02
NA (2021)_MLG164	N America	DT	PDSSLSSRGI	DR	IPIVGLSQIDA	PEDGTASTATTVQM	FGG	AF	YQ	AL	SPYV	LFTFV	HARSGG	SVL	LVSGGGV	DTPDSSLSSRGIDRIPIVGLSQIDAPEDGTASTATTVQMFGGAFYQALSPYVLFTFVHARSGGSVLLVSGGGV	1	0.02
NA (2021)_MLG165	N America	DT	PDSSSSNRGI	DR	TPIVGLSQIDA	PEDGTASTATTVQM	FGG	AL	YQ	VP	SPYV	LFTFV	QARSGG	SVS	LVSGDGV	DTPDSSSSNRGIDRTPIVGLSQIDAPEDGTASTATTVQMFGGALYQVPSPYVLFTFVQARSGGSVSLVSGDGV	1	0.02
NA (2021)_MLG166	N America	DT	PNSSLSSRGI	DR	TPIVGLSQIDA	PEDGTASTATTVQM	FGG	AL	YQ	AL	SPYV	LFTFV	QARSGG	SVL	LVSGGGV	DTPNSSLSSRGIDRTPIVGLSQIDAPEDGTASTATTVQMFGGALYQALSPYVLFTFVQARSGGSVLLVSGGGV	1	0.02
NA (2021)_MLG167	N America	DT	PDSSSSSRGI	DR	IPIVGLSQIDA	PEDGTASTATTVQM	FGG	AL	YQ	AL	SPYV	FFTFV	HARSGG	LVL	LVSGGGV	DTPDSSSSSRGIDRIPIVGLSQIDAPEDGTASTATTVQMFGGALYQALSPYVFFTFVHARSGGLVLLVSGGGV	1	0.02

NA (2021)_MLG168	N America	DT	PDSSSSSRGI	DR	IPIVGLSQIDA	PEDGTASAATTVQM	FGG	AL	YQ	AL	SPYV	LFTFV	HARSGG	SVL	LVSGGGV	DTPDSSSSSRGIDRIPIVGLSQIDAPEDGTASAATTVQMFGGALYQALSPYVLFTFVHARSGGSVLLVSGGGV	1	0.02
NA (2021)_MLG169	N America	DT	PDSSSSSRGI	DR	TPIVGLSQIDA	PEDGTASTATTVQM	FGG	AF	YQ	AL	SPYV	LFTFV	HARSGG	SVL	LVSGGGV	DTPDSSSSSRGIDRTPIVGLSQIDAPEDGTASTATTVQMFGGAFYQALSPYVLFTFVHARSGGSVLLVSGGGV	1	0.02
NA (2021)_MLG170	N America	DT	PDSSSSRRGI	DR	TPIVGLSQIDA	PEDGTASTATTVQM	FGG	AL	YQ	AL	SPYV	LFTFV	HARSGG	SVL	LVSGGGV	DTPDSSSSRRGIDRTPIVGLSQIDAPEDGTASTATTVQMFGGALYQALSPYVLFTFVHARSGGSVLLVSGGGV	1	0.02
NA (2021)_MLG171	N America	DT	PDSSSSSRGI	DR	IPIVGLSQIDA	PEDGTASTATTVQM	FGG	AL	YQ	VL	SPYV	LFTFV	HSRSGG	LVL	LVSGGGV	DTPDSSSSSRGIDRIPIVGLSQIDAPEDGTASTATTVQMFGGALYQVLSPYVLFTFVHSRSGGLVLLVSGGGV	1	0.02
NA (2021)_MLG172	N America	DT	PDSSSSSRGI	DR	TPIVGLSQIDA	PEDGTASTATTVQM	FGG	AL	YQ	AL	SPYV	FFTFV	HARSGG	SVL	LVSGGGV	DTPDSSSSSRGIDRTPIVGLSQIDAPEDGTASTATTVQMFGGALYQALSPYVFFTFVHARSGGSVLLVSGGGV	1	0.02
NA (2021)_MLG173	N America	DT	PDSSLSSRGI	DR	TPIVGLSQIDA	PEDGTASTATTVQM	FGG	AL	YQ	AL	SPYV	LFTFV	QARSGG	SLL	LVSGGGV	DTPDSSLSSRGIDRTPIVGLSQIDAPEDGTASTATTVQMFGGALYQALSPYVLFTFVQARSGGSLLLVSGGGV	1	0.02
NA (2021)_MLG174	N America	DT	PDSSSSSRGI	DR	IPIVGLSQIDA	PEDGTAGTATTVQM	FGG	AL	YQ	AL	SPYV	LFTFV	HARSGG	LVL	LVSGGGV	DTPDSSSSSRGIDRIPIVGLSQIDAPEDGTAGTATTVQMFGGALYQALSPYVLFTFVHARSGGLVLLVSGGGV	1	0.02
NA (2021)_MLG175	N America	DT	PYSSSSSRGI	DR	IPIVGLSQIDA	PEDGTASTATTVQM	FGG	AL	YQ	AL	SPYV	LFTFV	HARSGG	SVL	LVSGGGV	DTPYSSSSSRGIDRIPIVGLSQIDAPEDGTASTATTVQMFGGALYQALSPYVLFTFVHARSGGSVLLVSGGGV	1	0.02
NA (2021)_MLG176	N America	DT	SDSSSLSRGI	DR	TPIVGLSQIDA	PEDGTASTATTVQM	YGG	AL	YQ	AP	SPYV	LFTFV	QARSVG	SVS	LVSGGGV	DTSDSSSLSRGIDRTPIVGLSQIDAPEDGTASTATTVQMYGGALYQAPSPYVLFTFVQARSVGSVSLVSGGGV	1	0.02
NA (2021)_MLG177	N America	DT	PDSSSSSRGI	DC	IPIVGLSQIDA	PEDGTASTATTVQM	FGG	AL	YQ	AL	SPYV	LFTFV	HARSGG	LVL	LVSGGGV	DTPDSSSSSRGIDCIPIVGLSQIDAPEDGTASTATTVQMFGGALYQALSPYVLFTFVHARSGGLVLLVSGGGV	1	0.02
NA (2021)_MLG178	N America	DT	PDSSSSSRGI	DR	IPIVGLSQIDA	PEDGTASTATTVQM	FGG	SL	YQ	AL	SPYV	LFTFV	HARSGG	LVL	LVSGGGV	DTPDSSSSSRGIDRIPIVGLSQIDAPEDGTASTATTVQMFGGSLYQALSPYVLFTFVHARSGGLVLLVSGGGV	1	0.02
NA (2021)_MLG179	N America	DT	PDSSLSSRGI	DR	TLIVGLSQIDA	PEDGTASTATTVQM	FGG	AL	YQ	AL	SPYV	LFTFV	QARSGG	SVL	LVSGGGV	DTPDSSLSSRGIDRTLIVGLSQIDAPEDGTASTATTVQMFGGALYQALSPYVLFTFVQARSGGSVLLVSGGGV	1	0.02
NA (2021)_MLG180	N America	DT	PDSSLSSRGI	DR	TPIVGLSQIDA	PEDGTASTATTVQV	FGG	AL	YQ	AL	SPYV	LFTFV	QARSGG	SVL	LVNGGGV	DTPDSSLSSRGIDRTPIVGLSQIDAPEDGTASTATTVQVFGGALYQALSPYVLFTFVQARSGGSVLLVNGGGV	1	0.02
NA (2021)_MLG181	N America	DT	PDSSLSSRGV	DR	TPIVGLSQIDA	PEDGTASTATTVQM	FGG	AL	YQ	AL	SPYV	LFTFV	QARSGG	SVL	LVSGGGV	DTPDSSLSSRGVDRTPIVGLSQIDAPEDGTASTATTVQMFGGALYQALSPYVLFTFVQARSGGSVLLVSGGGV	1	0.02
NA (2021)_MLG182	N America	DT	PDSSSSSRGI	DR	IPIVGLSQIDA	PEDGTASTATTVQM	FGG	AL	YQ	AL	SPYV	LFTFV	HARSGG	SVL	FVSGGGV	DTPDSSSSSRGIDRIPIVGLSQIDAPEDGTASTATTVQMFGGALYQALSPYVLFTFVHARSGGSVLFVSGGGV	1	0.02
NA (2021)_MLG183	N America	DT	PDSSSSSRGI	DR	IPIVGLSQIDA	PEDGTASTATTVQM	FGG	AL	YQ	AL	SPYV	LFTFV	HARSGG	LVL	LFSGGGV	DTPDSSSSSRGIDRIPIVGLSQIDAPEDGTASTATTVQMFGGALYQALSPYVLFTFVHARSGGLVLLFSGGGV	1	0.02
NA (2021)_MLG184	N America	DT	PDSSSSSRGI	DR	IPIVGLSQIDA	LEDGTASTATTVQM	FGG	AL	YQ	AL	SPYV	LFTFV	HARSGG	LVL	LVSGGGV	DTPDSSSSSRGIDRIPIVGLSQIDALEDGTASTATTVQMFGGALYQALSPYVLFTFVHARSGGLVLLVSGGGV	1	0.02
NA (2021)_MLG185	N America	DT	PDSSLSSRGI	DR	IPIVGLSQIDA	PEDGTASTATTVQM	FGG	AL	YQ	AL	SPYV	LFTFV	HARSGG	LVL	LVSGGGV	DTPDSSLSSRGIDRIPIVGLSQIDAPEDGTASTATTVQMFGGALYQALSPYVLFTFVHARSGGLVLLVSGGGV	1	0.02
NA (2021)_MLG186	N America	DT	PDSSSLSRGI	DR	TPIVGLSQIDA	PEDGTASTATTVQM	YGG	AL	YQ	AP	SPYV	LFTFV	QARSVG	SVS	LVSGDGV	DTPDSSSLSRGIDRTPIVGLSQIDAPEDGTASTATTVQMYGGALYQAPSPYVLFTFVQARSVGSVSLVSGDGV	1	0.02
NA (2021)_MLG187	N America	DT	PDSSSSSRGI	DR	IPIVGLSQIDA	PEDGTASTATTVQM	FGG	AL	YQ	AP	SPYV	LFTFV	QARSGG	LVL	LVSGDGV	DTPDSSSSSRGIDRIPIVGLSQIDAPEDGTASTATTVQMFGGALYQAPSPYVLFTFVQARSGGLVLLVSGDGV	1	0.02
NA (2021)_MLG188	N America	DT	PDSSSSSRGI	DR	IPIVGLSQIDA	PEDGTASTATTVQM	FGG	AL	YQ	AL	SPYV	LFTFV	HARSGA	LVL	LVSGGGV	DTPDSSSSSRGIDRIPIVGLSQIDAPEDGTASTATTVQMFGGALYQALSPYVLFTFVHARSGALVLLVSGGGV	1	0.02
NA (2021)_MLG189	N America	DT	PDSSSSSRGI	DR	IPIVGLSQIDA	LEDGTASTATTVQM	FGG	AF	YQ	AL	SPYV	LFTFV	HARSGG	SVL	LVSGGGV	DTPDSSSSSRGIDRIPIVGLSQIDALEDGTASTATTVQMFGGAFYQALSPYVLFTFVHARSGGSVLLVSGGGV	1	0.02
NA (2021)_MLG190	N America	DT	PDSSSSSRGI	DR	TPIVGLSQIDA	PEDGTASTATTVQM	FGG	AL	YQ	AL	SPYV	LFTFV	QARSGG	LVL	LVSGDGV	DTPDSSSSSRGIDRTPIVGLSQIDAPEDGTASTATTVQMFGGALYQALSPYVLFTFVQARSGGLVLLVSGDGV	1	0.02
NA (2021)_MLG191	N America	DT	PDSSSSSRGI	DR	TPIVGLSQIDA	PEDGTASTATTVQM	FGG	AL	YQ	AL	SPYV	LFTFV	HARSGG	SVL	LVSGGGF	DTPDSSSSSRGIDRTPIVGLSQIDAPEDGTASTATTVQMFGGALYQALSPYVLFTFVHARSGGSVLLVSGGGF	1	0.02
NA (2021)_MLG192	N America	DT	PDSSSSSRGI	DR	IPIVGLSQIDA	PEDGTASTAITVQM	FGG	AF	YQ	AL	SPYV	LFTFV	HARSGG	LVL	LVSGGGV	DTPDSSSSSRGIDRIPIVGLSQIDAPEDGTASTAITVQMFGGAFYQALSPYVLFTFVHARSGGLVLLVSGGGV	1	0.02
NA (2021)_MLG193	N America	DT	PDSSSSCRGI	DR	TPIVGLSQIDA	PEDGTASTATTVQM	FGG	AF	YQ	AL	SPYV	LFTFV	HARSGG	SVL	LVSGGGV	DTPDSSSSCRGIDRTPIVGLSQIDAPEDGTASTATTVQMFGGAFYQALSPYVLFTFVHARSGGSVLLVSGGGV	1	0.02
NA (2021)_MLG194	N America	DT	PDSSSSSRGI	DR	IPIVGLSQIDA	PEDGTASTATTVQM	FGG	AL	YQ	AL	SSYV	LFTFV	HARSGG	SVL	LVSGGGV	DTPDSSSSSRGIDRIPIVGLSQIDAPEDGTASTATTVQMFGGALYQALSSYVLFTFVHARSGGSVLLVSGGGV	1	0.02
NA (2021)_MLG195	N America	DT	PDSSSSSRGI	DR	IPIVGLSQIDA	PEDGTASTATTVQM	FGG	AL	YQ	AL	SPYV	LFTLV	HARSGG	SVL	LVSGGGV	DTPDSSSSSRGIDRIPIVGLSQIDAPEDGTASTATTVQMFGGALYQALSPYVLFTLVHARSGGSVLLVSGGGV	1	0.02
NA (2021)_MLG196	N America	DT	PDSSSSSRGI	DR	IPIVGLSQIDA	PEDGTASTAITVQM	FGG	AL	YQ	AL	SPYV	LFTFV	HARSGG	LVL	LVSGGGV	DTPDSSSSSRGIDRIPIVGLSQIDAPEDGTASTAITVQMFGGALYQALSPYVLFTFVHARSGGLVLLVSGGGV	1	0.02
NA (2021)_MLG197	N America	DT	PDSSSSSRGI	DR	IPIVGLSQIDA	PEDGTASTATTVQM	FGG	AL	YQ	AL	SPYV	LFTFV	HSRSGG	LVL	LVSGGGV	DTPDSSSSSRGIDRIPIVGLSQIDAPEDGTASTATTVQMFGGALYQALSPYVLFTFVHSRSGGLVLLVSGGGV	1	0.02
NA (2021)_MLG198	N America	DT	PDSSSLSRGI	DR	TPIVGLSQIDA	PEDGTASTATTVQM	YGG	AF	YQ	AP	SPYV	LFTFV	QARSVG	SLS	LVSGGGV	DTPDSSSLSRGIDRTPIVGLSQIDAPEDGTASTATTVQMYGGAFYQAPSPYVLFTFVQARSVGSLSLVSGGGV	1	0.02
NA (2021)_MLG199	N America	DT	PDSSSSSRGI	DR	IPIVGLSQIDA	PEDGTASTATTVQM	FGG	AL	YQ	VL	SPYV	LFTFV	HARSGG	SVL	LVSGGGV	DTPDSSSSSRGIDRIPIVGLSQIDAPEDGTASTATTVQMFGGALYQVLSPYVLFTFVHARSGGSVLLVSGGGV	1	0.02
NA (2021)_MLG200	N America	DT	PDSSSSSRGI	DR	IPIVGLSQIDA	PEDGTASTATTVQM	FGG	AL	YQ	AL	SPYV	LFSFV	HARSGG	LVL	LVSGGGV	DTPDSSSSSRGIDRIPIVGLSQIDAPEDGTASTATTVQMFGGALYQALSPYVLFSFVHARSGGLVLLVSGGGV	1	0.02
NA (2021)_MLG201	N America	DT	PDSPSSSRGI	DR	IPIVGLSQIDA	PEDGTASTATTVQM	FGG	AL	YQ	AL	SPYV	LFTFV	HARSGG	SVL	LVSGGGV	DTPDSPSSSRGIDRIPIVGLSQIDAPEDGTASTATTVQMFGGALYQALSPYVLFTFVHARSGGSVLLVSGGGV	1	0.02
NA (2021)_MLG202	N America	DT	PDSSSSSRGI	DR	IPIVGFSQIDA	PEDGTASTATTVQM	FGG	AF	YQ	AL	SPYV	LFTFV	HARSGG	LVL	LVSGGGV	DTPDSSSSSRGIDRIPIVGFSQIDAPEDGTASTATTVQMFGGAFYQALSPYVLFTFVHARSGGLVLLVSGGGV	1	0.02
NA (2021)_MLG203	N America	DT	PDSSSSSRGI	DR	TPIVGLSQIDA	PEDGTASTATTVQM	FGG	AL	YQ	AL	SPYV	FFTFV	QSRSGG	SVL	LVSGGGV	DTPDSSSSSRGIDRTPIVGLSQIDAPEDGTASTATTVQMFGGALYQALSPYVFFTFVQSRSGGSVLLVSGGGV	1	0.02
NA (2021)_MLG204	N America	DT	PDSSSSSRGI	DR	IPIVGLSQIDA	PEDGTASTATTVQM	FGG	AL	YQ	AL	SPYV	LFTFV	HARSGG	LVL	LVSGGVV	DTPDSSSSSRGIDRIPIVGLSQIDAPEDGTASTATTVQMFGGALYQALSPYVLFTFVHARSGGLVLLVSGGVV	1	0.02
NA (2021)_MLG205	N America	DT	PDSSSSSRGI	DR	IPIVGLSQIDA	PEDGTASKATTVQM	FGG	AL	YQ	AL	SPYV	LFTFV	HARSGG	LVL	LVSGGGV	DTPDSSSSSRGIDRIPIVGLSQIDAPEDGTASKATTVQMFGGALYQALSPYVLFTFVHARSGGLVLLVSGGGV	1	0.02
NA (2021)_MLG206	N America	DT	PDSLSSSRGI	DR	TPIVGLSQIDA	PEDGTASTATTVQM	FGG	AL	YQ	AL	SPYV	LFTFV	HARSGG	SVL	LVSGGGV	DTPDSLSSSRGIDRTPIVGLSQIDAPEDGTASTATTVQMFGGALYQALSPYVLFTFVHARSGGSVLLVSGGGV	1	0.02
NA (2021)_MLG207	N America	DT	PDSSSSSRGI	DR	TPIVGLSQIDA	PEDGTASTATTVQM	FGG	AL	YQ	AL	SPYV	LFTFV	HARSGG	SLL	LVNGGGV	DTPDSSSSSRGIDRTPIVGLSQIDAPEDGTASTATTVQMFGGALYQALSPYVLFTFVHARSGGSLLLVNGGGV	1	0.02
NA (2021)_MLG208	N America	DT	PDSLSSSRGI	DR	IPIVGLSQIDA	LEDGTASTATTVQM	FGG	AL	YQ	AL	SPYV	LFTFV	HARSGG	LVL	LVSGGGV	DTPDSLSSSRGIDRIPIVGLSQIDALEDGTASTATTVQMFGGALYQALSPYVLFTFVHARSGGLVLLVSGGGV	1	0.02
NA (2021)_MLG209	N America	DT	PDSSLSSRGI	DR	TPIVGLSQIDA	PEDGTASTATTVQM	FGG	AL	YQ	AL	SPYV	LFIFV	QARSGG	SVL	LVSGGGV	DTPDSSLSSRGIDRTPIVGLSQIDAPEDGTASTATTVQMFGGALYQALSPYVLFIFVQARSGGSVLLVSGGGV	1	0.02
NA (2021)_MLG210	N America	DT	PDSSSSSKLI	DR	TPIVGLSQIDA	PEDGTASTATTVQM	FGG	AL	YQ	AL	SPYV	LFTFV	QARSGG	SVL	LVSGGGV	DTPDSSSSSKLIDRTPIVGLSQIDAPEDGTASTATTVQMFGGALYQALSPYVLFTFVQARSGGSVLLVSGGGV	1	0.02
NA (2021)_MLG211	N America	DT	PDSSSSSKPI	DR	TPIVGLSQIDA	PEDGTASTATTVQM	FGG	AL	YQ	AL	SPYV	LFTFV	QARSGG	SVL	LVSGGGV	DTPDSSSSSKPIDRTPIVGLSQIDAPEDGTASTATTVQMFGGALYQALSPYVLFTFVQARSGGSVLLVSGGGV	1	0.02
NA (2021)_MLG212	N America	DT	PDSSSSSKRI	DR	TPIVGLSQIDA	PEDGTASTATTVQM	FGG	AL	YQ	AP	SPYV	LFTFV	QARSGG	SVL	LVSGGGV	DTPDSSSSSKRIDRTPIVGLSQIDAPEDGTASTATTVQMFGGALYQAPSPYVLFTFVQARSGGSVLLVSGGGV	1	0.02
NA (2021)_MLG213	N America	DM	PDSSSSSKRI	DR	TPIVGLSQIDA	PEDGTASTATTVQM	FGG	AL	YQ	AL	SPYV	LFTFV	QARSGG	SVL	LVSGGGV	DMPDSSSSSKRIDRTPIVGLSQIDAPEDGTASTATTVQMFGGALYQALSPYVLFTFVQARSGGSVLLVSGGGV	1	0.02
NA (2021)_MLG214	N America	DT	PDSSSSSKRI	DR	TPIVGLSQIDA	PEDGTASTATTVQM	FGS	AL	YQ	AL	SPYV	LFTFV	QARSGG	SVL	LVSGGGV	DTPDSSSSSKRIDRTPIVGLSQIDAPEDGTASTATTVQMFGSALYQALSPYVLFTFVQARSGGSVLLVSGGGV	1	0.02
NA (2021)_MLG215	N America	DT	PDSSSSSKRI	DR	TPIVGLSQIDA	PEDGTASTATTVQM	FGG	AL	YQ	AL	SPYV	LFTFV	QARFGG	SVL	LVSGGGV	DTPDSSSSSKRIDRTPIVGLSQIDAPEDGTASTATTVQMFGGALYQALSPYVLFTFVQARFGGSVLLVSGGGV	1	0.02
NA (2021)_MLG216	N America	DT	PDSSSSSKRI	DR	TPIVGLSQIDA	PEDGTASTATTVQM	FGG	AL	YQ	AL	SPYV	LFTFV	QARSGG	SVL	LVSGGVV	DTPDSSSSSKRIDRTPIVGLSQIDAPEDGTASTATTVQMFGGALYQALSPYVLFTFVQARSGGSVLLVSGGVV	1	0.02
NA (2021)_MLG217	N America	DT	PDSSSSSKRI	DR	TPIVGLSQIDA	PEDGTASTATTVQM	FGG	AL	YQ	AL	SHYV	LFTFV	QARSGG	SVL	LVSGGGV	DTPDSSSSSKRIDRTPIVGLSQIDAPEDGTASTATTVQMFGGALYQALSHYVLFTFVQARSGGSVLLVSGGGV	1	0.02
NA (2021)_MLG218	N America	DT	PDSSSSSKRI	DR	TPIVGLSQIDA	PEDGTASKATTVQM	FGG	AL	YQ	AL	SPYV	LFTFV	QARSGG	SVL	LVSGGGV	DTPDSSSSSKRIDRTPIVGLSQIDAPEDGTASKATTVQMFGGALYQALSPYVLFTFVQARSGGSVLLVSGGGV	1	0.02
NA (2021)_MLG219	N America	DT	LDSSSSSKRI	DR	TPIVGLSQIDA	PEDGTASTATTVQM	FGG	AL	YQ	AL	SPYV	LFTFV	QARSGG	SVL	LVSGGGV	DTLDSSSSSKRIDRTPIVGLSQIDAPEDGTASTATTVQMFGGALYQALSPYVLFTFVQARSGGSVLLVSGGGV	1	0.02
NA (2021)_MLG220	N America	DT	PDSSSSSKRI	DR	TPIVGLSQIDA	PEDGTASIATTVQM	FGG	AL	YQ	AL	SPYV	LFTFV	QARSGG	SVL	LVSGGGV	DTPDSSSSSKRIDRTPIVGLSQIDAPEDGTASIATTVQMFGGALYQALSPYVLFTFVQARSGGSVLLVSGGGV	1	0.02
NA (2021)_MLG221	N America	DT	PDSSSSSKRI	DR	TPIVGLFQIDA	PEDGTASTATTVQM	FGG	AL	YQ	AL	SPYV	LFTFV	QARSGG	SVL	LVSGGGV	DTPDSSSSSKRIDRTPIVGLFQIDAPEDGTASTATTVQMFGGALYQALSPYVLFTFVQARSGGSVLLVSGGGV	1	0.02
NA (2021)_MLG222	N America	DT	PDSSSSSKRI	DR	TPIVCLSQIDA	PEDGTASTATTVQM	FGG	AL	YQ	AL	SPYV	LFTFV	QARSGS	SVL	LVSGGGV	DTPDSSSSSKRIDRTPIVCLSQIDAPEDGTASTATTVQMFGGALYQALSPYVLFTFVQARSGSSVLLVSGGGV	1	0.02
NA (2021)_MLG223	N America	DT	PDSSSSSKRI	DR	TLIVGLSQIDA	PEDGTASTATTVQM	FGG	AL	YQ	AL	SPYV	LFTFV	QARSGG	SVL	LVSGGGV	DTPDSSSSSKRIDRTLIVGLSQIDAPEDGTASTATTVQMFGGALYQALSPYVLFTFVQARSGGSVLLVSGGGV	1	0.02
NA (2021)_MLG224	N America	DT	PDSSSSSKRI	DR	TPIVGLSQIDA	SEDGTASTATTVQM	FGG	AF	YQ	AL	SPYV	LFTFV	QARSGG	SVL	LVSGGGV	DTPDSSSSSKRIDRTPIVGLSQIDASEDGTASTATTVQMFGGAFYQALSPYVLFTFVQARSGGSVLLVSGGGV	1	0.02
NA (2021)_MLG225	N America	DT	PDSSSSSKRI	DR	TPIVGLSQIDA	PEDGTASTATTVQM	FGG	AL	YQ	AL	SPYV	LFTFV	QTRSGG	SVL	LVSGGGV	DTPDSSSSSKRIDRTPIVGLSQIDAPEDGTASTATTVQMFGGALYQALSPYVLFTFVQTRSGGSVLLVSGGGV	1	0.02
NA (2021)_MLG226	N America	DT	TDSSSSSKRI	DR	TPIVGLSQIDA	PEDGTASTATTVQM	FGG	AL	YQ	AL	SPYV	LFTFV	QARSGG	SVL	LVSGGGV	DTTDSSSSSKRIDRTPIVGLSQIDAPEDGTASTATTVQMFGGALYQALSPYVLFTFVQARSGGSVLLVSGGGV	1	0.02
NA (2021)_MLG227	N America	DT	PDSSSSSKRI	DR	TPIVGLSQIDA	PEDGTASTATTVQM	FGG	AL	YQ	AL	SLYV	LFTFV	QARSGG	SVL	LVSGGGV	DTPDSSSSSKRIDRTPIVGLSQIDAPEDGTASTATTVQMFGGALYQALSLYVLFTFVQARSGGSVLLVSGGGV	1	0.02
NA (2021)_MLG228	N America	DT	PDSSSSSKRI	NR	TPIVGLSQIDA	PEDGTASTATTVQM	FGG	AL	YQ	AL	SPYV	LFTFV	QARSGG	SVL	LVSGGGV	DTPDSSSSSKRINRTPIVGLSQIDAPEDGTASTATTVQMFGGALYQALSPYVLFTFVQARSGGSVLLVSGGGV	1	0.02
NA (2021)_MLG229	N America	DT	PDSSSSSKRI	DR	TPIVGLSQIDA	PEDGTASTATTVQM	FGG	AL	YQ	AL	SPYV	LFTFV	QARSGG	SLL	FVSGGGV	DTPDSSSSSKRIDRTPIVGLSQIDAPEDGTASTATTVQMFGGALYQALSPYVLFTFVQARSGGSLLFVSGGGV	1	0.02
NA (2021)_MLG230	N America	DT	PDSSSSSKRI	DR	TPIVGLSQIDA	PEDGTASTATTVQM	FGG	AL	YQ	AL	SPYV	LFTFV	QASSGG	SVL	LVSGGGV	DTPDSSSSSKRIDRTPIVGLSQIDAPEDGTASTATTVQMFGGALYQALSPYVLFTFVQASSGGSVLLVSGGGV	1	0.02
NA (2021)_MLG231	N America	DT	PDSSSSSKRI	DR	TPIVGLSQIDA	PEDGTASTATTVQM	FGG	AL	YQ	AL	SPYV	LFTFV	QARSGG	SVL	LVSGDGV	DTPDSSSSSKRIDRTPIVGLSQIDAPEDGTASTATTVQMFGGALYQALSPYVLFTFVQARSGGSVLLVSGDGV	1	0.02

NA (2021)_MLG232	N America	DT	PDSSSSSKRI	DR	TPIVGLSQIDA	PEDGIASTATTVQM	FSG	AL	YQ	AL	SPYV	LFTFV	QARSGG	SVL	LVSGGGV	DTPDSSSSSKRIDRTPIVGLSQIDAPEDGIASTATTVQMFSGALYQALSPYVLFTFVQARSGGSVLLVSGGGV	1	0.02
NA (2021)_MLG233	N America	DT	PDSSSSSKRI	DR	TPIVGLSQIDA	PEDGTASTATTVQM	FGG	AL	YQ	AL	SPYV	LFTFV	QVRSGG	SVL	LVSGGGV	DTPDSSSSSKRIDRTPIVGLSQIDAPEDGTASTATTVQMFGGALYQALSPYVLFTFVQVRSGGSVLLVSGGGV	1	0.02
NA (2021)_MLG234	N America	DT	PDSSSSSKRI	DR	TPIVGLSQIDA	PENGTASTATTVQM	FGG	AL	YQ	AL	SPYV	LFTFV	QARSGG	SVL	LVSGGGV	DTPDSSSSSKRIDRTPIVGLSQIDAPENGTASTATTVQMFGGALYQALSPYVLFTFVQARSGGSVLLVSGGGV	1	0.02
NA (2021)_MLG235	N America	DT	PDSSSSSKRI	DR	TPIVGLSQIDA	PADGTASTATTVQM	FGG	AL	YQ	AL	SPYV	LFTFV	QARSGG	SVL	LVSGGGV	DTPDSSSSSKRIDRTPIVGLSQIDAPADGTASTATTVQMFGGALYQALSPYVLFTFVQARSGGSVLLVSGGGV	1	0.02
NA (2021)_MLG236	N America	DT	PDSSSSSKRI	DR	TPIVCLSQIDA	PEDGTASTATTVQM	FGG	AL	YQ	AL	SPYV	LFTFV	QARSGG	SVL	LVSGGGV	DTPDSSSSSKRIDRTPIVCLSQIDAPEDGTASTATTVQMFGGALYQALSPYVLFTFVQARSGGSVLLVSGGGV	1	0.02
NA (2021)_MLG237	N America	DT	PDSSSSSMGI	DR	TPIVGLSQIDA	PEDGTASTATTVQM	FGG	AL	YQ	AL	SPYV	LFTFL	QARSGG	SVL	LVSGGGV	DTPDSSSSSMGIDRTPIVGLSQIDAPEDGTASTATTVQMFGGALYQALSPYVLFTFLQARSGGSVLLVSGGGV	1	0.02
NA (2021)_MLG238	N America	DT	PDSSSSSSGI	DR	IPIVGLSQIDA	PEDGTASTATTVQM	FGG	AL	YQ	AL	SPYV	LFTFV	HARSGG	SVL	LVSGGGV	DTPDSSSSSSGIDRIPIVGLSQIDAPEDGTASTATTVQMFGGALYQALSPYVLFTFVHARSGGSVLLVSGGGV	1	0.02
OC (2020)_MLG1	Oceania	DT	PDSSSSSRGI	DR	TPIVGLSQIPA	SPEDGTASTATTVQM	FGG	AL	YQ	AL	SPYV	LFTFV	QARSGG	SVL	LVSGGGV	DTPDSSSSSRGIDRTPIVGLSQIPASPEDGTASTATTVQMFGGALYQALSPYVLFTFVQARSGGSVLLVSGGGV	233	14.20
OC (2020)_MLG2	Oceania	DT	PDSSSSSRGI	DR	IPIVGLSQIPA	SPEDGTASTATTVQM	FGG	AL	YQ	AL	SPYV	LFTFV	HARSGG	SVL	LVSGGGV	DTPDSSSSSRGIDRIPIVGLSQIPASPEDGTASTATTVQMFGGALYQALSPYVLFTFVHARSGGSVLLVSGGGV	173	10.50
OC (2020)_MLG3	Oceania	DT	PDSSSSSKRI	DR	TPIVGLSQIPA	SPEDGTASTATTVQM	FGG	AL	YQ	AL	SPYV	LFTFV	QARSGG	SVL	LVSGGGV	DTPDSSSSSKRIDRTPIVGLSQIPASPEDGTASTATTVQMFGGALYQALSPYVLFTFVQARSGGSVLLVSGGGV	160	9.70
OC (2020)_MLG4	Oceania	DT	PDSSSSSRGI	DR	TPIVGLSQIPA	SPEDGTASTATTVQM	FGG	AL	YQ	AP	SLCV	LFTFV	QARSGG	SVS	LVSGDGV	DTPDSSSSSRGIDRTPIVGLSQIPASPEDGTASTATTVQMFGGALYQAPSLCVLFTFVQARSGGSVSLVSGDGV	92	5.60
OC (2020)_MLG5	Oceania	DT	LDSSSLSRGI	DR	TPIVGLSQIPA	SPEDGTASTATTVQM	YGG	AL	YQ	AP	SPYV	LFTFV	QARSVG	SVS	LVSGDGV	DTLDSSSLSRGIDRTPIVGLSQIPASPEDGTASTATTVQMYGGALYQAPSPYVLFTFVQARSVGSVSLVSGDGV	80	4.90
OC (2020)_MLG6	Oceania	DT	PDSSSSSRGI	DR	TPIVGLSQIPA	SPEDGTASTATTVQM	FGG	AL	YQ	AL	SPYV	LFTFV	HARSGG	SVL	LVSGGGV	DTPDSSSSSRGIDRTPIVGLSQIPASPEDGTASTATTVQMFGGALYQALSPYVLFTFVHARSGGSVLLVSGGGV	65	3.90
OC (2020)_MLG7	Oceania	DT	PDSSSSSRGI	DR	IPIVGLSQIPA	SPEDGTASTVTTVQM	FGG	AL	YQ	AL	SPYV	LFTFV	HARSGG	SVL	LVSGGGV	DTPDSSSSSRGIDRIPIVGLSQIPASPEDGTASTVTTVQMFGGALYQALSPYVLFTFVHARSGGSVLLVSGGGV	54	3.30
OC (2020)_MLG8	Oceania	DT	PDSSSSSRGI	DR	TPIVGLSQIPA	SPEDGTASTATTVQM	FGG	AF	YQ	AP	SPYV	LFTFV	QARSGV	SVL	LVSGDGV	DTPDSSSSSRGIDRTPIVGLSQIPASPEDGTASTATTVQMFGGAFYQAPSPYVLFTFVQARSGVSVLLVSGDGV	50	3.00
OC (2020)_MLG9	Oceania	DT	PDSSSSSKRI	DR	TPFVGLSQIPA	SPEDGTASTATTVQM	FGG	AL	YQ	AL	SPYV	LFTFV	QARSGG	SVL	LVNGGGV	DTPDSSSSSKRIDRTPFVGLSQIPASPEDGTASTATTVQMFGGALYQALSPYVLFTFVQARSGGSVLLVNGGGV	47	2.90
OC (2020)_MLG10	Oceania	DT	PDSSSSSRGI	DR	TSIIGLSQIPA	SPEDGTASTATTVQM	FGG	AF	YQ	AP	SPYV	LFTFV	QARSGG	LVL	LVSGDGV	DTPDSSSSSRGIDRTSIIGLSQIPASPEDGTASTATTVQMFGGAFYQAPSPYVLFTFVQARSGGLVLLVSGDGV	46	2.80
OC (2020)_MLG11	Oceania	DT	PDSSSSSRGI	ER	TPIVGLSQIPA	SLEDGTASTATTVQM	FGG	AL	YQ	AP	SPYV	LLTFL	QARSGG	SLS	LVSGDGV	DTPDSSSSSRGIERTPIVGLSQIPASLEDGTASTATTVQMFGGALYQAPSPYVLLTFLQARSGGSLSLVSGDGV	37	2.20
OC (2020)_MLG12	Oceania	DT	LDSSSSSRGI	DR	TPIVGLSQIPA	SPEDGTASKATTVQM	FGG	AF	YQ	VP	SPYV	LFTFV	QARSGG	SVL	LVSGDGV	DTLDSSSSSRGIDRTPIVGLSQIPASPEDGTASKATTVQMFGGAFYQVPSPYVLFTFVQARSGGSVLLVSGDGV	34	2.10
OC (2020)_MLG13	Oceania	DT	PDSSSSSRGI	DR	TPIIGLSQIPA	SPEDGTASTATTVQM	FGG	AF	YQ	AP	SPYV	LFTFV	QARSGG	SVL	LVSGDGV	DTPDSSSSSRGIDRTPIIGLSQIPASPEDGTASTATTVQMFGGAFYQAPSPYVLFTFVQARSGGSVLLVSGDGV	28	1.70
OC (2020)_MLG14	Oceania	DT	PDSSSSSRGI	DR	IPIVGLSQIPA	SPEDGTASTATTVQM	FGG	VL	YQ	AL	SPYV	LFTFV	HARSGG	SVL	LVSGGGV	DTPDSSSSSRGIDRIPIVGLSQIPASPEDGTASTATTVQMFGGVLYQALSPYVLFTFVHARSGGSVLLVSGGGV	27	1.60
OC (2020)_MLG15	Oceania	DT	PDSSSSSRGI	DR	TPIVGLSQVSA	SPEDGTASTATTVQM	FGG	AF	YQ	AP	SPYV	LFTFV	QARSGV	SVL	LVSGDGV	DTPDSSSSSRGIDRTPIVGLSQVSASPEDGTASTATTVQMFGGAFYQAPSPYVLFTFVQARSGVSVLLVSGDGV	27	1.60
OC (2020)_MLG16	Oceania	DT	PDSSSSSRGI	DR	TPIVGLSQIPA	SPEDGTASTATTVQM	FGG	AL	YQ	AP	SPYV	LFTFV	QARSGG	SVL	LVSGDGV	DTPDSSSSSRGIDRTPIVGLSQIPASPEDGTASTATTVQMFGGALYQAPSPYVLFTFVQARSGGSVLLVSGDGV	23	1.40
OC (2020)_MLG17	Oceania	DT	PDSSSSSRGI	DR	IPIVGLSQIPA	SPEDGTASTVTTVQM	FGG	AL	YQ	AL	SPYV	LFTFV	HARSGG	SVL	LVSRGGV	DTPDSSSSSRGIDRIPIVGLSQIPASPEDGTASTVTTVQMFGGALYQALSPYVLFTFVHARSGGSVLLVSRGGV	23	1.40
OC (2020)_MLG18	Oceania	DT	PDSSSSSRGI	DR	IPIVGLSQIPA	SPEDGTASTATTVQM	FGG	AL	YQ	AL	SPYV	LFTFV	HARSGG	LVL	LVSGGGV	DTPDSSSSSRGIDRIPIVGLSQIPASPEDGTASTATTVQMFGGALYQALSPYVLFTFVHARSGGLVLLVSGGGV	21	1.30
OC (2020)_MLG19	Oceania	DT	PDSSSSSRGI	DR	TPIVGLSQIPA	SPEDGTASTAITVQM	FGG	AL	YQ	AL	SPYV	LFTFV	QARSGG	SVL	LVSGGGV	DTPDSSSSSRGIDRTPIVGLSQIPASPEDGTASTAITVQMFGGALYQALSPYVLFTFVQARSGGSVLLVSGGGV	18	1.10
OC (2020)_MLG20	Oceania	DT	PDSSSSSKRI	DR	TPIVGLSQIPA	SPEDGIASTATTVQM	FSG	AL	YQ	AL	SPYV	LFTFV	QARSGG	SVL	LVSGGGV	DTPDSSSSSKRIDRTPIVGLSQIPASPEDGIASTATTVQMFSGALYQALSPYVLFTFVQARSGGSVLLVSGGGV	15	0.90
OC (2020)_MLG21	Oceania	DT	PDSSSSSRGI	DR	IPIVGLSQIPA	SPEDVTASTATTVQM	FGG	AL	YQ	AL	SPYV	LFTFV	HARSGG	SVL	LVSGGVV	DTPDSSSSSRGIDRIPIVGLSQIPASPEDVTASTATTVQMFGGALYQALSPYVLFTFVHARSGGSVLLVSGGVV	14	0.90
OC (2020)_MLG22	Oceania	GT	PDSSSSSRGI	DR	TPIVGLSQIPA	SPEDGTASTATTVQM	FGG	AL	YQ	AL	SPYV	LFTFV	QARSGG	SVL	LVSGGGV	GTPDSSSSSRGIDRTPIVGLSQIPASPEDGTASTATTVQMFGGALYQALSPYVLFTFVQARSGGSVLLVSGGGV	13	0.80
OC (2020)_MLG23	Oceania	DT	PDSSSSSRGI	DC	TPIIGLSQIPA	SPEDGTASTATTVQI	FGG	AF	YQ	AP	SPYV	LFTFV	QARSGG	SVL	LVSGDGV	DTPDSSSSSRGIDCTPIIGLSQIPASPEDGTASTATTVQIFGGAFYQAPSPYVLFTFVQARSGGSVLLVSGDGV	13	0.80
OC (2020)_MLG24	Oceania	DM	PDSSSSSKRI	DR	TPIVGLSQIPA	SPEDGTASTATTVQM	FGG	AL	YQ	AL	SPYV	LFTFV	QARSGG	SVL	LVSGGGV	DMPDSSSSSKRIDRTPIVGLSQIPASPEDGTASTATTVQMFGGALYQALSPYVLFTFVQARSGGSVLLVSGGGV	13	0.80
OC (2020)_MLG25	Oceania	DT	PDSSSSSRGI	DR	TPIVDLSQIPT	SPEDGTASTATTVQM	FGG	AL	YQ	AP	SPYV	LFTFV	QARSGG	SVL	LVSGDGV	DTPDSSSSSRGIDRTPIVDLSQIPTSPEDGTASTATTVQMFGGALYQAPSPYVLFTFVQARSGGSVLLVSGDGV	11	0.70
OC (2020)_MLG26	Oceania	DT	PDSSSSSRGI	DR	TPIVGLSQIPA	SPEDGTASTATTVHM	FGG	AF	YQ	AL	SPYV	LFTFV	HARSGG	SVL	LVSGGGV	DTPDSSSSSRGIDRTPIVGLSQIPASPEDGTASTATTVHMFGGAFYQALSPYVLFTFVHARSGGSVLLVSGGGV	11	0.70
OC (2020)_MLG27	Oceania	DT	PDSSSSSRGI	DR	TPIVGLSQISA	SPEDGTASTATTVQM	FGG	AF	YQ	AP	SPYV	LFTFV	QARSGV	SVL	LVSGDGV	DTPDSSSSSRGIDRTPIVGLSQISASPEDGTASTATTVQMFGGAFYQAPSPYVLFTFVQARSGVSVLLVSGDGV	10	0.60
OC (2020)_MLG28	Oceania	DT	PDSSSSSRGI	DR	TPIVGLSQIPA	SPEDGTASTATTVQM	FGG	AL	YQ	AP	SPYV	LFTFV	QARSGG	SVS	LVSGDGV	DTPDSSSSSRGIDRTPIVGLSQIPASPEDGTASTATTVQMFGGALYQAPSPYVLFTFVQARSGGSVSLVSGDGV	10	0.60
OC (2020)_MLG29	Oceania	DT	PDSSLSSRGI	DR	TPIVGLSQIPA	SPEDGTASTATTVQM	FGG	AL	YQ	AL	SPYV	LFTFV	QARSGG	SVL	LVSGGGV	DTPDSSLSSRGIDRTPIVGLSQIPASPEDGTASTATTVQMFGGALYQALSPYVLFTFVQARSGGSVLLVSGGGV	9	0.50
OC (2020)_MLG30	Oceania	DT	PGSSSSSRGI	DR	TPIVGLSQIPA	LPEDGTASTATTVQM	FGG	AF	YQ	AP	SPYV	LFTFV	QARSGV	SVL	LVSGDGV	DTPGSSSSSRGIDRTPIVGLSQIPALPEDGTASTATTVQMFGGAFYQAPSPYVLFTFVQARSGVSVLLVSGDGV	9	0.50
OC (2020)_MLG31	Oceania	DT	PDSSSSSKRI	DR	TPFVGLSQIPA	SPEDGTASTATTVQM	FGG	AL	YQ	AL	SPYV	LFTFV	QARSGG	SVL	LVSGGGV	DTPDSSSSSKRIDRTPFVGLSQIPASPEDGTASTATTVQMFGGALYQALSPYVLFTFVQARSGGSVLLVSGGGV	9	0.50
OC (2020)_MLG32	Oceania	DT	PDSSSSSRGI	DR	TSIIGLSQIPA	SPEDGTASTATTVQM	FGG	AF	YQ	AP	SPYV	LFTFV	QARSGG	SVL	LVSGDGV	DTPDSSSSSRGIDRTSIIGLSQIPASPEDGTASTATTVQMFGGAFYQAPSPYVLFTFVQARSGGSVLLVSGDGV	8	0.50
OC (2020)_MLG33	Oceania	DT	PDSSSSNRGI	DR	TPIVGLSQIPA	SPEDGTASTATTVQM	FGG	AL	YQ	AP	SPYV	LFTFV	QARSGG	SVS	LVSGDGV	DTPDSSSSNRGIDRTPIVGLSQIPASPEDGTASTATTVQMFGGALYQAPSPYVLFTFVQARSGGSVSLVSGDGV	8	0.50
OC (2020)_MLG34	Oceania	DT	PDSSSSSRGI	DR	TPIVGLSQIPA	SPEDGTASTATTVQM	FGG	AL	YQ	AP	SPYV	LFTFV	QARSGV	SVL	LVSGDGV	DTPDSSSSSRGIDRTPIVGLSQIPASPEDGTASTATTVQMFGGALYQAPSPYVLFTFVQARSGVSVLLVSGDGV	8	0.50
OC (2020)_MLG35	Oceania	DT	PDSSSSSRGI	DR	TPIVGLSQIPA	SPEDVTASTATTVQM	FGG	AL	YQ	AL	SPYV	LFTFV	QARSGG	SVL	LVSGGVV	DTPDSSSSSRGIDRTPIVGLSQIPASPEDVTASTATTVQMFGGALYQALSPYVLFTFVQARSGGSVLLVSGGVV	7	0.40
OC (2020)_MLG36	Oceania	DT	PDSSSSSRGI	DR	TPIVGLSQIPA	SPEDGTASTATTVQM	FGG	AF	YQ	AP	SPYV	LFTFV	QARSGG	SVL	LVSGDGV	DTPDSSSSSRGIDRTPIVGLSQIPASPEDGTASTATTVQMFGGAFYQAPSPYVLFTFVQARSGGSVLLVSGDGV	7	0.40
OC (2020)_MLG37	Oceania	DT	PDSSSSSKRI	DR	TPIVGLSQIPA	SPEEGTASTATTVQM	FGG	AL	YQ	AL	SPYV	LFTFV	QARSGG	SVL	LVSGGGV	DTPDSSSSSKRIDRTPIVGLSQIPASPEEGTASTATTVQMFGGALYQALSPYVLFTFVQARSGGSVLLVSGGGV	7	0.40
OC (2020)_MLG38	Oceania	DT	PDSSSSSKRI	DR	TPIVGLSQIPA	SPEDVTASTATTVQM	FGG	AL	YQ	AL	SPYV	LFTFV	QARSGG	SVL	LVSGGGV	DTPDSSSSSKRIDRTPIVGLSQIPASPEDVTASTATTVQMFGGALYQALSPYVLFTFVQARSGGSVLLVSGGGV	5	0.30
OC (2020)_MLG39	Oceania	DT	PDSSSLSRGI	DR	TPIVGLSQIPA	SPEDGTASTATTVQM	YGG	AL	YQ	AP	SPYV	LFTFV	QARSVG	SVS	LVSGDGV	DTPDSSSLSRGIDRTPIVGLSQIPASPEDGTASTATTVQMYGGALYQAPSPYVLFTFVQARSVGSVSLVSGDGV	5	0.30
OC (2020)_MLG40	Oceania	DT	PDSSSSSRGI	DR	TPIVGLSQIPA	SPEDGTASTATTVHM	FGG	AL	YQ	AL	SPYV	LFTFV	HARSGG	SVL	LVSGGGV	DTPDSSSSSRGIDRTPIVGLSQIPASPEDGTASTATTVHMFGGALYQALSPYVLFTFVHARSGGSVLLVSGGGV	5	0.30
OC (2020)_MLG41	Oceania	DT	PGSSSSSRGI	DR	TPIVGLSQIPA	LPEDVTASTATTVQM	FGG	AF	YQ	AP	SPYL	LFTFV	QARSGV	SVL	LVSGDVV	DTPGSSSSSRGIDRTPIVGLSQIPALPEDVTASTATTVQMFGGAFYQAPSPYLLFTFVQARSGVSVLLVSGDVV	5	0.30
OC (2020)_MLG42	Oceania	DT	PDSSSSSKRI	DR	TPIVGLSQIPA	SPEDGTASTATTVQM	FGG	AL	YQ	AL	SPYV	LFTFV	QARSGG	SVL	FVSGGGV	DTPDSSSSSKRIDRTPIVGLSQIPASPEDGTASTATTVQMFGGALYQALSPYVLFTFVQARSGGSVLFVSGGGV	4	0.20
OC (2020)_MLG43	Oceania	DT	PDSSSSSRGI	DR	TPIVGLSQIPA	SPEDVTASTATTVQM	FGG	AF	YQ	AP	SPYV	LFTFV	QARSGV	SVL	LVSGDVV	DTPDSSSSSRGIDRTPIVGLSQIPASPEDVTASTATTVQMFGGAFYQAPSPYVLFTFVQARSGVSVLLVSGDVV	4	0.20
OC (2020)_MLG44	Oceania	DT	PGSSSSSRGI	DR	TPIVGLSQIPA	LPEDGTASTATTVQM	FGG	AF	YQ	AP	SPYL	LFTFV	QARSGV	SVL	LVSGDGV	DTPGSSSSSRGIDRTPIVGLSQIPALPEDGTASTATTVQMFGGAFYQAPSPYLLFTFVQARSGVSVLLVSGDGV	4	0.20
OC (2020)_MLG45	Oceania	DM	PDSSSSSKRI	DR	TPIVGLSQIPA	SPEDVTASTATTVQM	FGG	AL	YQ	AL	SPYV	LFTFV	QARSGG	SVL	LVSGGVV	DMPDSSSSSKRIDRTPIVGLSQIPASPEDVTASTATTVQMFGGALYQALSPYVLFTFVQARSGGSVLLVSGGVV	4	0.20
OC (2020)_MLG46	Oceania	DT	PDSSSSSRGI	DR	TPIVGLSQIPA	SPEDVTASTATTVQM	FGG	AL	YQ	AL	SPYV	LFTFV	QARSGG	SVL	LVSGGGV	DTPDSSSSSRGIDRTPIVGLSQIPASPEDVTASTATTVQMFGGALYQALSPYVLFTFVQARSGGSVLLVSGGGV	4	0.20
OC (2020)_MLG47	Oceania	DT	PDSSSSSRGI	DR	IPIVGLSQIPA	SPEDVTASTATTVQM	FGG	AL	YQ	AL	SPYV	LFTFV	HARSGG	SVL	LVSGGGV	DTPDSSSSSRGIDRIPIVGLSQIPASPEDVTASTATTVQMFGGALYQALSPYVLFTFVHARSGGSVLLVSGGGV	4	0.20
OC (2020)_MLG48	Oceania	DT	PDSSLSSRGI	DR	TPIVGLSQIPA	SPEDGTASTATTVQM	FGG	AL	YQ	AL	SPYV	LFTFV	HARSGG	SVL	LVSGGGV	DTPDSSLSSRGIDRTPIVGLSQIPASPEDGTASTATTVQMFGGALYQALSPYVLFTFVHARSGGSVLLVSGGGV	4	0.20
OC (2020)_MLG49	Oceania	DT	PDSSSSSKRI	DR	TPIVGLSQIPA	SPEDGIASTATTVQM	FSG	AF	YQ	AL	SPYV	LFTFV	QARSGG	SVL	LVSGGGV	DTPDSSSSSKRIDRTPIVGLSQIPASPEDGIASTATTVQMFSGAFYQALSPYVLFTFVQARSGGSVLLVSGGGV	3	0.20
OC (2020)_MLG50	Oceania	DT	PDSSSSSRGI	DR	TSIIGLSQIPA	SPEDGTASTATTVQM	FGG	AL	YQ	AP	SPYV	LFTFV	QARSGG	LVL	LVSGDGV	DTPDSSSSSRGIDRTSIIGLSQIPASPEDGTASTATTVQMFGGALYQAPSPYVLFTFVQARSGGLVLLVSGDGV	3	0.20
OC (2020)_MLG51	Oceania	DT	PDSSSSSRGI	DR	TPIVGLSQIPA	SPEDVTASTATTVQM	FGG	AL	YQ	AP	SLCV	LFTFV	QARSGG	SVS	LVSGDVV	DTPDSSSSSRGIDRTPIVGLSQIPASPEDVTASTATTVQMFGGALYQAPSLCVLFTFVQARSGGSVSLVSGDVV	3	0.20
OC (2020)_MLG52	Oceania	DT	PGSSSSSRGI	DR	TPIVGLSQIPA	SPEDGTASTATTVQM	FGG	AF	YQ	AP	SPYV	LFTFV	QARSGV	SVL	LVSGDGV	DTPGSSSSSRGIDRTPIVGLSQIPASPEDGTASTATTVQMFGGAFYQAPSPYVLFTFVQARSGVSVLLVSGDGV	3	0.20
OC (2020)_MLG53	Oceania	DT	PDSSSSSRGI	DR	TPIIGLSQIPA	SPEDGTASTATTVQM	FGG	AL	YQ	AP	SPYV	LFTFV	QARSGG	SVL	LVSGDGV	DTPDSSSSSRGIDRTPIIGLSQIPASPEDGTASTATTVQMFGGALYQAPSPYVLFTFVQARSGGSVLLVSGDGV	3	0.20
OC (2020)_MLG54	Oceania	DT	PDSSSSSKRI	DR	TPIVGLSQIPA	SPEDGTASTATTVQM	FGG	AF	YQ	AL	SPYV	LFTFV	QARSGG	SVL	LVSGGGV	DTPDSSSSSKRIDRTPIVGLSQIPASPEDGTASTATTVQMFGGAFYQALSPYVLFTFVQARSGGSVLLVSGGGV	3	0.20
OC (2020)_MLG55	Oceania	DT	PDSSSSSRGI	DR	TSIIGLSQIPA	SPEDVTASTATTVQM	FGG	AF	YQ	AP	SPYV	LFTFV	QARSGG	LVL	LVSGDVV	DTPDSSSSSRGIDRTSIIGLSQIPASPEDVTASTATTVQMFGGAFYQAPSPYVLFTFVQARSGGLVLLVSGDVV	3	0.20
OC (2020)_MLG56	Oceania	DT	PDSSSSSRGI	DR	TPIVGLSQIPA	SPEDGTASTATTVQM	FGG	AF	YQ	AL	SPYV	LFTFV	QARSGG	SVL	LVSGGGV	DTPDSSSSSRGIDRTPIVGLSQIPASPEDGTASTATTVQMFGGAFYQALSPYVLFTFVQARSGGSVLLVSGGGV	3	0.20
OC (2020)_MLG57	Oceania	DT	PDSSSSSKRI	DR	IPIVGLSQIPA	SPEDGTASTATTVQM	FGG	AL	YQ	AL	SPYV	LFTFV	HARSGG	SVL	LVSGGGV	DTPDSSSSSKRIDRIPIVGLSQIPASPEDGTASTATTVQMFGGALYQALSPYVLFTFVHARSGGSVLLVSGGGV	3	0.20

OC (2020)_MLG58	Oceania	DT	PDSSSSSRGI	DR	TPIVGLSQIPA	SPEDGTASTATTVQM	FGG	AL	YQ	AL	SPYV	LFTFV	QARSGG	SVL	LVSGGVV	DTPDSSSSSRGIDRTPIVGLSQIPASPEDGTASTATTVQMFGGALYQALSPYVLFTFVQARSGGSVLLVSGGVV	2	0.10
OC (2020)_MLG59	Oceania	DT	PDSSSSSRGI	DR	TPIVGLSQIPA	SPEDVTASTAITVQM	FGG	AL	YQ	AL	SPYV	LFTFV	QARSGG	SVL	LVSGGVV	DTPDSSSSSRGIDRTPIVGLSQIPASPEDVTASTAITVQMFGGALYQALSPYVLFTFVQARSGGSVLLVSGGVV	2	0.10
OC (2020)_MLG60	Oceania	DT	PDSSSSSRGI	DR	TPIVGLSQIPA	SPEDGTASTATTVQM	FGG	AL	YQ	AP	SPYV	LFTFV	QARSGG	SVL	LVSGGGV	DTPDSSSSSRGIDRTPIVGLSQIPASPEDGTASTATTVQMFGGALYQAPSPYVLFTFVQARSGGSVLLVSGGGV	2	0.10
OC (2020)_MLG61	Oceania	DT	LDSSSSSRGI	DR	TPIVGLSQIPA	SPEDVTASKATTVQM	FGG	AF	YQ	VP	SPYV	LFTFV	QARSGG	SVL	LVSGDVV	DTLDSSSSSRGIDRTPIVGLSQIPASPEDVTASKATTVQMFGGAFYQVPSPYVLFTFVQARSGGSVLLVSGDVV	2	0.10
OC (2020)_MLG62	Oceania	DT	PDSSSSSRGI	DR	TSIIGLSQIPA	SPEDVTASTATTVQM	FGG	AF	YQ	AP	SPYV	LFTFV	QARSGG	LVL	LVSGDGV	DTPDSSSSSRGIDRTSIIGLSQIPASPEDVTASTATTVQMFGGAFYQAPSPYVLFTFVQARSGGLVLLVSGDGV	2	0.10
OC (2020)_MLG63	Oceania	DT	PDSSSSSKRI	DR	TPIVGLSQIPA	SPEDGTASTATTVQM	FGG	AL	YQ	AP	SPYV	LFTFV	QARSGG	SVL	LVSGGGV	DTPDSSSSSKRIDRTPIVGLSQIPASPEDGTASTATTVQMFGGALYQAPSPYVLFTFVQARSGGSVLLVSGGGV	2	0.10
OC (2020)_MLG64	Oceania	DT	PDSSSSSRGI	DR	TPIVGLSQIPA	SPEDGTASTATTVQM	FGG	AF	YQ	AP	SPYV	LFTFV	QARSGG	SVL	LVSGGGV	DTPDSSSSSRGIDRTPIVGLSQIPASPEDGTASTATTVQMFGGAFYQAPSPYVLFTFVQARSGGSVLLVSGGGV	2	0.10
OC (2020)_MLG65	Oceania	DT	PDSSSSSRGI	DR	TPIVDLSQIPT	SPEDVTASTATTVQM	FGG	AL	YQ	AP	SPYV	LFTFV	QARSGG	SVL	LVSGDVV	DTPDSSSSSRGIDRTPIVDLSQIPTSPEDVTASTATTVQMFGGALYQAPSPYVLFTFVQARSGGSVLLVSGDVV	2	0.10
OC (2020)_MLG66	Oceania	DT	LDSSSSSRGI	DR	TPIVGLSQIPA	SPEDGTARKATTVQM	FGG	AF	YQ	VP	SPYV	LFTFV	QARSGG	SVL	LVSGDGV	DTLDSSSSSRGIDRTPIVGLSQIPASPEDGTARKATTVQMFGGAFYQVPSPYVLFTFVQARSGGSVLLVSGDGV	2	0.10
OC (2020)_MLG67	Oceania	DT	LDSSSSSRGI	DR	TPIVGLSQIPA	SPEDGTARKATTVQM	FGG	AL	YQ	VP	SPYV	LFTFV	QARSGG	SVL	LVSGDGV	DTLDSSSSSRGIDRTPIVGLSQIPASPEDGTARKATTVQMFGGALYQVPSPYVLFTFVQARSGGSVLLVSGDGV	2	0.10
OC (2020)_MLG68	Oceania	DT	PDSSSSSRGI	DR	TPIVGLSQIPA	LPEDGTASTATTVQM	FGG	AL	YQ	AL	SPYV	LFTFV	QARSGG	SVL	LVSGGGV	DTPDSSSSSRGIDRTPIVGLSQIPALPEDGTASTATTVQMFGGALYQALSPYVLFTFVQARSGGSVLLVSGGGV	2	0.10
OC (2020)_MLG69	Oceania	DT	PDSSSSSKRI	DR	TPIVGLSQIPA	SPEDGTASTATTVQM	FGG	AL	YQ	AL	SPYV	LFTFV	QARSGG	SLL	LVSGGGV	DTPDSSSSSKRIDRTPIVGLSQIPASPEDGTASTATTVQMFGGALYQALSPYVLFTFVQARSGGSLLLVSGGGV	2	0.10
OC (2020)_MLG70	Oceania	DT	PDSSSSSRGI	DR	TPIVGLSQVSA	SPEDGTASTATTVQM	FGG	AL	YQ	AP	SPYV	LFTFV	QARSGV	SVL	LVSGDGV	DTPDSSSSSRGIDRTPIVGLSQVSASPEDGTASTATTVQMFGGALYQAPSPYVLFTFVQARSGVSVLLVSGDGV	2	0.10
OC (2020)_MLG71	Oceania	DT	PDSSSSSRGI	DR	IPIVGLSQIPA	LPEDGTASTVTTVQM	FGG	AL	YQ	AL	SPYV	LFTFV	HARSGG	SVL	LVSGGGV	DTPDSSSSSRGIDRIPIVGLSQIPALPEDGTASTVTTVQMFGGALYQALSPYVLFTFVHARSGGSVLLVSGGGV	2	0.10
OC (2020)_MLG72	Oceania	DT	PDSSSSSKRI	DR	TPIVGLSQIPA	SPEDGTASTATTVQM	FGS	AL	YQ	AL	SPYV	LFTFV	QARSGG	SVL	LVSGGGV	DTPDSSSSSKRIDRTPIVGLSQIPASPEDGTASTATTVQMFGSALYQALSPYVLFTFVQARSGGSVLLVSGGGV	2	0.10
OC (2020)_MLG73	Oceania	DT	PDSSSSSRGI	DR	TPIVGLSQIPA	SPEDVTASTATTVQM	FGG	AL	YQ	AP	SPYV	LFTFV	QARSGG	SVL	LVSGDVV	DTPDSSSSSRGIDRTPIVGLSQIPASPEDVTASTATTVQMFGGALYQAPSPYVLFTFVQARSGGSVLLVSGDVV	2	0.10
OC (2020)_MLG74	Oceania	DT	PDSSSSSKRI	DR	TPIVGLSQIPA	SPEDVTASTATTVQM	FGG	AL	YQ	AL	SPYV	LFTFV	QARSGG	SVL	LVSGGVV	DTPDSSSSSKRIDRTPIVGLSQIPASPEDVTASTATTVQMFGGALYQALSPYVLFTFVQARSGGSVLLVSGGVV	2	0.10
OC (2020)_MLG75	Oceania	DT	PDSSSSSRGI	DR	IPIVGLSQIPA	SPEDGTASTATTVQM	FGG	AF	YQ	AL	SPYV	LFTFV	HARSGG	SVL	LVSGGGV	DTPDSSSSSRGIDRIPIVGLSQIPASPEDGTASTATTVQMFGGAFYQALSPYVLFTFVHARSGGSVLLVSGGGV	2	0.10
OC (2020)_MLG76	Oceania	DT	PDSSSSSRGI	DR	TSIIGLSQIPA	SPEDGTASTATTVQL	FGG	AL	YQ	AP	SPYV	LFTFV	QARSGG	LVL	LVSGDGV	DTPDSSSSSRGIDRTSIIGLSQIPASPEDGTASTATTVQLFGGALYQAPSPYVLFTFVQARSGGLVLLVSGDGV	2	0.10
OC (2020)_MLG77	Oceania	DT	PDSSSSSRGI	DC	TPIIGLSQIPA	SPEDGTASTATTVQM	FGG	AF	YQ	AP	SPYV	LFTFV	QARSGG	SVL	LVSGDGV	DTPDSSSSSRGIDCTPIIGLSQIPASPEDGTASTATTVQMFGGAFYQAPSPYVLFTFVQARSGGSVLLVSGDGV	2	0.10
OC (2020)_MLG78	Oceania	DT	PDSSSSSRGI	DR	TPIVGLSQIPA	SPEDGTASTATTVQM	FGG	AL	YQ	AL	SPYV	LFTFV	QARSGG	SVL	LVSGDGV	DTPDSSSSSRGIDRTPIVGLSQIPASPEDGTASTATTVQMFGGALYQALSPYVLFTFVQARSGGSVLLVSGDGV	2	0.10
OC (2020)_MLG79	Oceania	DT	PDSSSSSRGI	DR	TSIIGLSQIPA	SPEDGTASTATTVQM	FGG	AF	YQ	AP	SPYV	LFTFV	QARSGG	LVL	LVSGDVV	DTPDSSSSSRGIDRTSIIGLSQIPASPEDGTASTATTVQMFGGAFYQAPSPYVLFTFVQARSGGLVLLVSGDVV	2	0.10
OC (2020)_MLG80	Oceania	DT	PDSSSSSRGI	DR	IPIVGLSQIPA	SPEDGTASTATTVQM	FGG	AL	YQ	AL	SPYV	LFTFV	HVRSGG	SVL	LVSGGGV	DTPDSSSSSRGIDRIPIVGLSQIPASPEDGTASTATTVQMFGGALYQALSPYVLFTFVHVRSGGSVLLVSGGGV	2	0.10
OC (2020)_MLG81	Oceania	DT	PDSSSSSRGI	DR	IPIVGLSQIPA	SPEDGTASTATTVQM	FGG	AL	YQ	AP	SPYV	LFTFV	HARSGG	SVL	LVSGGGV	DTPDSSSSSRGIDRIPIVGLSQIPASPEDGTASTATTVQMFGGALYQAPSPYVLFTFVHARSGGSVLLVSGGGV	2	0.10
OC (2020)_MLG82	Oceania	DT	LDSSSSSRGI	DR	TPIVGLSQIPA	SPEDGTARKATTVQM	FGG	AF	YH	VP	SPYV	LFTFV	QARSGG	SVL	LVSGDGV	DTLDSSSSSRGIDRTPIVGLSQIPASPEDGTARKATTVQMFGGAFYHVPSPYVLFTFVQARSGGSVLLVSGDGV	2	0.10
OC (2020)_MLG83	Oceania	DT	PDSSSSSRGI	DR	IPIVGLSQIPA	SPEDGTASTVTTVQM	FGG	AF	YQ	AL	SPYV	LFTFV	HARSGG	SVL	LVSRGGV	DTPDSSSSSRGIDRIPIVGLSQIPASPEDGTASTVTTVQMFGGAFYQALSPYVLFTFVHARSGGSVLLVSRGGV	2	0.10
OC (2020)_MLG84	Oceania	DT	PDSSSSSRGI	DR	IPIVGLSQIPA	SPEDGTASTVTTVQM	FGG	AL	YQ	AP	SPYV	LFTFV	HARSGG	SVL	LVSGGGV	DTPDSSSSSRGIDRIPIVGLSQIPASPEDGTASTVTTVQMFGGALYQAPSPYVLFTFVHARSGGSVLLVSGGGV	2	0.10
OC (2020)_MLG85	Oceania	DT	PDSSSSSRGI	DR	TPIVGLSQIPA	SPEDATASTATTVQM	FGG	AL	YQ	AL	SPYV	LFTFV	QARSGG	SVL	LVSGGGV	DTPDSSSSSRGIDRTPIVGLSQIPASPEDATASTATTVQMFGGALYQALSPYVLFTFVQARSGGSVLLVSGGGV	1	0.10
OC (2020)_MLG86	Oceania	DT	PDSSSSSRGI	DR	TPIVGLSQIPA	SPEDGTASTATTVQM	YGG	AL	YQ	AL	SPYV	LFTFV	QARSVG	SVL	LVSGDGV	DTPDSSSSSRGIDRTPIVGLSQIPASPEDGTASTATTVQMYGGALYQALSPYVLFTFVQARSVGSVLLVSGDGV	1	0.10
OC (2020)_MLG87	Oceania	DT	LDSSSSSRGI	DR	TPIVGLSQIPA	SPEDGTASTATTVQM	YGG	AL	YQ	AP	SPYV	LFTFV	QARSGG	SVS	LVSGDGV	DTLDSSSSSRGIDRTPIVGLSQIPASPEDGTASTATTVQMYGGALYQAPSPYVLFTFVQARSGGSVSLVSGDGV	1	0.10
OC (2020)_MLG88	Oceania	DT	PDSSSSSRGI	DR	TPIVGLSQIPA	SPEDGTASTATTVQM	YGG	AL	YQ	AL	SPYV	LFTFV	QARSGG	SVL	LVSGGGV	DTPDSSSSSRGIDRTPIVGLSQIPASPEDGTASTATTVQMYGGALYQALSPYVLFTFVQARSGGSVLLVSGGGV	1	0.10
OC (2020)_MLG89	Oceania	DT	PDSSSSSKRI	DR	TPFVGLSQIPA	SSEDGTASTATTVQM	FGG	AL	YQ	AL	SPYV	LFTFV	QARSGG	SVL	LVNGGGV	DTPDSSSSSKRIDRTPFVGLSQIPASSEDGTASTATTVQMFGGALYQALSPYVLFTFVQARSGGSVLLVNGGGV	1	0.10
OC (2020)_MLG90	Oceania	YT	PDSSSSSKRI	DR	TPIVGLSQIPA	SPEDGTASTATTVQM	FGG	AL	YQ	AL	SPYV	LFTFV	QARSGG	SVL	LVSGGGV	YTPDSSSSSKRIDRTPIVGLSQIPASPEDGTASTATTVQMFGGALYQALSPYVLFTFVQARSGGSVLLVSGGGV	1	0.10
OC (2020)_MLG91	Oceania	DT	PDSSSSSRGI	DR	IPIVGLSQIPA	SPEDGTASTATTVQM	FGG	AF	YQ	AL	SPYV	LFTFV	HARSGV	SVL	LVSGDGV	DTPDSSSSSRGIDRIPIVGLSQIPASPEDGTASTATTVQMFGGAFYQALSPYVLFTFVHARSGVSVLLVSGDGV	1	0.10
OC (2020)_MLG92	Oceania	DT	PDSSSSSKRI	DR	TPIVGLSQIPA	SPEDGTASTATTVHM	FGG	AL	YQ	AL	SPYV	LFTFV	QARSGG	SVL	LVSGGGV	DTPDSSSSSKRIDRTPIVGLSQIPASPEDGTASTATTVHMFGGALYQALSPYVLFTFVQARSGGSVLLVSGGGV	1	0.10
OC (2020)_MLG93	Oceania	DT	PDSSSSSRGI	DR	TPIVGLSQIPA	SPEDGTASTATTVQM	FSG	AL	YQ	AL	SPYV	LFTFV	QARSGG	SVL	LVSGGGV	DTPDSSSSSRGIDRTPIVGLSQIPASPEDGTASTATTVQMFSGALYQALSPYVLFTFVQARSGGSVLLVSGGGV	1	0.10
OC (2020)_MLG94	Oceania	DT	PDSSSSSRGI	DR	IPIVGLSQIPA	SPEDVTASTATTVQM	FGG	VL	YQ	AL	SPYV	LFTFV	HARSGG	SVL	LVSGGGV	DTPDSSSSSRGIDRIPIVGLSQIPASPEDVTASTATTVQMFGGVLYQALSPYVLFTFVHARSGGSVLLVSGGGV	1	0.10
OC (2020)_MLG95	Oceania	DT	PDSSSSSRGI	DR	TPIVGLSQIPA	SPEDGTASTATTVQM	FGG	AL	YQ	AP	SPCV	LFTFV	QARSGG	SVS	LVSGDGV	DTPDSSSSSRGIDRTPIVGLSQIPASPEDGTASTATTVQMFGGALYQAPSPCVLFTFVQARSGGSVSLVSGDGV	1	0.10
OC (2020)_MLG96	Oceania	DT	PDSSSSSRGI	DR	TPIVGLSQIPA	SPEDGTASTATTVQM	FGG	AL	YQ	AL	LPYV	LFTFV	HARSGG	SVL	LVSGGGV	DTPDSSSSSRGIDRTPIVGLSQIPASPEDGTASTATTVQMFGGALYQALLPYVLFTFVHARSGGSVLLVSGGGV	1	0.10
OC (2020)_MLG97	Oceania	DT	PDSSSSSRGI	DR	IPIVGLSQIPA	SPEDGTASTATTVQL	FGG	AL	YQ	AL	SPYV	LFTFV	HARSGG	SVL	LVSGGGV	DTPDSSSSSRGIDRIPIVGLSQIPASPEDGTASTATTVQLFGGALYQALSPYVLFTFVHARSGGSVLLVSGGGV	1	0.10
OC (2020)_MLG98	Oceania	DT	PDSSSSSRGI	DR	TPIVGLSQIPA	SPEDGTASTAITVQM	FGG	AC	YQ	AL	SPYV	LFTFV	QARSGG	SVL	LVSGGGV	DTPDSSSSSRGIDRTPIVGLSQIPASPEDGTASTAITVQMFGGACYQALSPYVLFTFVQARSGGSVLLVSGGGV	1	0.10
OC (2020)_MLG99	Oceania	DT	PDSSSSSKRI	DR	TPIVGLSQIPA	SPEEVTASTATTVQM	FGG	AL	YQ	AL	SPYV	LFTFV	QARSGG	SVL	LVSGGVV	DTPDSSSSSKRIDRTPIVGLSQIPASPEEVTASTATTVQMFGGALYQALSPYVLFTFVQARSGGSVLLVSGGVV	1	0.10
OC (2020)_MLG100	Oceania	DT	PDSSSSSRGI	DR	TPIVGLSQVSA	SSEDGTASTATTVQM	FGG	AL	YQ	VP	SPYV	LFTFV	QVRFGV	SVL	LVSGDGV	DTPDSSSSSRGIDRTPIVGLSQVSASSEDGTASTATTVQMFGGALYQVPSPYVLFTFVQVRFGVSVLLVSGDGV	1	0.10
OC (2020)_MLG101	Oceania	DT	PDSSSSSRGI	DR	TPIVGLSQIPA	SPEDGTASTATTVQM	FGG	AL	YQ	AL	SPYV	LFTFV	QARSGG	SVL	LASGGGV	DTPDSSSSSRGIDRTPIVGLSQIPASPEDGTASTATTVQMFGGALYQALSPYVLFTFVQARSGGSVLLASGGGV	1	0.10
OC (2020)_MLG102	Oceania	DT	PDSSSSSRGI	DR	TPIVGLFQIPA	SPEDGTASTATTVQM	FGG	AL	YQ	AL	SPYV	LFTFV	QARSGG	SVL	LVSGGGV	DTPDSSSSSRGIDRTPIVGLFQIPASPEDGTASTATTVQMFGGALYQALSPYVLFTFVQARSGGSVLLVSGGGV	1	0.10
OC (2020)_MLG103	Oceania	DT	PDSSSSNRGI	DR	TPIVGLSQIPA	SPEDGTASTATTVHM	FGG	AF	YQ	AL	SPYV	FFTFV	HARSGG	SVL	LVSGGGV	DTPDSSSSNRGIDRTPIVGLSQIPASPEDGTASTATTVHMFGGAFYQALSPYVFFTFVHARSGGSVLLVSGGGV	1	0.10
OC (2020)_MLG104	Oceania	DT	PYSSSSSKRI	DR	TPIVGLSQIPA	SPEDGTASTATTVQM	FGG	AL	YQ	AL	SPYV	LFTFV	QARSGG	SVL	LVSGGGV	DTPYSSSSSKRIDRTPIVGLSQIPASPEDGTASTATTVQMFGGALYQALSPYVLFTFVQARSGGSVLLVSGGGV	1	0.10
OC (2020)_MLG105	Oceania	DT	PGSSSSSRGI	DR	TPIVGLSQIPA	LPEDGTASTATTVQM	FGG	AF	YQ	AL	SPYV	LFTFV	HARSGV	SVL	LVSGDGV	DTPGSSSSSRGIDRTPIVGLSQIPALPEDGTASTATTVQMFGGAFYQALSPYVLFTFVHARSGVSVLLVSGDGV	1	0.10
OC (2020)_MLG106	Oceania	DT	PGSSSSSRGI	DR	IPIVGLSQIPA	SPEDGTASTATTVQM	FGG	AF	YQ	AL	SPYV	LFTFV	HARSGV	SVS	LVSGGGV	DTPGSSSSSRGIDRIPIVGLSQIPASPEDGTASTATTVQMFGGAFYQALSPYVLFTFVHARSGVSVSLVSGGGV	1	0.10
OC (2020)_MLG107	Oceania	DT	PDSSSSSRGI	DR	IPIVGLSQIPA	SPEDGTASKATTVQM	FGG	AL	YQ	AL	SPYV	LFTFV	HARSGG	SVL	LVSGGGV	DTPDSSSSSRGIDRIPIVGLSQIPASPEDGTASKATTVQMFGGALYQALSPYVLFTFVHARSGGSVLLVSGGGV	1	0.10
OC (2020)_MLG108	Oceania	DT	PDSSSSSRGI	DR	TPIVGLSQIPA	SPEDGTASTATTVHM	FGG	AL	YQ	AL	SPYV	LFTFV	HARSGG	SVL	LASGGGV	DTPDSSSSSRGIDRTPIVGLSQIPASPEDGTASTATTVHMFGGALYQALSPYVLFTFVHARSGGSVLLASGGGV	1	0.10
OC (2020)_MLG109	Oceania	DT	PDSSSPSRGI	DR	TPIVGLSQIPA	LPEDGTASTATTVQM	FGG	AL	YQ	AL	SPYV	LFTFV	QARSGG	SVL	LVSGGGV	DTPDSSSPSRGIDRTPIVGLSQIPALPEDGTASTATTVQMFGGALYQALSPYVLFTFVQARSGGSVLLVSGGGV	1	0.10
OC (2020)_MLG110	Oceania	DT	PDSSSSSRGI	DR	IPIVGLSQIPA	SPEDVTVSTATTVQM	FGV	AL	YQ	AL	SPYV	LFTFV	HARSGG	SFL	LVSGGVV	DTPDSSSSSRGIDRIPIVGLSQIPASPEDVTVSTATTVQMFGVALYQALSPYVLFTFVHARSGGSFLLVSGGVV	1	0.10
OC (2020)_MLG111	Oceania	DT	PDSSSSSRGI	ER	TPIVGLSQIPA	SLEDGTASTATTVQM	FGG	AL	YQ	AP	SPYV	LLTFV	QARSGG	SLS	LVSGDGV	DTPDSSSSSRGIERTPIVGLSQIPASLEDGTASTATTVQMFGGALYQAPSPYVLLTFVQARSGGSLSLVSGDGV	1	0.10
OC (2020)_MLG112	Oceania	DT	PDSSSSSRGI	DR	TPIVGLSQVSA	SPEDVTASTATTVQM	FGG	AF	YQ	AP	SPYV	LFTFV	QARSGV	SVL	LVSGDGV	DTPDSSSSSRGIDRTPIVGLSQVSASPEDVTASTATTVQMFGGAFYQAPSPYVLFTFVQARSGVSVLLVSGDGV	1	0.10
OC (2020)_MLG113	Oceania	DT	PDSSSSSRGI	DR	TSIIGLSQIPA	SPEDGTASTATTVQM	FGG	AF	YQ	AP	SPYV	LFTFV	QARSGG	LVL	LVSGGGV	DTPDSSSSSRGIDRTSIIGLSQIPASPEDGTASTATTVQMFGGAFYQAPSPYVLFTFVQARSGGLVLLVSGGGV	1	0.10
OC (2020)_MLG114	Oceania	DT	PGSSSSSRGI	DR	IPIVGLSQIPA	SLEDGTASTATTVQM	FGG	AL	YQ	AL	SPYV	LFTFV	HARSGV	SVL	LVSGGGV	DTPGSSSSSRGIDRIPIVGLSQIPASLEDGTASTATTVQMFGGALYQALSPYVLFTFVHARSGVSVLLVSGGGV	1	0.10
OC (2020)_MLG115	Oceania	DT	PDSSSSSRGI	DR	IPIVGLSQIPA	SPEDGTASTATTVQM	FGG	AL	YQ	AL	SPYV	LFTFV	HARSGG	SVL	LVSGDGV	DTPDSSSSSRGIDRIPIVGLSQIPASPEDGTASTATTVQMFGGALYQALSPYVLFTFVHARSGGSVLLVSGDGV	1	0.10
OC (2020)_MLG116	Oceania	DT	LDSSSSSRGI	DR	TPIVGLSQIPA	LPEDGTASKATTVQM	FGG	AF	YQ	VP	SPYV	LFTFV	QARSGG	SVL	LVSGDGV	DTLDSSSSSRGIDRTPIVGLSQIPALPEDGTASKATTVQMFGGAFYQVPSPYVLFTFVQARSGGSVLLVSGDGV	1	0.10
OC (2020)_MLG117	Oceania	DT	PDSSSSSKRI	DR	TPFVGLSQIPA	SPEDGTASTATTVQM	FGG	AF	YQ	AL	SPYV	LFTFV	QARSGG	SVL	LVNGGGV	DTPDSSSSSKRIDRTPFVGLSQIPASPEDGTASTATTVQMFGGAFYQALSPYVLFTFVQARSGGSVLLVNGGGV	1	0.10
OC (2020)_MLG118	Oceania	DT	PDSSSSSRGI	DR	TPIVGLSQIPA	SPEDGTASTATTVQM	FGG	AL	YQ	AL	SPYV	LFTFV	HARSGG	SVL	LVIGGGV	DTPDSSSSSRGIDRTPIVGLSQIPASPEDGTASTATTVQMFGGALYQALSPYVLFTFVHARSGGSVLLVIGGGV	1	0.10
OC (2020)_MLG119	Oceania	DT	PDSSSSSKRI	DR	TPIVGLSQIPA	SPEDGIASTATTVQM	FSG	AL	YQ	AL	SPYV	LFTFV	QARSGG	SVL	LVSGGVV	DTPDSSSSSKRIDRTPIVGLSQIPASPEDGIASTATTVQMFSGALYQALSPYVLFTFVQARSGGSVLLVSGGVV	1	0.10
OC (2020)_MLG120	Oceania	DT	PDSSSSSRGI	DR	TPIVGLSQIPA	SPEDVTASTATTVQM	FGG	AL	YQ	AL	SPYV	LFTFV	HARSGG	SVL	LVSGGGV	DTPDSSSSSRGIDRTPIVGLSQIPASPEDVTASTATTVQMFGGALYQALSPYVLFTFVHARSGGSVLLVSGGGV	1	0.10
OC (2020)_MLG121	Oceania	DT	PDSSSSSRGI	ER	TPIVGLSQIPA	SLEDVTASTATTVQM	FGG	AL	YQ	AP	SPYV	LFTFV	QARSGG	SLS	LVSGDVV	DTPDSSSSSRGIERTPIVGLSQIPASLEDVTASTATTVQMFGGALYQAPSPYVLFTFVQARSGGSLSLVSGDVV	1	0.10

OC (2020)_MLG122	Oceania	DT	PDSSSSSRGI	DR	TPIVGLSQIPA	SPEDVTASTATTVQM	FGG	AL	YQ	AL	SPYV	LFTFV	QARSGG	SVL	LVSGDVV	DTPDSSSSSRGIDRTPIVGLSQIPASPEDVTASTATTVQMFGGALYQALSPYVLFTFVQARSGGSVLLVSGDVV	1	0.10
OC (2020)_MLG123	Oceania	DT	PDSSSSSRGI	DR	TPIVGLSQISA	SPEDVTASTATTVQM	FGG	AF	YQ	AP	SPYV	LFTFV	QARSGV	SVL	LVSGDGV	DTPDSSSSSRGIDRTPIVGLSQISASPEDVTASTATTVQMFGGAFYQAPSPYVLFTFVQARSGVSVLLVSGDGV	1	0.10
OC (2020)_MLG124	Oceania	DT	LDSSSLSRGI	DR	TPIVGLSQIPA	SPEDVTASTATTVQM	YGG	AL	YQ	AP	SPYV	LFTFV	QARSVG	SVS	LVSGDVV	DTLDSSSLSRGIDRTPIVGLSQIPASPEDVTASTATTVQMYGGALYQAPSPYVLFTFVQARSVGSVSLVSGDVV	1	0.10
OC (2020)_MLG125	Oceania	DT	PDSSSSSRGI	DR	IPIVGLSQIPA	SPEDGTASTATTVQM	FGG	AL	YQ	AP	SPYV	LFTFV	HARSGV	SVL	LVSGDGV	DTPDSSSSSRGIDRIPIVGLSQIPASPEDGTASTATTVQMFGGALYQAPSPYVLFTFVHARSGVSVLLVSGDGV	1	0.10
OC (2020)_MLG126	Oceania	DT	LDSSSLSRGI	DR	TPIVGLSQIPA	SPEDGTASTATTVQM	YGG	AF	YQ	AP	SPYV	LFTFV	QARSVG	SVS	LVSGDGV	DTLDSSSLSRGIDRTPIVGLSQIPASPEDGTASTATTVQMYGGAFYQAPSPYVLFTFVQARSVGSVSLVSGDGV	1	0.10
OC (2020)_MLG127	Oceania	DT	PDSSSSSKRI	DR	TPIVGLSQIPA	SPEDGTASTATTVQM	FGG	AL	YQ	AL	SPYV	LFTFV	QARSGC	SVL	LVSGEGV	DTPDSSSSSKRIDRTPIVGLSQIPASPEDGTASTATTVQMFGGALYQALSPYVLFTFVQARSGCSVLLVSGEGV	1	0.10
OC (2020)_MLG128	Oceania	DT	LDSSSLSRGI	DC	TPIVGLSQIPA	SPEDGTASTATTVQM	YGG	AL	YQ	AP	SPYV	LFTFV	QARSVG	SVS	LVSGDGV	DTLDSSSLSRGIDCTPIVGLSQIPASPEDGTASTATTVQMYGGALYQAPSPYVLFTFVQARSVGSVSLVSGDGV	1	0.10
OC (2020)_MLG129	Oceania	DT	LDSSSSSRGI	DR	TPIVGLSQIPA	SPEDVTASKATTVQM	FGG	AL	YQ	VP	SPYV	LFTFV	QARSGG	SVL	LVSGDVV	DTLDSSSSSRGIDRTPIVGLSQIPASPEDVTASKATTVQMFGGALYQVPSPYVLFTFVQARSGGSVLLVSGDVV	1	0.10
OC (2020)_MLG130	Oceania	DT	LDSSSSSKRI	DR	TPIVGLSQIPA	SPEDGTASTATTVQM	FGG	AL	YQ	AL	SPYV	LFTFV	QARSGG	SVL	LVSGGGV	DTLDSSSSSKRIDRTPIVGLSQIPASPEDGTASTATTVQMFGGALYQALSPYVLFTFVQARSGGSVLLVSGGGV	1	0.10
OC (2020)_MLG131	Oceania	DT	PDSSSSSKRI	DR	TPIVGLSQIPA	SPEDGIASTATTVQM	FSG	AF	YQ	AP	SPYV	LFTFV	QARSGG	SVL	LVSGGGV	DTPDSSSSSKRIDRTPIVGLSQIPASPEDGIASTATTVQMFSGAFYQAPSPYVLFTFVQARSGGSVLLVSGGGV	1	0.10
OC (2020)_MLG132	Oceania	DT	PDSSSSSRGI	DR	TPIVGLSQIPA	SPEDGTASTATTVQM	FGG	AL	YQ	AP	PPYV	LFTFV	HARSGG	SVL	LVSGDGV	DTPDSSSSSRGIDRTPIVGLSQIPASPEDGTASTATTVQMFGGALYQAPPPYVLFTFVHARSGGSVLLVSGDGV	1	0.10
OC (2020)_MLG133	Oceania	DT	PDSSSSSKRI	DR	TPIVGLSQIPA	SPEEGTASTATTVQM	FGG	AL	YQ	AL	SPYV	LFTFV	QARSGG	SVL	LVSGDGV	DTPDSSSSSKRIDRTPIVGLSQIPASPEEGTASTATTVQMFGGALYQALSPYVLFTFVQARSGGSVLLVSGDGV	1	0.10
OC (2020)_MLG134	Oceania	DT	PDSSSSSRGI	DR	TPIVGLSQIPA	SPEDGTASTATTVQM	FGG	AF	YQ	AP	SLCV	LFTFV	QARSGG	SVS	LVSGDGV	DTPDSSSSSRGIDRTPIVGLSQIPASPEDGTASTATTVQMFGGAFYQAPSLCVLFTFVQARSGGSVSLVSGDGV	1	0.10
OC (2020)_MLG135	Oceania	DT	PDSSSSSRGI	DR	IPIVGLSQIPA	SPEDGTASTATTVQM	FGG	AL	YQ	AL	SPYV	LFTFV	HARSGG	SVL	LVSGGVV	DTPDSSSSSRGIDRIPIVGLSQIPASPEDGTASTATTVQMFGGALYQALSPYVLFTFVHARSGGSVLLVSGGVV	1	0.10
OC (2020)_MLG136	Oceania	DT	PDSSSSSRGI	DR	TPIVDLSQIPT	SPEDGTASTATTVQM	FGG	AF	YQ	AP	SPYV	LFTFV	QARSGG	SVL	LVSGDGV	DTPDSSSSSRGIDRTPIVDLSQIPTSPEDGTASTATTVQMFGGAFYQAPSPYVLFTFVQARSGGSVLLVSGDGV	1	0.10
OC (2020)_MLG137	Oceania	DT	PDSSSSSKRT	DR	TPIVGLSQIPA	SPEDGTASTATTVQM	FGG	AL	YQ	AL	SPYV	LFTFV	QARSGG	SVL	LVSGGGV	DTPDSSSSSKRTDRTPIVGLSQIPASPEDGTASTATTVQMFGGALYQALSPYVLFTFVQARSGGSVLLVSGGGV	1	0.10
OC (2020)_MLG138	Oceania	DT	PDSSSSNRGI	DR	TPIVGLSQIPA	SPEDGTASTATTVQM	FGG	AF	YQ	AP	SPYV	LFTFV	QARSGG	SVS	LVSGDGV	DTPDSSSSNRGIDRTPIVGLSQIPASPEDGTASTATTVQMFGGAFYQAPSPYVLFTFVQARSGGSVSLVSGDGV	1	0.10
OC (2020)_MLG139	Oceania	DT	PDSSSSSSGI	DR	IPIVGLSQIPA	SPEDGTASTATTVQM	FGG	AL	YQ	AL	SPYV	LFTFV	HARSGG	LVL	LVSGGGV	DTPDSSSSSSGIDRIPIVGLSQIPASPEDGTASTATTVQMFGGALYQALSPYVLFTFVHARSGGLVLLVSGGGV	1	0.10
OC (2020)_MLG140	Oceania	DT	PDSSSSSRGI	DR	IPIVGLSQIPA	SPEDGTASTATTVQM	FGG	AL	YQ	AL	SPYV	LFTFL	HARSGG	SVL	LVSGGGV	DTPDSSSSSRGIDRIPIVGLSQIPASPEDGTASTATTVQMFGGALYQALSPYVLFTFLHARSGGSVLLVSGGGV	1	0.10
OC (2020)_MLG141	Oceania	DT	PGSSSSSRGI	DR	TPIVGLSQIPA	LPEDGTASTATTVQM	FGG	AL	YQ	AP	SPYV	LFTFV	QARSGV	SVL	LVSGDGV	DTPGSSSSSRGIDRTPIVGLSQIPALPEDGTASTATTVQMFGGALYQAPSPYVLFTFVQARSGVSVLLVSGDGV	1	0.10
OC (2020)_MLG142	Oceania	DT	LDSSSLSRGI	DR	TPIVGLSQIPA	SPEDGTASTATTVQL	YGG	AL	YQ	AP	SPYV	LFTFV	QARSVG	SVS	LVSGDGV	DTLDSSSLSRGIDRTPIVGLSQIPASPEDGTASTATTVQLYGGALYQAPSPYVLFTFVQARSVGSVSLVSGDGV	1	0.10
OC (2020)_MLG143	Oceania	DT	PDSSSSSRGI	DR	TPIVGLSQIPA	SPEDVTASTAITVQM	FGG	AL	YQ	AL	SPYV	LFTFV	QARSGG	SVL	LVSGGGV	DTPDSSSSSRGIDRTPIVGLSQIPASPEDVTASTAITVQMFGGALYQALSPYVLFTFVQARSGGSVLLVSGGGV	1	0.10
OC (2020)_MLG144	Oceania	DT	PDSSSSSRGI	DR	TPIVGLSQIPA	SPEDGTASTVTTVQM	FGG	AL	YQ	AL	SPYV	LFTFV	QARSGG	SVL	LVSRGGV	DTPDSSSSSRGIDRTPIVGLSQIPASPEDGTASTVTTVQMFGGALYQALSPYVLFTFVQARSGGSVLLVSRGGV	1	0.10
OC (2020)_MLG145	Oceania	DT	PDSSSSSRGI	ER	TPIVGLSQIPA	SLEDGTASTATTVQM	FGG	AL	YQ	AL	SPYV	LLTFV	QARSGV	SLS	LVSGDGV	DTPDSSSSSRGIERTPIVGLSQIPASLEDGTASTATTVQMFGGALYQALSPYVLLTFVQARSGVSLSLVSGDGV	1	0.10
OC (2020)_MLG146	Oceania	DT	PDSSASSRGI	DR	TPIVGLSQIPA	SPEDGTASTATTVQM	FGG	AL	YQ	AP	SLCV	LFTFV	QARSGG	SVS	LVSGDGV	DTPDSSASSRGIDRTPIVGLSQIPASPEDGTASTATTVQMFGGALYQAPSLCVLFTFVQARSGGSVSLVSGDGV	1	0.10
OC (2020)_MLG147	Oceania	DT	PDSSSSSRGI	DR	TPIVGLSQIPA	SPEDGTASTATTVQM	FGG	AL	YQ	AP	SPYV	LFTFV	QARSVG	SVL	LVSGGGV	DTPDSSSSSRGIDRTPIVGLSQIPASPEDGTASTATTVQMFGGALYQAPSPYVLFTFVQARSVGSVLLVSGGGV	1	0.10
OC (2020)_MLG148	Oceania	DT	LDSSSSSRGI	DR	TPIVGLSQIPA	SPEDGTARKATTVQM	FGG	AF	YQ	VP	SPYV	LFTFV	QARSGG	SVL	LVSGDVV	DTLDSSSSSRGIDRTPIVGLSQIPASPEDGTARKATTVQMFGGAFYQVPSPYVLFTFVQARSGGSVLLVSGDVV	1	0.10
OC (2020)_MLG149	Oceania	DT	PDSSSSSRGI	DR	TSIIGLSQIPA	LPEDGTASTATTVQM	FGG	AF	YQ	AP	SPYV	LFTFV	QARSGG	LVL	LVSGDGV	DTPDSSSSSRGIDRTSIIGLSQIPALPEDGTASTATTVQMFGGAFYQAPSPYVLFTFVQARSGGLVLLVSGDGV	1	0.10
OC (2020)_MLG150	Oceania	DT	PDSSSSSRGI	DR	IPIVGLSQIPA	SPEDGTASTVTTVQM	FGG	AL	YQ	AL	SPYV	LFTFV	HARSGG	SVL	LVSGGVV	DTPDSSSSSRGIDRIPIVGLSQIPASPEDGTASTVTTVQMFGGALYQALSPYVLFTFVHARSGGSVLLVSGGVV	1	0.10
OC (2020)_MLG151	Oceania	DT	PDSSSSSRGI	DR	IPIVGLSQIPA	SPEDGTASTVTTVQM	FGG	AF	YQ	AL	SPYV	LFTFV	HARSGG	SVL	LVSGGGV	DTPDSSSSSRGIDRIPIVGLSQIPASPEDGTASTVTTVQMFGGAFYQALSPYVLFTFVHARSGGSVLLVSGGGV	1	0.10
OC (2020)_MLG152	Oceania	DT	PDSSLSSRGI	DR	TPIVGLSQIPA	SPEDGTASTATTVQM	FGG	AL	YQ	AL	SPYV	LFTFV	HARFGG	SVL	LVSGGGV	DTPDSSLSSRGIDRTPIVGLSQIPASPEDGTASTATTVQMFGGALYQALSPYVLFTFVHARFGGSVLLVSGGGV	1	0.10
OC (2020)_MLG153	Oceania	GT	PDSSSSSRGI	DR	TPIVGLSQIPA	SPEDGTASTATTVQM	FGG	AF	YQ	AL	SPYV	LFTFV	QARSGG	SVL	LVSGGGV	GTPDSSSSSRGIDRTPIVGLSQIPASPEDGTASTATTVQMFGGAFYQALSPYVLFTFVQARSGGSVLLVSGGGV	1	0.10
OC (2020)_MLG154	Oceania	DT	PDSSSSSKRI	DR	TPIVGLSQIPA	SPEDGTASTATTVQM	FGG	AL	YQ	AL	SPYV	LFTFV	QARSGG	SVL	LVSGGVV	DTPDSSSSSKRIDRTPIVGLSQIPASPEDGTASTATTVQMFGGALYQALSPYVLFTFVQARSGGSVLLVSGGVV	1	0.10
OC (2020)_MLG155	Oceania	DT	PDSSSSSRGI	DR	TPIVGLSQIPA	SSEDGTASTATTVQM	FGG	AL	YQ	AL	SPYV	LFTFV	QARSGG	SVL	LVSGGGV	DTPDSSSSSRGIDRTPIVGLSQIPASSEDGTASTATTVQMFGGALYQALSPYVLFTFVQARSGGSVLLVSGGGV	1	0.10
OC (2020)_MLG156	Oceania	DT	PDSSSSSKRI	DR	TSIVGLSQIPA	SPEDGTASTATTVQM	FGG	AL	YQ	AL	SPYV	LFTFV	QARSGG	LVL	LVSGDGV	DTPDSSSSSKRIDRTSIVGLSQIPASPEDGTASTATTVQMFGGALYQALSPYVLFTFVQARSGGLVLLVSGDGV	1	0.10
OC (2020)_MLG157	Oceania	DT	PDSSSSNRGI	DR	TPIVGLSQIPA	SPEDGTASTATTVHM	FGG	AF	YQ	AL	SPYV	LFTFV	HARSGG	SVL	LVSGGGV	DTPDSSSSNRGIDRTPIVGLSQIPASPEDGTASTATTVHMFGGAFYQALSPYVLFTFVHARSGGSVLLVSGGGV	1	0.10
OC (2020)_MLG158	Oceania	DT	PDSSSSSRGI	DR	TPIVGLSQIPA	SPEDGTASTAITVQM	FGG	AL	YQ	AL	SPYV	LFTFV	QARSGG	SVL	LVSGDGV	DTPDSSSSSRGIDRTPIVGLSQIPASPEDGTASTAITVQMFGGALYQALSPYVLFTFVQARSGGSVLLVSGDGV	1	0.10
OC (2020)_MLG159	Oceania	DT	PDSSSSSKGI	DR	TPIVGLSQIPA	SPEDGTASTATTVQM	FGG	AF	YQ	AP	SPYV	LFTFV	QARSGV	SVL	LVSGDGV	DTPDSSSSSKGIDRTPIVGLSQIPASPEDGTASTATTVQMFGGAFYQAPSPYVLFTFVQARSGVSVLLVSGDGV	1	0.10
OC (2020)_MLG160	Oceania	DT	PDYSSSSRGI	DR	IPIVGLSQIPA	SPEDGTASTATTVQM	FGG	AL	YQ	AL	SPYV	LFTFV	HARSGG	SVL	LVSGGGV	DTPDYSSSSRGIDRIPIVGLSQIPASPEDGTASTATTVQMFGGALYQALSPYVLFTFVHARSGGSVLLVSGGGV	1	0.10
OC (2020)_MLG161	Oceania	DT	PDSSSSSKRI	DR	TPIVGLSQIPA	SPEDGTASTATTVQM	FSG	AL	YQ	AL	SPYV	LFTFV	QARSGG	SVL	LVSGGGV	DTPDSSSSSKRIDRTPIVGLSQIPASPEDGTASTATTVQMFSGALYQALSPYVLFTFVQARSGGSVLLVSGGGV	1	0.10
OC (2020)_MLG162	Oceania	DT	PDSSSSSRGI	DR	TSIIGFSQIPA	SPEDVTASTATTVQM	FGG	AF	YQ	AP	SPYV	LFTFV	QARSGG	LLL	LVSGDVV	DTPDSSSSSRGIDRTSIIGFSQIPASPEDVTASTATTVQMFGGAFYQAPSPYVLFTFVQARSGGLLLLVSGDVV	1	0.10
OC (2020)_MLG163	Oceania	DT	PDSSSSSRGI	DR	TPIVGLSQIPA	SSEDGTASTATTVQM	FGG	AL	YQ	AL	SPYV	LFTFV	HARSGG	SVL	LVSGGGV	DTPDSSSSSRGIDRTPIVGLSQIPASSEDGTASTATTVQMFGGALYQALSPYVLFTFVHARSGGSVLLVSGGGV	1	0.10
OC (2020)_MLG164	Oceania	DT	PDSSSSSKRI	DR	TPIVGLSQIPA	SPEDGTASTATTVQM	FGG	AL	YQ	AL	SLYV	LFTFV	QARSGG	SVL	LVSGGGV	DTPDSSSSSKRIDRTPIVGLSQIPASPEDGTASTATTVQMFGGALYQALSLYVLFTFVQARSGGSVLLVSGGGV	1	0.10
OC (2020)_MLG165	Oceania	DT	PDSSSSSRGI	DR	IPIVGLSQIPA	LPEDGTASTATTVQM	FGG	AL	YQ	AP	SPYV	LFTFV	HARSGG	SVL	LVSGGGV	DTPDSSSSSRGIDRIPIVGLSQIPALPEDGTASTATTVQMFGGALYQAPSPYVLFTFVHARSGGSVLLVSGGGV	1	0.10
OC (2020)_MLG166	Oceania	DT	PDSSSSSKRI	DR	TPIVGLFQIPA	SPEDGTASTATTVQM	FGG	AL	YQ	AL	SPYV	LFTFV	QARSGG	SVL	LVSGGGV	DTPDSSSSSKRIDRTPIVGLFQIPASPEDGTASTATTVQMFGGALYQALSPYVLFTFVQARSGGSVLLVSGGGV	1	0.10
OC (2020)_MLG167	Oceania	DT	PDSSSSSRGI	DR	TPIVGLSQIPA	SPEDGTASTATTVQM	FGG	AF	YQ	AP	SPCV	LFTFV	QARSGV	SVL	LVSGDGV	DTPDSSSSSRGIDRTPIVGLSQIPASPEDGTASTATTVQMFGGAFYQAPSPCVLFTFVQARSGVSVLLVSGDGV	1	0.10
OC (2020)_MLG168	Oceania	DT	PDSSSSSRGI	DR	TPIVGLSQIPA	SPEDGTASTATTVQM	FGG	AL	YQ	AP	SLCV	LFTFV	QARSGG	SVS	LVSGDVV	DTPDSSSSSRGIDRTPIVGLSQIPASPEDGTASTATTVQMFGGALYQAPSLCVLFTFVQARSGGSVSLVSGDVV	1	0.10
OC (2020)_MLG169	Oceania	DT	PDSSSSSRGI	DR	TPIVGLSQIPA	SPEDGTASTATTVQM	FGG	AL	YQ	AL	SPYL	LFTFV	QARSGG	SVL	LVSGGGV	DTPDSSSSSRGIDRTPIVGLSQIPASPEDGTASTATTVQMFGGALYQALSPYLLFTFVQARSGGSVLLVSGGGV	1	0.10
OC (2020)_MLG170	Oceania	DT	PYSSSSSKRI	DR	TPIVGLSQIPA	SPEDVTASTATTVQM	FGG	AL	YQ	AL	SPYV	LFTFV	QARSGG	SVL	LVSGGVV	DTPYSSSSSKRIDRTPIVGLSQIPASPEDVTASTATTVQMFGGALYQALSPYVLFTFVQARSGGSVLLVSGGVV	1	0.10
OC (2020)_MLG171	Oceania	GT	PDSSSSSRGI	DR	TPIVGLSQIPA	SPEDVTASTATTVQM	FGG	AL	YQ	AL	SPYV	LFTFV	QARSGG	SVL	LVSGGVV	GTPDSSSSSRGIDRTPIVGLSQIPASPEDVTASTATTVQMFGGALYQALSPYVLFTFVQARSGGSVLLVSGGVV	1	0.10
OC (2020)_MLG172	Oceania	DT	PDSSSSSRGI	DR	TPIVGLSQIPA	SPEDGTASTATTVQM	FGG	AL	YQ	AL	SPYV	LFTFV	HARSGG	SVL	LVSGDGV	DTPDSSSSSRGIDRTPIVGLSQIPASPEDGTASTATTVQMFGGALYQALSPYVLFTFVHARSGGSVLLVSGDGV	1	0.10
OC (2020)_MLG173	Oceania	DT	LDSSSSSRGI	DR	TPIVGLSQIPA	SPEDGTASTATTVQM	YGG	AL	YQ	AP	SPYV	LFTFV	QARSVG	SVL	LVSGGGV	DTLDSSSSSRGIDRTPIVGLSQIPASPEDGTASTATTVQMYGGALYQAPSPYVLFTFVQARSVGSVLLVSGGGV	1	0.10
OC (2020)_MLG174	Oceania	DT	PDSSSSSKRI	DR	TPIVGLSQIPA	SPEDGTASTATTVQM	FSG	AL	YQ	AP	SPYV	LFTFV	QARSGG	SVL	LVSGGGV	DTPDSSSSSKRIDRTPIVGLSQIPASPEDGTASTATTVQMFSGALYQAPSPYVLFTFVQARSGGSVLLVSGGGV	1	0.10
OC (2020)_MLG175	Oceania	DT	PDSSSSSRGI	DR	TSIIGLSQIPA	SPEDVTASTATTVQM	FGG	AF	YQ	AP	SPYV	LFTFV	QARSGG	LFL	LVSGDVV	DTPDSSSSSRGIDRTSIIGLSQIPASPEDVTASTATTVQMFGGAFYQAPSPYVLFTFVQARSGGLFLLVSGDVV	1	0.10
OC (2020)_MLG176	Oceania	DT	PDSSSSSRGI	DR	TSIIGLSQIPA	SPEDGTASTATTVQM	FGG	AL	YQ	AP	SPYV	LFTFV	QARSGG	LVL	LVSGDVV	DTPDSSSSSRGIDRTSIIGLSQIPASPEDGTASTATTVQMFGGALYQAPSPYVLFTFVQARSGGLVLLVSGDVV	1	0.10
OC (2020)_MLG177	Oceania	DM	PDSSSSSRGI	DR	IPIVGLSQIPA	SPEDGTASIVTTVQM	FGG	AL	YQ	AL	SPYV	LFTFV	HARSGG	SVL	LVSGGGV	DMPDSSSSSRGIDRIPIVGLSQIPASPEDGTASIVTTVQMFGGALYQALSPYVLFTFVHARSGGSVLLVSGGGV	1	0.10
OC (2020)_MLG178	Oceania	DT	PDSSSSSRGI	DR	TPIVGLSQIPA	SPEDGTASTATSVHM	FGG	AL	YQ	AL	SPYV	LFTFV	HARSGG	SVL	LVSGGGV	DTPDSSSSSRGIDRTPIVGLSQIPASPEDGTASTATSVHMFGGALYQALSPYVLFTFVHARSGGSVLLVSGGGV	1	0.10
OC (2020)_MLG179	Oceania	DT	PDSSSSSKRI	DR	TPIVGLSQIPA	SPEDGTASTATTVQM	FGG	AL	YQ	AL	SPYV	LFTFV	QARSGG	SVL	LVSGDGV	DTPDSSSSSKRIDRTPIVGLSQIPASPEDGTASTATTVQMFGGALYQALSPYVLFTFVQARSGGSVLLVSGDGV	1	0.10
OC (2020)_MLG180	Oceania	DT	PDSSLSSRGI	DR	TPIVGLSQIPA	SPEDGTASTATTVQM	FGG	AL	YQ	AL	SPYV	LFTFV	HARSGG	SVL	LVSGDGV	DTPDSSLSSRGIDRTPIVGLSQIPASPEDGTASTATTVQMFGGALYQALSPYVLFTFVHARSGGSVLLVSGDGV	1	0.10
OC (2020)_MLG181	Oceania	DT	PDSSSSSKRI	DR	TPFVGLSQVPA	SPEDGTASTATTVQM	FGG	AL	YQ	AL	SPYV	LFTFV	QARSGG	SVL	LVNGGGV	DTPDSSSSSKRIDRTPFVGLSQVPASPEDGTASTATTVQMFGGALYQALSPYVLFTFVQARSGGSVLLVNGGGV	1	0.10
SA (2020)_MLG1	S America	DT	PDSSSSSKRT	DR	TPIVGLSQIPA	SPEDGTASTATTVQM	FGG	AL	YQ	AL	SPYV	LFTFV	QARSGG	SVL	LVSGGGV	DTPDSSSSSKRTDRTPIVGLSQIPASPEDGTASTATTVQMFGGALYQALSPYVLFTFVQARSGGSVLLVSGGGV	89	25.60
SA (2020)_MLG2	S America	DT	PDSSSSSRGI	DR	TPIVGLSQIPA	SPEDGTASTATTVQM	FGG	AL	YQ	AL	SPYV	LFTFV	QARSGG	SVL	LVSGGGV	DTPDSSSSSRGIDRTPIVGLSQIPASPEDGTASTATTVQMFGGALYQALSPYVLFTFVQARSGGSVLLVSGGGV	58	16.70
SA (2020)_MLG3	S America	DT	PDSSSSSKRI	DR	TPIVGLSQIPA	SPEDGTASTATTVQM	FGG	AL	YQ	AL	SPYV	LFTFV	QARSGG	SVL	LVSGGGF	DTPDSSSSSKRIDRTPIVGLSQIPASPEDGTASTATTVQMFGGALYQALSPYVLFTFVQARSGGSVLLVSGGGF	42	12.10
SA (2020)_MLG4	S America	DT	PDSSSSSRGI	DR	TPIVGLSQIPA	SPEDGTASTATTVQM	FGG	AL	YQ	AL	SPYV	LFTFV	HARSGG	SVL	LVSGGGV	DTPDSSSSSRGIDRTPIVGLSQIPASPEDGTASTATTVQMFGGALYQALSPYVLFTFVHARSGGSVLLVSGGGV	35	10.10

SA (2020)_MLG5	S America	DT	PDSSSSSRGI	DR	IPIVGLSQIPA	SPEDGTASTATTVQM	FGG	AL	YQ	AL	SPYV	LFTFV	HARSGG	SVL	LVSGGGV	DTPDSSSSSRGIDRIPIVGLSQIPASPEDGTASTATTVQMFGGALYQALSPYVLFTFVHARSGGSVLLVSGGGV	32	9.20
SA (2020)_MLG6	S America	DT	PDSSSSSKRI	DR	TPIVGLSQIPA	SPEDGTASTATTVQM	FGG	AL	YQ	AL	SPYV	LFTFV	QARSGG	SVL	LVSGGGV	DTPDSSSSSKRIDRTPIVGLSQIPASPEDGTASTATTVQMFGGALYQALSPYVLFTFVQARSGGSVLLVSGGGV	30	8.60
SA (2020)_MLG7	S America	DT	PDSSSSSRGI	DR	TPIVGLSQIPA	SPEDGTASTATTVQM	FGG	AF	YQ	AP	SPYV	LFTFV	QARSGV	SVL	LVSGDGV	DTPDSSSSSRGIDRTPIVGLSQIPASPEDGTASTATTVQMFGGAFYQAPSPYVLFTFVQARSGVSVLLVSGDGV	6	1.70
SA (2020)_MLG8	S America	DT	PDSSSSSRGI	DR	TPIVGLSQIPA	SPEDGTASTATTVQM	FGG	AL	YQ	AP	SPYV	LFTFV	QARSGG	SVS	LVSGDGV	DTPDSSSSSRGIDRTPIVGLSQIPASPEDGTASTATTVQMFGGALYQAPSPYVLFTFVQARSGGSVSLVSGDGV	6	1.70
SA (2020)_MLG9	S America	DT	PDSSSSSRGI	DR	TPIVGLSQIPA	SPEDGTASTATTVQM	FGG	AL	YQ	AP	SPYV	LFTFV	QARSGG	SVL	LVSGDGV	DTPDSSSSSRGIDRTPIVGLSQIPASPEDGTASTATTVQMFGGALYQAPSPYVLFTFVQARSGGSVLLVSGDGV	4	1.10
SA (2020)_MLG10	S America	DT	PDSSSSSRGI	DR	TPIVGLSQIPA	SPEDGTASTATTVQM	FGG	AL	YQ	AP	SPYV	LFTFV	QARSGG	SVL	LVSGGGV	DTPDSSSSSRGIDRTPIVGLSQIPASPEDGTASTATTVQMFGGALYQAPSPYVLFTFVQARSGGSVLLVSGGGV	4	1.10
SA (2020)_MLG11	S America	DT	PDSSSLSRGI	DR	TPIVGLSQIPA	SPEDGTASTATTVQM	YGG	AL	YQ	AP	SPYV	LFTFV	QARSVG	SVS	LVSGDGV	DTPDSSSLSRGIDRTPIVGLSQIPASPEDGTASTATTVQMYGGALYQAPSPYVLFTFVQARSVGSVSLVSGDGV	3	0.90
SA (2020)_MLG12	S America	DM	PDSSSSSKRI	DR	TPIVGLSQIPA	SPEDGTASTATTVQM	FGG	AL	YQ	AL	SPYV	LFTFV	QARSGG	SVL	LVSGGGV	DMPDSSSSSKRIDRTPIVGLSQIPASPEDGTASTATTVQMFGGALYQALSPYVLFTFVQARSGGSVLLVSGGGV	3	0.90
SA (2020)_MLG13	S America	DT	PDSSSSSRGI	DR	TPIVGLSQIPA	SPEDGTASTATTVQM	FGG	AL	YQ	AP	SLCV	LFTFV	QARSGG	SVS	LVSGDGV	DTPDSSSSSRGIDRTPIVGLSQIPASPEDGTASTATTVQMFGGALYQAPSLCVLFTFVQARSGGSVSLVSGDGV	3	0.90
SA (2020)_MLG14	S America	DT	PDSSSSSRGI	DR	TPIIGLSQIPA	SPEDGTASTATTVQM	FGG	AF	YQ	AP	SPYV	LFTFV	QARSGG	SVL	LVSGDGV	DTPDSSSSSRGIDRTPIIGLSQIPASPEDGTASTATTVQMFGGAFYQAPSPYVLFTFVQARSGGSVLLVSGDGV	2	0.60
SA (2020)_MLG15	S America	DT	PDSSSSSRGI	DR	IPIVGLSQIPA	SPEDGTASTATTVQM	FGG	AL	YQ	AL	SPYV	LFTFV	HARSGG	SVL	LVSGDGV	DTPDSSSSSRGIDRIPIVGLSQIPASPEDGTASTATTVQMFGGALYQALSPYVLFTFVHARSGGSVLLVSGDGV	2	0.60
SA (2020)_MLG16	S America	GT	PDSSSSSRGI	DR	TPIVGLSQIPA	SPEDGTASTATTVQM	FGG	AL	YQ	AL	SPYV	LFTFV	QARSGG	SVL	LVSGGGV	GTPDSSSSSRGIDRTPIVGLSQIPASPEDGTASTATTVQMFGGALYQALSPYVLFTFVQARSGGSVLLVSGGGV	2	0.60
SA (2020)_MLG17	S America	DT	PDSSSSSRGI	DR	TPIVGLSQIPA	SPEDGTASTATTVQM	FGG	AL	YQ	AP	SPYV	LFTFV	QARAGG	SAL	LVSGDGV	DTPDSSSSSRGIDRTPIVGLSQIPASPEDGTASTATTVQMFGGALYQAPSPYVLFTFVQARAGGSALLVSGDGV	2	0.60
SA (2020)_MLG18	S America	DT	PDSSSSSKRI	DR	TPIVGLSQVPA	SPEDGTASTATTVQM	FGG	AL	YQ	AL	SPYV	LFTFV	QARSGG	SVL	LVSGGGV	DTPDSSSSSKRIDRTPIVGLSQVPASPEDGTASTATTVQMFGGALYQALSPYVLFTFVQARSGGSVLLVSGGGV	2	0.60
SA (2020)_MLG19	S America	DT	PDSSSSSRGI	DR	TPIVGLSQVPA	SPEDGTASTATTVQM	FGG	AL	YQ	AL	SPYV	LFTFV	QARSGG	SVL	LVSGGGV	DTPDSSSSSRGIDRTPIVGLSQVPASPEDGTASTATTVQMFGGALYQALSPYVLFTFVQARSGGSVLLVSGGGV	2	0.60
SA (2020)_MLG20	S America	DT	PDSSSSSKRT	DR	TPIVGLSQVPA	SPEDGTASTATTVQM	FGG	AL	YQ	AL	SPYV	LFTFV	QARSGG	SVL	LVSGGGV	DTPDSSSSSKRTDRTPIVGLSQVPASPEDGTASTATTVQMFGGALYQALSPYVLFTFVQARSGGSVLLVSGGGV	1	0.30
SA (2020)_MLG21	S America	DT	PDSSSSSRGI	DR	TPIVGLSQIPA	SPEDGTASTATTVQM	FGG	AL	YQ	VL	SPYV	LFTFV	QARSGG	SVL	LVSGGGV	DTPDSSSSSRGIDRTPIVGLSQIPASPEDGTASTATTVQMFGGALYQVLSPYVLFTFVQARSGGSVLLVSGGGV	1	0.30
SA (2020)_MLG22	S America	DT	PDSSSSSRGI	DR	TPIVGLSQIPA	SPEDGTASTATTVQM	FGG	AL	YQ	AP	SPYV	LFTFV	QARSGV	SVL	LVSGDGV	DTPDSSSSSRGIDRTPIVGLSQIPASPEDGTASTATTVQMFGGALYQAPSPYVLFTFVQARSGVSVLLVSGDGV	1	0.30
SA (2020)_MLG23	S America	DT	PDSSSSSKRI	DR	TPIVGLSQIPA	SPEDVTASTATTVQM	FGG	AL	YQ	AL	SPYV	LFTFV	QARSGG	SVL	LVSGGGF	DTPDSSSSSKRIDRTPIVGLSQIPASPEDVTASTATTVQMFGGALYQALSPYVLFTFVQARSGGSVLLVSGGGF	1	0.30
SA (2020)_MLG24	S America	DT	PDSSSSSRGT	DR	TPIVGLSQIPA	SPEDGTASTATTVQM	FGG	AL	YQ	AP	SPYV	LFTFV	QARSGG	SVL	LVSGGGV	DTPDSSSSSRGTDRTPIVGLSQIPASPEDGTASTATTVQMFGGALYQAPSPYVLFTFVQARSGGSVLLVSGGGV	1	0.30
SA (2020)_MLG25	S America	DT	PDSSSSSRGI	DR	TPIVGLSQIPA	SPEDGTASTATTVQM	FGG	AL	YQ	AL	SPYV	LFTFV	HARSGG	SVL	LVSGDGV	DTPDSSSSSRGIDRTPIVGLSQIPASPEDGTASTATTVQMFGGALYQALSPYVLFTFVHARSGGSVLLVSGDGV	1	0.30
SA (2020)_MLG26	S America	DT	PDSSSSSKRI	DR	IPIVGLSQIPA	SPEDGTASTATTVQM	FGG	AL	YQ	AL	SPYV	LFTFV	QARSGG	SVL	LVSGGGF	DTPDSSSSSKRIDRIPIVGLSQIPASPEDGTASTATTVQMFGGALYQALSPYVLFTFVQARSGGSVLLVSGGGF	1	0.30
SA (2020)_MLG27	S America	DT	PDSSSSSRGI	DR	TPIVGLSQIPA	SPEDGTASTATTVQM	FGG	AL	YQ	AL	SPYV	LFTFV	HARSGG	SVL	LVIGGGV	DTPDSSSSSRGIDRTPIVGLSQIPASPEDGTASTATTVQMFGGALYQALSPYVLFTFVHARSGGSVLLVIGGGV	1	0.30
SA (2020)_MLG28	S America	DT	PDSSSSSRGI	DR	IPIVGLSQIPA	SPEDGTASTATTVQM	FGG	AL	YQ	AL	SPYV	LFTFV	QARSGG	SVL	LVSGGGV	DTPDSSSSSRGIDRIPIVGLSQIPASPEDGTASTATTVQMFGGALYQALSPYVLFTFVQARSGGSVLLVSGGGV	1	0.30
SA (2020)_MLG29	S America	DT	PDSSSSSRGI	DR	TPIVGLSQIPA	SPEDGTASTATTVQM	FGG	AL	YQ	AP	SPYV	LFTFV	HARSGG	SVL	LVSGGGV	DTPDSSSSSRGIDRTPIVGLSQIPASPEDGTASTATTVQMFGGALYQAPSPYVLFTFVHARSGGSVLLVSGGGV	1	0.30
SA (2020)_MLG30	S America	DT	PDSSSSSKRI	DR	TPIVGLSQIPA	SPEDGIASTATTVQM	FSG	AF	YQ	AL	SPYV	LFTFV	QARSGG	SVL	LVSGGGV	DTPDSSSSSKRIDRTPIVGLSQIPASPEDGIASTATTVQMFSGAFYQALSPYVLFTFVQARSGGSVLLVSGGGV	1	0.30
SA (2020)_MLG31	S America	DT	PDSSSSSRGI	DR	TPIVGLSQIPA	SPEEGTASTATTVQM	FGG	AL	YQ	AL	SPYV	LFTFV	QARSGG	SVL	LVSGGGV	DTPDSSSSSRGIDRTPIVGLSQIPASPEEGTASTATTVQMFGGALYQALSPYVLFTFVQARSGGSVLLVSGGGV	1	0.30
SA (2020)_MLG32	S America	DT	PDSSSSSRGI	DR	TPIVGLSQIPA	SPEDGTASTATTVQM	FGG	AL	YQ	AL	SPYV	LFTFV	QARSGG	SVL	LVSGGGF	DTPDSSSSSRGIDRTPIVGLSQIPASPEDGTASTATTVQMFGGALYQALSPYVLFTFVQARSGGSVLLVSGGGF	1	0.30
SA (2020)_MLG33	S America	DT	PDSSSSSKRT	DR	TPIVGLSQIPA	SSEDGTASTATTVQM	FGG	AF	YQ	AL	SPYV	LFTFV	QARSGG	SVL	LVSGGGV	DTPDSSSSSKRTDRTPIVGLSQIPASSEDGTASTATTVQMFGGAFYQALSPYVLFTFVQARSGGSVLLVSGGGV	1	0.30
SA (2020)_MLG34	S America	DT	PDSSSSSKRI	DR	TPIVGLSQIPA	SPEDGTASTATTVQM	FGS	AF	YQ	AL	SPYV	LFTFV	QARSGG	SVL	LVSGGGV	DTPDSSSSSKRIDRTPIVGLSQIPASPEDGTASTATTVQMFGSAFYQALSPYVLFTFVQARSGGSVLLVSGGGV	1	0.30
SA (2020)_MLG35	S America	DT	PDSSSSSKRI	DR	TPIVGLSQIPA	SPEDGIASTATTVQM	FSG	AL	YQ	AL	SPYV	LFTFV	QARSGG	SVL	LVSGGGV	DTPDSSSSSKRIDRTPIVGLSQIPASPEDGIASTATTVQMFSGALYQALSPYVLFTFVQARSGGSVLLVSGGGV	1	0.30
SA (2020)_MLG36	S America	DT	PDSSSSSKRI	DR	TPIVGLSQIPA	SPEDGTASTATTVQM	FGG	AL	YQ	AL	SPYV	LFTFV	QARFGG	SVL	LVSGGGF	DTPDSSSSSKRIDRTPIVGLSQIPASPEDGTASTATTVQMFGGALYQALSPYVLFTFVQARFGGSVLLVSGGGF	1	0.30
SA (2020)_MLG37	S America	DT	PDSSSSSRGI	DR	TPIVGLSQVSA	SPEDGTASTATTVQM	FGG	AF	YQ	AP	SPYV	LFTFV	QARSGV	SVL	LVSGDGV	DTPDSSSSSRGIDRTPIVGLSQVSASPEDGTASTATTVQMFGGAFYQAPSPYVLFTFVQARSGVSVLLVSGDGV	1	0.30
SA (2020)_MLG38	S America	DT	PDSSLSSRGI	DR	TPIVGLSQIPA	SPEDGTASTATTVQM	FGG	AL	YQ	AL	SPYV	LFTFV	QARSGG	SVL	LVSGGGV	DTPDSSLSSRGIDRTPIVGLSQIPASPEDGTASTATTVQMFGGALYQALSPYVLFTFVQARSGGSVLLVSGGGV	1	0.30
SA (2020)_MLG39	S America	DT	PDSSSSSRGI	DR	TPIVDLSQIPT	SPEDGTASTATTVQM	FGG	AL	YQ	AP	SPYV	LFTFV	QARSGG	SVL	LVSGDGV	DTPDSSSSSRGIDRTPIVDLSQIPTSPEDGTASTATTVQMFGGALYQAPSPYVLFTFVQARSGGSVLLVSGDGV	1	0.30
SA (2020)_MLG40	S America	DT	PDSSSSSRGI	DR	TPIVGLSQVPA	SPEDGTASTATTVQM	FGG	AL	YQ	AL	SPYV	LFTFV	HARSGG	SVL	LVSGGGV	DTPDSSSSSRGIDRTPIVGLSQVPASPEDGTASTATTVQMFGGALYQALSPYVLFTFVHARSGGSVLLVSGGGV	1	0.30
SA (2021)_MLG40	S America	DT	PDSSSSSKRI	DR	TPIVGLSQIDA	PEDGTASTATTVQM	FGG	AL	YQ	AL	SPYV	LFTFV	QARSGG	SVL	LVSGGGF	DTPDSSSSSKRIDRTPIVGLSQIDAPEDGTASTATTVQMFGGALYQALSPYVLFTFVQARSGGSVLLVSGGGF	764	77.88
SA (2021)_MLG41	S America	DT	PDSSSSSKRI	DR	TPIVGLSQIDA	PEDGTASTATTVQM	FGG	AL	YQ	AL	SPYV	LFTFV	QARSGG	SVL	LVSGGGV	DTPDSSSSSKRIDRTPIVGLSQIDAPEDGTASTATTVQMFGGALYQALSPYVLFTFVQARSGGSVLLVSGGGV	53	5.40
SA (2021)_MLG42	S America	DT	PDSSSSSKRI	DR	TPIVGLSQIDA	PEDGTASTATTVQM	FGG	AL	YQ	AP	SPYV	LFTFV	QARSGG	SVL	LVSGGGF	DTPDSSSSSKRIDRTPIVGLSQIDAPEDGTASTATTVQMFGGALYQAPSPYVLFTFVQARSGGSVLLVSGGGF	25	2.55
SA (2021)_MLG43	S America	DT	PDSSSSSKRT	DR	TPIVGLSQIDA	PEDGTASTATTVQM	FGG	AL	YQ	AL	SPYV	LFTFV	QARSGG	SVL	LVSGGGV	DTPDSSSSSKRTDRTPIVGLSQIDAPEDGTASTATTVQMFGGALYQALSPYVLFTFVQARSGGSVLLVSGGGV	19	1.94
SA (2021)_MLG44	S America	DT	LDSSSSSKRI	DR	TPIVGLSQIDA	PEDGTASTATTVQM	FGG	AL	YQ	AL	SPYV	LFTFV	QARSGG	SVL	LVSGGGF	DTLDSSSSSKRIDRTPIVGLSQIDAPEDGTASTATTVQMFGGALYQALSPYVLFTFVQARSGGSVLLVSGGGF	13	1.33
SA (2021)_MLG45	S America	DT	PDSSSSSKRI	DR	TPIVGLSQIDA	PEDGTASTATTVQM	FGG	AL	YQ	AL	SPYV	LFTFV	QARSGG	SVL	LVNGGGF	DTPDSSSSSKRIDRTPIVGLSQIDAPEDGTASTATTVQMFGGALYQALSPYVLFTFVQARSGGSVLLVNGGGF	10	1.02
SA (2021)_MLG46	S America	DT	PDSSSSSKRI	DR	TPIVGLSQIDA	PEDGTASTATTVQM	FGG	AF	YQ	AL	SPYV	LFTFV	QARSGG	SVL	LVSGGGF	DTPDSSSSSKRIDRTPIVGLSQIDAPEDGTASTATTVQMFGGAFYQALSPYVLFTFVQARSGGSVLLVSGGGF	7	0.71
SA (2021)_MLG47	S America	DT	PDSSSSSKRI	DR	TPIVGLSQIDA	PEDGIASTATTVQM	FGG	AL	YQ	AL	SPYV	LFTFV	QARSGG	SVL	LVSGGGF	DTPDSSSSSKRIDRTPIVGLSQIDAPEDGIASTATTVQMFGGALYQALSPYVLFTFVQARSGGSVLLVSGGGF	6	0.61
SA (2021)_MLG48	S America	DT	PDSSSSSKRI	DR	TPIVGLSQIDA	PEDGIASTATTVQM	FSG	AL	YQ	AL	SPYV	LFTFV	QARSGG	SVL	LVSGGGV	DTPDSSSSSKRIDRTPIVGLSQIDAPEDGIASTATTVQMFSGALYQALSPYVLFTFVQARSGGSVLLVSGGGV	6	0.61
SA (2021)_MLG49	S America	DT	PDSSSSSKRI	DR	TPIVGLSQIDA	LEDGTASTATTVQM	FGG	AL	YQ	AL	SPYV	LFTFV	QARSGG	SVL	LVSGGGF	DTPDSSSSSKRIDRTPIVGLSQIDALEDGTASTATTVQMFGGALYQALSPYVLFTFVQARSGGSVLLVSGGGF	6	0.61
SA (2021)_MLG50	S America	DT	PDSSSSSRGI	DR	TPIVGLSQIDA	PEDGTASTATTVQM	FGG	AL	YQ	AL	SPYV	LFTFV	HARSGG	SVL	LVSGGGV	DTPDSSSSSRGIDRTPIVGLSQIDAPEDGTASTATTVQMFGGALYQALSPYVLFTFVHARSGGSVLLVSGGGV	5	0.51
SA (2021)_MLG51	S America	DT	PDSSSSSRGI	DR	TPIVGLSQIDA	PEDGTASTATTVQM	FGG	AL	YQ	AL	SPYV	LFTFV	QARSGG	SVL	LVSGGGV	DTPDSSSSSRGIDRTPIVGLSQIDAPEDGTASTATTVQMFGGALYQALSPYVLFTFVQARSGGSVLLVSGGGV	4	0.41
SA (2021)_MLG52	S America	DT	PDSSSSSKRI	DR	TPIVGLSQIDA	PEDGTASTATTVQM	FGG	AL	YQ	AL	SPYV	LFTFV	QARSGC	SVL	LVSGGGF	DTPDSSSSSKRIDRTPIVGLSQIDAPEDGTASTATTVQMFGGALYQALSPYVLFTFVQARSGCSVLLVSGGGF	4	0.41
SA (2021)_MLG53	S America	DT	PDSSSSSKRI	DR	TPIVGLSQIDA	PEDGTASTATTVQM	FGG	AL	YQ	AL	SPYV	LFTFV	QARSGG	SLL	LVSGGGF	DTPDSSSSSKRIDRTPIVGLSQIDAPEDGTASTATTVQMFGGALYQALSPYVLFTFVQARSGGSLLLVSGGGF	4	0.41
SA (2021)_MLG54	S America	DT	PDSSSSSRGI	DR	IPIVGLSQIDA	PEDGTASTATTVQM	FGG	AL	YQ	AL	SPYV	LFTFV	HARSGG	SVL	LVSGGGV	DTPDSSSSSRGIDRIPIVGLSQIDAPEDGTASTATTVQMFGGALYQALSPYVLFTFVHARSGGSVLLVSGGGV	3	0.31
SA (2021)_MLG55	S America	DT	PDSSSSSKRI	DR	TPIVGLSQIDA	LEDGTASTATTVQM	FGG	AL	YQ	AL	SPYV	LFTFV	QARSGG	SVL	LVSGGGV	DTPDSSSSSKRIDRTPIVGLSQIDALEDGTASTATTVQMFGGALYQALSPYVLFTFVQARSGGSVLLVSGGGV	3	0.31
SA (2021)_MLG56	S America	DT	PDSSSSSKRT	DR	TPIVGLSQIDA	PEDGTASTATTVQM	FGG	AF	YQ	AL	SPYV	LFTFV	QARSGG	SVL	LVSGGGV	DTPDSSSSSKRTDRTPIVGLSQIDAPEDGTASTATTVQMFGGAFYQALSPYVLFTFVQARSGGSVLLVSGGGV	3	0.31
SA (2021)_MLG57	S America	DT	PDSSSSSKRI	DR	TPIVGLSQIDA	PEDGTASTATTVQM	FGG	AL	YQ	AL	SPYV	LFTFV	HARSGG	SVL	LVSGGGF	DTPDSSSSSKRIDRTPIVGLSQIDAPEDGTASTATTVQMFGGALYQALSPYVLFTFVHARSGGSVLLVSGGGF	3	0.31
SA (2021)_MLG58	S America	DT	PDSSSSSRGI	DR	TPIVGLSQIDA	PEDGTASTATTVQM	FGG	AL	YQ	AL	SPYV	LFTFV	QARSGG	SVL	LVSGGGF	DTPDSSSSSRGIDRTPIVGLSQIDAPEDGTASTATTVQMFGGALYQALSPYVLFTFVQARSGGSVLLVSGGGF	2	0.20
SA (2021)_MLG59	S America	DT	LDSSSSSKRI	DR	TPIVGLSQIDA	PEDGIASTATTVQM	FSG	AL	YQ	AL	SPYV	LFTFV	QARSGG	SVL	LVSGGGV	DTLDSSSSSKRIDRTPIVGLSQIDAPEDGIASTATTVQMFSGALYQALSPYVLFTFVQARSGGSVLLVSGGGV	2	0.20
SA (2021)_MLG60	S America	DT	PDSSSSSKRI	DR	TPIVGLSQIDA	PEDGTASTAITVQM	FGG	AL	YQ	AL	SPYV	LFTFV	QARSGG	SVL	LVSGGGF	DTPDSSSSSKRIDRTPIVGLSQIDAPEDGTASTAITVQMFGGALYQALSPYVLFTFVQARSGGSVLLVSGGGF	2	0.20
SA (2021)_MLG61	S America	DT	LDSSSSSRGI	DR	TPIVGLSQIDA	PEDGIASTATTVQM	FSG	AL	YQ	AL	SPYV	LFTFV	QARSGG	SVL	LVSGGGV	DTLDSSSSSRGIDRTPIVGLSQIDAPEDGIASTATTVQMFSGALYQALSPYVLFTFVQARSGGSVLLVSGGGV	1	0.10
SA (2021)_MLG62	S America	DT	PDSLSSSRGI	DR	TPIVGLSQIDA	PEDGTASTATTVQM	FGG	AL	YQ	AL	SPYV	LFTFV	HARSGG	SVL	LVSGGGV	DTPDSLSSSRGIDRTPIVGLSQIDAPEDGTASTATTVQMFGGALYQALSPYVLFTFVHARSGGSVLLVSGGGV	1	0.10
SA (2021)_MLG63	S America	DT	PDSSLSSRGI	DR	TPIVGLSQIDA	PEDGTASTATTVQM	FGG	AL	YQ	AL	SPYV	LFTFV	HARSGG	SVL	LVSGGGV	DTPDSSLSSRGIDRTPIVGLSQIDAPEDGTASTATTVQMFGGALYQALSPYVLFTFVHARSGGSVLLVSGGGV	1	0.10
SA (2021)_MLG64	S America	DT	PDSSSLSRGI	DR	IPIVGLSQIDA	PEDGTASTATTVQM	FGG	AL	YQ	AL	SPYV	LFTFV	HARSGG	SVL	LVNGGGV	DTPDSSSLSRGIDRIPIVGLSQIDAPEDGTASTATTVQMFGGALYQALSPYVLFTFVHARSGGSVLLVNGGGV	1	0.10
SA (2021)_MLG65	S America	DT	PDSSSSSRGI	DR	TPIVGLSQIDA	PEDGTVSTATTVQM	FGG	AL	YQ	AL	SPYV	LFTFV	QARSGG	SVL	LVSGGGV	DTPDSSSSSRGIDRTPIVGLSQIDAPEDGTVSTATTVQMFGGALYQALSPYVLFTFVQARSGGSVLLVSGGGV	1	0.10
SA (2021)_MLG66	S America	DT	PDSSLSSRGI	DR	TPIVGLSQIDA	PEDGTASTATTVQM	FGG	AL	YQ	AL	SPYV	LFTFV	QARSGG	SVL	LVSGGGV	DTPDSSLSSRGIDRTPIVGLSQIDAPEDGTASTATTVQMFGGALYQALSPYVLFTFVQARSGGSVLLVSGGGV	1	0.10
SA (2021)_MLG67	S America	DT	PDSSSSSKPT	DR	TPIVGLSQIDA	PEDGTASTATTVQM	FGG	AL	YQ	AL	SPYV	LFTFV	QARSGG	SVL	LVSGGGV	DTPDSSSSSKPTDRTPIVGLSQIDAPEDGTASTATTVQMFGGALYQALSPYVLFTFVQARSGGSVLLVSGGGV	1	0.10

SA (2021)_MLG68	S America	DT	PDSSSSSKRI	DR	TPIVGLSQIDA	PEDGTASTATTVQM	FGG	AL	YQ	AL	SPYV	LFTFV	QARSGG	SVL	FVSGGGF	DTPDSSSSSKRIDRTPIVGLSQIDAPEDGTASTATTVQMFGGALYQALSPYVLFTFVQARSGGSVLFVSGGGF	1	0.10
SA (2021)_MLG69	S America	DT	PDSSSSSKRI	DR	TPIVGLSQIDA	PEDGTASTATTVQM	FGG	AL	YQ	AL	SPYV	LFTFV	QARSGG	SVL	LVNGGGV	DTPDSSSSSKRIDRTPIVGLSQIDAPEDGTASTATTVQMFGGALYQALSPYVLFTFVQARSGGSVLLVNGGGV	1	0.10
SA (2021)_MLG70	S America	YT	PDSSSSSKRI	DR	TPIVGLSQIDA	PEDGTASTATTVQM	FGG	AL	YQ	AL	SPYV	LFTFV	QARSGG	SVL	LVSGGGF	YTPDSSSSSKRIDRTPIVGLSQIDAPEDGTASTATTVQMFGGALYQALSPYVLFTFVQARSGGSVLLVSGGGF	1	0.10
SA (2021)_MLG71	S America	DT	LDSSSSSKRI	DR	TPIVGLSQIDA	PEDGIASTATTVQM	FGG	AL	YQ	AL	SPYV	LFTFV	QARSGG	SVL	LVSGGGF	DTLDSSSSSKRIDRTPIVGLSQIDAPEDGIASTATTVQMFGGALYQALSPYVLFTFVQARSGGSVLLVSGGGF	1	0.10
SA (2021)_MLG72	S America	DT	PDSSSSSKRI	DR	TPIVGLSQIDA	PEDGTASTATTVQM	FGG	AL	YQ	AL	SPYV	LFTFV	QARSGG	SVL	LVRGGGF	DTPDSSSSSKRIDRTPIVGLSQIDAPEDGTASTATTVQMFGGALYQALSPYVLFTFVQARSGGSVLLVRGGGF	1	0.10
SA (2021)_MLG73	S America	DT	PDSSSSSKRI	DR	TPIVGLSQIDA	PEDGTASTATTVQM	FGG	AL	YQ	AL	SPYV	LFTFV	QARSGG	SVL	FVSGGGV	DTPDSSSSSKRIDRTPIVGLSQIDAPEDGTASTATTVQMFGGALYQALSPYVLFTFVQARSGGSVLFVSGGGV	1	0.10
SA (2021)_MLG74	S America	DT	PDSSSSSKRI	DR	TPIVGLSQIDA	PEDGTASTATTVQM	FGG	AL	YQ	AL	SPYV	LFTFV	QDRSGG	SVL	LVSGGGF	DTPDSSSSSKRIDRTPIVGLSQIDAPEDGTASTATTVQMFGGALYQALSPYVLFTFVQDRSGGSVLLVSGGGF	1	0.10
SA (2021)_MLG75	S America	DT	PYSSSSSKRI	DR	TPIVGLSQIDA	PEDGTASTATTVQM	FGG	AL	YQ	AL	SPYV	LFTFV	QARSGG	SVL	LVSGGGF	DTPYSSSSSKRIDRTPIVGLSQIDAPEDGTASTATTVQMFGGALYQALSPYVLFTFVQARSGGSVLLVSGGGF	1	0.10
SA (2021)_MLG76	S America	DT	PDSSSSSKRT	GR	TPIVGLSQIDA	PEDGTASTATTVQM	FGG	AL	YQ	AL	SPYV	LFTFV	QARSGG	SVL	LVSGGGV	DTPDSSSSSKRTGRTPIVGLSQIDAPEDGTASTATTVQMFGGALYQALSPYVLFTFVQARSGGSVLLVSGGGV	1	0.10
SA (2021)_MLG77	S America	DT	PDSSSSSKRI	DR	TPIVGLSRIDA	PEDGTASTATTVQM	FGG	AL	YQ	AL	SPYV	LFTFV	QARSGG	SVL	FVSGGGF	DTPDSSSSSKRIDRTPIVGLSRIDAPEDGTASTATTVQMFGGALYQALSPYVLFTFVQARSGGSVLFVSGGGF	1	0.10
SA (2021)_MLG78	S America	DT	PDSSSSSKRI	DR	TPIVGLSQIDA	PEDGTASTATTVQM	FGG	AL	YQ	AL	SPYV	LFTFV	QARSGG	LVL	LVSGGGF	DTPDSSSSSKRIDRTPIVGLSQIDAPEDGTASTATTVQMFGGALYQALSPYVLFTFVQARSGGLVLLVSGGGF	1	0.10
SA (2021)_MLG79	S America	DT	PDSSSSSKRT	DR	TPIVGLSQIDA	PEDGTASTVTTVQM	FGG	AL	YQ	AL	SPYV	LFTFV	QARSGG	SVL	LVSGGGV	DTPDSSSSSKRTDRTPIVGLSQIDAPEDGTASTVTTVQMFGGALYQALSPYVLFTFVQARSGGSVLLVSGGGV	1	0.10
SA (2021)_MLG80	S America	DT	LDSSSSSKRI	DR	TPIVGLSQIDA	PEDGTASTVTTVQM	FGG	AL	YQ	AL	SPYV	LFTFV	QARSGG	SVL	LVSGGGF	DTLDSSSSSKRIDRTPIVGLSQIDAPEDGTASTVTTVQMFGGALYQALSPYVLFTFVQARSGGSVLLVSGGGF	1	0.10
SA (2021)_MLG81	S America	DT	PDSSSSSKRI	DR	TPIVGLSQIDA	PEDGTASTATTVQM	FGG	AL	YQ	AL	SPYV	FFTFV	QARSGG	SVL	LVSGGGF	DTPDSSSSSKRIDRTPIVGLSQIDAPEDGTASTATTVQMFGGALYQALSPYVFFTFVQARSGGSVLLVSGGGF	1	0.10
SA (2021)_MLG82	S America	DT	TDSSSSSKRI	DR	TPIVGLSQIDA	PEDGTASTATTVQM	FGG	AL	YQ	AL	SPYV	LFTFV	QARSGG	SVL	LVSGGGF	DTTDSSSSSKRIDRTPIVGLSQIDAPEDGTASTATTVQMFGGALYQALSPYVLFTFVQARSGGSVLLVSGGGF	1	0.10
SA (2021)_MLG83	S America	DT	LDSSSSSKRI	DR	TPIVGLSQIDA	PEDGTASTATTVQM	FGG	AF	YQ	AL	SPYV	LFTFV	QARSGG	SVL	LVSGGGF	DTLDSSSSSKRIDRTPIVGLSQIDAPEDGTASTATTVQMFGGAFYQALSPYVLFTFVQARSGGSVLLVSGGGF	1	0.10
SA (2021)_MLG84	S America	DT	PDSSSSSKRI	DR	TPIVGLSQIDA	PEDGTASTATTVQM	FGG	AL	YQ	AL	SPYV	LFTFA	QARSGG	SVL	LVSGGGF	DTPDSSSSSKRIDRTPIVGLSQIDAPEDGTASTATTVQMFGGALYQALSPYVLFTFAQARSGGSVLLVSGGGF	1	0.10
SA (2021)_MLG85	S America	DT	PYSSSSSKRT	DR	TPIVGLSQIDA	PEDGTASTATTVQM	FGG	AL	YQ	AL	SPYV	LFTFV	QARSGG	SVL	LVSGGGV	DTPYSSSSSKRTDRTPIVGLSQIDAPEDGTASTATTVQMFGGALYQALSPYVLFTFVQARSGGSVLLVSGGGV	1	0.10
SA (2021)_MLG86	S America	DT	PDSSSSSKRI	DR	TPIVGLSQIDA	PEDGTASTATTVQM	FGG	AL	YQ	AL	SLYV	LFTFV	QARSGG	SVL	LVSGGGF	DTPDSSSSSKRIDRTPIVGLSQIDAPEDGTASTATTVQMFGGALYQALSLYVLFTFVQARSGGSVLLVSGGGF	1	0.10
SA (2021)_MLG87	S America	DT	PDSSSSSKRT	DR	TPIVGLSQIDA	PEDGTASTATTVQM	FGG	AL	YQ	AL	SPYV	LFTFV	QARSGG	SVL	LVSGGVV	DTPDSSSSSKRTDRTPIVGLSQIDAPEDGTASTATTVQMFGGALYQALSPYVLFTFVQARSGGSVLLVSGGVV	1	0.10
SA (2021)_MLG88	S America	DT	PDSSSSSKRT	DR	TPIVGLSQIDA	PEDGTASTATTVQM	FGG	AL	YQ	AL	SPYV	LFTFV	QARSGG	SVL	LVSGGGF	DTPDSSSSSKRTDRTPIVGLSQIDAPEDGTASTATTVQMFGGALYQALSPYVLFTFVQARSGGSVLLVSGGGF	1	0.10
SA (2021)_MLG89	S America	DT	PDSSSSSKRI	DR	TPIVGLSQIDA	PEDGTASTATTVQM	FGG	AL	YQ	AL	SPYV	LFTFV	QARSGG	SFL	LVSGGGF	DTPDSSSSSKRIDRTPIVGLSQIDAPEDGTASTATTVQMFGGALYQALSPYVLFTFVQARSGGSFLLVSGGGF	1	0.10
SA (2021)_MLG90	S America	DT	PDSSSSSKRI	DR	TPIVGLSQIDA	PEDGTASTATTVQM	FGG	AL	YQ	AL	SPYV	FFTFV	QARSGG	SVL	LVSGGGV	DTPDSSSSSKRIDRTPIVGLSQIDAPEDGTASTATTVQMFGGALYQALSPYVFFTFVQARSGGSVLLVSGGGV	1	0.10
SA (2021)_MLG91	S America	VT	PDSSSSSKRI	DR	TPIVGLSQIDA	PEDGTASTATTVQM	FGG	AL	YQ	AL	SPYV	LFTFV	QARSGG	SVL	LVSGGGF	VTPDSSSSSKRIDRTPIVGLSQIDAPEDGTASTATTVQMFGGALYQALSPYVLFTFVQARSGGSVLLVSGGGF	1	0.10
SA (2021)_MLG92	S America	DT	PDSSSSSKRI	DR	TPIVSLSQIDA	PEDGTASTATTVQM	FGG	AL	YQ	AL	SPYV	LFTFV	QARSGG	SVL	LVSGGGV	DTPDSSSSSKRIDRTPIVSLSQIDAPEDGTASTATTVQMFGGALYQALSPYVLFTFVQARSGGSVLLVSGGGV	1	0.10
SA (2021)_MLG93	S America	DT	PDSSSSSKRI	DR	TPIVGLSQIDA	PEDGTASTATTVQM	FGG	AL	YQ	AL	SPYV	LFTFV	QARFGG	SVL	LVSGGGF	DTPDSSSSSKRIDRTPIVGLSQIDAPEDGTASTATTVQMFGGALYQALSPYVLFTFVQARFGGSVLLVSGGGF	1	0.10
SA (2021)_MLG94	S America	DT	PDSSSSSKRI	DR	TPIVGLSQIDA	PEDGTASTATTVQM	FGG	AL	YQ	AL	SHYV	LFTFV	QARSGG	SVL	LVSGGGF	DTPDSSSSSKRIDRTPIVGLSQIDAPEDGTASTATTVQMFGGALYQALSHYVLFTFVQARSGGSVLLVSGGGF	1	0.10
SA (2021)_MLG95	S America	DT	PDSSSSSKRI	DR	TPIVGLSQIDA	PEDGTASTATTVQM	FGG	AL	YQ	AL	SSYV	LFTFV	QARSGG	SVL	LVSGGGF	DTPDSSSSSKRIDRTPIVGLSQIDAPEDGTASTATTVQMFGGALYQALSSYVLFTFVQARSGGSVLLVSGGGF	1	0.10
SA (2021)_MLG96	S America	DT	PDSSSSSKRI	DR	TPIVGLSQIDA	PEDGTASTATTVQM	FGG	AF	YQ	AL	SPYV	LFTFV	QARSGG	SLL	LVSGGGF	DTPDSSSSSKRIDRTPIVGLSQIDAPEDGTASTATTVQMFGGAFYQALSPYVLFTFVQARSGGSLLLVSGGGF	1	0.10
SA (2021)_MLG97	S America	DT	PDSSSSSMGI	DR	TPIVGLSQIDA	PEDGTASTATTVQM	FGG	AL	YQ	AL	SPYV	LFTFV	QARSGG	SVL	LVSGGGV	DTPDSSSSSMGIDRTPIVGLSQIDAPEDGTASTATTVQMFGGALYQALSPYVLFTFVQARSGGSVLLVSGGGV	1	0.10
