# Supplementary material for: Contrasting Epidemiology and Population Genetics of COVID-19 Infections Defined by Multilocus Genotypes in SARS-CoV-2 Genomes Sampled Globally
Source: Viruses. 2022 Jun 29;14(7):1434. doi: 10.3390/v14071434 (PMC9316073; doi:10.3390/v14071434)
Supplement: Supplementary file 1 [file viruses-14-01434-s001.zip › Chan et al_Supplementary data_ver03-05-22_Viruses.pdf]

Supplementary data

Table S1. Pairwise t-test (Holms Corrected) for difference in the proportion of genomes sampled among the four age groups in the different geographical regions.

	Africa	Asia	Europe	North America	Oceania
Asia	0.0001	-	-	-	-
Europe	0.0001	0.0001	-	-	-
North America	0.0001	0.00015	0.0001	-	-
Oceania	0.0001	0.00214	0.0001	0.3015	-
South America	0.0001	0.00559	0.0001	0.84368	0.53861

P-value < 0.05 was considered statistically significant.

Table S2. Pairwise t-test (Holms Corrected) for difference in the proportion of genomes sampled among the genders in the different geographical regions.

	Africa	Asia	Europe	North America	Oceania
Asia	0.0001	-	-	-	-
Europe	0.0242	0.0001	-	-	-
North America	0.0001	0.1939	0.032	-	-
Oceania	0.0001	0.0001	0.4786	0.4786	-
South America	0.0184	0.017	0.6614	0.655	0.9538

P-value < 0.05 was considered statistically significant.

Table S3. The 74 loci/codons considered polymorphic and included in the study.

Genetic diversity in genes coding for non-structural proteins					
Protein	Gene	Codon	No of alleles	He	E.5
NSP1	ORF1ab	75	2	0.02	0.36
	ORF1ab	207	3	0.01	0.32
NSP2	ORF1ab	265	3	0.24	0.63
	ORF1ab	271	3	0.02	0.35
	ORF1ab	300	2	0.02	0.36
	ORF1ab	378	2	0.05	0.42
	ORF1ab	392	3	0.01	0.31
	ORF1ab	454	2	0.01	0.33
	ORF1ab	481	2	0.01	0.33
	ORF1ab	676	3	0.02	0.36
	ORF1ab	739	2	0.09	0.47
	ORF1ab	765	2	0.02	0.35
	ORF1ab	876	4	0.01	0.30
NSP3	ORF1ab	944	2	0.01	0.32
	ORF1ab	971	4	0.03	0.34
	ORF1ab	972	2	0.00	0.21
	ORF1ab	1036	4	0.02	0.33
	ORF1ab	1100	3	0.03	0.37
	ORF1ab	1246	2	0.03	0.38
	ORF1ab	1812	3	0.04	0.39
	ORF1ab	2015	4	0.04	0.39
	ORF1ab	2016	3	0.08	0.46
	ORF1ab	2129	3	0.03	0.37
	ORF1ab	2153	2	0.01	0.30
	ORF1ab	2202	4	0.01	0.31
	ORF1ab	2586	2	0.02	0.34
	ORF1ab	2702	2	0.02	0.34
	ORF1ab	2796	5	0.01	0.30
NSP4	ORF1ab	3071	2	0.10	0.49
	ORF1ab	3278	2	0.03	0.38
	ORF1ab	3334	3	0.02	0.34
NSP5	ORF1ab	3529	2	0.01	0.31
	ORF1ab	3606	3	0.21	0.60
NSP8	ORF1ab	4080	2	0.03	0.38
	ORF1ab	4140	2	0.01	0.32
NSP12	ORF1ab	4489	2	0.09	0.47
	ORF1ab	4715	4	0.42	0.85
NSP13	ORF1ab	5809	3	0.01	0.33
	ORF1ab	5828	3	0.04	0.40
	ORF1ab	5865	3	0.04	0.40
	ORF1ab	5894	2	0.00	0.27
NSP14	ORF1ab	6102	2	0.02	0.36
	ORF1ab	6158	2	0.02	0.35
	ORF1ab	6297	2	0.01	0.34
	ORF1ab	6302	2	0.00	0.21
	ORF1ab	6474	3	0.01	0.33

Genetic diversity in genes coding for structural and accessory proteins					
Protein	Gene	Codon	No of alleles	He	E.5
Membrane (M)	M	3	3	0.03	0.36
	M	175	2	0.02	0.35
Nucleocapsid (N)	N	13	2	0.11	0.50
	N	22	4	0.01	0.29
	N	183	3	0.00	0.23
	N	187	2	0.02	0.34
	N	194	3	0.07	0.44
	N	197	3	0.10	0.48
	N	202	4	0.03	0.37
	N	203	5	0.39	0.80
	N	204	4	0.38	0.80
	N	292	3	0.03	0.38
Spike glycoprotein (S)	S	54	2	0.03	0.38
	S	320	5	0.04	0.39
	S	477	5	0.02	0.32
	S	485	2	0.01	0.31
	S	614	3	0.41	0.84
	S	1124	2	0.02	0.36
	S	1176	2	0.02	0.34
ORF3a	ORF3a	57	2	0.37	0.78
	ORF3a	99	3	0.01	0.31
	ORF3a	126	3	0.02	0.34
	ORF3a	165	3	0.01	0.31
	ORF3a	196	2	0.10	0.49
	ORF3a	251	5	0.09	0.43
ORF8	ORF8	24	4	0.05	0.40
	ORF8	62	5	0.03	0.36
	ORF8	84	3	0.21	0.60

Table S4. Genetic diversity within genes in SARS-CoV-2. The genetic diversity parameters are indicated by h (number of haplotypes), He (heterozygosity), evenness score (E.5) and genetic differentiation index (Gst).

Gene	h	*He*	E.5	Gst (95% CI)
NSP1	4	0.04	0.34	0.014 (0.009-0.019)
NSP2	25	0.43	0.43	0.064 (0.053-0.075)
NSP3	47	0.30	0.29	0.050 (0.044-0.056)
NSP4	5	0.15	0.43	0.125 (0.097-0.153)
NSP5	4	0.22	0.57	0.065 (0.053-0.077)
NSP8	3	0.04	0.36	0.117 (0.095-0.138)
NSP12	6	0.44	0.71	0.127 (0.113-0.140)
NSP13	9	0.06	0.35	0.023 (0.016-0.030)
NSP14	9	0.05	0.30	0.021 (0.016-0.026)
ORF3a	19	0.53	0.59	0.082 (0.070-0.095)
ORF8	13	0.26	0.49	0.145 (0.123-0.166)
Membrane (M)	4	0.05	0.35	0.015 (0.010-0.020)
Nucleocapsid (N)	30	0.60	0.54	0.100 (0.089-0.112)
Spike (S)	17	0.50	0.61	0.117 (0.104-0.129)
Mean overall	14	0.26	0.46	0.094 (0.089-0.099)

The genetic diversity parameters are indicated by h (number of haplotypes), He (heterozygosity), evenness score (E.5) and genetic differentiation index (Gst). 95% confidence interval (95% CI)

Table S5. Unique MLGs. Data attached in a separate pdf file.

Table S6. Private alleles in each geographical region.

Gene	Gene	Codon/locus	Allele/mutation	Africa	Asia	Europe	North America	Oceania	South America
ORF1ab	NSP1	207	R -> G	-	-	0.1	-	-	-
ORF1ab	NSP1	265	T -> N	0.2	-	-	-	-	-
ORF1ab	NSP2	271	P -> S	-	-	-	-	4.4	-
ORF1ab	NSP2	392	G -> C	-	-	0.2	-	-	-
ORF1ab	NSP2	676	Q -> P	-	4.5	-	-	-	-
ORF1ab	NSP2	676	Q -> R	-	-	-	0.2	-	-
ORF1ab	NSP2	876	A -> P	-	0.1	-	-	-	-
ORF1ab	NSP2	876	A -> V	-	-	0.1	-	-	-
ORF1ab	NSP3	971	P -> Q	-	0.1	-	-	-	-
ORF1ab	NSP3	972	E -> V	-	0.1	-	-	-	-
ORF1ab	NSP3	1036	D -> Y	0.2	-	-	-	-	-
ORF1ab	NSP3	1036	D -> G	-	0.1	-	-	-	-
ORF1ab	NSP3	1100	G -> A	-	-	-	-	0.1	-
ORF1ab	NSP3	1812	A -> D	-	7.4	-	-	-	-
ORF1ab	NSP3	1812	A -> V	-	-	-	-	0.1	-
ORF1ab	NSP3	2015	S -> G	-	0.1	-	-	-	-
ORF1ab	NSP3	2015	S -> N	-	0.1	-	-	-	-
ORF1ab	NSP3	2129	A -> G	-	-	-	0.2	-	-
ORF1ab	NSP3	2153	T -> I	-	-	-	-	1.4	-
ORF1ab	NSP3	2202	T -> A	0.2	-	-	-	-	-
ORF1ab	NSP3	2202	T -> N	-	0.1	-	-	-	-
ORF1ab	NSP3	2586	V -> G	-	3.1	-	-	-	-
ORF1ab	NSP3	2796	M -> T	-	0.1	-	-	-	-
ORF1ab	NSP4	3334	G -> V	-	-	-	-	0.1	-
ORF1ab	NSP5	3529	A -> V	-	-	-	-	1.7	-
ORF1ab	NSP5	3606	L -> C	-	-	-	-	0.1	-
ORF1ab	NSP8	4080	Y -> H	15.6	-	-	-	-	-
ORF1ab	NSP12	4715	P -> T	-	-	0.1	-	-	-
ORF1ab	NSP12	4715	P -> S	-	-	0.1	-	-	-
ORF1ab	NSP13	5809	S -> P	-	-	-	-	0.1	-
ORF1ab	NSP13	5865	Y -> F	-	-	-	0.2	-	-
ORF1ab	NSP14	6297	T -> I	-	2.8	-	-	-	-
ORF1ab	NSP14	6302	F -> L	-	0.1	-	-	-	-
ORF1ab	NSP14	6474	V -> I	0.2	-	-	-	-	-
ORF3a	ORF3a	99	A -> S	-	0.1	-	-	-	-
ORF3a	ORF3a	165	S -> A	-	-	-	-	-	0.6
ORF3a	ORF3a	251	G -> D	0.5	-	-	-	-	-
ORF3a	ORF3a	251	G -> S	-	-	-	5.4	-	-
ORF3a	ORF3a	251	G -> C	-	-	-	-	0.1	-
ORF8	ORF8	24	S -> A	-	-	-	0.2	-	-
ORF8	ORF8	24	S -> P	-	-	-	0.2	-	-
ORF8	ORF8	62	V -> E	-	-	-	0.2	-	-
ORF8	ORF8	62	V -> F	-	-	-	-	0.1	-
ORF8	ORF8	62	V -> A	-	-	-	-	-	0.6
ORF8	ORF8	84	L -> F	-	0.1	-	-	-	-
Membrane	M	3	D -> Y	-	-	-	-	0.1	-
Nucleocapsid	N	22	D -> N	-	0.1	-	-	-	-
Nucleocapsid	N	183	S -> F	-	0.1	-	-	-	-
Nucleocapsid	N	187	S -> L	8.5	-	-	-	-	-
Nucleocapsid	N	194	S -> A	-	-	-	-	0.1	-
Nucleocapsid	N	197	S -> P	-	-	-	-	0.1	-
Nucleocapsid	N	202	S -> C	-	-	0.1	-	-	-
Nucleocapsid	N	202	S -> I	-	-	0.1	-	-	-
Nucleocapsid	N	203	R -> G	-	0.1	-	-	-	-
Nucleocapsid	N	203	R -> M	-	-	0.1	-	-	-
Nucleocapsid	N	204	G -> T	-	0.1	-	-	-	-
Nucleocapsid	N	204	G -> L	-	-	0.1	-	-	-
Nucleocapsid	N	292	I -> V	0.2	-	-	-	-	-
Spike	S	320	V -> G	-	-	10.7	-	-	-
Spike	S	320	V -> F	-	-	0.1	-	-	-
Spike	S	320	V -> I	-	-	-	0.2	-	-
Spike	S	320	V -> A	-	-	-	-	0.1	-
Spike	S	477	S -> R	0.2	-	-	-	-	-
Spike	S	477	S -> G	-	-	0.1	-	-	-
Spike	S	485	G -> R	-	-	-	-	1.6	-
Spike	S	614	D -> E	-	-	-	-	0.1	-
Total			66	9	19	12	8	16	2

The numbers represent the proportion of genomes with the allele.

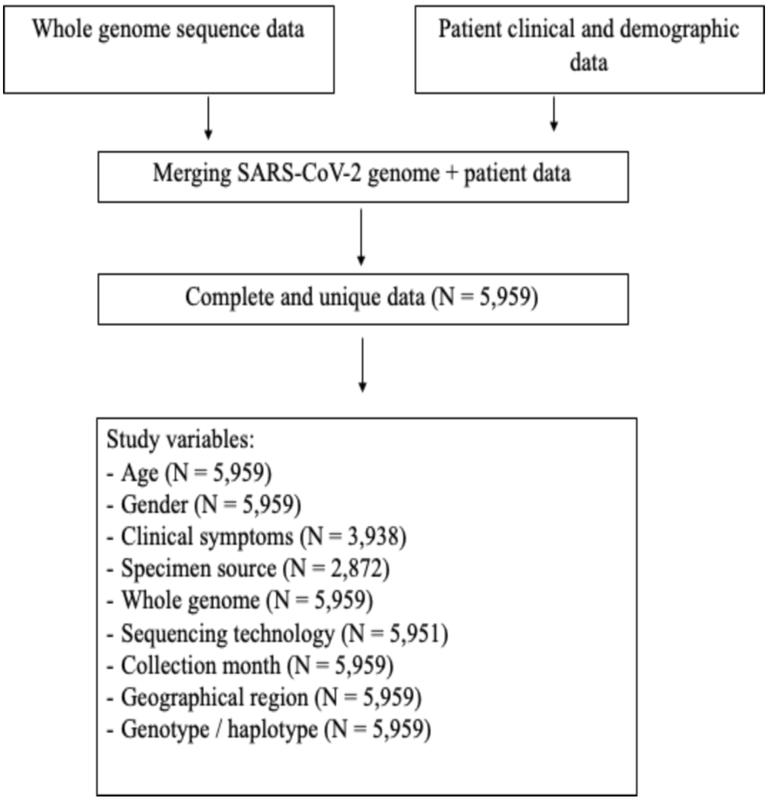

Figure S1. Workflow for data acquisition and cleaning prior to analysis.

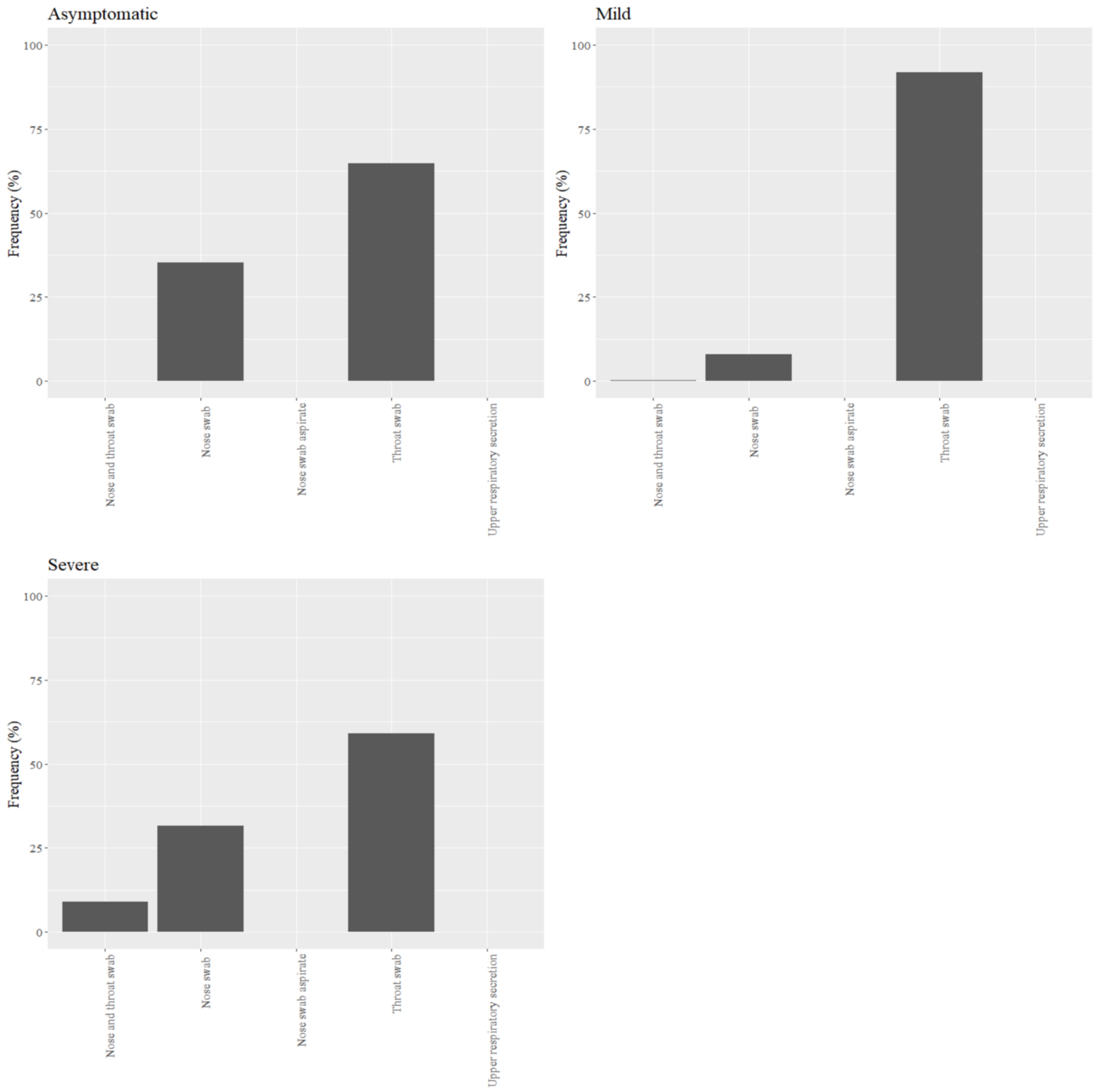

Figure S2. The proportion of genomes isolated using the different upper respiratory tract sample types by COVID-19 disease phenotype in the global population.

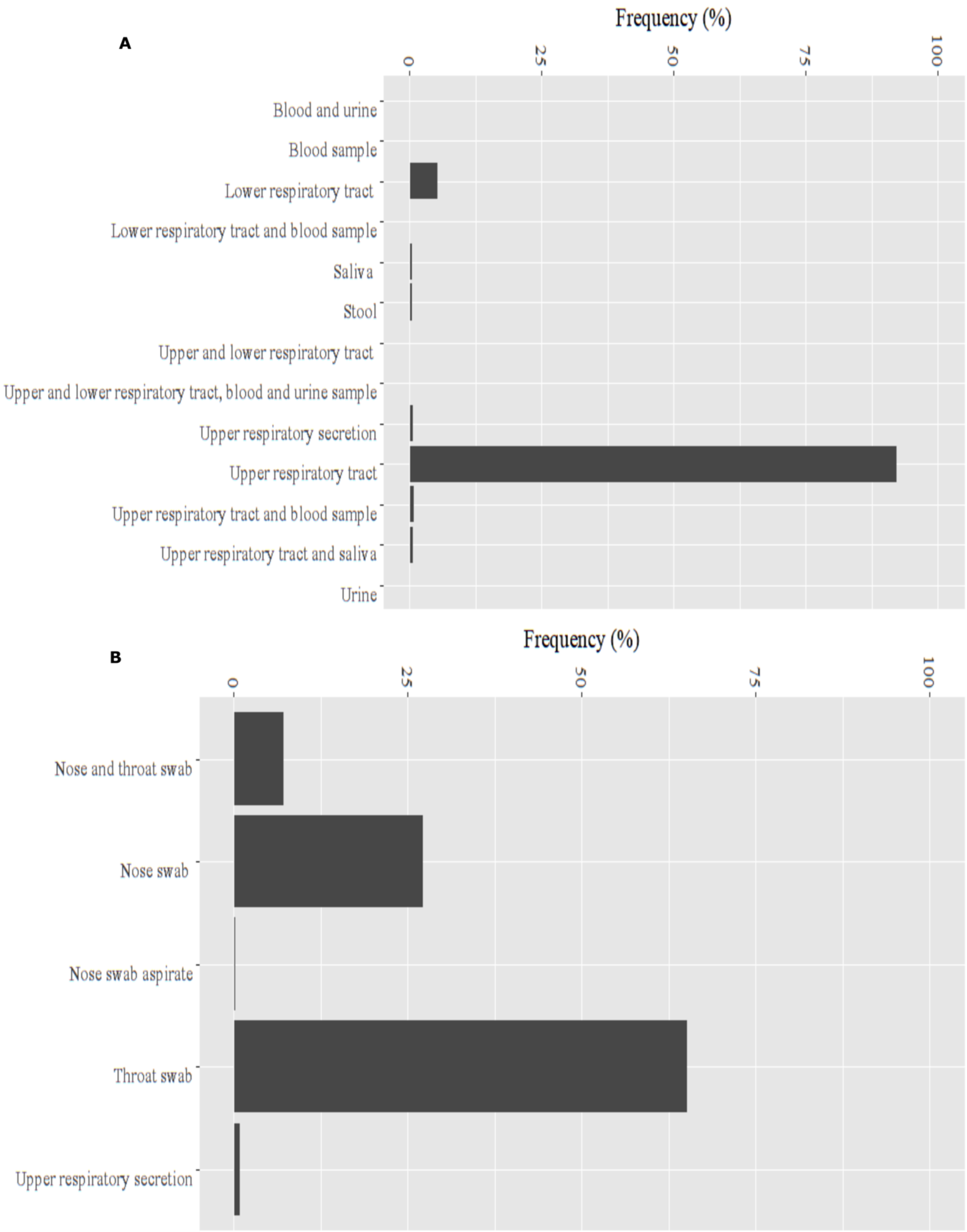

Figure S3. Specimen types collected for SARS-CoV-2 diagnosis and genome isolation. Upper graph details the proportion of genomes isolated using 13 specimen types. Lower graph details the proportion of genomes for the different upper respiratory tract (URT) sample types.

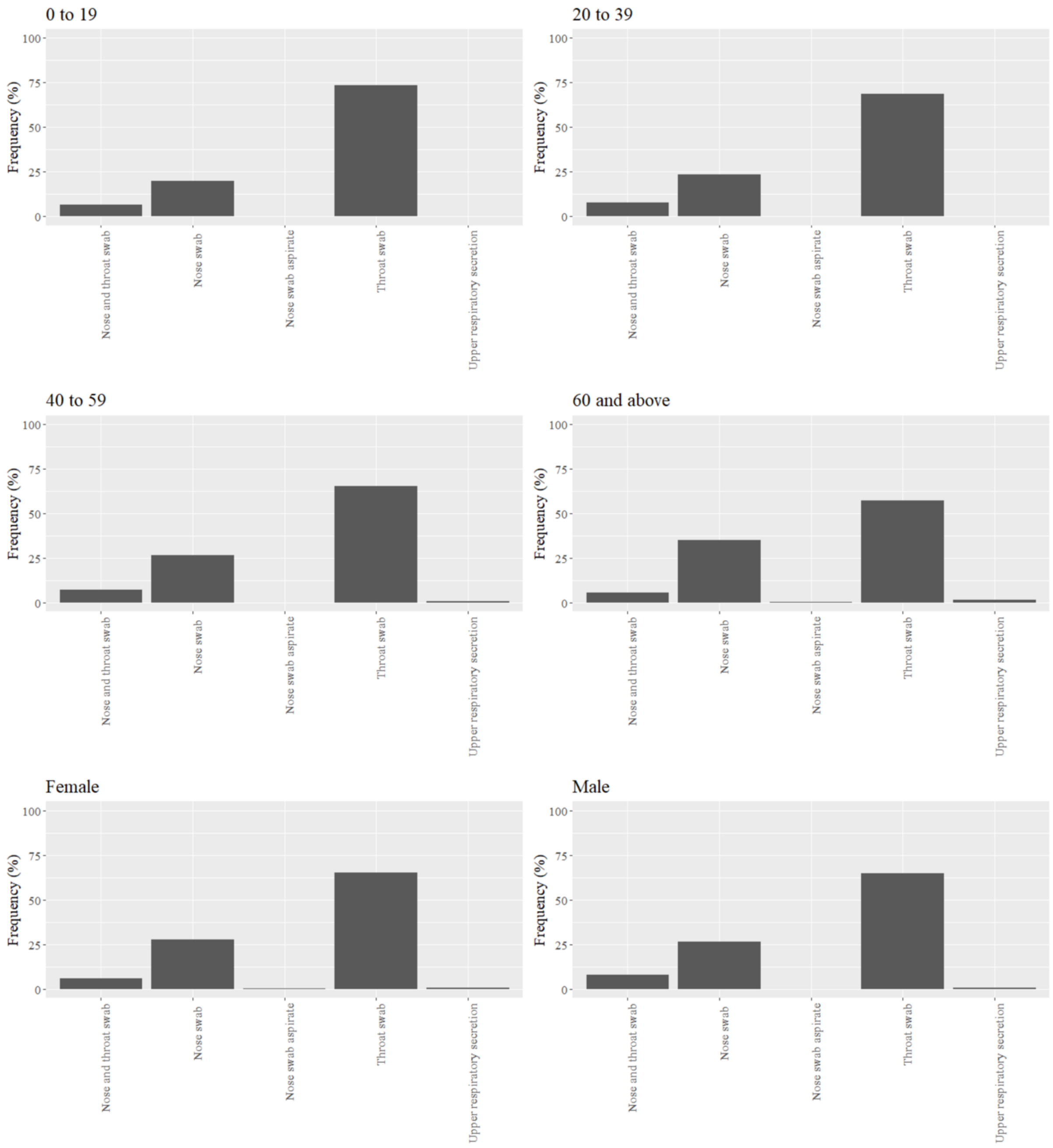

Figure S4. The proportion of genomes isolated using the different upper respiratory tract sample types by age groups and gender in the global population.

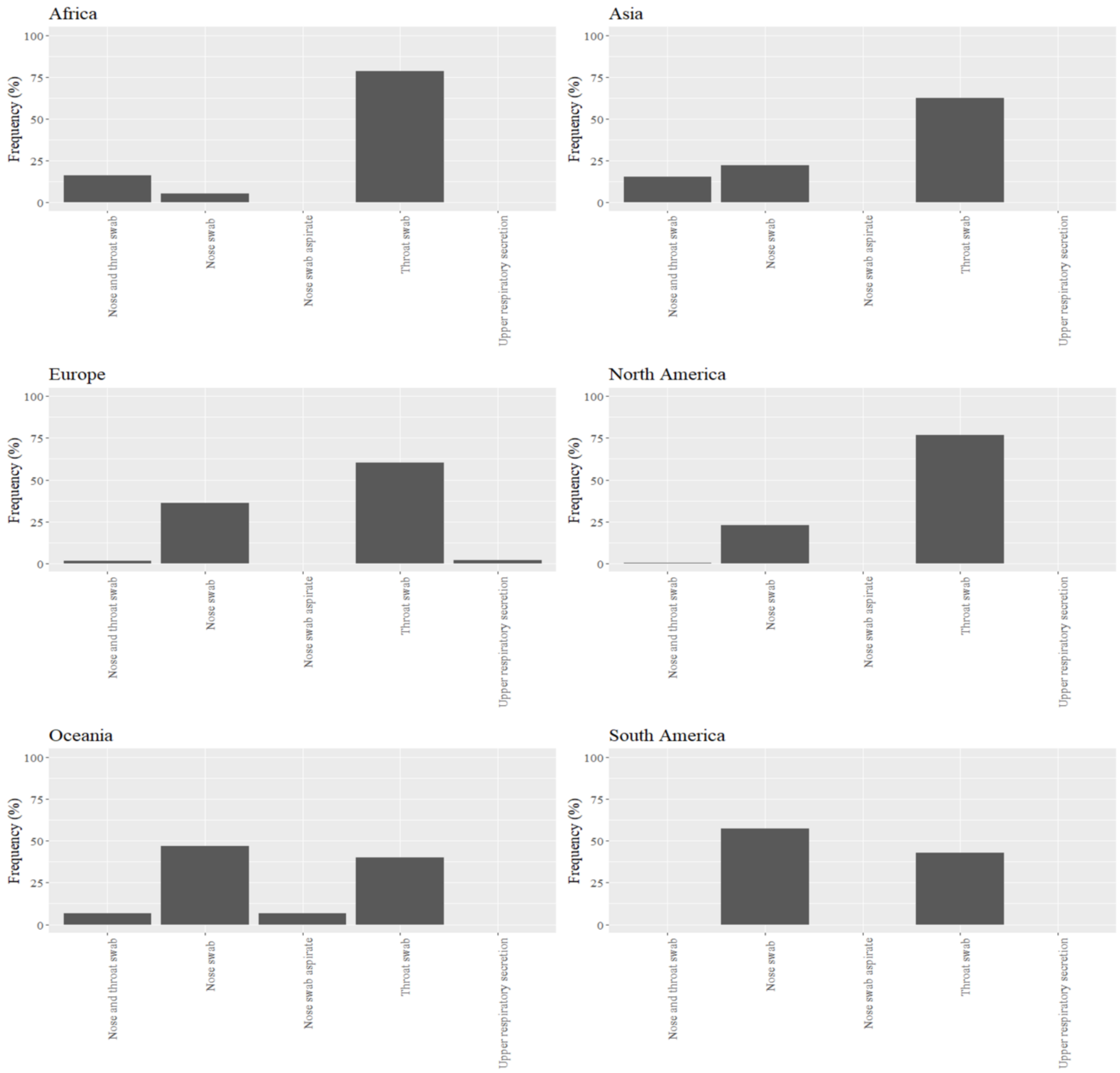

Figure S5. The proportion of genomes isolated using the different upper respiratory tract sample types by continent.

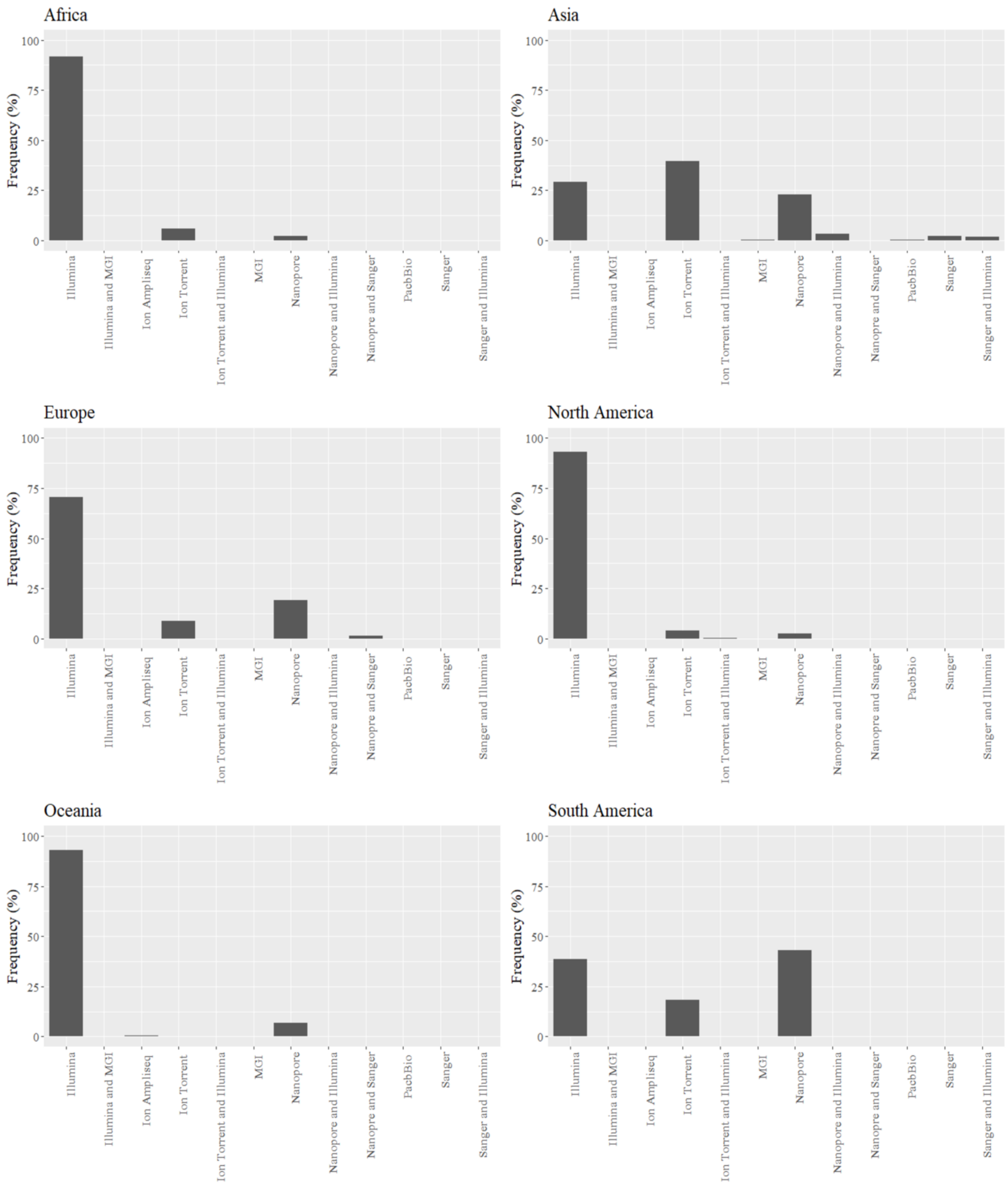

Figure S6. The proportion of genomes sequenced using the different sequencing technologies or platforms is shown for each continent.

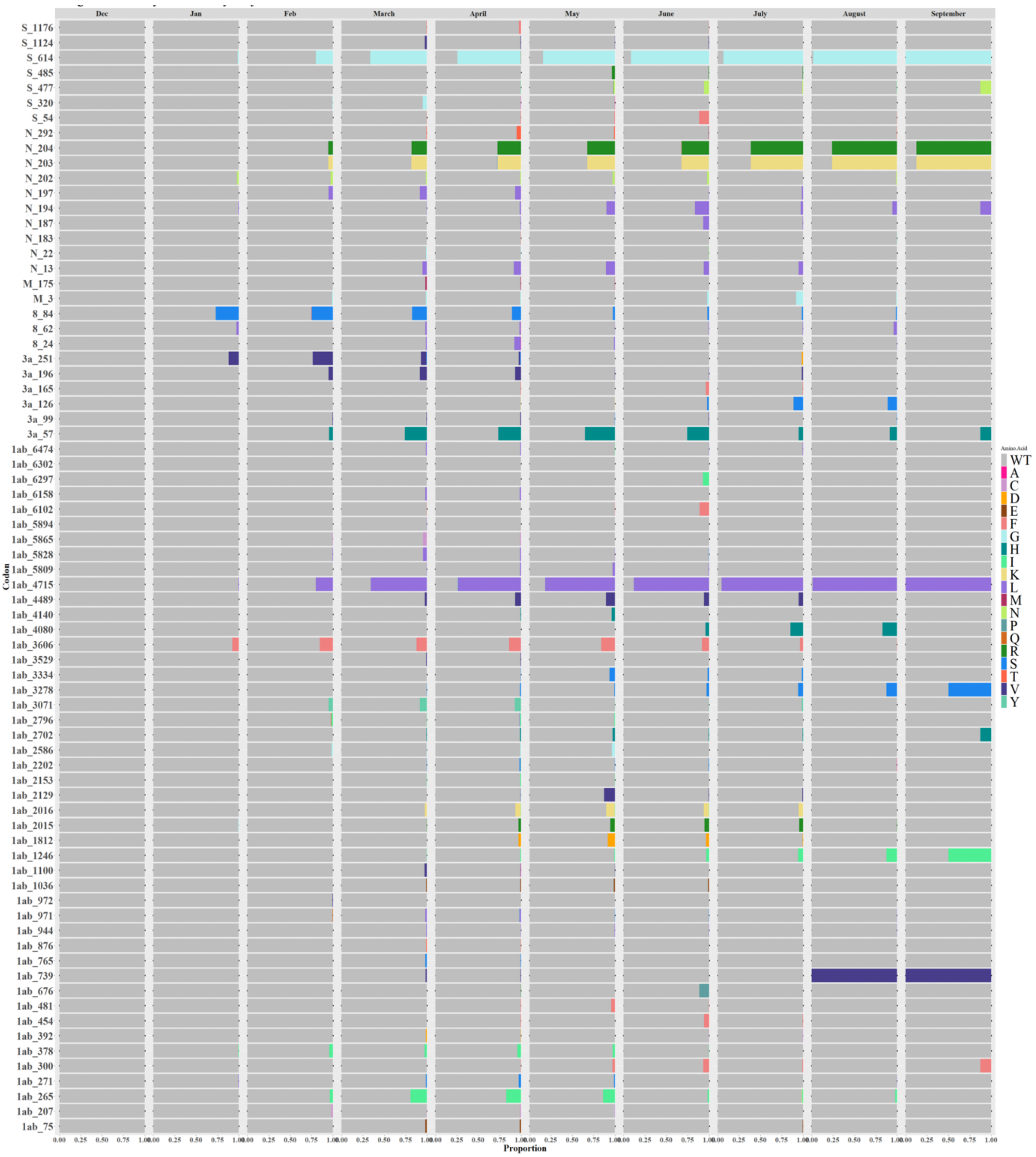

Figure S7. Longitudinal prevalence of alleles detected at the 74 loci in the global SARS-CoV-2 population sampled from December 2019 to September 2020.

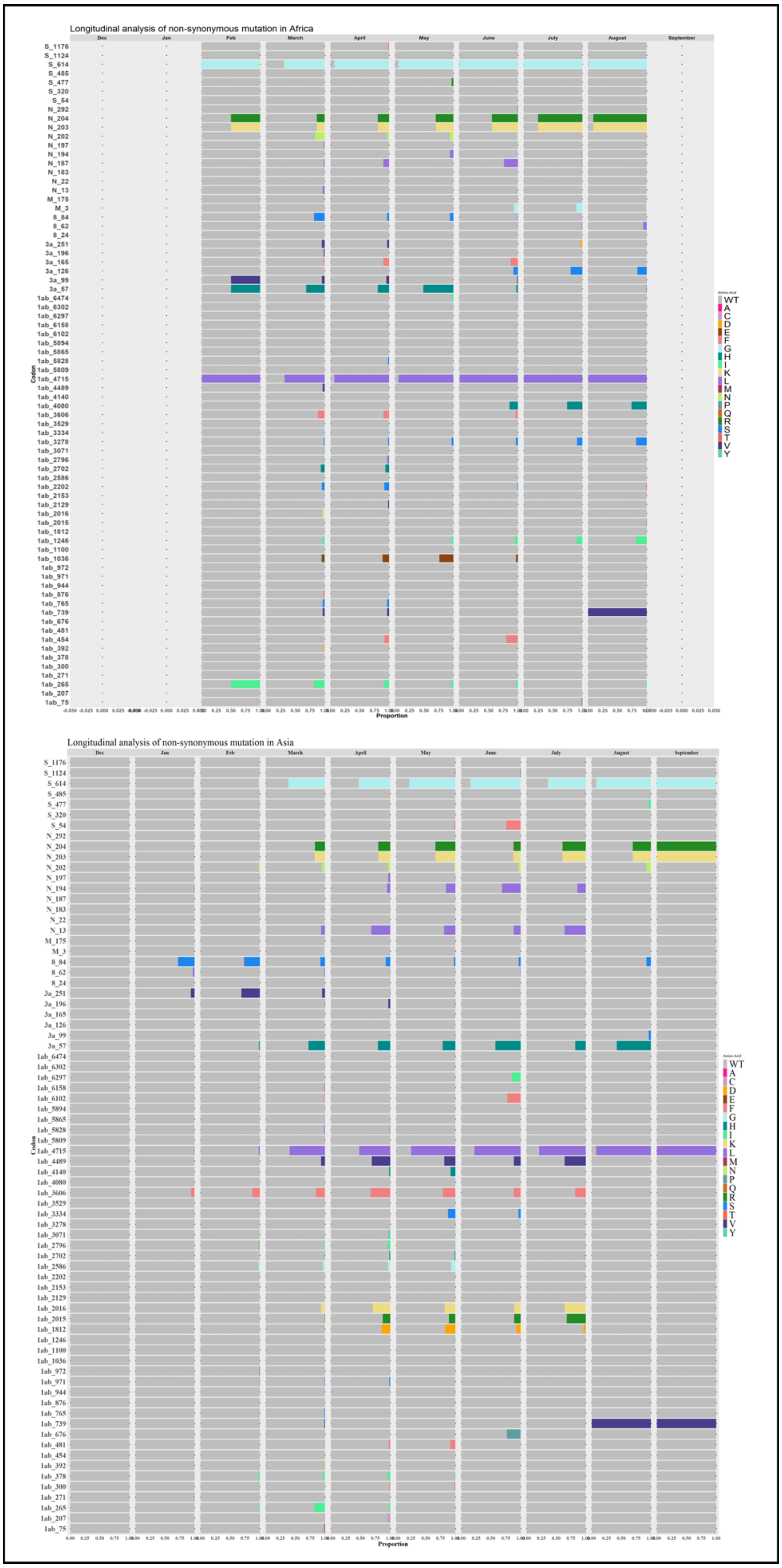

Figure S8. Longitudinal prevalence of alleles detected at the 74 loci in Africa and Asia SARS-CoV-2 populations sampled from December 2019 to September 2020.

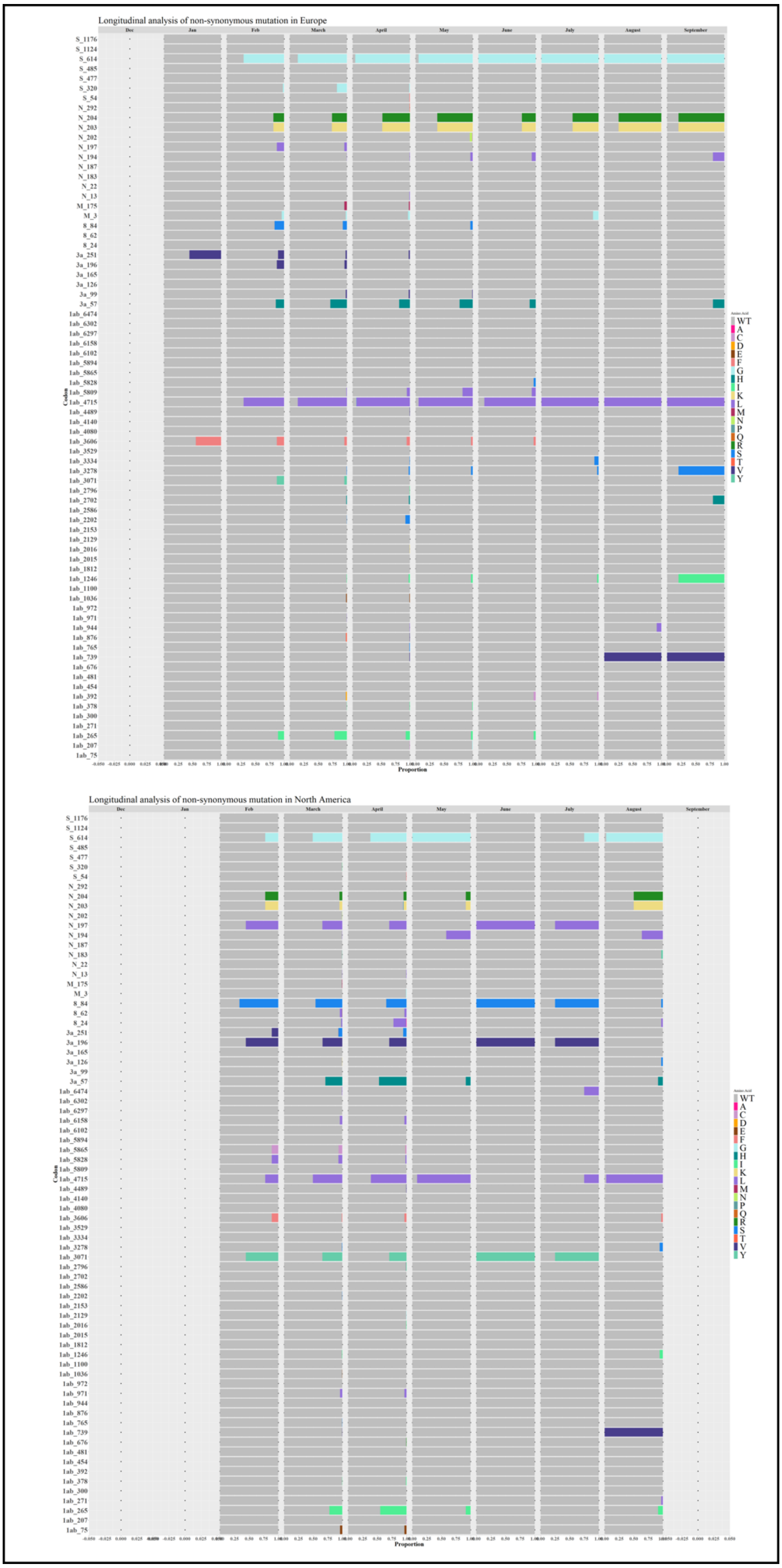

Figure S9. Longitudinal prevalence of alleles detected at the 74 loci in Europe and North America SARS-CoV-2 populations sampled from December 2019 to September 2020.

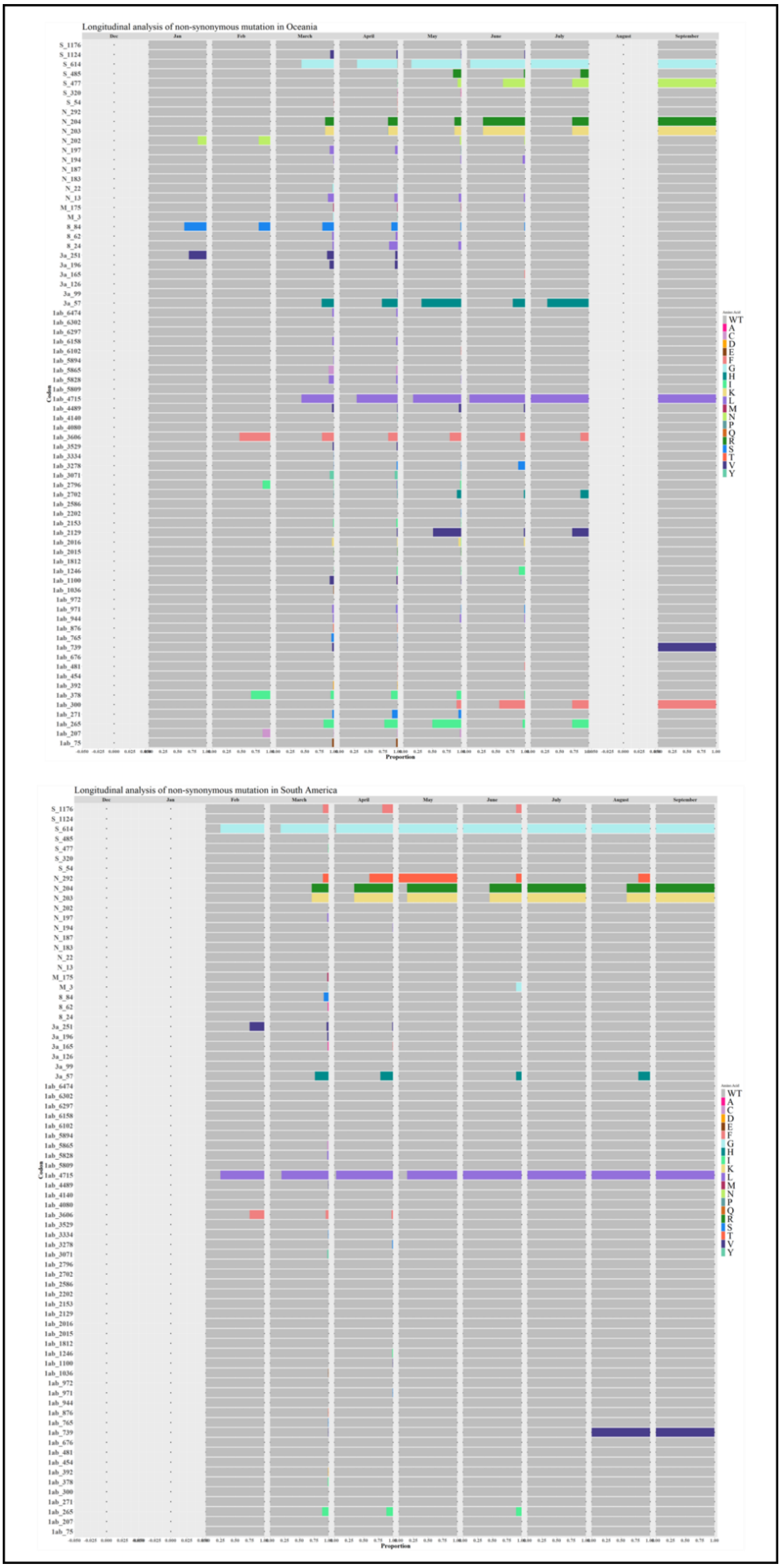

Figure S10. Longitudinal prevalence of alleles detected at the 74 loci in Oceania and South America SARS-CoV-2 populations sampled from December 2019 to September 2020.

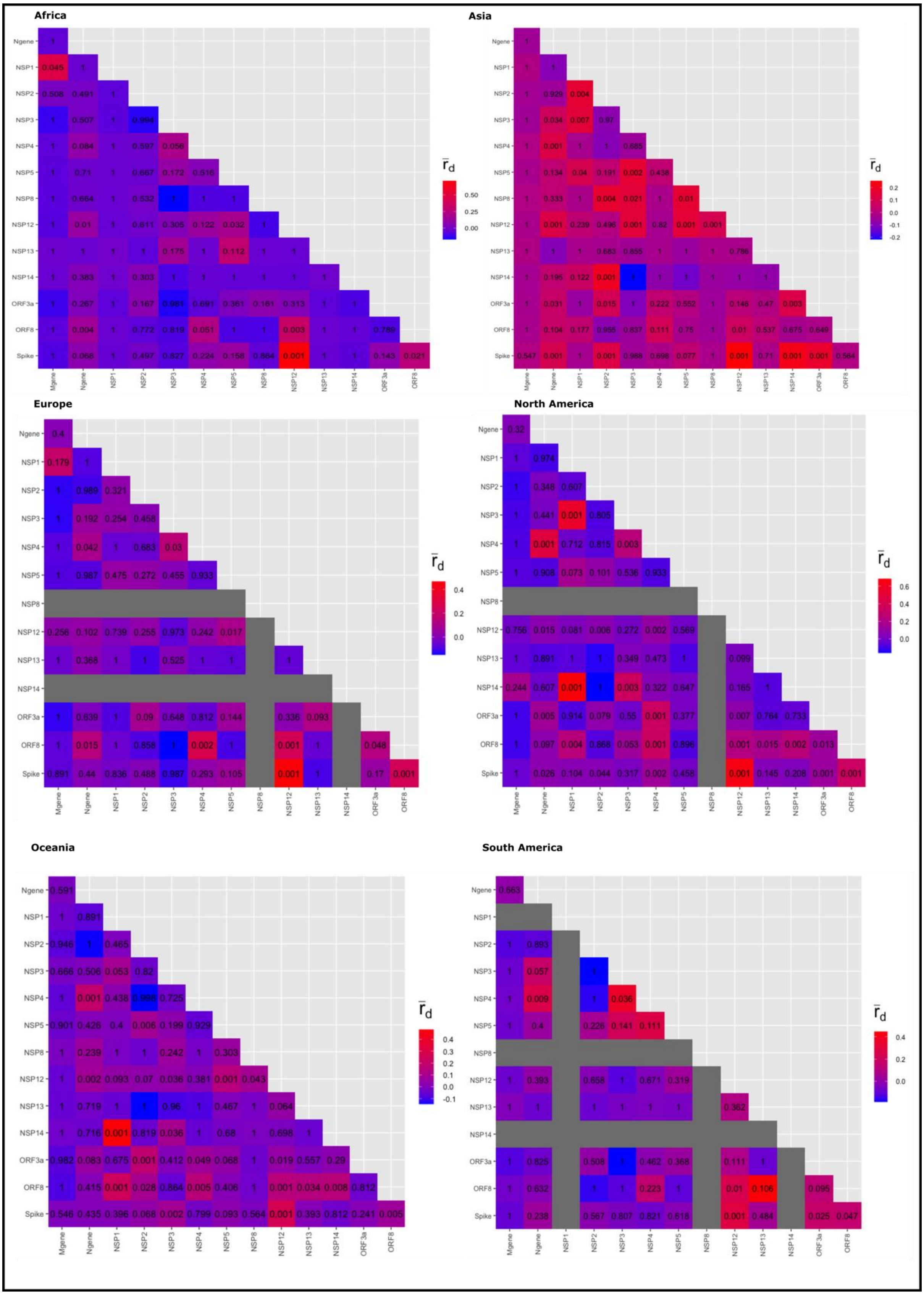

Figure S11. Pairwise LD estimates among genes in the genomes sampled per continent. The $\bar{r}d$ score ranges from 0 (no LD) to 1 (complete LD). The values in the heatmap indicate the P-value associated with the pairwise $\bar{r}d$ estimates. The NSP12 and S LD signal ($\bar{r}d \geq 0.3$, P-value < 0.001) was prominent in all continents.

Africa

	North Africa	Central Africa	West Africa
Central Africa	0.217		
West Africa	0.151	0.227	
South Africa	0.327	0.374	0.216

Genetic Diff.	Gst
Little	≤ 0.09
Moderate	0.1 - 0.19
Great	≥ 0.2

Asia

	China	Central Asia	East Asia	South Asia	Southeast Asia
Central Asia	0.396				
East Asia	0.135	0.392			
South Asia	0.364	0.503	0.197		
Southeast Asia	0.241	0.400	0.151	0.152	
Western Asia	0.430	0.543	0.227	0.111	0.283

Europe

	Eastern Europe	Northern Europe	Southern Europe
Northern Europe	0.248		
Southern Europe	0.076	0.069	
Western Europe	0.181	0.174	0.107

Figure S12. Genetic differentiation within Africa, Asia and Europe.
